# MarkerMatch: a proximity-based probe-matching algorithm for joint analysis of copy-number variants from different genotyping arrays

**DOI:** 10.1093/bioinformatics/btag341

**Published:** 2026-05-24

**Authors:** Franjo Ivankovic, Dongmei Yu, James Shen, Lingyu Zhan, Maria Niarchou, Ariadne Kaylor, Laura Domènech, Tyne W Miller-Fleming, Luz M Porras, Paola Giusti-Rodríguez, Roel A Ophoff, Jeremiah M Scharf, Carol A Mathews

**Affiliations:** Department of Psychiatry, Center for OCD, Anxiety, and Related Disorders, McKnight Brain Institute, College of Medicine, University of Florida, Gainesville, FL 32603, United States; University of Florida Genetics Institute, Genetics and Genomics Graduate Program, College of Medicine, University of Florida, Gainesville, FL 32610, United States; Analytic and Translational Genetics Unit, Massachusetts General Hospital, Boston, MA 02114, United States; Stanley Center for Psychiatric Research, Broad Institute of MIT and Harvard, Cambridge, MA 02142, United States; Program in Brain Health, Molecular and Population Genetics Program, Broad Institute of MIT and Harvard, Cambridge, MA 02142, United States; Stanley Center for Psychiatric Research, Broad Institute of MIT and Harvard, Cambridge, MA 02142, United States; Psychiatric and Neurodevelopmental Genetics Unit, Departments of Psychiatry and Neurology, Center for Genomic Medicine, Massachusetts General Hospital, Boston, MA 02114, United States; Department of Psychiatry, Center for OCD, Anxiety, and Related Disorders, McKnight Brain Institute, College of Medicine, University of Florida, Gainesville, FL 32603, United States; Center for Neurobehavioral Genetics, Semel Institute for Neuroscience and Human Behavior, University of California, Los Angeles, CA 90024, United States; The Collaboratory, Institute for Quantitative and Computational Biosciences, University of California, Los Angeles, CA 90095, United States; Division of Genetic Medicine and Clinical Pharmacology, Department of Medicine, Vanderbilt University Medical Center, Nashville, TN 37240, United States; Psychiatric and Neurodevelopmental Genetics Unit, Departments of Psychiatry and Neurology, Center for Genomic Medicine, Massachusetts General Hospital, Boston, MA 02114, United States; Stanley Center for Psychiatric Research, Broad Institute of MIT and Harvard, Cambridge, MA 02142, United States; Psychiatric and Neurodevelopmental Genetics Unit, Departments of Psychiatry and Neurology, Center for Genomic Medicine, Massachusetts General Hospital, Boston, MA 02114, United States; Division of Genetic Medicine and Clinical Pharmacology, Department of Medicine, Vanderbilt University Medical Center, Nashville, TN 37240, United States; Department of Psychiatry, Center for OCD, Anxiety, and Related Disorders, McKnight Brain Institute, College of Medicine, University of Florida, Gainesville, FL 32603, United States; Department of Psychiatry, Center for OCD, Anxiety, and Related Disorders, McKnight Brain Institute, College of Medicine, University of Florida, Gainesville, FL 32603, United States; Center for Neurobehavioral Genetics, Semel Institute for Neuroscience and Human Behavior, University of California, Los Angeles, CA 90024, United States; Department of Human Genetics, David Geffen School of Medicine, University of California, Los Angeles, CA 90095, United States; Stanley Center for Psychiatric Research, Broad Institute of MIT and Harvard, Cambridge, MA 02142, United States; Psychiatric and Neurodevelopmental Genetics Unit, Departments of Psychiatry and Neurology, Center for Genomic Medicine, Massachusetts General Hospital, Boston, MA 02114, United States; Division of Movement Disorders, Department of Neurology, Massachusetts General Hospital, Boston, MA 02114, United States; Division of Cognitive and Behavioral Neurology, Brigham and Women’s Hospital, Boston, MA 02115, United States; Department of Psychiatry, Center for OCD, Anxiety, and Related Disorders, McKnight Brain Institute, College of Medicine, University of Florida, Gainesville, FL 32603, United States; University of Florida Genetics Institute, Genetics and Genomics Graduate Program, College of Medicine, University of Florida, Gainesville, FL 32610, United States; Evelyn F. and William L. McKnight Brain Institute, College of Medicine, University of Florida, Gainesville, FL 32603, United States

## Abstract

**Motivation:**

Copy-number variants (CNVs) are a form of genetic structural variation with increasing importance in complex human disorders. Both DNA sequencing and microarray data can be used to detect CNVs, which can be used in genetic association tests. Unlike genotypes, CNV detection in microarrays requires the use of observed intensity signals at each probe, which limits the imputability for analyses that span multiple array types. Thus far, a consensus set of probes (those present on all arrays) has been used to circumvent the problem of differing array-specific sensitivities. This has led to excessive reduction in overall sensitivity since arrays can have an undesirably low probe overlap. To overcome this limitation, we developed MarkerMatch, a proximity-based algorithm that matches probes across different genotyping microarrays to maximize the number of probes considered in the CNV calling algorithm, thereby increasing the resolution and sensitivity while preserving precision.

**Results:**

By analyzing CNV calls from 4906 individuals genotyped across three different arrays, we show that the MarkerMatch approach improves sensitivity by increasing the density of probes available for CNV calling while maintaining precision or improving it relative to the current practice (e.g. use of consensus probes only). We further demonstrate that MarkerMatch matches the CNV detection from current practice in terms of F1 score and PPV for larger CNVs. We also optimize MarkerMatch parameters, D_MAX_ and Method, and find an optimal D_MAX_ setting at 10 kb, with no clear optimal candidate based on Method, indicating that parameters for this metric should be determined on a use case basis.

**Availability:**

The R package for MarkerMatch is available at: https://github.com/FranjoIM/MarkerMatch. The code used for analysis and implementation is available at: https://doi.org/10.5281/zenodo.18460979. The live notebook is available at https://fivankovic.notion.site/2026-markermatch.

## 1 Introduction

Copy-number variants (CNVs) are a form of structural variant involving unbalanced rearrangements leading to increased (duplication) or decreased (deletion) DNA content (Zarrei *et al.* 2015). CNVs have been studied in the context of various complex human disorders to better understand their underlying pathobiology (Lionel *et al.* 2011, Olson *et al.* 2014, Huang *et al.* 2017, Marshall *et al.* 2017, Wang S *et al.* 2018, Nakatochi *et al*. 2021, Fu *et al.* 2022). Microarrays are one of the most commonly used technologies to assay CNVs due to their relatively low cost and widespread use in genome-wide association studies and biobanks such as All of Us and the UK Biobank (Bycroft *et al.* 2018, Bick *et al.* 2024).

While microarrays were not designed for the specific purpose of assaying CNVs, several algorithms have been developed to accurately assess CNV events using microarray data. Some software, e.g. PennCNV, QuantiSNP, Birdseye, and GenoCN, employ Bayesian approaches, such as Hidden Markov Models (HMM), to call CNVs using the intensity data from genotyping microarrays (Colella *et al.* 2007, Wang K *et al.* 2007, McCarroll *et al.* 2008, Sun *et al.* 2009). Other approaches like cnvPartition or iPattern employ recursive partitioning and/or clustering to determine copy-number states (Pinto *et al.* 2011, Illumina 2017). More recent methods have focused on combining or building on existing approaches to fine-tune CNV calling performance (Zhang *et al.* 2019, Lavrichenko *et al.* 2021).

Genotyping microarrays vary in the density and selection of probes, with some arrays designed to capture variation specific to populations, diseases, or genomic regions (Ehli *et al.* 2017). This variability presents challenges to meta-analysis efforts, which are crucial for aggregating sufficiently powered datasets to detect genetic associations in complex traits (Zeggini and Ioannidis 2009).

By leveraging the effects of linkage disequilibrium, accurate imputation of non-genotyped probes it is possible to enable joint and meta-analysis across different genotyping arrays (Scheet and Stephens 2006, Marchini and Howie 2010). However, CNV detection algorithms rely on direct probe intensity measures that may vary across arrays, and which cannot be imputed for CNV analysis.

Probe density and distribution vary across different array products, which may lead to array-type biases in CNV call sensitivity and specificity (Wang K *et al.* 2007). Traditionally, to avoid such biases, researchers have taken manifest intersections and focused only on probes that were genotyped on all SNP arrays for CNV detection and analyses (Huang *et al.* 2017). However, this approach works only if the genotyping microarrays considered are highly similar; if the overlap between probes across the genotyping microarrays is low, then the overall resolution and sensitivity of CNV detection and analyses can be severely impacted.

To overcome this limitation, we developed MarkerMatch. MarkerMatch is a proximity-based algorithm that matches probes across different genotyping microarrays within a specified genomic distance (a distance between a probe on reference array and those on the matched array) to maximize the number of probes considered in the CNV calling algorithm, thereby increasing the resolution and sensitivity of subsequent genome-wide CNV association analyses without affecting their specificity. MarkerMatch returns a list of probes for each of the matched arrays to be used in CNV calling. We tested MarkerMatch in two independent experiments: a within-array experiment to test the effects of probe selection on CNV calling, and a cross-array experiment to test the reliability of CNV calling in samples genotyped on multiple arrays.

## 2 Materials and methods

### 2.1 Samples and software

Samples from two independent cohorts (totaling 4906 individuals) and three different Illumina array products (Global Screening Array, GSA1; Omni2.5 array, OMNI; and Omni Express Exome array, OEE) were used to test and validate MarkerMatch algorithms. A summary of the cohorts, specific genotyping platforms, sample sizes, and probe density information is shown in Table 1.

**Table 1:** Samples used in this study.

Collection	Chip	*N*	Probe density
SSC	OMNI	4239	2 376 441
TAAICG	OEE	667	931 967
	GSA1	667	600 679

SSC, Simons Simplex Collection; TAAICG, Tourette Association of America International Consortium for Genetics; OMNI, Illumina Infinium Omni2.5; OEE, Illumina Infinium OmniExpressExome; GSA1, Illumina Infinium Global Screening Array (v1). Probe density denotes number of probes for autosomal (chr1-22) regions only.

Briefly, we used existing genomic data from the Simons Simplex Collection (SSC) and Tourette Association of America International Consortium for Genetics (TAAICG), detailed descriptions of which are provided in the Supplementary Information (Fischbach and Lord 2010, Scharf *et al.* 2013, Sanders *et al.* 2015, Huang *et al.* 2017). Table 2 summarizes software used in this study.

**Table 2:** Software used in this study.

Software	Version	References
betareg	3.2–1	Cribari-Neto and Zeileis (2010)
GenomeStudio	2.0.5	Illumina (2020)
ggpubr	0.6.0	Kassambara (2023)
PennCNV	1.0.5	Wang K *et al.* (2007, 2008) and Diskin *et al.* (2008)
parameters	0.28.3	Lüdecke *et al.* (2019)
plink	1.90 beta 7.4	Purcell *et al.* (2007)
R	4.5.2	R Core Team (2021)
R Studio	2024.12.1	Posit Team (2024)
tidyverse	1.3.2	Wickham *et al.* (2019)

### 2.2 Development of MarkerMatch

**Figure 1:**
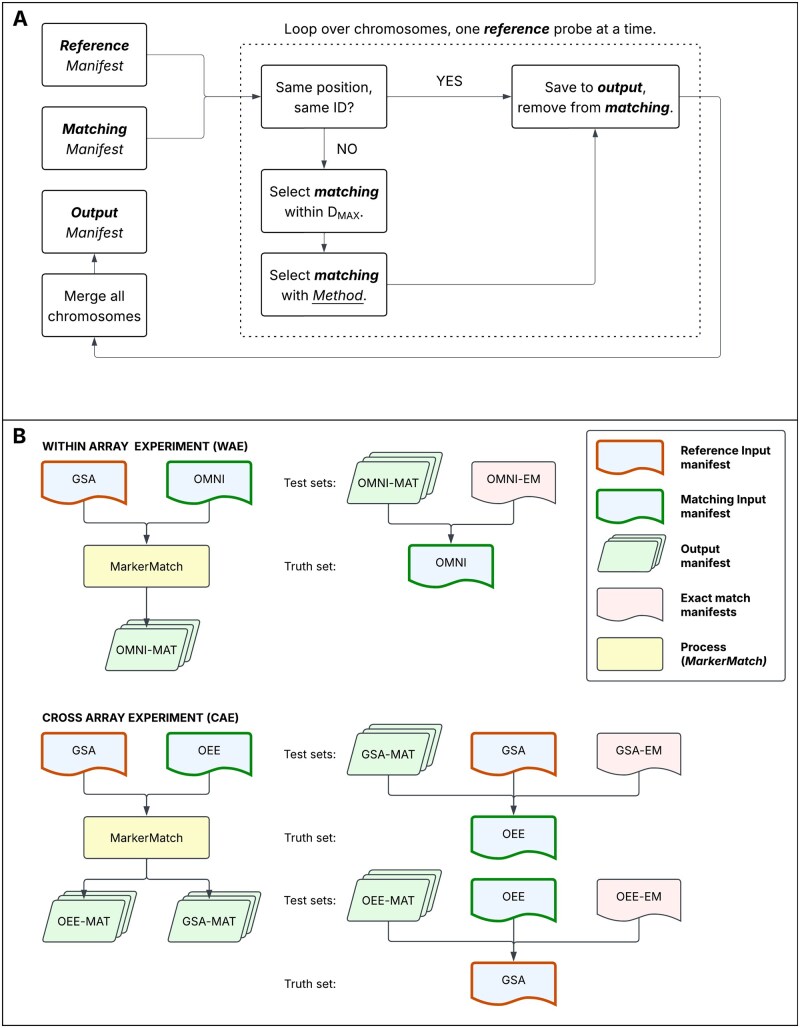
MarkerMatch and validation study diagrams. (A) Diagram depicting the MarkerMatch algorithm. MarkerMatch follows a step-by-step process to identify the best match for each probe across the two selected manifests, while ensuring no duplicates. Specifically, in the first step (exact matching) MarkerMatch will take an intersection of probes from two manifests and keep them in the output. The second step (Method matching): MarkerMatch will take a probe from the reference manifest and match it with all the remaining probes (those not used in the first step) in the matching manifest within the specified D_MAX_ distance. A probe from the matched manifest that has the smallest difference in the selected Method from the selected probe from the reference manifest will be retained. Once a probe from the matching manifest is paired with a reference probe, it is removed from the matching manifest. This prevents it from being matched again, avoiding repetitive matching of identical probes from the matched manifest. This process will continue until all reference probes have been considered. (B) Graphical representation of experimental setup. Blue boxes represent unprocessed array data, with red borders representing reference manifests and green borders representing matching manifests. Yellow boxes represent the MarkerMatch algorithm for WAE (for all Methods and 10 bp < D_MAX_ < 5 Mb) and CAE (for all Methods and D_MAX_ = 10 kb). Green boxes represent output manifests of the MarkerMatch algorithm (-MAT suffix indicates output manifests from MarkerMatch). Red boxes represent exact match manifests as currently used in CNV association analyses (intersections, also consensus manifests and -EM suffix). In WAE, we compared matched OMNI callsets to full OMNI as a truth set (also Full Set). In CAE, we compared matched GSA1 callsets to full OEE as a truth set, as well as matched OEE callsets to full GSA1 as a truth set. OMNI: Omni2.5 array, GSA1: Global Screening Array v1, OEE: Omni Express Exome array. These processes have been repeated for each iteration of Method and D_MAX_ combination.

MarkerMatch follows a simple loop and match algorithm (Fig. 1A) to identify the best-matching probes between two manifests at a time. MarkerMatch takes in two annotated manifests, the smaller of which is considered the reference manifest and the larger the matching manifest. Both reference and matching manifests must contain the following information: (i) probe name, (ii) chromosome, (iii) genomic position, (iv) B-allele frequency (BAF), (v) mean of the log-R ratio (LRR mean), and (vi) standard deviation of LRR (LRR sd). In addition to the two manifests, the MarkerMatch function requires two pre-specified parameters: D_MAX_ (which determines the maximum allowable distance from which a probe can be selected) and Method (which determines what metric should be prioritized for matching), and returns a 1:1 set of matched probes from the two manifests. There are 4 options for the Method parameter in the MarkerMatch function that can be used to select probes: Distance, BAF, LRR mean, and LRR sd.

MarkerMatch follows a step-by-step process to identify the best match for each probe across the two selected manifests, while ensuring no duplicates. Specifically, in the first step (exact matching) MarkerMatch will take an intersection of probes from two manifests and keep them. In the second step (nearby matching), MarkerMatch will take probes from the reference manifest and match them with all the remaining probes (those not used in the first step) in the matching manifest within the specified D_MAX_ distance. A probe from this set that has the smallest difference in the selected Method from the identified probe in the reference manifest will be selected and saved into the output manifest. Once a probe from the matching manifest is paired with a reference probe, it is removed from the matching manifest to avoid it being matched again. This process will continue until all reference probes are considered.

MarkerMatch was written as an R package dependent only on tidyverse packages (Wickham *et al.* 2019) and is easy and flexible to implement.

### 2.3 CNV calling

The Illumina GenomeStudio final reports were exported from for each array and passed into PennCNV to call CNVs (Wang K *et al.* 2007, Illumina 2020: 202). Data preprocessing, array clustering, genotyping quality control, CNV calling and quality control are described in detail in the Supplementary Information.

For the Within-Array Experiment (WAE) utilizing SSC data, we performed CNV calling for the GSA1-matched OMNI manifest at variable MarkerMatch matching metrics (Distance, LRR mean, LRR standard deviation, and BAF), and variable maximum allowable distances (10 bp, 50 bp, 100 bp, 500 bp, 1 kb, 5 kb, 10 kb, 50 kb, 100 kb, 500 kb, 1 Mb, and 5 Mb), as well as for the full OMNI manifest (full set) and for the intersection of the OMNI manifest with the GSA1 manifest (exact match). We additionally modeled validation metric performance of MarkerMatch callsets relative to the Full Set to determine the optimal D_MAX_ parameter setting.

For the Cross-Array Experiment (CAE) utilizing TAAICG data, we performed CNV calling for the full OEE manifest (full OEE set), the full GSA1 manifest (full GSA1 set), an intersection of the OEE manifest with the GSA1 manifest (exact match), as well as the GSA1-matched OEE manifest at a fixed maximum allowable distance of 10 kb and variable MarkerMatch matching metrics (Distance, LRR mean, LRR standard deviation, and BAF).

### 2.4 Validation

We performed two independent validations of MarkerMatch: the Within-Array Experiment (WAE) examined MarkerMatch performance in probe reductions within the same array (OMNI data from SSC), and the Cross-Array Experiment (CAE), which examined MarkerMatch performance across arrays using the OEE and GSA1 data from TAAICG. A detailed explanation of these two experiments is provided in the Supplementary Information, and the graphical representation is shown in Fig. 1B.

For each experiment, we derived a partial confusion matrix including true positive, false positive, and false negative counts. Truth sets were full set OMNI, OEE, and GSA1 CNV callsets. True negative counts were impossible to determine as we do not know the true copy-number states for the examined genomes. Based on the partial confusion matrix, we derived the following metrics: true positive rate (sensitivity, recall), false negative rate (FNR), positive predictive value (PPV, precision), false discovery rate (FDR), F1 score (F1; harmonic mean of precision and recall), Fowlkes–Mallows index (FMI; geometric mean of precision and recall); and Jaccard index (JI; ratio of the intersection to the union of the two sets). These data were used to assess performance of specific D_MAX_ and Method parameter configurations in MarkerMatch callsets, and to inform decisions for optimal parameter selection. The full methodology is available in Supplementary Information.

## 3 Results

### 3.1 Implementation of MarkerMatch

**Figure 2:**
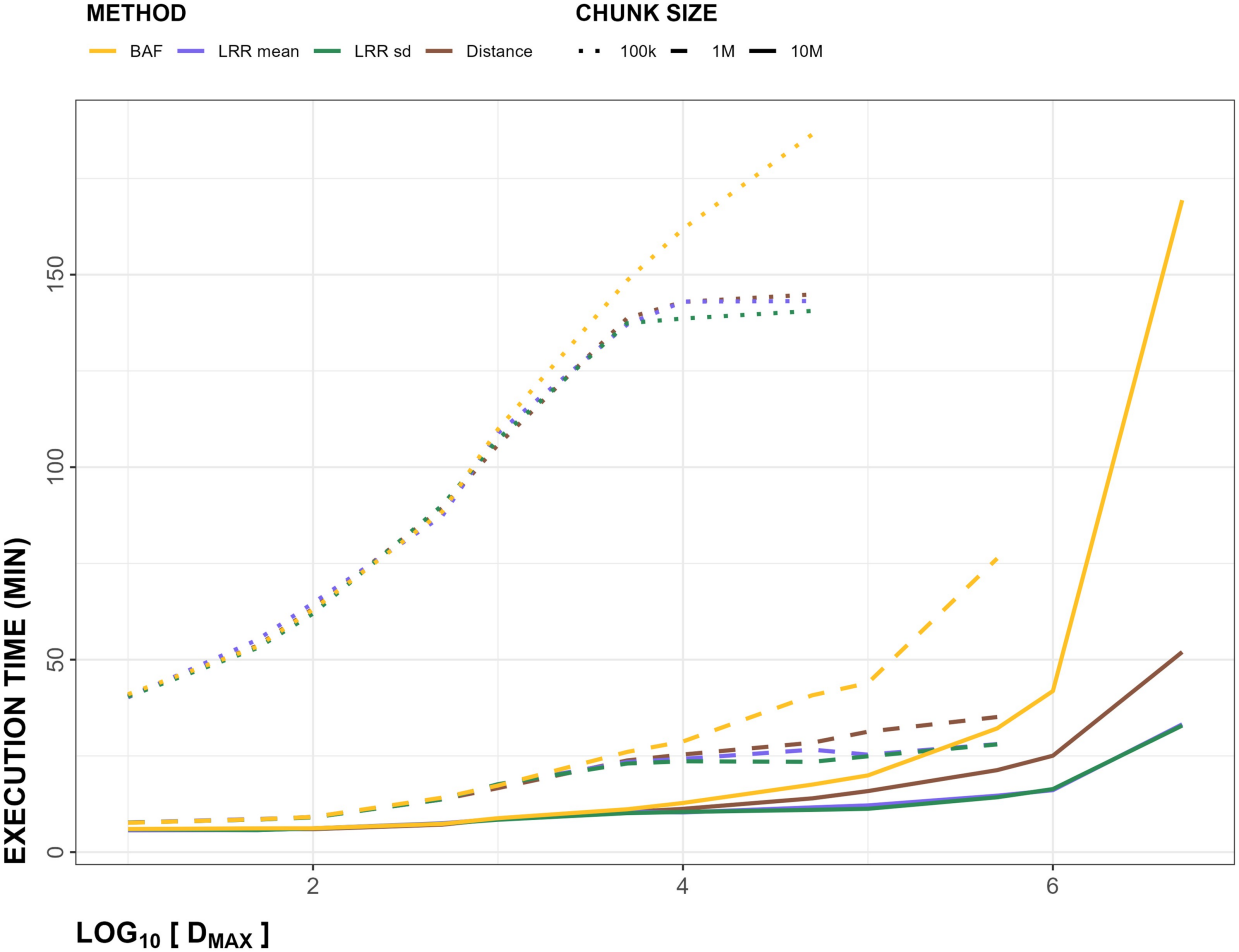
MarkerMatch execution times. Execution time curves for whole genomes, shown as function of time (in minutes) on the y-axis, and log_10_ of maximum matching distance *D_MAX_* (in bp) on x-axis. Line type shows chunk size (100 000 in dotted, 1 000 000 in dashed, and 10 000 000 in solid). *Method* is shown in colors (BAF in yellow, LRR mean in purple, LRR sd in green, and Distance in brown).

The total genome-wide runtime for MarkerMatch is dependent on chosen D_MAX_ and Chunk parameters (Fig. 2, Supplementary Table S1A), with average running time across all Methods for D_MAX_ parameter of 10 kb and Chunk parameters of 100 kn, 1 Mb, and 10 Mb being 2.4 hours, 25.5 minutes, and 11.2 minutes, respectively.

**Figure 3:**
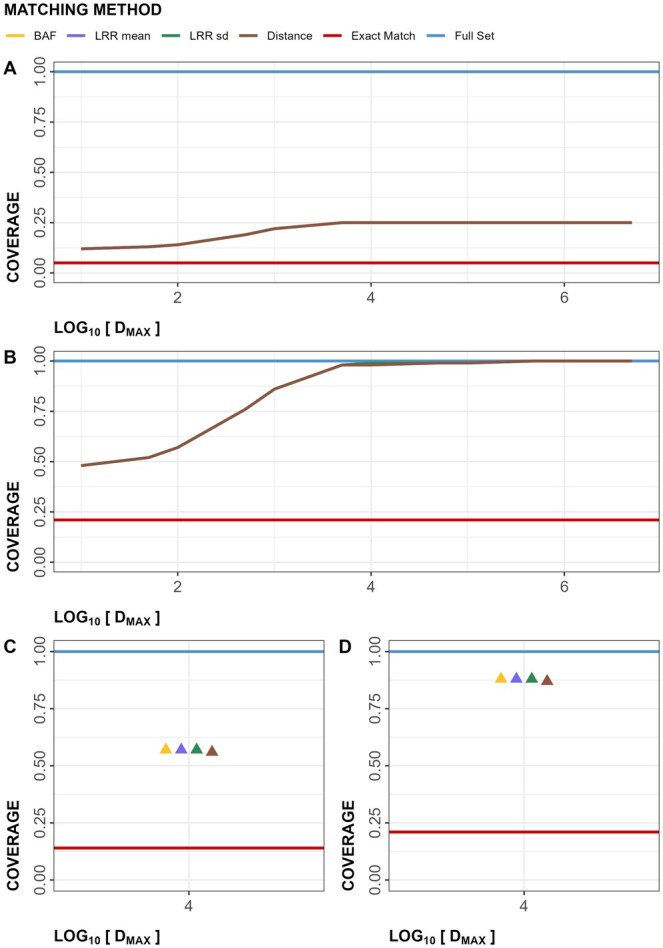
Array coverage. Figure showing coverage of arrays in Within-Array Experiment (WAE; A: OMNI, B: GSA1) and Cross-Array Experiment (CAE; C: OEE, D: GSA1). In all graphs, y-axis is showing the coverage rates, and the x-axis is showing maximum allowable distances in base-pairs, log_10_ (D_MAX_). Note: Lines for all matching Methods in panels A and B are overlapped. Points on panels C and D are horizontally jittered for visibility, but log_10_(D_MAX_) is 4 for all matching Methods. OMNI: Omni2.5 array, GSA1: Global Screening Array v1, OEE: Omni Express Exome array.

Analysis of array coverage shows successful recovery of GSA1 coverage when matched with both the OMNI and OEE arrays (Fig. 3A–D, Table 3, Supplementary Table S1B). For the OMNI array, coverage plateaued at a D_MAX_ value of 10 kb, with 25% coverage of the OMNI array (Fig. 3A) and 98% coverage of the GSA1 array (Fig. 3B). In contrast, the exact match approach resulted in retention of 5% of probes from the OMNI array (Fig. 3A) and 21% of probes from the GSA1 array (Fig. 3B).

**Table 3:** Summaries of MarkerMatch outcomes at D_MAX_ = 10 kb across all Method parameters (BAF, LRR mean, LRR sd, and Distance), as well as Full Set and Exact Match reference comparisons.

Measure	Full Set	BAF	LRR mean	LRR sd	Distance	Exact Match
OMNI array, OMNI matched to GSA1						
Coverage	1.00	0.25	0.25	0.25	0.25	0.05
Gaps	2.79 (0.79)	3.41 (0.79)	3.41 (0.79)	3.41 (0.79)	3.41 (0.79)	4.05 (0.74)
BAF	0.07 (0.25)	0.05 (0.21)	0.09 (0.24)	0.12 (0.27)	0.11 (0.26)	0.26 (0.21)
LRR sd	0.10 (0.04)	0.10 (0.04)	0.10 (0.05)	0.10 (0.05)	0.10 (0.04)	0.10 (0.04)
LRR mean	−0.002 (0.004)	−0.002 (0.003)	−0.002 (0.004)	−0.002 (0.004)	−0.002 (0.004)	−0.001 (0.004)
GSA1 array, OMNI matched to GSA1						
Coverage	1.00	0.99	0.99	0.99	0.98	0.21
OEE array, OEE matched to GSA1						
Coverage	1.00	0.57	0.57	0.57	0.56	0.14
Gaps	3.11 (1.15)	3.44 (0.87)	3.44 (0.86)	3.44 (0.86)	3.46 (0.83)	4.04 (0.74)
BAF	0.14 (0.30)	0.15 (0.27)	0.18 (0.28)	0.18 (0.28)	0.19 (0.28)	0.25 (0.22)
LRR sd	0.13 (0.06)	0.13 (0.06)	0.13 (0.06)	0.12 (0.06)	0.13 (0.06)	0.13 (0.05)
LRR mean	−0.003 (0.009)	−0.003 (0.009)	−0.003 (0.009)	−0.003 (0.008)	−0.003 (0.009)	−0.003 (0.009)
GSS1 array, OEE matched to GSA1						
Coverage	1.00	0.88	0.88	0.88	0.87	0.21
Gaps	3.40 (0.83)	3.45 (0.81)	3.45 (0.81)	3.45 (0.81)	3.56 (0.81)	4.04 (0.74)
BAF	0.04 (0.20)	0.05 (0.22)	0.05 (0.21)	0.05 (0.21)	0.05 (0.21)	0.25 (0.22)
LRR sd	0.11 (0.06)	0.11 (0.06)	0.11 (0.06)	0.11 (0.06)	0.11 (0.06)	0.11 (0.05)
LRR mean	−0.003 (0.006)	−0.003 (0.006)	−0.003 (0.006)	−0.003 (0.006)	−0.003 (0.006)	−0.002 (0.006)

Coverage is displayed in rate; gaps and LRR sds are displayed in log_10_[median] (log_10_[IQR]); BAFs and LRR means are displayed in median (IQR). Within-array experiment outcomes are detailed in OMNI array, OMNI matched to GSA1 and GSA1 array, OMNI matched to GSA1 segments. Cross-array experiment outcomes are detailed in OEE array, OEE matched to GSA1 and GSA1 array, OEE matched to GSA1 segments.

For the OEE array, coverage at D_MAX_ = 10 kb was 57% of the OEE array (Fig. 3C) and 88% of the GSA1 array (Fig. 3D). The exact match approach resulted in the retention of 14% probes from the OEE array (Fig. 3C) and 21% probes from the GSA1 array (Fig. 3D).

Inter-probe gaps (i.e. gaps between SNPs used by PennCNV to make CNV calls) were also reduced in size on both the OMNI (median 2.6 kb at D_MAX_ = 10 kb) and OEE (median 2.8 kb at D_MAX_ = 10 kb) arrays matched to the GSA1 array, relative to their Exact Match counterparts with a median of about 11 kb (Supplementary Fig. S1A–C, Table 3, Supplementary Table S1C).

BAF distributions of Full Set arrays were better approximated by MarkerMatch than Exact Match configurations (Supplementary Fig. S2A–C, Table 3, Supplementary Table S1D). Notably, the median BAF differed for each Method, with the median BAF being 0.05 for Method = BAF, 0.09 for Method = LRR mean, 0.12 for Method = LRR sd, and 0.11 for Method = Distance arrays, whereas the median BAF values for Exact Match and Full Set were 0.26 and 0.07, respectively.

Conversely, LRR sds and LRR means showed relatively little variability between various Method and D_MAX_ configurations of MarkerMatch, as well as between MarkerMatch configurations and Full Set and Exact Match, with median LRR sd values around 0.12 and LRR mean values around −0.002 (Supplementary Figs. S3A–C, S5A–C, S6A–C, Table 3, Supplementary Tables S1E, S1F).

### 3.2 Within-Array Experiment (WAE)

Within-Array Experiment (WAE) results are summarized in Table 4, and in Supplementary Tables S1G–I. Briefly, the Full Set callset resulted in the most CNV calls (low-stringency QC = 280 041; medium-stringency QC = 47 307) and Exact Match resulted in the fewest (low-stringency QC = 10 917; medium-stringency QC = 1522). MarkerMatch callsets counted 3–8 times more CNV calls relative to Exact Match across all four Methods at D_MAX_ = 10 kb (low-stringency QC range 53 862—57 105; medium-stringency QC range 12 724—14 060).

**Table 4:** Summaries of MarkerMatch CNV callsets at D_MAX_ = 10 kb across all Method parameters (BAF, LRR mean, LRR sd, and Distance), as well as Full Set and Exact Match reference comparisons for OMNI array.

Type	QC	Full Set	BAF	LRR mean	LRR sd	Distance	Exact Match
CNV number, total count							
All	Low	280,041	53,862	54,939	57,105	56,132	10,917
	Medium	47,307	12,724	13,150	13,907	14,060	1,522
Deletions	Low	209,926	34,312	34,161	34,551	32,937	4,932
	Medium	27,245	5,882	5,992	6,305	6,413	427
Duplications	Low	70,115	19,550	20,778	22,554	23,195	5,985
	Medium	20,062	6,842	7,158	7,602	7,647	1,095
CNV number, per sample mean							
All	Low	66.06	12.71	12.96	13.47	13.24	2.58
	Medium	11.16	3.00	3.10	3.28	3.32	0.36
Deletions	Low	49.52	8.09	8.06	8.15	7.77	1.16
	Medium	6.43	1.39	1.41	1.49	1.51	0.10
Duplications	Low	16.54	4.61	4.90	5.32	5.47	1.41
	Medium	4.73	1.61	1.69	1.79	1.80	0.26
CNV size (bp), mean							
All	Low	34,937.28	66,985.92	67,811.99	69,266.55	69,624.58	121,392.18
	Medium	109,786.43	172,497.81	173,323.25	173,164.33	176,006.76	405,581.26
Deletions	Low	20,910.50	43,930.18	45,032.45	46,366.99	49,187.02	97,980.55
	Medium	84,401.60	141,689.55	144,569.35	144,082.17	147,634.49	466,522.89
Duplications	Low	76,933.81	107,450.82	105,263.70	104,346.94	98,646.00	140,684.78
	Medium	144,260.06	198,983.38	197.393.30	197,284.70	199,800.59	381,816.81
CNV confidence score, mean							
All	Low	47.38	35.26	37.49	37.14	37.21	31.19
	Medium	133.71	80.34	86.49	86.10	83.81	93.34
Deletions	Low	44.05	33.54	34.83	34.08	34.68	29.15
	Medium	137.33	88.56	90.99	87.64	86.12	112.08
Duplications	Low	57.33	38.29	41.86	41.83	40.80	32.87
	Medium	128.80	73.28	82.72	84.83	81.88	86.03
Sample number, total count (with at least 1 CNV call)							
All	Low	4,239	4,239	4,239	4,239	4,239	4,239
	Medium	3,827	3,925	3,927	3,956	3,941	3,508
Sample LRR means, mean							
All	Low	−0.004	−0.004	−0.004	−0.004	−0.004	−0.004
	Medium	−0.004	−0.004	−0.004	−0.004	−0.004	−0.003
Sample LRR sds, mean							
All	Low	0.12	0.12	0.12	0.12	0.12	0.12
	Medium	0.11	0.11	0.12	0.12	0.11	0.11
Sample BAF means, mean							
All	Low	0.50	0.50	0.50	0.50	0.50	0.50
	Medium	0.50	0.50	0.50	0.50	0.50	0.50
Sample BAF sds, mean							
All	Low	0.04	0.04	0.04	0.04	0.04	0.04
	Medium	0.04	0.04	0.04	0.04	0.04	0.03
Sample BAF drifts, mean							
All	Low	0	0	0	0	0	0
	Medium	0	0	0	0	0	0
Sample GCWF, mean							
All	Low	0	0	0	0	0	0
	Medium	0	0	0	0	0	0
Callset PPV							
All	Low	Reference	0.90	0.90	0.89	0.90	0.82
	Medium	1.00	0.99	0.99	0.99	0.99	1.00
Deletions	Low	1.00	0.93	0.92	0.91	0.93	0.81
	Medium	1.00	1.00	1.00	0.99	0.99	1.00
Duplications	Low	1.00	0.85	0.87	0.86	0.85	0.84
	Medium	1.00	0.99	0.99	0.99	0.98	1.00

Full tables including other D_MAX_ parameter values and specific size bins, as well as other statistics like medians and IQRs, are available in the Supplementary Tables S1G–I. Note: callset PPVs are based on comparisons to OMNI array Full Set callset. In the QC column, low stands for low-stringency QC and medium stands for medium-stringency QC.

WAE per-sample CNV calls, summarized in Table 4 and Supplementary Table S1G, were highest for the Full Set callset (low-stringency QC = 66.06; medium-stringency QC = 11.16), lowest for Exact Match (low-stringency QC = 2.58, medium-stringency QC = 0.36), and about 5–9 times the Exact Match in MarkerMatch callsets across all four Methods at D_MAX_ = 10 kb (low-stringency QC range 12.71–13.47; medium-stringency QC range 3.00–3.32).

The average CNV sizes (in bp) identified in WAE were smaller on denser MarkerMatch configurations (Table 4, Supplementary Table S1G). Full Set averages were the smallest (low-stringency QC 34 937.28 bp; medium-stringency QC 109 786.43 bp), Exact Match were the largest (low-stringency QC 121 392.18 bp; medium-stringency QC 405 581.26 bp), and the MarkerMatch callsets were intermediate (low-stringency QC range 66 985.92 bp—69 624.58 bp; medium-stringency QC 172 497.81 bp—176 006.76 bp).

Average confidence scores in WAE were larger on denser configurations (Table 4, Supplementary Table S1G), ranging from 133.71 on medium-stringency QC Full Set to 31.19 on low-stringency QC Exact Match. As with other metrics, CNV confidence scores were intermediate for MarkerMatch callsets (low-stringency QC range 35.26–37.49; medium-stringency QC range 80.34–86.49).

The overall number of samples with at least one CNV call were consistent after low-stringency QC across all approaches (*N* = 4239), although they did vary somewhat after medium-stringency QC (*N* range 5508–3956) as shown in Table 4 and Supplementary Table S1H.

Sample-specific metrics did not vary substantially across low- or medium-stringency QC, or Full Set/Exact Match/MarkerMatch configurations, with average LRR means at −0.004, LRR sds at 0.11–0.12, BAF means at 0.50, BAF sds at 0, BAF drifts at 0, and GCWFs at 0 (Table 4, Supplementary Table S1H).

#### 3.2.1 Genome-wide validation

**Figure 4:**
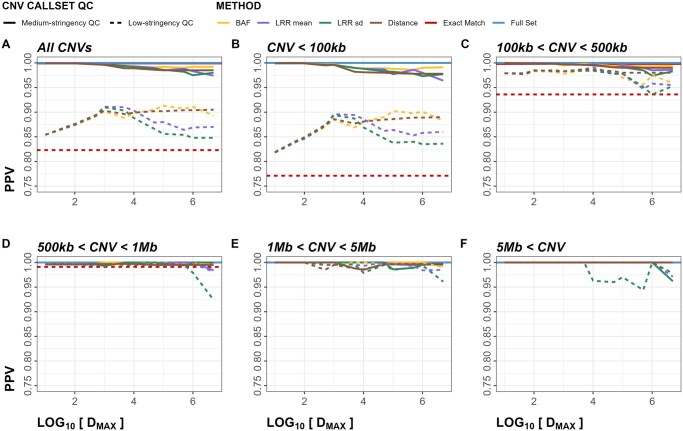
Within-Array Experiment (WAE) positive predictive value (PPV) plots for both deletions and duplications. Panels (A–F) show PPV metrics stratified by CNV size (all, under 100 kb, between 100 kb and 500 kb, between 500 kb and 1 Mb, between 1 Mb and 5 Mb, and over 5 Mb, respectively). Dashed line represents low-stringency QC, solid line represents medium-stringency QC. Line colors represent different MarkerMatching methods (B-allele frequency, BAF in yellow; Log-R ratio, LRR mean in purple; LRR standard deviation, sd in green; and Distance in brown). Red line shows exact match (intersection), and blue line shows the full complement of the array (truth set).

WAE genome-wide validation resulted in higher or about equal PPVs for MarkerMatch callsets matched at D_MAX_ = 10 kb for all Method parameters (ranges 0.89–0.90 and 0.99 for low- and medium-stringency QC, respectively) compared to Exact Match (0.82 and 1.00 for low- and medium-stringency QC, respectively) (Fig. 4, Table 4, Supplementary Table S1I).

Other metrics performed similarly, with larger CNVs having consistently better performance than smaller CNVs (Supplementary Figs. S7–12, Supplementary Table S1I). Additionally, deletions had a slightly better performance than duplications (Supplementary Figs. S12–25, Supplementary Table S1I). The genome-wide PPV plots (Fig. 4) indicated that, in the majority of D_MAX_ and Method parameter configurations, MarkerMatch somewhat or substantially outperformed the Exact Match approach.

#### 3.2.2 Regional validation

Performance of MarkerMatch callsets at D_MAX_ = 10 kb was consistent across various Method parameters in the medium-stringency QC callset, with average PPVs of 0.99 in segmental duplications region. There were no calls in segmental duplication regions in the exact match callset. Additionally, many of the MarkerMatch callsets, as well as Exact Match callset at medium-stringency QC lack CNV calls in RepeatMasker regions. For low-stringency QC, D_MAX_ = 10 kb, callsets, region-specific average PPVs were 0.97 (telomeric), 0.79 (centromeric), 0.94 (immunoglobulin and T-cell receptor), 0.81 (RepeatMasker), and 0.91 (segmental duplications).

Detailed reports for other D_MAX_ parameter settings and performance metrics are available in the Supplementary Table S1J. These data are graphically shown for all validation metrics in the Supplementary Figs. S26–32.

#### 3.2.3 Selection of D_MAX_

Analysis of validation metrics after loess-smoothing and Full Set scaling (see the supplement equation Eq. 8) indicates that, for the majority of metrics (across sensitivity, PPV, F1, FMI, and JI), regardless of CNV size (all, CNV < 100 kb, 100 kb < CNV < 500 kb, or 500 kb < CNV < 1 Mb), CNV type (all, deletions, or duplications), or Method parameter (BAF, LRR mean, LRR sd, or Distance), the peak, plateau, and/or inflection point occurred in the range of D_MAX_ between 10 kb and 100 kb (Supplementary Figs. S32–37). Further inspection indicated that D_MAX_ = 10 kb was the optimal maximum allowable distance to match within and was thus chosen as the D_MAX_ setting for the Cross-Array Experiment (CAE).

#### 3.2.4 Selection of method

Analysis of the plotted validation metrics after Full Set scaling (see the supplement equation Eq. 8) indicated that, for the majority of metrics (across sensitivity, PPV, F1, FMI, and JI), regardless of CNV size (all, CNV < 100 kb, 100 kb < CNV < 500 kb, or 500 kb < CNV < 1 Mb), CNV type (all, deletions, or duplications), there was no substantial difference in performance across various Method parameters (Supplementary Figs. S38–42). There were no significant differences between PPV values. No significant differences were observed between any two Method’s metrics, for any CNV type and CNV size strata after FDR correction (Supplementary Table S1K). We thus opted to examine all Method parameters in the Cross-Array Experiment (CAE).

### 3.3 Cross-Array Experiment (CAE)

Cross-Array Experiment (CAE) results are summarized in Tables 5 and 6, Supplementary Tables S1L–N. Briefly, similarly to WAE, Full Sets in both the OEE and GSA1 array resulted in the most CNV calls (low-stringency QC 14 587 calls and 19 396 calls; medium-stringency QC 2293 and 1818 calls on GSA1 and OEE, respectively) and Exact Match the fewest (low-stringency QC on GSA1 array 2348 and 1837 calls; medium-stringency QC 265 and 224 calls on GSA1 and OEE, respectively). MarkerMatch callsets counted 4–7 times more CNV calls relative to Exact Match across all four Methods at D_MAX_ = 10 kb on both arrays. While the average number of low-stringency QC calls overall was not substantially different between the OEE and GSA1 arrays (GSA1 overall callsets counted up to 1.3 times more), the differences between the two arrays observed in medium-stringency QC callsets were negligent across all four Method metrics.

**Table 5:** Summaries of MarkerMatch CNV callsets at D_MAX_ = 10 kb across all Method parameters (BAF, LRR mean, LRR sd, and Distance), as well as Full Set and Exact Match reference comparisons for GSA1 array (Ref = OEE).

Type	QC	Full Set	BAF	LRR mean	LRR sd	Distance	Exact Match
CNV number, total count							
All	Low	14,587	11,122	10,528	10,504	10,548	2,348
	Medium	2,293	1,479	1,468	1,502	1,440	265
Deletions	Low	8,128	6,145	5,788	5,895	5,851	1,161
	Medium	1,125	633	616	634	617	94
Duplications	Low	6,459	4,977	4,740	4,609	4,697	1,187
	Medium	1,168	846	852	868	823	171
CNV number, per sample mean							
All	Low	22.14	16.88	15.98	15.94	16.01	3.56
	Medium	3.48	2.24	2.23	2.28	2.19	0.40
Deletions	Low	12.33	9.32	8.78	8.95	8.88	1.76
	Medium	1.71	0.96	0.93	0.96	0.94	0.14
Duplications	Low	9.80	7.55	7.19	6.99	7.13	1.80
	Medium	1.77	1.28	1.29	1.32	1.25	0.26
CNV size (bp), mean							
All	Low	73,798.56	61,311.54	65,546.20	66,219.47	65,368.46	96,579.43
	Medium	141,937.21	153,459.19	155,319.35	151,147.99	153,297.19	330,493.62
Deletions	Low	61,635.64	49,199.29	53,316.02	54,142.43	52,465.93	84,716.61
	Medium	126,697.18	142,918.91	154,486.94	140,667.14	145,616.92	326,764.23
Duplications	Low	89,104.36	76,266.30	80,480.43	81,666.23	81,440.98	108,182.42
	Medium	156,616.17	161,345.72	155,921.19	158,803.37	159,055.05	332,543.69
CNV confidence score, mean							
All	Low	25.44	24.57	25.52	25.78	24.81	24.19
	Medium	61.31	75.38	74.70	74.00	75.55	86.28
Deletions	Low	25.38	24.96	25.93	26.00	25.25	21.65
	Medium	63.33	83.72	85.37	84.44	86.00	87.15
Duplications	Low	25.51	24.08	25.02	25.49	24.26	26.68
	Medium	59.37	69.14	66.99	66.37	67.71	85.80
Sample number, total count (with at least 1 CNV call)							
All	Low	659	659	659	659	659	659
	Medium	539	538	542	543	542	594
Sample LRR means, mean							
All	Low	−0.008	−0.008	−0.008	−0.008	−0.008	−0.006
	Medium	−0.005	−0.004	−0.004	−0.004	−0.004	−0.004
Sample LRR sds, mean							
All	Low	0.14	0.14	0.14	0.14	0.14	0.13
	Medium	0.13	0.13	0.13	0.13	0.13	0.12
Sample BAF means, mean							
All	Low	0.50	0.50	0.50	0.50	0.50	0.50
	Medium	0.50	0.50	0.50	0.50	0.50	0.50
Sample BAF sds, mean							
All	Low	0.05	0.04	0.04	0.04	0.04	0.04
	Medium	0.04	0.04	0.04	0.04	0.04	0.04
Sample BAF drifts, mean							
All	Low	0.001	0.001	0.001	0.001	0.001	0
	Medium	0	0	0	0	0	0
Sample GCWF, mean							
All	Low	0.001	0	0	0	0	0.001
	Medium	0.001	0	0	0	0	0.001
Callset PPV							
All	Low	0.28	0.35	0.38	0.39	0.37	0.58
	Medium	0.71	0.86	0.85	0.85	0.86	0.98
Deletions	Low	0.32	0.40	0.44	0.44	0.42	0.55
	Medium	0.73	0.91	0.90	0.91	0.90	0.97
Duplications	Low	0.23	0.29	0.31	0.32	0.31	0.60
	Medium	0.69	0.83	0.81	0.82	0.83	0.98

Full tables including specific size bins, as well as other statistics like medians and IQRs, are available in the Supplementary Tables S1L–N. Note: callset PPVs are based on comparisons to OEE array Full Set callset. In the QC column, low stands for low-stringency QC and medium stands for medium-stringency QC.

**Table 6:** Summaries of MarkerMatch CNV callsets at D_MAX_ = 10 kb across all Method parameters (BAF, LRR mean, LRR sd, and Distance), as well as Full Set and Exact Match reference comparisons for OEE array (Ref = GSA1).

Type	QC	Full Set	BAF	LRR mean	LRR sd	Distance	Exact Match
CNV number, total count							
All	Low	19,396	8,787	8,123	8,196	8,384	1,837
	Medium	1,818	1,294	1,439	1,472	1,498	224
Deletions	Low	13,906	6,078	5,448	5,404	5,486	1,044
	Medium	931	583	640	673	679	79
Duplications	Low	5,490	2,709	2,675	2,792	2,898	793
	Medium	887	711	799	799	819	145
CNV number, per sample mean							
All	Low	29.08	13.17	12.18	12.29	12.57	2.75
	Medium	2.73	1.94	2.16	2.21	2.25	0.34
Deletions	Low	20.85	9.11	8.17	8.10	8.22	1.57
	Medium	1.40	0.87	0.96	1.01	1.02	0.12
Duplications	Low	8.23	4.06	4.01	4.19	4.34	1.19
	Medium	1.33	1.07	1.20	1.20	1.23	0.22
CNV size (bp), mean							
All	Low	142,442.52	193,053.68	117,325.03	150,784.79	167,945.38	111,219.17
	Medium	123,114.00	157,747.10	155,170.38	153,784.43	155,054.38	308,415.88
Deletions	Low	164,984.43	228,648.16	117,288.53	168,919.72	199,188.42	92,595.79
	Medium	99,150.95	143,765.17	138,267.75	135,978.59	136,990.15	331,080.61
Duplications	Low	85,344.56	113,192.77	117,399.36	115,684.10	108,801.38	135,737.21
	Medium	148,265.74	169,211.89	168,709.41	168,782.34	170,030.70	296,067.52
CNV confidence score, mean							
All	Low	33.28	31.74	34.58	34.09	33.57	27.51
	Medium	102.39	89.65	94.95	92.47	91.53	82.49
Deletions	Low	30.17	27.72	29.28	29.25	29.06	22.82
	Medium	96.65	89.79	89.81	87.63	87.26	83.98
Duplications	Low	41.15	40.76	45.39	43.48	42.12	33.67
	Medium	108.42	89.54	99.07	96.55	95.07	81.67
Sample number, total count (with at least 1 CNV call)							
All	Low	667	667	667	667	667	667
	Medium	402	549	607	608	614	535
Sample LRR means, mean							
All	Low	−0.003	−0.004	−0.003	−0.003	−0.004	−0.005
	Medium	−0.002	−0.002	−0.002	−0.002	−0.002	−0.003
Sample LRR sds, mean							
All	Low	0.12	0.12	0.12	0.11	0.12	0.13
	Medium	0.11	0.11	0.11	0.11	0.11	0.12
Sample BAF means, mean							
All	Low	0.50	0.50	0.50	0.50	0.50	0.50
	Medium	0.50	0.50	0.50	0.50	0.50	0.50
Sample BAF sds, mean							
All	Low	0.03	0.03	0.03	0.03	0.03	0.03
	Medium	0.03	0.03	0.03	0.03	0.03	0.03
Sample BAF drifts, mean							
All	Low	0	0	0	0	0	0
	Medium	0	0	0	0	0	0
Sample GCWF, mean							
All	Low	0	0	0	0	0	0
	Medium	0	0	0	0	0	0
Callset PPV							
All	Low	0.22	0.42	0.46	0.46	0.45	0.66
	Medium	0.74	0.90	0.92	0.92	0.92	1.00
Deletions	Low	0.19	0.39	0.44	0.43	0.43	0.53
	Medium	0.77	0.94	0.96	0.96	0.95	0.99
Duplications	Low	0.28	0.50	0.52	0.51	0.50	0.84
	Medium	0.71	0.86	0.89	0.89	0.89	1.00

Full tables including specific size bins, as well as other statistics like medians and IQRs, are available in the Supplementary Tables S1L–N. Note: callset PPVs are based on comparisons to GSA1 array Full Set callset. In the QC column, low stands for low-stringency QC and medium stands for medium-stringency QC.

CAE per-sample CNV calls, summarized in Tables 5 and 6, Supplementary Table S1L, were highest for the Full Set callset (low-stringency QC 22.1 and 29.1; medium stringency QC 3.4 and 2.7 for the GSA1 and OEE arrays, respectively), lowest for Exact Match (low-stringency QC 3.6 and 2.8; medium-stringency QC 0.4 and 0.2 for the GSA1 and OEE arrays, respectively), and about 2–6 times the Exact Match in MarkerMatch callsets across all Methods at D_MAX_ = 10 kb (low-stringency QC ranges of 15.9–16.9 and 12.2–13.2; medium-stringency QC ranges of 2.5–2.3 and 1.9–2.3 for GSA1 and OEE arrays, respectively).

In CAE, the average CNV sizes were smaller on the denser configurations (Tables 5 and 6, Supplementary Table S1L). The averages CNV sizes for GSA1 were smallest for MarkerMatch callsets (low-stringency QC range 61.3 kb–66.2 kb; medium-stringency QC range 151.1 kb–155.3 kb), followed by Full Set (low-stringency QC 73.8 kb; medium-stringency QC 141.9 kb). Exact Match had the largest average CNV sizes in GSA1 (low-stringency QC 96.6 kb; medium-stringency QC 330.5 kb).

The average CNV sizes for low-stringency OEE were smallest for Exact Match callset (111.2 kb), LRR mean callset (117.3 kb), followed by Full Set (142.4 kb), and then the rest of MarkerMatch callsets (150.8 kb–193.1 kb). The average CNV sizes for medium-stringency OEE followed similar trends as GSA1, with Full set being the smallest (123.1 kb), followed by MarkerMatch callsets (153.8 kb–157.7 kb), and Exact Match being the largest (308.4 kb).

Average CNV confidence scores for the GSA1 array were about the same across all low-stringency QC configurations (Tables 5 and 6, Supplementary Table S1L). This included Full Set, Exact Match, and various Method configurations of MarkerMatch callsets (range 24.2–25.8). However, in the medium-stringency QC, Exact Match had the highest CNV confidence scores (86.3), followed by MarkerMatch callsets (range 74.0–75.6), and Full Set (61.3). For the OEE array, the spread was a bit wider among the low-stringency QC callsets (range 27.5–34.6), without clear segregation among the Full Set and MarkerMatch callsets, with Exact Match still being lowest. For medium-stringency QC callsets in the OEE array, we saw the highest average CNV confidence scores for Full Set (102.4), followed by MarkerMatch (range 89.7–95.0), and Exact Match (82.5).

The overall number of samples with at least one CNV call was consistent after low-stringency QC callsets across the board in both GSA1 and OEE array (*N* = 677, Tables 5 and 6, Supplementary Table S1M). The number of samples passing the medium-stringency QC varied somewhat across the callsets (N ranges 542–594 and 402–614, for GSA1 and OEE, respectively), with the OEE array showing a much higher range after medium-stringency QC.

Sample-specific metrics did not vary substantially across low- or medium-stringency QC, or Full Set/Exact Match/MarkerMatch configurations, or GSA1/OEE arrays (Tables 5 and 6, Supplementary Table S1M).

#### 3.3.1 Genome-wide validation

**Figure 5:**
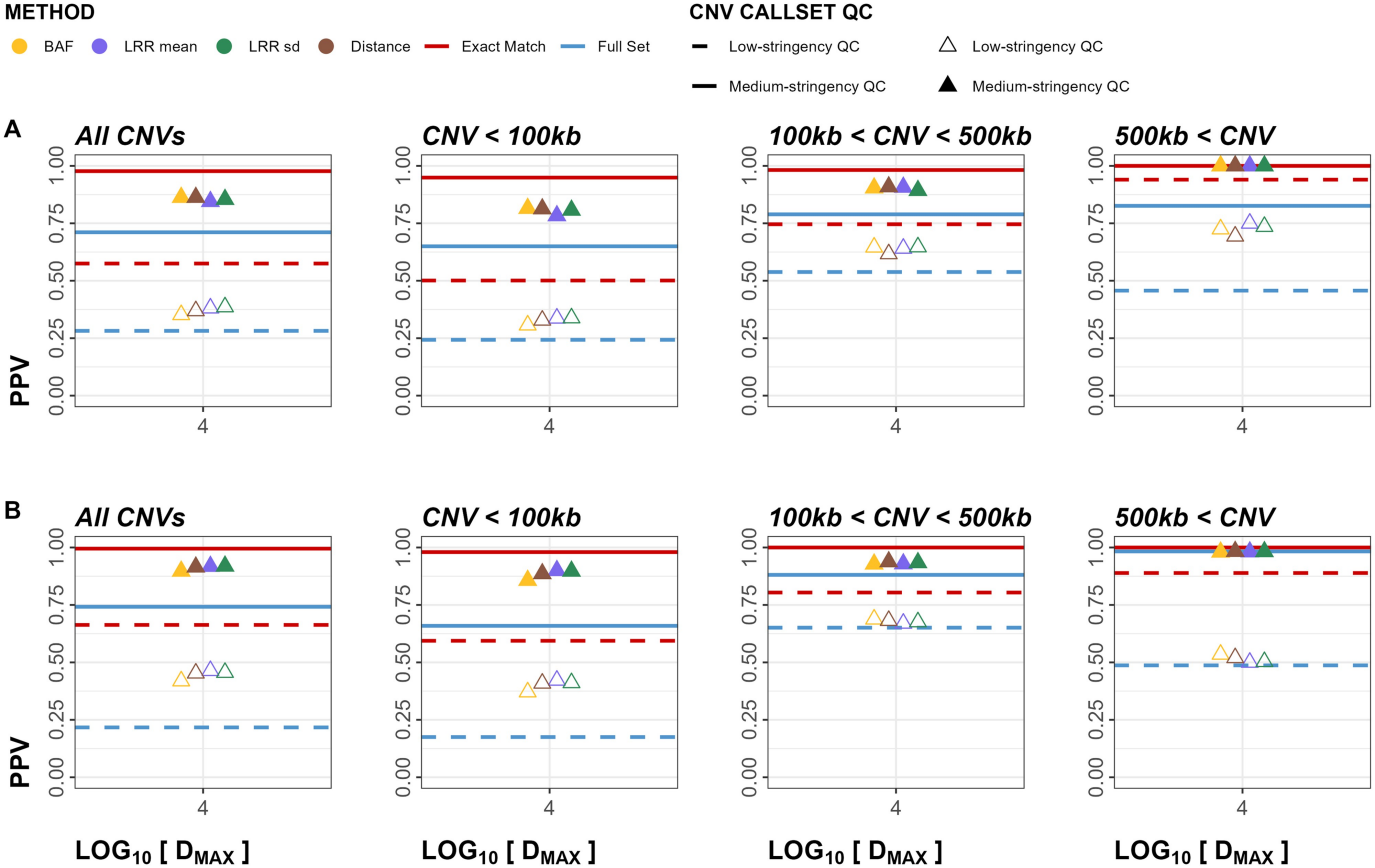
Cross-Array Experiment (CAE) positive predictive value (PPV) plots for both deletions and duplications. Panel (A) represents calls in Global Screening Array (GSA1) validated in Omni Express Exome (OEE). Panel (B) represents calls in OEE validated in GSA1. Empty triangles and dashed lines represent low-stringency QC callsets, whereas solid lines and filled triangles represent medium-stringency QC callsets. Metrics are stratified by CNV size (all, under 100 kb, between 100 kb and 500 kb, and over 500 kb, from left to right, respectively). Colors show different MarkerMatching methods (B-allele frequency, BAF in yellow; Log-R ratio, LRR mean in purple; LRR standard deviation, sd in green; and Distance in brown). Red lines show exact match (intersection), and blue lines show the full complement of the array (truth set).

CAE genome-wide validation of the GSA1 callsets (using low-stringency Full Set OEE as the truth set) resulted in PPVs that were the lowest for the Full Set (0.28), highest in Exact Match (0.58), and intermediate in the MarkerMatch (range 0.35–0.39) low-stringency QC callsets (Fig. 5A, Table 5, Supplementary Table S1N). In medium-stringency QC GSA1 callsets, the performance was significantly lower in Full Set (0.71) than in MarkerMatch callsets (range 0.85–0.86), and largest in Exact Match (0.98). CAE genome-wide validation of OEE callsets (using low-stringency Full Set GSA1 as truth set) has resulted in PPVs that were comparable to those in GSA1 callsets, however overall, slightly larger. Analysis of the genome-wide PPV plots (Fig. 5) indicates that, in most Method parameter configurations, MarkerMatch performed almost as well as the Exact Match approach, especially for larger CNVs (< 500 kb).

Other metrics performed similarly, with larger CNVs having consistently better performance than smaller CNVs (Supplementary Figs. S43–48, Supplementary Table S1N). Additionally, deletions had a noticeably better performance than duplications (Supplementary Figs. S49–62, Supplementary Table S1N).

#### 3.3.2 Regional validation

Filtering on medium-stringency QC indicated that Exact Match outperformed MarkerMatch in variable genomic regions including telomeric, centromeric, immunoglobulin, segmental duplication, and simple repeat regions (Supplementary Figs. S63–69, Supplementary Table S1O) in terms of PPV. However, when accounting for the higher sensitivity of the MarkerMatch using the F1, FMI, and JI metrics, MarkerMatch either matched or outperformed Exact Match across the board.

#### 3.3.3 Determination of optimal minimum CNV size and SNP coverage thresholds

Beta regression of PPV on the GSA1 array resulted in significant associations with CNV length cutoff, probe coverage cutoff, and their interaction terms ([OR_PPV_ = 1.10, p_PPV_ < 0.001]; [OR_PPV_ = 1.08, p_PPV_ < 0.001]; [OR_PPV_ = 1.00, p_PPV_ < 0.001] respectively). In terms of PPV, LRR sd was not significantly different. BAF ([OR_PPV_ = 1.14, p_PPV_ = 0.05]) and Distance ([OR_PPV_ = 1.25, p_PPV_ < 0.001]) seemed to overperform LRR mean. The. The model’s pseudo R^2^ was 0.25.

In OEE, similarly, the probe coverage, CNV length cutoff and the two terms’ interaction seemed to increase PPV ([OR_ppv_ = 1.06, p_PPV_ < 0.001], [OR_PPV_ = 1.03, p_PPV_ < 0.001]; [OR_PPV_ = 1.00, p_PPV_ < 0.001] respectively). BAF was the only Method parameter found to significantly differ from LRR mean, overperforming ([OR_PPV_ = 1.13, p_PPV_ = 0.007]). Distance was not significantly different from LRR mean. The model’s pseudo R^2^ was 0.37.

Graphical representations are shown in Supplementary Figs. S70–75 and model summaries in the Supplementary Information.

#### 3.3.4 Sample-wise performance

**Figure 6:**
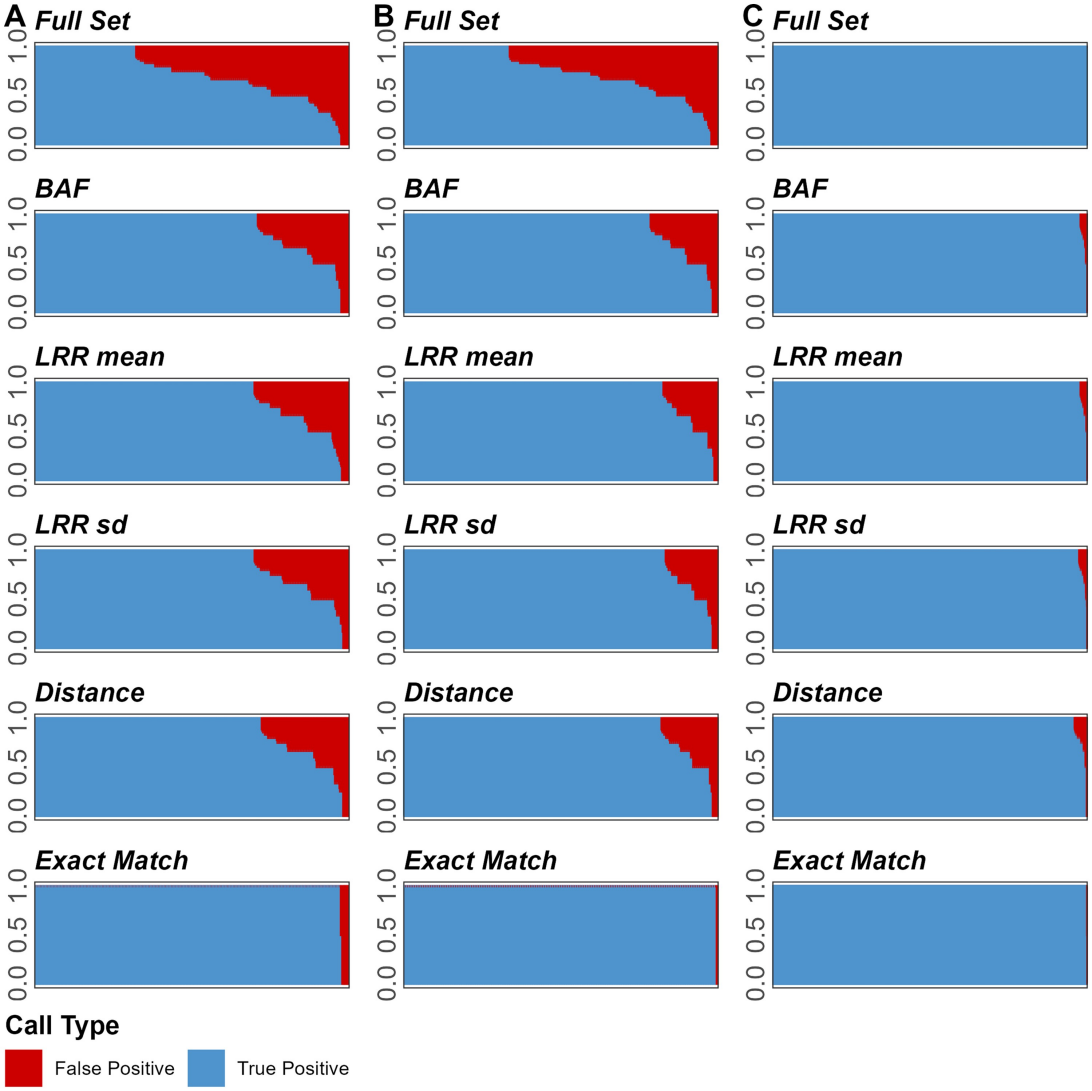
Sample-wise performance. Figure showing proportions of true positive (blue) to false positive (red) CNV calls across medium-stringency QC callsets for Cross-Array Experiment (Global Screening Array and Omni Express Exome array on columns A and B, respectively) and Within-Array Experiment (Omni2.5 array on panel C). Y-axis shows positive predictive value (PPV), x-axis shows samples (ordered in descending PPV).

The plots of performance by sample suggested that a substantial number of false positive calls were driven by either poorly performing samples or high rates of false negatives (Fig. 6). For example, considering Full Set, 359 individuals with PPV < 0.9 in the GSA1 callset (67.5%) and 260 individuals with PPV < 0.9 in the OEE callset (66.0%) accounted for over 99% of false positive calls in both arrays (Supplementary Tables S1P-Q, S2A). Because F1 scores are affected by the changes in sensitivity, and by the QC process in particular, we see an overall downward shift in distributions of F1 scores, especially for Exact Match (Supplementary Table S2B). Furthermore, a large number of individuals in Exact Match had only 1 remaining CNV call after medium-stringency QC (81.4% in OMNI, 81.7% in GSA1, and 78.0% in OEE).

Plotting the curves along this sample-wise PPV thresholding indicated that, overall, conducting this CNV sample QC step may improve callset PPV and F1 scores, with noticeable reduction of F1 scores at conservative thresholds (Supplementary Figs. S76–77). Due to excessive drop-offs of samples and reduction in sensitivity, we do not recommend filtering on sample-wise F1 scores.

## 4 Discussion

We described a new approach to CNV pre-calling quality control to increase sensitivity in cross-array CNV studies. Instead of exclusively using consensus probes across all arrays considered (an intersection of common probes, or an approach that we call Exact Match in this study), we postulated that using probes in the same genomic neighborhood of the reference probe should result in an identical CNV state call (i.e. duplication or deletion). This ability to rely on similar genomic regions as opposed to identical probes would thus result in improved sensitivity of CNV calling by allowing higher coverage of available array probes (Fig. 3).

Using simulated (within array experiment; WAE) data from the OMNI array, we show that using MarkerMatch not only substantially increased the sensitivity of CNV calling (3-fold increase in sensitivity from 0.02 in Exact Match to average sensitivity of 0.06 in MarkerMatch approach), it did so without a negative impact to PPV (1.00 in *Exact Match* compared to 0.99 in MarkerMatch) shown in Fig. 4 and Table 4. Fluctuations in apparent performance are dependent on the number of CNVs in the truth set, for example, in specific subsets of CNV callsets such as CNV size cutoff > 5 Mb only have a small number of CNV calls, therefore, a single false positive call may greatly affect observed PPV (Fig. 4F). We identified a 10 000 bp D_MAX<_ parameter as a reasonable setting, and note that none of the specific Method parameters had a clear performance edge over the others. Noticeably, however, performance metrics that consider both sensitivity and PPV, such as F1 score, Fowlkes-Mallows index, and Jaccard index, showed MarkerMatch substantially overperforming Exact Match (Supplementary Figs. S9–11).

In the cross-array experiment (CAE), we used TAAICG samples for which data were generated on two different arrays and show that both Exact Match and MarkerMatch reduced some of the batch effects associated with the use of different arrays (PPV of Full Set callset 0.71 vs. 0.85–0.98 in Exact Match and MarkerMatch callsets). CAE also demonstrated that MarkerMatch performed somewhat worse than Exact Match in terms of PPV (Fig. 5, Tables 5 and 6). Similar to the WAE, performance metrics that consider both sensitivity and PPV, such as F1, FMI, and JI, showed MarkerMatch outperforming Exact Match (Supplementary Figs. S46–48). We also determined that increases in CNV length and probe coverage cutoffs drive improvements in PPV, but may cause reduction in overall sensitivity, as expected (Supplementary Figs. S70–75). Furthermore, a possible overperformance in terms of PPV in Exact Match callsets could be due to average CNV size, which is about 2–3 times that of CNVs in Full Set and MarkerMatch callsets.

We inspected sample-wise performance rates to determine whether low PPVs were driven by individuals with low PPV (Fig. 6) and found that individual samples with sample-wise PPV < 0.9 accounted for a disproportionate number of false positive CNV calls. We further examined whether eliminating these samples would substantially improve quality of the callsets and found that while removing them leads to noticeable improvements in PPV and F1 scores, overly conservative thresholding may lead to excessive sample removal and reductions in F1 scores (Supplementary Figs. S76 and S77). Notably, excluding samples with low PPV values (e.g. PPV < 0.5) does not appear to decrease sensitivity or F1 scores.

While the MarkerMatch approach is successful in its primary function by increasing/rescuing sensitivity while also maintaining PPV and F1 scores with proper QC, MarkerMatch does not eliminate batch effects attributable to the use of different arrays. We found some evidence of the potential reduction in batch and array effects in this study, but this needs to be further explored.

Additional analyses are necessary to determine how significant array-specific batch effects really are, how much Exact Match or MarkerMatch approaches really alleviate them, and what their quantifiable consequence to downstream CNV analyses might be. Thus, it is noteworthy that batch and array effects, albeit demonstrably reduced by MarkerMatch, remain an important consideration in downstream CNV association analyses. Finally, because we lacked access to adequate ancestrally diverse data, we did not examine the effects of ancestry composition on the MarkerMatch algorithm.

## Supplementary Material

btag341_Supplementary_Data

## Data Availability

The data used in this article were provided by the Simons Foundation for Autism Research and the Tourette Association of America International Consortium for Genetics by permission. Data are accessible through the Simons Foundation for Autism Research and the Tourette Association of America International Consortium for Genetics by request.
