# Supplementary material for: MarkerMatch: a proximity-based probe-matching algorithm for joint analysis of copy-number variants from different genotyping arrays
Source: Bioinformatics. 2026 May 24;42(6):btag341. doi: 10.1093/bioinformatics/btag341 (PMC13257853; doi:10.1093/bioinformatics/btag341)
Supplement: btag341_Supplementary_Data [file btag341_supplementary_data.zip › Ivankovic_Supplementary Information.pdf]

MarkerMatch: A Proximity-Based Probe-Matching Algorithm for Joint Analysis of Copy-Number Variants from Different Genotyping Arrays | Supplementary Information

MarkerMatch is available as an R package at: https://github.com/FranjoIM/MarkerMatch. The code used for analysis and implementation is available at: https://doi.org/10.5281/zenodo.18460979. The live notebook is available at https://fivankovic.notion.site/2026-markermatch.

Methods

Samples and Software

Simons Simplex Collection (SSC)

The Simons Simplex Collection (SSC) was collected by the Simons Foundation Autism Research Initiative (SFARI) and contains over 2000 families in which probands have moderate to severe symptoms of autism spectrum disorder (ASD) and relatively little intellectual disability (Fischbach and Lord 2010). Data from 1,069 such families (N = 4,239) were generated using Illumina Infinium Omni2.5 (OMNI) assay with ~2,440,000 genome-wide probes (Sanders et al. 2015).

Tourette Association of America International Consortium for Genetics (TAAICG)

The Tourette Association of America International Consortium for Genetics (TAAICG) is a collection of individuals with Tourette syndrome (TS) and their families, primarily from TS specialty clinics throughout North America, Europe, and Israel (Scharf et al. 2013). From this collection, we analyzed 3,920 individuals genotyped on Illumina Infinium OmniExpressExome (OEE) with ~960,000 probes (Huang et al. 2017) and 4,389 individuals genotyped on Illumina Infinium Global Screening Array v1 (GSA1) with ~700,000 probes.

Data Preprocessing

Clustering and Data Export

Intensity files from genotyping microarrays were imported and clustered de novo in Illumina GenomeStudio software (Illumina 2020). Subsequently, we exported log-R ratio (LRR) and B-allele frequency (BAF) intensity data for all markers and all samples for downstream analysis into an Illumina final report format. We also exported the manifest containing probe name, chromosome, position, and probe BAF, and mean and standard deviation of LRR. The resulting files were passed into the MarkerMatch script for matching. The code available in the GitHub repository.

Genotyping QC and Processing

In addition to processing data for CNV calling, genetic data were exported in Plink format from Illumina GenomeStudio for additional processing. The resulting Plink files were converted to bfile format. All datasets were processed through basic genotyping QC steps (Purcell et al. 2007; Marees et al. 2018). Namely, data were filtered to remove individuals with problematic sex information. For all datasets, only the autosomal SNPs were kept for further analysis. Serial, stepwise filtering was performed to retain only samples and SNPs with genotyping rate > 0.98. We then removed SNPs in Hardy-Weinberg disequilibrium by filtering out SNPs with $P_{HWE} < 10^{-6}$. Lastly, we removed individuals with unusually high or low heterozygosity by filtering out individuals with F_{HET} values outside of the three standard deviations of the cohorts' mean. Ultimately, we filtered out SNPs with MAF < 0.01. Only the samples passing this QC were retained in the validation study.

For the Cross-Array Experiment, we merged the individual batches into two TAAICG datasets: OEE and GSA1 (as determined by the array), each dataset filtered to retain only the intersection of SNPs from the respective array's batches. We then updated the datasets to only keep SNPs common to both OEE and GSA1 arrays, alleles of some SNPs in GSA1 were flipped to have the same allele configuration as those in OEE and rsIDs in GSA1 were updated to have the same rsID as those in OEE if the positions and alleles were matching. We then used the identity-by-descent approach to

identify and remove potential cross-contamination and monozygotic twins in each dataset. Finally, we merged OEE and GSA1 into a single Plink file, and ran a single-line QC to remove samples and SNPs with genotyping rate < 0.98, MAF < 0.01, and $P_{HWE} < 10^{-6}$. The resulting dataset was processed through identity-by-descent to identify “monozygotic twins” who in our case actually represented the same individuals genotyped on two different arrays. We used the resulting ID matches to update IDs to be identical in both TAAICG/OEE and TAAICG/GSA1 CNV callsets.

CNV Calling

Final reports exported from Illumina GenomeStudio were split into sample intensity files. We GC wave-adjusted the intensity files prior to CNV calling to reduce artifacts due to genomic waves in GC-content (Diskin et al. 2008). We then called CNVs using PennCNV as described in previous CNV studies (Huang et al. 2017).

CNVs were called individually, followed by merging of artificially broken up large CNVs through an iterative merging of adjacent CNV calls within single samples with a specified fraction parameter. The resulting PennCNV callset was frozen as the low-stringency QC callset. The final dataset included 62 PennCNV callsets.

CNV Quality Control

We applied the following QC metrics to derive the medium-stringency QC callset by removing samples with: (1) failing genotyping QC; (2) LRR mean outside of median +/- 4 median absolute deviations (MAD); (3) LRR standard deviation above median + 4 MAD; (4) BAF mean outside of median +/- 4 MAD; (5) BAF standard deviation above median + 4MAD; (6) BAF drift above median + 4 MAD; (7) GCWF outside median +/- 4 MAD; and (8) CNV count above callset median + 4 MAD.

We also removed CNVs based on the following criteria: (1) CNV spanning < 10 probes; and (2) CNV length < 20,000bp. CNV overlapping with telomeric, centromeric, immunoglobulin/T-cell receptor, and segmental duplication regions were annotated for further examination.

CNVs were additionally annotated by the PennCNV confidence decile, thresholds for which were determined by the full set, low stringency QC, Within-Array Experiment CNV callset.

CNV Call Validation

Partial Confusion Matrix and Performance Metrics

In the Within-Array Experiment (WAE), we used the full OMNI manifest CNV callset as the ground truth to which we compared intersection of OMNI manifest with GSA1 manifest and different configurations of GSA-matched OMNI manifests. In the Cross-Array Experiment (CAE), we used the full GSA1 and OEE manifest CNV callsets as external and internal ground truths, respectively, to which we compared intersection of OEE manifest with GSA1 manifest and different configurations of GSA-matched OEE manifests.

To facilitate accurate measurement, Plink was used to identify samples which have been genotyped using both OEE and GSA1 arrays; the remaining samples were removed from the analysis. For the WAE, the low-stringency QC full set was considered a reference callset. For the CAE, the low-stringency QC OEE full set was considered a reference callset for GSA1 marker-matched callsets, and the low-stringency QC GSA1 full set was considered a reference callset for OEE marker-matched callsets. Supplemental **Figure 1B** shows the graphical representation of both validation studies.

True positives (TP) were defined as CNVs in test callsets that overlapped CNVs called in the Full Set, low-stringency QC callset. False positives (FP) were defined as CNVs in test callsets that did not overlap CNVs called in the Full Set, low-stringency QC callset. False negatives (FN) were defined as CNVs in the Full Set, low-stringency QC callset that did not overlap CNVs in test callsets. Here, we use test callsets to describe all callsets except the Full Set, low-stringency QC callset.

The following validation metrics were considered: true positive rate (sensitivity), false negative rate (FNR), positive predictive value (PPV; precision), false discovery rate (FDR), F1 score (F1; harmonic mean of precision and recall), Fowlkes–Mallows index (FMI; geometric mean of precision

and recall); and Jaccard index (JI; ratio of the intersection to the union of the two sets).. To calculate these metrics, we used the equations **Eq. 1-7**.

In both WAE and CAE, we performed these analyses genome-wide and for CNVs overlapping centromeric, telomeric, and segmental duplications regions.

$$\text{Sensitivity} = TP / (TP + FN) = 1 - FNR \qquad \text{(Eq. 1)}$$

$$FNR = FN / (TP + FN) = 1 - \text{Sensitivity} \qquad \text{(Eq. 2)}$$

$$PPV = TP / (TP + FP) = 1 - FDR \qquad \text{(Eq. 3)}$$

$$FDR = FP / (TP + FP) = 1 - PPV \qquad \text{(Eq. 4)}$$

$$F1 = 2 * PPV * \text{Sensitivity} / (PPV + \text{Sensitivity}) \qquad \text{(Eq. 5)}$$

$$FMI = \text{sqrt}(PPV * \text{Sensitivity}) \qquad \text{(Eq. 6)}$$

$$JI = TP / (TP + FN + FP) \qquad \text{(Eq. 7)}$$

Optimizing D_{MAX} Parameter Selection

Only medium-stringency QC WAE callsets were used to optimize the D_{MAX} parameter selection. In order to do that, we have first re-calibrated the performance metrics by scaling them to the respective medium-stringency QC Full Set callset values, following the equation **Eq. 8**, where M_{CP} is a scaled performance metric, M_{CO} is original performance metric for that (subset of) callset, and M_{FS} is performance metric of (that subset's) Full Set. Only sensitivity, PPV, F1, FMI, and JI were included in this analysis. FNR and FDR were omitted as they are directly related to sensitivity and PPV, respectively.

$$M_{CP} = M_{CO} \ / \ M_{FS} \qquad \text{(Eq. 8)}$$

This was done to compensate for metric fluctuations of the Full Set performance metrics due to the quality control process. Once the metrics were scaled, they were re-plotted with loess smoothing

function, for all CNV sizes except very large CNVs (< 1Mb), as there were too few to have a proper loess linear model fit. The smoothing was done to enable easier detection of major inflection points in validity metrics along the D_{MAX} parameter values.

Optimizing Method Parameter Selection

Only medium-stringency QC WAE and CAE callsets were used to optimize the Method parameter selection. In order to do that, we have first re-calibrated the performance metrics according to the method described above. Once the metrics were scaled, they were-replotted as Method-specific boxplots, stratified by CNV type and CNV size (except very large CNVs, < 1Mb). Box plots and medians were inspected visually to determine which Methods were performing better.

Following that, we performed CNV type and CNV size-stratified Welch two-sample t-test, assuming unequal variances between the scaled metric values (sensitivity’, PPV’, F1’, FMI’, and JI’) between each combination of the four Method parameters, to evaluate whether the visual distribution shifts are statistically significant. We report both nominal p values, as well as FDR-adjusted q values.

Optimizing Minimum CNV Length and Probe Coverage Thresholds

We also performed analysis to evaluate the optimal CNV length and probe coverage thresholds for the quality control purposes using the CAE callsets. While the performance metric data may contain 0 and 1 values, there is nothing intrinsically important about such extreme metric values to warrant zero/one inflated beta modeling approach. In other words, 1 and 0.9999 and 0 and 0.0001 are essentially the same for our modeling purposes. Thus, we transformed the metrics to fit (0, 1) distribution as opposed to [0, 1] distribution using the equation **Eq. 9** (Smithson and Verkuilen 2006). This will allow us to model metrics using regular beta distribution, as opposed to piecewise zero-one inflated beta modeling.

$$\text{Metric (scaled)} = (\text{Metric} * (N - 1) + 0.5) / N \qquad \text{(Eq. 9)}$$

The multivariable beta regression models were fit associating various scaled and transformed performance metric values to CNV length cutoff (varied from 20kb to 500kb in 20kb increments), probe coverage cutoff (varied from 10 to 50 in the increments of 5), and Method (measured relative to LRR mean for all MarkerMatch Method parameter values), according to the equation **Eq. 10**, across the medium-stringency QC callsets for both GSA1 and OEE arrays (where each array was analyzed independently). Notably, for the mean model, we included an interaction term between CNV length cutoff and probe coverage cutoff, while for the precision model we did not include any interaction terms.

Metric ~ CNV length cutoff * probe coverage cutoff + Method |

CNV length cutoff + probe coverage cutoff + Method (Eq. 10)

In addition to regression models, we have plotted graphs showing scaled performance metrics relative to different combinations of CNV length cutoff and probe coverage cutoff, stratified by Method, D_{MAX}, and QC. Note units of CNV length cutoff are 10,000bp, e.g. value of 1=10,000bp, in the models. We also plotted performance metrics, specifically PPV and F1 score, at different CNV length and probe coverage cutoffs to see if most optimal cutoffs can be determined visually.

Sample-Wise Performance

In order to determine whether there are samples driving high rates of false-positives, we plotted sample-wise stacked TP and FP rates for visual examination. We then re-calculated performance metrics to determine whether this as a QC step would lead to dramatic improvements in performance. This step was done on both WAE and CAE data.

Results

Marker Matching

Inter-Probe Gaps

Analysis of inter-probe gaps (i.e., gaps between SNPs used by PennCNV to make CNV calls) shows successful reduction of gap-sizes between SNPs on both OMNI and OEE when matched with GSA1, relative to the typical Exact Match approach (**Supplemental Figure S1, Table 3, Supplemental Table S1C**). The reduction of inter-probe gaps in OMNI array when MarkerMatched to GSA1 at various Method and D_{MAX} parameters appears to plateau at about D_{MAX} = 10kb, where the median gaps were around 2.5kb, as compared to median gaps at Exact Match at about 11.2kb and Full Set at about 0.6kb (**Table 3, Supplemental Figure S1A**).

Similarly, when matching OEE to GSA1, Exact Match resulted in gaps with a median of about 10.9kb, D_{MAX} = 10kb MarkerMatch sets resulted in gaps with a median of about 2.8kb, and Full Set had gaps with a median of about 1.3kb on the OEE array (**Table 3, Supplemental Figure S1B**). Exact Match resulted in gaps with a median of about 10.9kb, D_{MAX} = 10kb MarkerMatch sets resulted in gaps with a median of about 2.8kb, and Full Set had gaps with a median of about 2.5kb on the GSA1 array (**Table 3, Supplemental Figure S1C**).

BAF

Analysis of BAFs shows that MarkerMatch approximates Full Set BAF distributions better than Exact Match. In the case of OMNI array, improvements in BAF plateaued at D_{MAX} = 10kb for all Method settings (**Supplemental Figure S2, Table 3, Supplemental Table S1D**). Notably, the median BAF differed for each Method, with median BAF being 0.05 for Method = BAF, 0.09 for Method = LRR mean, 0.12 for Method = LRR sd, and 0.11 for Method = Distance arrays, whereas the median BAF values for Exact Match and Full Set were 0.26 and 0.07, respectively (**Table 3, Supplemental Figure**

S2A). Notably, it appears that setting the Method parameter to BAF may result in a subset array with deflated BAF values.

When matching OEE to GSA1, the median BAF on OEE array also differed for each Method, with median BAF being 0.15 for Method = BAF, 0.18 for Method = LRR mean, 0.18 for Method = LRR sd, and 0.19 for Method = Distance arrays, whereas the median BAF values for Exact Match and Full Set were 0.25 and 0.14, respectively (**Table 3, Supplemental Figure S2B**). The median BAF on the GSA1 array was identical for each Method with the value of 0.05, whereas the median BAF values for Exact Match and Full Set were 0.25 and 0.4, respectively (**Table 3, Supplemental Figure S2C**).

LRR sd

Analysis of LRR sds has shown very little variability between the Full Set, Exact Match, and various configurations of the D_{MAX} and Method parameters of MarkerMatched arrays in OMNI, OEE, and GSA1 datasets (**Supplemental Figure S3, Table 3, Supplemental Table S1E**), with median LRR sd values all being between 1.25 and 1.35.

LRR mean

Analysis of LRR means has shown very little variability between the Full Set, Exact Match, and various configurations of the D_{MAX} and Method parameters of MarkerMatched arrays in OMNI, OEE, and GSA1 datasets (**Supplemental Figures S4-5, Table 3, Supplemental Table S1F**), with median LRR mean values all being around 0.

Within-Array Experiment

CNV Call Rate Summaries

Full Set, low-stringency QC callset had a per-sample average of 66.1 (st.dev = 20.7) CNV calls. The CNV call rate for deletions was substantially higher than that for duplications, 49.5 ± 19.0 and 16.5 ± 7.5 respectively. The results for medium-stringency callset followed similar trends: 11.2 ±

4.9 (Full Set deletions and duplications), 6.4 ± 3.3 (Full Set deletions), 4.7 ± 2.7 (Full Set duplications).

Exact Match, low-stringency QC callset had a per-sample average of 2.6 (st.dev = 1.8) CNV calls. The CNV call rate for deletions was slightly lower than that for duplications, 1.2 ± 1.2 and 1.4 ± 1.4 respectively. The results for medium-stringency callset followed similar trends: 0.4 ± 0.6 (Exact Match deletions and duplications), 0.1 ± 0.3 (Exact Match deletions), 0.3 ± 0.5 (Exact Match duplications).

D_{MAX} = 10kb, Method = BAF, low-stringency QC callset had a per-sample average of 12.7 (st.dev = 5.2). The CNV call rate for deletions was substantially higher than that for duplications, 8.1 ± 4.5 and 4.6 ± 2.6 respectively. The results for medium-stringency callset followed similar trends: 3.0 ± 1.2 (D_{MAX} = 10kb, Method = BAF deletions and duplications), 1.4 ± 1.2 (D_{MAX} = 10kb, Method = BAF deletions), 1.6 ± 1.4 (D_{MAX} = 10kb, Method = BAF duplications).

D_{MAX} = 10kb, Method = LRR mean, low-stringency QC callset had a per-sample average of 13.0 (st.dev = 5.6). The CNV call rate for deletions was substantially higher than that for duplications, 8.1 ± 4.9 and 4.9 ± 2.7 respectively. The results for medium-stringency callset followed similar trends: 3.1 ± 2.0 (D_{MAX} = 10kb, Method = LRR mean deletions and duplications), 1.4 ± 1.2 (D_{MAX} = 10kb, Method = LRR mean deletions), 1.7 ± 1.4 (D_{MAX} = 10kb, Method = LRR mean duplications).

D_{MAX} = 10kb, Method = LRR sd, low-stringency QC callset had a per-sample average of 13.5 (st.dev = 5.7). The CNV call rate for deletions was substantially higher than that for duplications, 8.2 ± 4.9 and 5.3 ± 2.9 respectively. The results for medium-stringency callset followed similar trends: 3.3 ± 2.0 (D_{MAX} = 10kb, Method = LRR sd deletions and duplications), 1.5 ± 1.3 (D_{MAX} = 10kb, Method = LRR sd deletions), 1.8 ± 1.4 (D_{MAX} = 10kb, Method = LRR sd duplications).

D_{MAX} = 10kb, Method = Distance, low-stringency QC callset had a per-sample average of 13.2 (st.dev = 5.1). The CNV call rate for deletions was substantially higher than that for duplications, 7.8 ± 4.4 and 5.5 ± 2.7 respectively. The results for medium-stringency callset followed similar trends: 3.3 ±

2.0 (D_{MAX} = 10kb, Method = Distance deletions and duplications), 1.5 ± 1.3 (D_{MAX} = 10kb, Method = Distance deletions), 1.8 ± 1.4 (D_{MAX} = 10kb, Method = Distance duplications).

Detailed reports for other D_{MAX} parameter settings (10bp, 50bp, 100bp, 500bp, 1kb, 5kb, 50kb, 100kb, 500kb, 1Mb, and 5Mb) are available in the **Supplemental Table S1G**.

CNV Size Summaries

Full Set, low-stringency QC callset had an average CNV size of 34.9kb (st.dev = 248.2kb). The average sizes of deletions were substantially smaller than those of duplications, 20.9kb ± 121.9kb and 76.9kb ± 446.4kb respectively. The results for medium-stringency callset followed similar trends: 109.8kb ± 376.5kb (Full Set deletions and duplications), 84.4kb ± 281.2kb (Full Set deletions), 144.2kb ± 474.2kb (Full Set duplications).

Exact Match, low-stringency QC callset had an average CNV size of 121.4kb (st.dev = 367.5kb). The average sizes of deletions were substantially smaller than those of duplications, 98.0kb ± 392.2kb and 140.7kb ± 344.7kb respectively. The results for medium-stringency callset followed similar trends: 405.6kb ± 853.0kb (Exact Match deletions and duplications), 466.5kb ± 1,179.8kb (Exact Match deletions), 381.8kb ± 683.8kb (Exact Match duplications).

D_{MAX} = 10kb, Method = BAF, low-stringency QC callset had an average CNV size of 67.0kb (st.dev = 285.2kb). The average sizes of deletions were substantially smaller than those of duplications, 43.9kb ± 174.7kb and 107.5kb ± 409.8kb respectively. The results for medium-stringency callset followed similar trends: 172.5kb ± 476.0kb (D_{MAX} = 10kb, Method = BAF deletions and duplications), 141.7kb ± 382.3kb (D_{MAX} = 10kb, Method = BAF deletions), 199.0kb ± 542.4kb (D_{MAX} = 10kb, Method = BAF duplications).

D_{MAX} = 10kb, Method = LRR mean, low-stringency QC callset had an average CNV size of 67.8kb (st.dev = 262.9kb). The average sizes of deletions were substantially smaller than those of duplications, 45.0kb ± 177.3kb and 105.3kb ± 358.9kb respectively. The results for medium-stringency callset followed similar trends: 173.3kb ± 420.8kb (D_{MAX} = 10kb, Method = LRR

mean deletions and duplications), 144.6kb ± 373.8kb (D_{MAX} = 10kb, Method = LRR mean deletions), 197.4kb ± 455.0kb (D_{MAX} = 10kb, Method = LRR mean duplications).

D_{MAX} = 10kb, Method = LRR sd, low-stringency QC callset had an average CNV size of 69.3kb (st.dev = 287.5kb). The average sizes of deletions were substantially smaller than those of duplications, 46.4kb ± 181.6kb and 104.3kb ± 395.9kb respectively. The results for medium-stringency callset followed similar trends: 173.2kb ± 459.1kb (D_{MAX} = 10kb, Method = LRR sd deletions and duplications), 144.1kb ± 371.3kb (D_{MAX} = 10kb, Method = LRR sd deletions), 197.3kb ± 519.7kb (D_{MAX} = 10kb, Method = LRR sd duplications).

D_{MAX} = 10kb, Method = Distance, low-stringency QC callset had an average CNV size of 69.6kb (st.dev = 288.1kb). The average sizes of deletions were substantially smaller than those of duplications, 49.2kb ± 180.9kb and 98.6kb ± 391.1kb respectively. The results for medium-stringency callset followed similar trends: 176.0kb ± 495.7kb (D_{MAX} = 10kb, Method = Distance deletions and duplications), 147.6kb ± 378.7kb (D_{MAX} = 10kb, Method = Distance deletions), 199.8kb ± 574.6kb (D_{MAX} = 10kb, Method = Distance duplications).

Detailed reports for other D_{MAX} parameter settings (10bp, 50bp, 100bp, 500bp, 1kb, 5kb, 50kb, 100kb, 500kb, 1Mb, and 5Mb) are available in the **Supplemental Table S1G**.

CNV Confidence Score Summaries

Full Set, low-stringency QC callset had an average CNV confidence score of 47.4 (st.dev = 171.2). The average confidence scores were roughly similar for deletions and duplications, 44.1 ± 176.1 and 57.3 ± 155.3 respectively. The results for medium-stringency callset followed similar trends, except with overall higher confidence scores: 133.7 ± 373.7 (Full Set deletions and duplications), 137.3 ± 439.7 (Full Set deletions), 128.8 ± 258.5 (Full Set duplications).

Exact Match, low-stringency QC callset had an average CNV confidence score of 31.2 (st.dev = 69.3). The average confidence scores were roughly similar for deletions and duplications, 29.1 ± 84.6 and 32.9 ± 53.5 respectively. The results for medium-stringency callset followed similar trends,

except with overall higher confidence scores: 93.3 ± 160.1 (Exact Match deletions and duplications), 112.1 ± 255.7 (Exact Match deletions), 86 ± 100.1 (Exact Match duplications).

D_{MAX} = 10kb, Method = BAF, low-stringency QC callset had an average CNV confidence score of 35.3 (st.dev = 109.0). The average confidence scores were roughly similar for deletions and duplications, 33.5 ± 125.3 and 38.3 ± 71.8 respectively. The results for medium-stringency callset followed similar trends, except with overall higher confidence scores: 80.3 ± 208.0 (D_{MAX} = 10kb, Method = BAF deletions and duplications), 88.6 ± 283.5 (D_{MAX} = 10kb, Method = BAF deletions), 73.3 ± 106.4 (D_{MAX} = 10kb, Method = BAF duplications).

D_{MAX} = 10kb, Method = LRR mean, low-stringency QC callset had an average CNV confidence score of 37.5 (st.dev = 115.8). The average confidence scores were roughly similar for deletions and duplications, 34.8 ± 131.1 and 41.9 ± 84.8 respectively. The results for medium-stringency callset followed similar trends, except with overall higher confidence scores: 86.5 ± 208.4 (D_{MAX} = 10kb, Method = LRR mean deletions and duplications), 91.0 ± 275.8 (D_{MAX} = 10kb, Method = LRR mean deletions), 82.7 ± 126.9 (D_{MAX} = 10kb, Method = LRR mean duplications).

D_{MAX} = 10kb, Method = LRR sd, low-stringency QC callset had an average CNV confidence score of 37.1 (st.dev = 114.6). The average confidence scores were roughly similar for deletions and duplications, 34.1 ± 130.8 and 41.8 ± 83.8 respectively. The results for medium-stringency callset followed similar trends, except with overall higher confidence scores: 86.1 ± 215.4 (D_{MAX} = 10kb, Method = LRR sd deletions and duplications), 87.6 ± 287.9 (D_{MAX} = 10kb, Method = LRR sd deletions), 84.8 ± 126.9 (D_{MAX} = 10kb, Method = LRR sd duplications).

D_{MAX} = 10kb, Method = Distance, low-stringency QC callset had an average CNV confidence score of 37.2 (st.dev = 106.1). The average confidence scores were roughly similar for deletions and duplications, 34.7 ± 121.0 and 40.8 ± 80.3 respectively. The results for medium-stringency callset followed similar trends, except with overall higher confidence scores: 83.8 ± 198.1 (D_{MAX} = 10kb,

Method = Distance deletions and duplications), 86.1 ± 261.1 (D_{MAX} = 10kb, Method = Distance deletions), 81.9 ± 122.6 (D_{MAX} = 10kb, Method = Distance duplications).

Detailed reports for other D_{MAX} parameter settings (10bp, 50bp, 100bp, 500bp, 1kb, 5kb, 50kb, 100kb, 500kb, 1Mb, and 5Mb) are available in the **Supplemental Table S1G**.

Sensitivity Analyses

The overall sensitivity of the Exact Match approach was 0.04 and 0.02, for low- and medium-stringency QC callsets, respectively. Sensitivity was higher for larger CNVs, with the medium-stringency QC sensitivities of 0 (under 100kb), 0.19 (100kb to 500kb), 0.53 (500kb to 1Mb), 0.35 (1Mb to 5Mb), and 0.32 (over 5Mb).

The MarkerMatch algorithm shows sensitivity to be similar across the different Method parameters. The sensitivity seems to grow before plateauing at about D_{MAX} = 10kb across all size bins, at about 0.06 (all sizes), 0.03 (under 100kb), 0.53 (100kb to 500kb), 0.62 (500kb to 1Mb), 0.37 (1Mb to 5Mb), and 0.4 (over 5Mb), averaged across all four Method parameters, at medium-stringency QC.

Full Set-specific analysis indicates a significant potential drop in sensitivity due to quality control process (medium-stringency QC callset shows over 75% reduction in sensitivity, at 0.24), however this effect seems to taper off among larger CNVs, with size-bin specific sensitivity reductions of about 81% (under 100kb),13% (100kb to 500kb), 8% (500kb to 1Mb), 25% (1Mb to 5Mb), and 22% (over 5Mb).

Detailed reports for other D_{MAX} parameter settings (10bp, 50bp, 100bp, 500bp, 1kb, 5kb, 50kb, 100kb, 500kb, 1Mb, and 5Mb) are available in the **Supplemental Table S1I**. Sensitivity WAE plots are available in **Supplemental Figure S6** for all CNV types, as well as **Supplemental Figures S12** and **S19** for deletions and duplications, respectively.

Positive Predictive Value Analyses

The overall positive predictive value (PPV) of the Exact Match approach was 0.82 and 1.00, for low- and medium-stringency QC callsets, respectively. PPV for the Exact Match was 1.00 across the board, regardless of the CNV size.

The MarkerMatch algorithm shows PPV to be similar across the different Method parameters. The PPV seems to be constant at 1.00 until about D_{MAX} = 10kb (all sizes) before it begins to decrease, averaged across all four Method parameters, at medium-stringency QC.

Detailed reports for other D_{MAX} parameter settings (10bp, 50bp, 100bp, 500bp, 1kb, 5kb, 50kb, 100kb, 500kb, 1Mb, and 5Mb) are available in the **Supplemental Table S1I**. PPV WAE plots are available in **Figure 4** for all CNV types, as well as **Supplemental Figures S13** and **S20** for deletions and duplications, respectively.

False Negative Rate Analyses

The false negative rate (FNR) is a complement to sensitivity, as per equations **Eq. 1** and **Eq. 3**. Overall, FNRs appear to be highest for the Exact Match. FNR tends to drop with larger-size CNV bins. A detailed FNR report is available in the **Supplemental Table S1I**. FNR WAE plots are available in **Supplemental Figure S7** for all CNV types, as well as **Supplemental Figures S14** and **S21** for deletions and duplications, respectively.

False Discovery Rate Analyses

The false discovery rate (FDR) is a complement to PPV, as per equations **Eq. 2** and **Eq. 4**. A detailed FDR report is available in the **Supplemental Table S1I**. FDR WAE plots are available in **Supplemental Figure S8** for all CNV types, as well as **Supplemental Figures S15** and **S22** for deletions and duplications, respectively.

F1 Score Analyses

F1 score for MarkerMatch continuously outperformed the Exact Match callset, when compared to the Full Set callset, with an exception of large CNV subsets of D_{MAX} < 100bp callsets. Similarly to

other metrics, F1 scores plateau around D_{MAX} = 10kb and appear to be consistent across different Method parameters.

The overall F1 score (F1) of the Exact Match approach was 0.07 and 0.08, for low- and medium-stringency QC callsets, respectively. The F1 seems to grow before plateauing at about D_{MAX} = 10kb across all size bins, at about 0.09 (all sizes), 0.04 (under 100kb), 0.61 (100kb to 500kb), 0.68 (500kb to 1Mb), 0.45 (1Mb to 5Mb), and 0.60 (over 5Mb), averaged across all four Method parameters, at medium-stringency QC.

A detailed F1 report is available in the **Supplemental Table S1I**. F1 WAE plots are available in **Supplemental Figure S9** for all CNV types, as well as **Supplemental Figures S16** and **S23** for deletions and duplications, respectively.

Fowlkes-Mallows Index Analyses

Similarly to F1, the Fowlkes-Mallows index (FMI) continuously outperformed the Exact Match callset, when compared to the Full Set callset, with an exception of large CNV subsets of D_{MAX} < 100bp callsets. Similarly to other metrics, FMIs plateau around D_{MAX} = 10kb and appear to be consistent across different Method parameters. A detailed FMI report is available in the **Supplemental Table S1I**. FMI WAE plots are available in **Supplemental Figure S10** for all CNV types, as well as **Supplemental Figures S17** and **S24** for deletions and duplications, respectively.

Jaccard Index Analyses

Similarly to both FMI and F1, and sensitivity, the Jaccard index (JI) continuously outperformed the Exact Match callset, when compared to the Full Set callset, with an exception of large CNV subsets of D_{MAX} < 100bp callsets. Similarly to other metrics, JIs plateau around D_{MAX} = 10kb and appear to be consistent across different Method parameters. A detailed JI report is available in the **Supplemental Table S1I**. JIWAE plots are available in **Supplemental Figure S11** for all CNV types, as well as **Supplemental Figures S18** and **S25** for deletions and duplications, respectively.

Deletions vs. Duplications

Deletions and duplications followed roughly the same trend when it came to validation metrics, with some exceptions. Namely, in Exact Match, in the medium-stringency QC callset, duplications had about 5-fold higher sensitivity and JI, about 10-fold higher F1, and about 3-fold higher FMI. The similar was the case with MarkerMatch callsets for all Method parameters and D_{MAX} = 10kb, specifically, values for sensitivity, FDR, F1, FMI, and JI, which were higher for deletions than duplications, on average. Values for PPV and FNR were roughly the same for deletions and duplications.

Detailed reports for other D_{MAX} parameter settings (10bp, 50bp, 100bp, 500bp, 1kb, 5kb, 50kb, 100kb, 500kb, 1Mb, and 5Mb) are available in the **Supplemental Table S1I**. These data have been graphically shown for deletions and duplications in the **Supplemental Figures S12-25**.

Cross-Array Experiment

CNV Call Rate Summaries

Full Set low-stringency QC callset from the GSA1 array had a per-sample average of 22.1 (st.dev = 17.3) CNV calls. The CNV call rate for deletions was slightly higher compared to that for duplications, 12.3 ± 12.0 and 9.8 ± 15.3, respectively. The results for medium-stringency QC callset were less divergent: 3.5 ± 2.5 (Full Set deletions and duplications), 1.7 ± 1.6 (Full Set deletions), 1.8 ± 1.7 (Full Set duplications). The low-stringency QC callset from the OEE array had a per-sample average of 29.1 (st.dev = 37.9) CNV calls. The CNV call rate for deletions was noticeably higher than that for duplications, 20.9 ± 35.2 and 8.2 ± 8.0, respectively. The results for the medium-stringency QC callset followed somewhat different trends, with deletions and duplications being more similar: 2.7 ± 2.8 (Full Set deletions and duplications), 1.4 ± 1.7 (Full Set deletions), 1.3 ± 1.6 (Full Set duplications).

Exact Match low-stringency QC callset from the GSA1 array had a per-sample average of 3.6 (st.dev = 2.4) CNV calls. The CNV call rate for deletions was about the same as that for duplications,

1.8 ± 2.0 and 1.8 ± 1.7, respectively. The results for medium-stringency QC callset had deletions slightly less frequent than duplications: 0.4 ± 0.6 (Exact Match deletions and duplications), 0.1 ± 0.4 (Exact Match deletions), 0.3 ± 0.5 (Exact Match duplications). The low-stringency QC callset from the OEE array had a per-sample average of 2.8 (st.dev = 2.1) CNV calls. The CNV call rate for deletions was somewhat higher than that for duplications, 1.6 ± 2.0 and 1.2 ± 1.1, respectively. The results for the medium-stringency QC callset followed somewhat different trends, with deletions and duplications being more similar: 0.3 ± 0.6 (Exact Match deletions and duplications), 0.1 ± 0.4 (Exact Match deletions), 0.2 ± 0.5 (Exact Match duplications).

The call rates for MarkerMatched callsets were typically between the FullSet and ExactMatch, with deletion call rate either matching or outpacing that of duplications in low-stringency callsets and vice-verse in medium-stringency callsets. Detailed reports for other strata are available in the **Supplemental Table S1L**.

CNV Size Summaries

Full Set low-stringency QC callset from the GSA1 array had an average CNV size of 73.8kb (st.dev = 336.7kb). The average sizes of deletions were substantially smaller than those for duplications, 61.6kb ± 326.3kb and 89.1kb ± 348.8kb, respectively. The results for medium-stringency QC callset followed similar trends: 141.9kb ± 272.4kb (Full Set deletions and duplications), 126.7kb ± 324.2kb (Full Set deletions), 156.6kb ± 210.0kb (Full Set duplications). The low-stringency QC callset from the OEE array had an average CNV size of 142.4kb (st.dev = 3.4Mb). The average sizes of deletions were substantially larger than those for duplications, 165.0kb ± 4.1Mb and 85.3kb ± 382.7kb, respectively. The results for the medium-stringency QC showed inverse trends: 123.1kb ± 176.8kb (Full Set deletions and duplications), 99.2kb ± 153.6kb (Full Set deletions), 148.3kb ± 195.3kb (Full Set duplications).

Exact Match low-stringency QC callset from the GSA1 array had an average CNV size of 73.8kb (st.dev = 336.7kb). The average sizes of deletions were substantially smaller than those for

duplications, 61.6kb ± 326.3kb and 89.1 ± 348.8kb, respectively. The results for medium-stringency QC callset followed similar trends: 141.9kb ± 272.4kb (Exact Match deletions and duplications), 126.7kb ± 324.2kb (Exact Match deletions), 156.6kb ± 210.0kb (Exact Match duplications). The low-stringency QC callset from the OEE array had an average CNV size of 142.4kb (st.dev = 3.4Mb). The average sizes of deletions were substantially larger than those for duplications, 165.0kb ± 4.1Mb and 85.3kb ± 382.7kb, respectively. The results for the medium-stringency QC callset followed inverse trends: 123.1kb ± 176.8kb (Exact Match deletions and duplications), 99.2kb ± 153.6kb (Exact Match deletions), 148.3kb ± 195.2kb (Exact Match duplications).

The CNV sizes for MarkerMatched callsets were typically between the FullSet and ExactMatch, with deletion call rate either matching or outpacing that of duplications in low-stringency callsets and vice-verse in medium-stringency callsets. Detailed reports for other strata are available in the **Supplemental Table S1L**.

CNV Confidence Score Summaries

Full Set low-stringency QC callset from the GSA1 array had an average CNV confidence score of 25.4 (st.dev = 49.7). The confidence scores of deletions were similar to those for duplications, 25.4 ± 48.9 and 25.5 ± 50.7, respectively. The results for medium-stringency QC callset followed slightly different trends, with deletions having somewhat higher scores: 61.3 ± 100.2 (Full Set deletions and duplications), 63.3 ± 99.7 (Full Set deletions), 59.4 ± 100.7 (Full Set duplications). The low-stringency QC callset from the OEE array had an average CNV confidence score of 33.3 (st.dev = 64.6). The average confidence scores of deletions were somewhat lower than those for duplications, 30.2 ± 54.7 and 41.2 ± 84.1, respectively. The results for the medium-stringency QC callset followed similar trends: 102.4 ± 152.1 (Full Set deletions and duplications), 96.7 ± 138.3 (Full Set deletions), 108.4 ± 165.3 (Full Set duplications).

Exact Match low-stringency QC callset from the GSA1 array had an average CNV confidence score of 24.2 (st.dev = 38.1). The confidence scores of deletions were slightly smaller than those for

duplications, 21.7 ± 33.7 and 26.7 ± 41.9, respectively. The results for medium-stringency QC callset showed similar CNV scores between all CNV types: 86.3 ± 83.7 (Exact Match deletions and duplications), 87.2 ± 83.7 (Exact Match deletions), 85.8 ± 83.9 (Exact Match duplications). The low-stringency QC callset from the OEE array had an average CNV confidence score of 27.5 (st.dev = 49.0). The average confidence scores of deletions were noticeably lower than those for duplications, 22.8 ± 31.1 and 33.7 ± 46.8, respectively. The results for medium-stringency QC callset showed similar CNV scores between all CNV types: 82.5 ± 72.4 (Exact Match deletions and duplications), 84.0 ± 65.3 (Exact Match deletions), 81.7 ± 76.1 (Exact Match duplications).

The CNV confidence scores for MarkerMatched callsets were typically between the FullSet and ExactMatch. Detailed reports for other strata are available in the **Supplemental Table S1L**.

Sensitivity Analyses

The overall sensitivity of the Exact Match approach on the GSA1 array was 0.08 and 0.04, and on the OEE array was 0.10 and 0.06, for low- and medium-stringency QC callsets respectively. In the medium-stringency QC GSA1 callset, the sensitivity was higher for larger CNVs in both arrays. The MarkerMatch algorithm shows sensitivity to be similar across the different Method parameters on both arrays, typically outperforming Exact Match.

Full Set sensitivity analysis indicates just how small the overlap of surveyed genomic regions is between the two arrays, with GSA1 array only ever capturing 30.0% of CNVs called on OEE array, and OEE capturing only 40.0% of CNVs called on GSA1 array. In addition to the batch-wise reduction in sensitivity, there also seem to be QC-related sensitivity reductions (e.g., lower sensitivity in OEE low- vs. medium-stringency QC callset, 18% vs. 40% respectively).

Detailed report is available in the **Supplemental Table S1N**. Sensitivity CAE plots are available in **Supplemental Figure S43** for all CNV types, as well as **Supplemental Figures S49** and **S56** for deletions and duplications, respectively.

Positive Predictive Value Analyses

PPV in both arrays shows slightly lower performance relative to Exact Match, but better performance relative to Full Set. The gap with Exact Match is very low in large CNVs (<100kb) and virtually nonexistent in very large CNVs (<500kb). Detailed report is available in the **Supplemental Table S1N**. Sensitivity CAE plots are available in **Figure 5** for all CNV types, as well as **Supplemental Figures S50** and **S57** for deletions and duplications, respectively.

False Negative Rate Analyses

The false negative rate (FNR) is a complement to sensitivity, as per equations **Eq. 1** and **Eq. 3**. A detailed FNR report is available in the **Supplemental Table S1N**. FNR CAE plots are available in **Supplemental Figure S44** for all CNV types, as well as **Supplemental Figures S51** and **S58** for deletions and duplications, respectively.

False Discovery Rate Analyses

The false discovery rate (FDR) is a complement to PPV, as per equations **Eq. 2** and **Eq. 4**. A detailed FDR report is available in the **Supplemental Table S1N**. FDR CAE plots are available in **Supplemental Figure S45** for all CNV types, as well as **Supplemental Figures S52** and **S59** for deletions and duplications, respectively.

F1 Score Analyses

F1 score for MarkerMatch performed either as well as Exact Match and Full Set, or better, for GSA1 array and slightly worse than Full Set in OEE array. This effect seemed to follow in CNV size-stratified cases for all CNV-size and array configurations, with the exception of large CNVs (over 500kb) on the OEE array, where Full Set performed noticeably better than MarkerMatch or Exact Match callsets. A detailed F1 report is available in the **Supplemental Table S1N**. F1 CAE plots are available in **Supplemental Figure S46** for all CNV types, as well as **Supplemental Figures S53** and **S60** for deletions and duplications, respectively.

Fowlkes-Mallows Index Analyses

The results for FMI were similar to those of F1 in the CAE. A detailed FMI report is available in the **Supplemental Table S1N**. FMI CAE plots are available in **Supplemental Figure S47** for all CNV types, as well as **Supplemental Figures S54** and **S61** for deletions and duplications, respectively.

Jaccard Index Analyses

The results for JI were similar to those of F1 and FMI in the CAE. A detailed JI report is available in the **Supplemental Table S1N**. JI CAE plots are available in **Supplemental Figure S48** for all CNV types, as well as **Supplemental Figures S55** and **S62** for deletions and duplications, respectively.

Deletions vs. Duplications

A detailed report is available in the **Supplemental Table S1N**. These data have been graphically shown for deletions and duplications in the **Supplemental Figures S49-62**.

Determination of Optimal Minimum CNV Size and SNP Coverage Thresholds

Beta regression of sensitivity on the GSA1 array has shown CNV length cutoff, probe coverage cutoff, and their interaction terms to be significantly associated ([OR_{TPR} = 1.05, p_{TPR} < 0.001]; [OR_{TPR} = 0.99, p_{TPR} < 0.001]; [OR_{TPR} = 1.00, p_{TPR} < 0.001] respectively). In the OEE array, we observed similar associations ([OR_{TPR} = 1.06, p_{TPR} < 0.001]; [OR_{TPR} = 1.02, p_{TPR} < 0.001]; [OR_{TPR} = 1.00, p_{TPR} < 0.001] respectively). While the associations were significant, the effect sizes were rather small. The model's pseudo R^2 were 0.87 and 0.95 for GSA1 and OEE, respectively. In both arrays, Method terms were either insignificant or, where significant, with limited effect sizes. In the GSA1 array, Distance showed slight underperformance [OR_{TPR} = 0.91, p_{TPR} = 0.001] relative to LRR mean. In the OEE array, BAF showed slight overperformance [OR_{TPR} = 1.07, p_{TPR} < 0.001] relative to LRR mean.

Beta regression of PPV in the GSA1 array indicated positive association with BAF [OR_{PPV} = 1.14, p_{PPV} = 0.046] and Distance [OR_{PPV} = 1.25, p_{PPV} < 0.001] Method terms, relative to LRR mean. Similarly to sensitivity, association between PPV and CNV length cutoff, probe coverage cutoff, and

their interaction terms was positive and significant ([OR_{TPR} = 1.10, p_{TPR} < 0.001]; [OR_{TPR} = 1.08, p_{TPR} < 0.001]; [OR_{TPR} = 1.00, p_{TPR} < 0.001] respectively). Model pseudo R^2 was 0.26. In the case of the OEE array, only the BAF Method was significantly associated with PPV [OR_{PPV} = 1.13, p_{PPV} = 0.007]. Association with CNV length cutoff, probe coverage cutoff, and their interaction terms was similar to the GSA1 array. Model pseudo R^2 was 0.37.

Beta regressions of F1, FMI, and JI scores have indicated trends similar to PPV and sensitivity above. An inspection of the plots displaying PPV and F1 scores across various breakdowns of CNV length and probe coverage cutoffs shows size-dependent diminishing of batch (array) and MarkerMatch/Method effects (**Supplemental Figures S70-75**). While it is evident larger CNV sizes and higher probe coverage are generally favorable, there are no specific recommendable cutoff values that drastically improve either PPV or F1, and any improvements are far from perfect.

We include model summaries below.

GSA1 Sensitivity (Pseudo R^2 = 0.87)

Parameter	Coefficient	SE	z	p
(Intercept)	0.8206867	3.890491e-02	-4.1686018	3.064739e-05
CNV Length Cutoff	1.0506034	1.974039e-03	26.2723759	3.968200e-152
Probe Coverage Cutoff	0.9882037	1.496797e-03	-7.8343629	4.712258e-15
Method: LRR sd	1.0123897	2.975370e-02	0.4189761	6.752336e-01
Method: BAF	1.0192850	3.010433e-02	0.6467434	5.177980e-01
Method: Distance	0.9112510	2.626960e-02	-3.2238343	1.264866e-03
CNV Length : Probe Coverage	1.0007716	6.670642e-05	11.5713026	5.760146e-31

OEE Sensitivity (Pseudo R^2 = 0.95)

Parameter	Coefficient	SE	z	p
(Intercept)	0.3572785	4.830658e-03	-76.1232159	0.000000e+00
CNV Length Cutoff	1.0629909	6.445882e-04	100.7378236	0.000000e+00
Probe Coverage Cutoff	1.0245529	4.345388e-04	57.1912943	0.000000e+00
Method: LRR sd	0.9918905	9.060354e-03	-0.8914094	3.727096e-01
Method: BAF	1.0712383	1.031571e-02	7.1461462	8.924803e-13
Method: Distance	1.0086641	9.233035e-03	0.9424377	3.459686e-01
CNV Length : Probe Coverage	0.9994262	2.164833e-05	-26.4985462	1.007355e-154

GSA1 PPV (Pseudo R^2 = 0.25)

Parameter	Coefficient	SE	z	p
(Intercept)	1.1524109	0.1007036140	1.6233444	1.045158e-01
CNV Length Cutoff	1.0970100	0.0047551935	21.3598586	3.157659e-101
Probe Coverage Cutoff	1.0768512	0.0029494345	27.0327603	6.091717e-161
Method: LRR sd	1.0366988	0.0687551471	0.5434378	5.868284e-01
Method: BAF	1.1401466	0.0748043665	1.9990546	4.560245e-02
Method: Distance	1.2534073	0.0820957513	3.4484327	5.638500e-04
CNV Length : Probe Coverage	0.9984796	0.0001345965	-11.2875601	1.511750e-29

OEE PPV (Pseudo R^2 = 0.37)

Parameter	Coefficient	SE	z	p
(Intercept)	2.2422580	0.1413442538	12.8097612	1.445787e-37
CNV Length Cutoff	1.0337236	0.0031001026	11.0596085	1.969554e-28
Probe Coverage Cutoff	1.0610667	0.0021379281	29.4183954	3.195075e-190
Method: LRR sd	0.9830258	0.0418762368	-0.4018812	6.877714e-01
Method: BAF	1.1341758	0.0526180738	2.7138927	6.649769e-03
Method: Distance	1.0792370	0.0507671389	1.6210589	1.050050e-01
CNV Length : Probe Coverage	0.9996064	0.0001026302	-3.8339241	1.261151e-04

GSA1 F1 (Pseudo R^2 = 0.85)

Parameter	Coefficient	SE	z	p
(Intercept)	1.2118369	5.399755e-02	4.3120294	1.617629e-05
CNV Length Cutoff	1.0568041	2.024202e-03	28.8448331	5.882339e-183
Probe Coverage Cutoff	0.9953196	1.379358e-03	-3.3852088	7.112414e-04
Method: LRR sd	1.0104802	2.918984e-02	0.3609112	7.181658e-01
Method: BAF	1.0217359	2.951330e-02	0.7444256	4.566190e-01
Method: Distance	0.9348388	2.643594e-02	-2.3827608	1.718336e-02
CNV Length : Probe Coverage	1.0005335	6.665875e-05	8.0052328	1.192409e-15

OEE F1 (Pseudo R^2 = 0.96)

Parameter	Coefficient	SE	z	p
(Intercept)	0.6215814	9.186466e-03	-32.1728458	4.233094e-227
CNV Length Cutoff	1.0600607	6.328502e-04	97.6997597	0.000000e+00
Probe Coverage Cutoff	1.0265713	4.814318e-04	55.9190999	0.000000e+00
Method: LRR sd	1.0034898	9.681120e-03	0.3610994	7.180251e-01
Method: BAF	1.0942104	1.145941e-02	8.5968688	8.192057e-18
Method: Distance	1.0274454	9.890514e-03	2.8126617	4.913331e-03
CNV Length : Probe Coverage	0.9994759	2.112916e-05	-24.7995466	9.066452e-136

GSA1 FMI (Pseudo R^2 = 0.85)

Parameter	Coefficient	SE	z	p
(Intercept)	1.2869238	5.472099e-02	5.9325068	2.983440e-09
CNV Length Cutoff	1.0558604	1.935216e-03	29.6568177	2.770108e-193
Probe Coverage Cutoff	0.9993004	1.312102e-03	-0.5329909	5.940399e-01
Method: LRR sd	1.0090898	2.784860e-02	0.3278796	7.430027e-01
Method: BAF	1.0227167	2.815420e-02	0.8159642	4.145206e-01
Method: Distance	0.9471422	2.564013e-02	-2.0060545	4.485044e-02
CNV Length : Probe Coverage	1.0004114	6.309582e-05	6.5219451	6.940144e-11

OEE FMI (Pseudo R^2 = 0.96)

Parameter	Coefficient	SE	z	p
(Intercept)	0.7540753	1.214734e-02	-17.5221573	9.707362e-69
CNV Length Cutoff	1.0547499	6.718000e-04	83.6886982	0.000000e+00
Probe Coverage Cutoff	1.0252156	5.075343e-04	50.3037416	0.000000e+00
Method: LRR sd	1.0077020	1.042018e-02	0.7419773	4.581011e-01
Method: BAF	1.0999262	1.185133e-02	8.8395441	9.610997e-19
Method: Distance	1.0330797	1.062158e-02	3.1653350	1.549045e-03
CNV Length : Probe Coverage	0.9995378	2.160254e-05	-21.3922538	1.577497e-101

GSA1 PPV (Pseudo R^2 = 0.85)

Parameter	Coefficient	SE	z	p
(Intercept)	0.6103213	2.816647e-02	-10.6991810	1.026818e-26
CNV Length Cutoff	1.0569370	2.082604e-03	28.1032629	8.936779e-174
Probe Coverage Cutoff	0.9950409	1.442762e-03	-3.4287026	6.064736e-04
Method: LRR sd	1.0103778	2.954177e-02	0.3531094	7.240064e-01
Method: BAF	1.0222640	2.986481e-02	0.7537317	4.510103e-01
Method: Distance	0.9305211	2.675241e-02	-2.5047211	1.225480e-02
CNV Length : Probe Coverage	1.0005628	6.869367e-05	8.1951057	2.503730e-16

OEE PPV (Pseudo R^2 = 0.96)

Parameter	Coefficient	SE	z	p
(Intercept)	0.3108267	4.681315e-03	-77.5865596	0.000000e+00
CNV Length Cutoff	1.0601514	6.378029e-04	97.0914983	0.000000e+00
Probe Coverage Cutoff	1.0265838	4.828447e-04	55.7819344	0.000000e+00
Method: LRR sd	1.0037292	9.701666e-03	0.3851034	7.001608e-01
Method: BAF	1.0983226	1.152737e-02	8.9356994	4.046108e-19
Method: Distance	1.0278771	9.919101e-03	2.8492649	4.382038e-03
CNV Length : Probe Coverage	0.9994727	2.118577e-05	-24.8820960	1.162616e-136

References

Diskin SJ, Li M, Hou C *et al.* Adjustment of genomic waves in signal intensities from whole-genome SNP genotyping platforms. *Nucleic Acids Research* 2008;**36**:e126–e126.

Fischbach GD, Lord C. The Simons Simplex Collection: A Resource for Identification of Autism Genetic Risk Factors. *Neuron* 2010;**68**:192–5.

Huang AY, Yu D, Davis LK *et al.* Rare Copy Number Variants in NRXN1 and CNTN6 Increase Risk for Tourette Syndrome. *Neuron* 2017;**94**:1101-1111.e7.

Illumina. GenomeStudio. 2020.

Marees AT, De Kluiver H, Stringer S *et al.* A tutorial on conducting genome‐wide association studies: Quality control and statistical analysis. *Int J Methods Psych Res* 2018;**27**:e1608.

Purcell S, Neale B, Todd-Brown K *et al.* PLINK: A Tool Set for Whole-Genome Association and Population-Based Linkage Analyses. *The American Journal of Human Genetics* 2007;**81**:559–75.

Sanders SJ, He X, Willsey AJ *et al.* Insights into Autism Spectrum Disorder Genomic Architecture and Biology from 71 Risk Loci. *Neuron* 2015;**87**:1215–33.

Scharf JM, Yu D, Mathews CA *et al.* Genome-wide association study of Tourette's syndrome. *Mol Psychiatry* 2013;**18**:721–8.

Smithson M, Verkuilen J. A better lemon squeezer? Maximum-likelihood regression with beta-distributed dependent variables. *Psychological Methods* 2006;**11**:54–71.

Supplementary Tables

**Supplementary Table S1**
**Table S1A**. Tabulated results of execution times (S1A: Execution Times).
**Table S1B**. Tabulated array coverage metrics (S1B: Array Coverage).
**Table S1C**. Tabulated inter-marker gap statistics (S1C: Inter-Marker Gaps).
**Table S1D**. Tabulated BAF metric statistics (S1D: BAF Metric).
**Table S1E**. Tabulated LRR sd metric statistics (S1E: LRR sd Metric).
**Table S1F**. Tabulated LRR mean metric statistics (S1F: LRR mean Metric).
**Table S1G**. Tabulated Within-Array Experiment callset summaries (S1G: WAE Callset Summaries).
**Table S1H**. Tabulated Within-Array Experiment callset sample summaries (S1H: WAE Callset Sample Summaries).
**Table S1I**. Tabulated Within-Array Experiment callset analytics (S1I: WAE Genome-Wide Callset Analytics).
**Table S1J**. Tabulated Within-Array Experiment callset analytics (S1J: WAE Regional Callset Analytics).
**Table S1K**. Tabulated Within-Array Experiment Method modeling (S1K: WAE Method Modeling).
**Table S1L**. Tabulated Cross-Array Experiment callset summaries (S1L: CAE Callset Summaries).
**Table S1M**. Tabulated Cross-Array Experiment callset sample summaries (S1M: CAE Callset Sample Summaries).
**Table S1N**. Tabulated Cross-Array Experiment callset analytics (S1N: CAE Genome-Wide Callset Analytics).
**Table S1O**. Tabulated Cross-Array Experiment callset analytics (S1O: CAE Regional Callset Analytics).
**Table S1P**. Tabulated sample-wise performance (S1P: Sample-WIse Performance).
**Table S1Q**. Tabulated cumulative metric-wise performance (S1Q: Cumulative Metric-Wise Performance).

**Table 2A**. Table summarizing sample counts within specified ranges of PPV for medium-stringency QC callsets.																		
**PPV Values**	**Full Set**			**Distance**			**BAF**			**LRR mean**			**LRR sd**			**Exact Match**		
	**OMNI**	**GSA1**	**OEE**	**OMNI**	**GSA1**	**OEE**	**OMNI**	**GSA1**	**OEE**	**OMNI**	**GSA1**	**OEE**	**OMNI**	**GSA1**	**OEE**	**OMNI**	**GSA1**	**OEE**
0.0	0	14	9	8	10	10	2	13	9	2	12	7	5	10	10	1	5	1
(0.0, 0.1)	0	0	0	0	0	0	0	0	0	0	0	0	0	0	0	0	0	0
[0.1, 0.2)	0	4	3	0	0	0	0	0	0	0	2	0	0	1	0	0	0	0
[0.2, 0.3)	0	12	6	0	6	2	0	3	2	0	6	2	0	3	1	0	0	0
[0.3, 0.4)	0	23	14	0	7	3	0	4	5	1	5	8	0	6	6	0	0	0
[0.4, 0.5)	0	15	8	0	1	0	0	1	1	0	2	0	1	3	1	0	0	0
[0.5, 0.6)	0	70	46	15	29	29	16	38	31	11	40	25	12	38	22	1	1	0
[0.6, 0.7)	0	95	61	25	44	21	11	45	25	21	41	27	20	44	29	0	0	0
[0.7, 0.8)	0	66	49	29	18	20	16	16	13	18	17	8	21	21	11	0	0	0
[0.8, 0.9)	0	60	64	79	24	14	40	26	20	30	26	16	44	26	11	0	0	0
[0.9, 1.0)	0	1	1	1	0	0	0	0	0	1	0	0	0	0	0	0	0	0
1.0	3,827	172	133	3,665	360	448	3,676	356	386	3,692	350	439	3,722	353	452	1,243	213	176

**Table 2B**. Table summarizing sample counts within specified ranges of F1 for medium-stringency QC callsets.																		
**F1 Values**	**Full Set**			**Distance**			**BAF**			**LRR mean**			**LRR sd**			**Exact Match**		
	**OMNI**	**GSA1**	**OEE**	**OMNI**	**GSA1**	**OEE**	**OMNI**	**GSA1**	**OEE**	**OMNI**	**GSA1**	**OEE**	**OMNI**	**GSA1**	**OEE**	**OMNI**	**GSA1**	**OEE**
0.0	0	14	9	8	10	10	2	13	9	2	12	7	5	10	10	1	5	1
(0.0, 0.1)	1	66	35	1,630	90	78	1,856	83	66	1,770	90	72	1,690	89	73	1,229	108	57
[0.1, 0.2)	55	120	65	1,872	152	144	1,685	153	143	1,766	155	141	1,836	155	145	15	91	89
[0.2, 0.3)	640	144	72	306	138	142	213	137	130	227	134	141	282	135	147	0	13	24
[0.3, 0.4)	1,445	116	82	6	68	79	5	72	73	11	72	88	12	72	82	0	0	5
[0.4, 0.5)	1,221	50	64	0	30	63	0	31	49	0	29	50	0	30	56	0	2	1
[0.5, 0.6)	417	17	46	0	7	19	0	9	16	0	6	23	0	11	19	0	0	0
[0.6, 0.7)	46	4	17	0	4	9	0	4	4	0	3	9	0	3	9	0	0	0
[0.7, 0.8)	2	1	4	0	0	3	0	0	2	0	0	1	0	0	2	0	0	0
[0.8, 0.9)	0	0	0	0	0	0	0	0	0	0	0	0	0	0	0	0	0	0
[0.9, 1.0)	0	0	0	0	0	0	0	0	0	0	0	0	0	0	0	0	0	0
1.0	0	0	0	0	0	0	0	0	0	0	0	0	0	0	0	0	0	0

Supplementary Figures

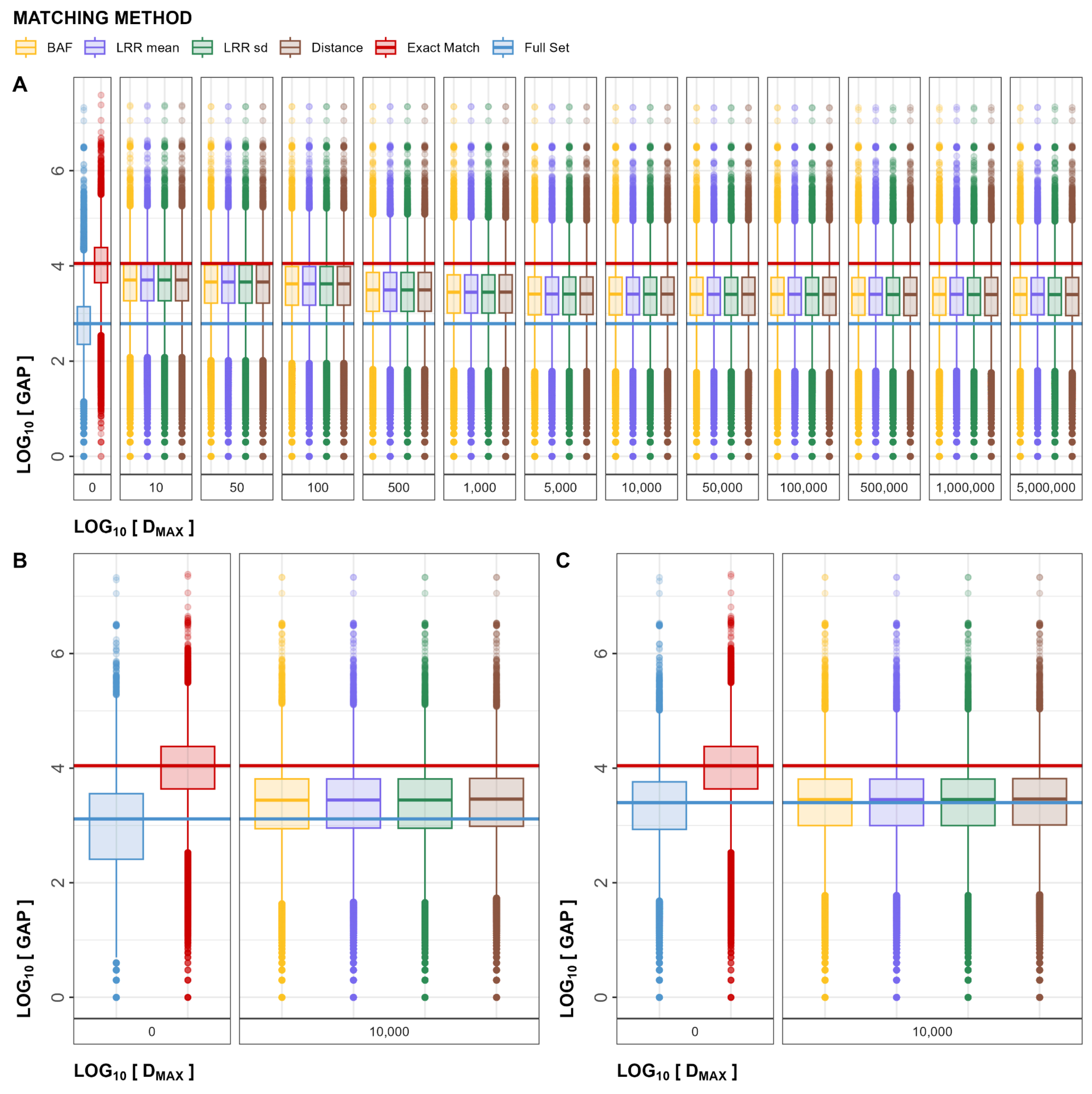

Figure S1. Inter-marker gaps in the Within-Array Experiment (A) and Cross-Array Experiment (B, C). Box plots of $\log_{10}$ transformations of the gap size between two adjacent markers are shown for full set (in blue), Exact Match (in red), and an array of MarkerMatch configurations (BAF based in yellow, LRR mean based in purple, LRR standard deviation based in green, and distance based in brown). X-axis shows Full Set and Exact Match for reference (with median lines spanning the entire graph), as well as different D_{MAX} parameters (ranging from 10bp to 5,000,000bp). Gaps are shown for OMNI array (A), OEE array (B), and GSA1 array (C). OMNI: Omni2.5 array, GSA1: Global Screening Array, OEE: Omni Express Exome array.

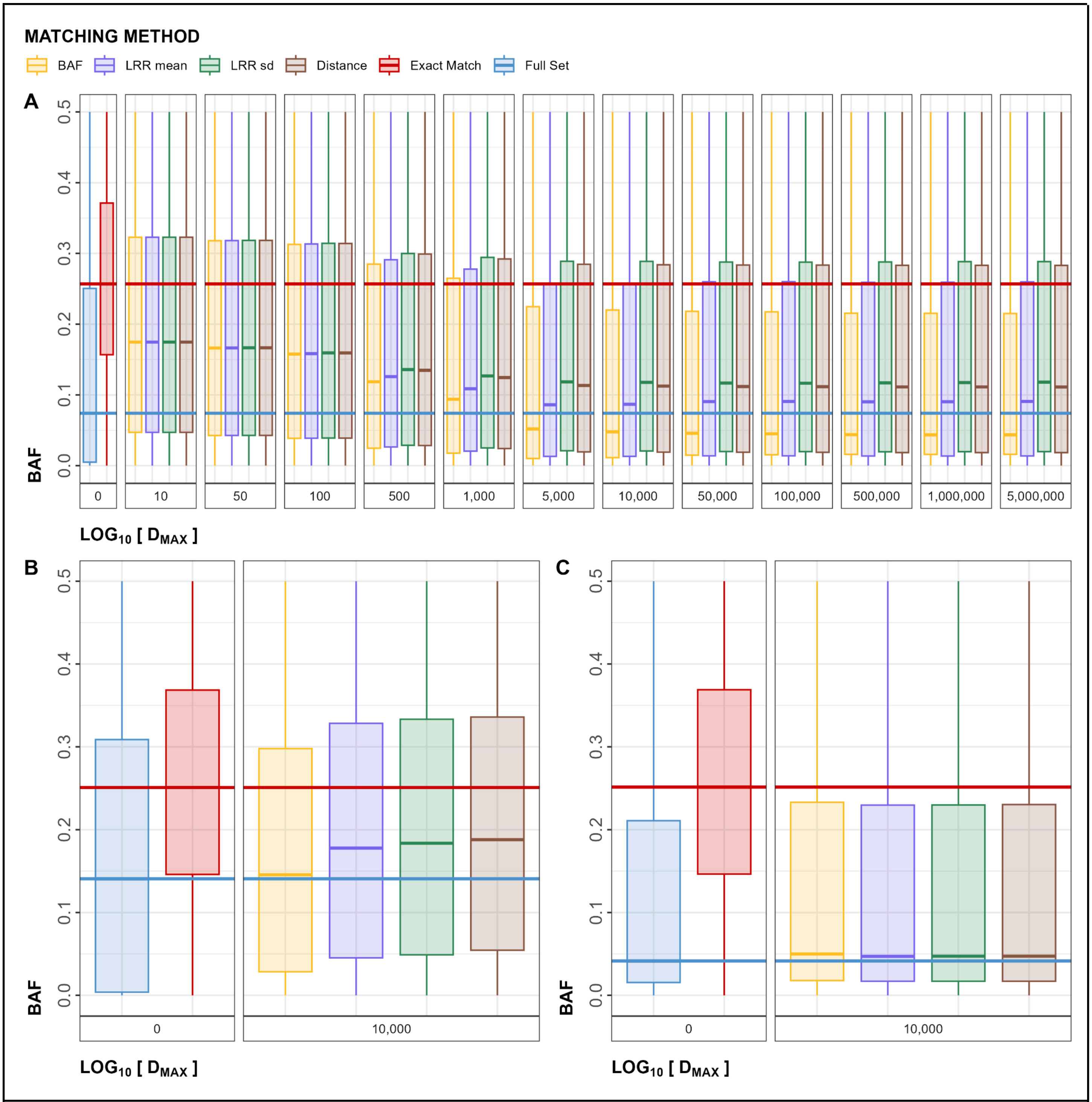

Figure S2. BAF metrics in the Within-Array Experiment (A) and Cross-Array Experiment (B, C). Box plots of marker-wise BAF are shown for Full Set (in blue), Exact Match (in red), and an array of MarkerMatch configurations (BAF based in yellow, LRR mean based in purple, LRR standard deviation based in green, and distance based in brown). X-axis shows Full Set and Exact Match for reference (with median lines spanning the entire graph), as well as different D_{MAX} parameters (ranging from 10bp to 5,000,000bp). BAFs are shown for OMNI array (A), OEE array (B), and GSA1 array (C). OMNI: Omni2.5 array, GSA1: Global Screening Array, OEE: Omni Express Exome array.

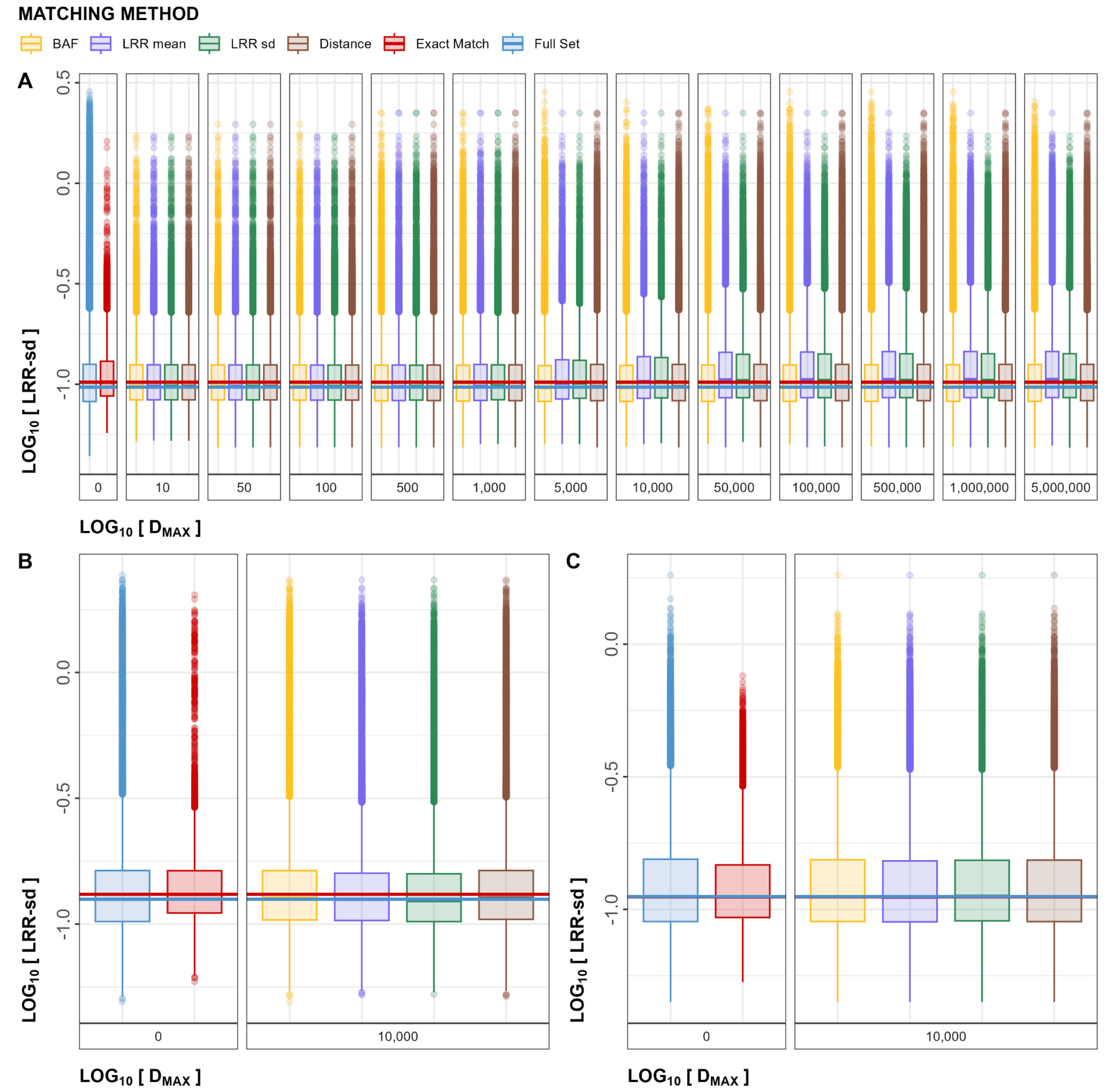

Figure S3. LRR sd metrics in the Within-Array Experiment (A) and Cross-Array Experiment (B, C). Box plots of log_{10} transformations of marker-wise LRR sds are shown for full set (in blue), Exact match (in red), and an array of MarkerMatch configurations (BAF based in yellow, LRR mean based in purple, LRR standard deviation based in green, and distance based in brown). X-axis shows Full Set and Exact Match for reference (with median lines spanning the entire graph), as well as different D_{MAX} parameters (ranging from 10bp to 5,000,000bp). LRR sds are shown for OMNI array (A), OEE array (B), and GSA1 array (C). OMNI: Omni2.5 array, GSA1: Global Screening Array, OEE: Omni Express Exome array.

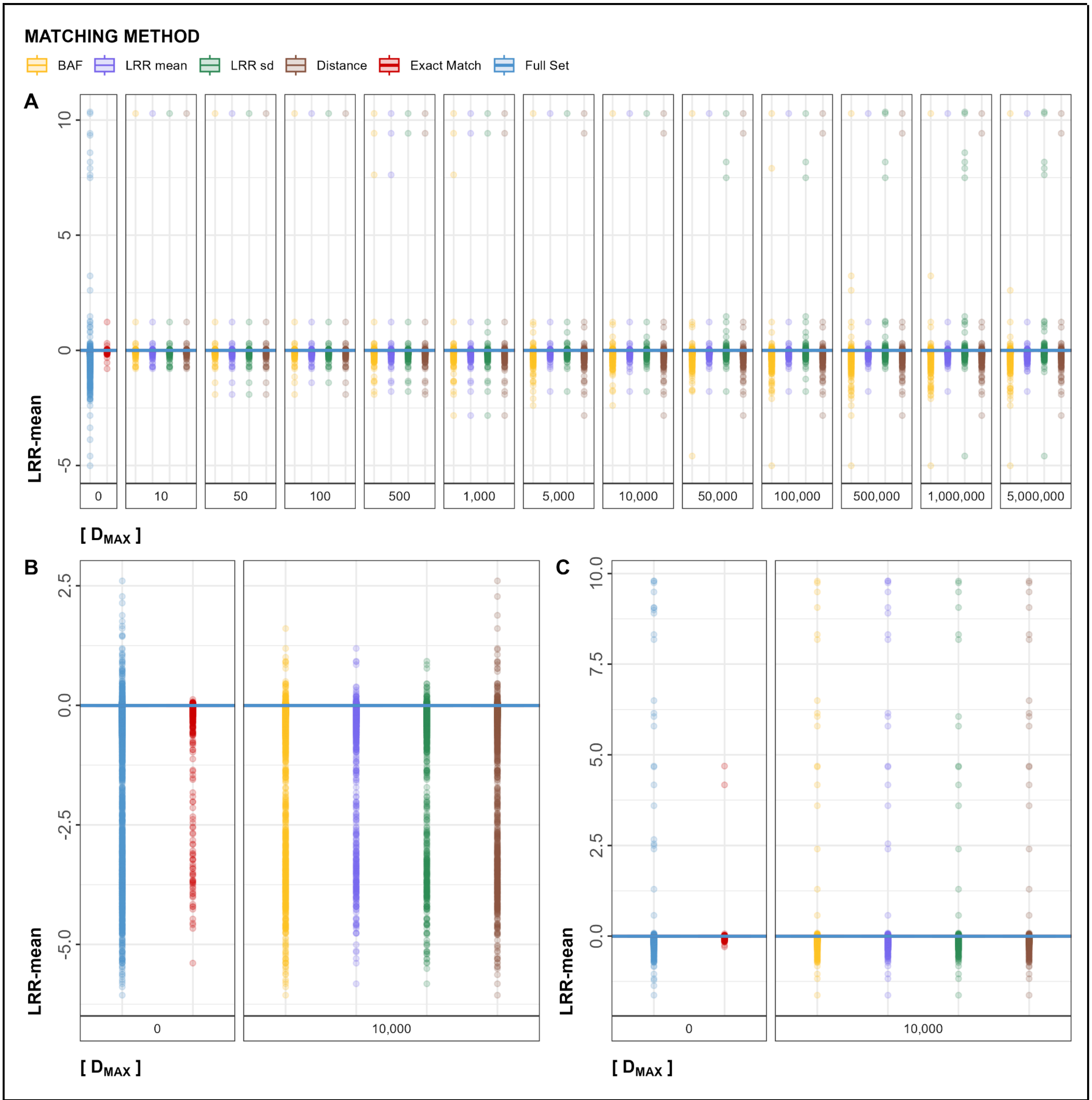

Figure S4. LRR mean metrics in the Within-Array Experiment (A) and Cross-Array Experiment (B, C). Box plots of marker-wise LRR means are shown for full set (in blue), Exact match (in red), and an array of MarkerMatch configurations (BAF based in yellow, LRR mean based in purple, LRR standard deviation based in green, and distance based in brown). X-axis shows Full Set and Exact Match for reference (with median lines spanning the entire graph), as well as different D_{MAX} parameters (ranging from 10bp to 5,000,000bp). LRR means are shown for OMNI array (A), OEE array (B), and GSA1 array (C). OMNI: Omni2.5 array, GSA1: Global Screening Array, OEE: Omni Express Exome array.

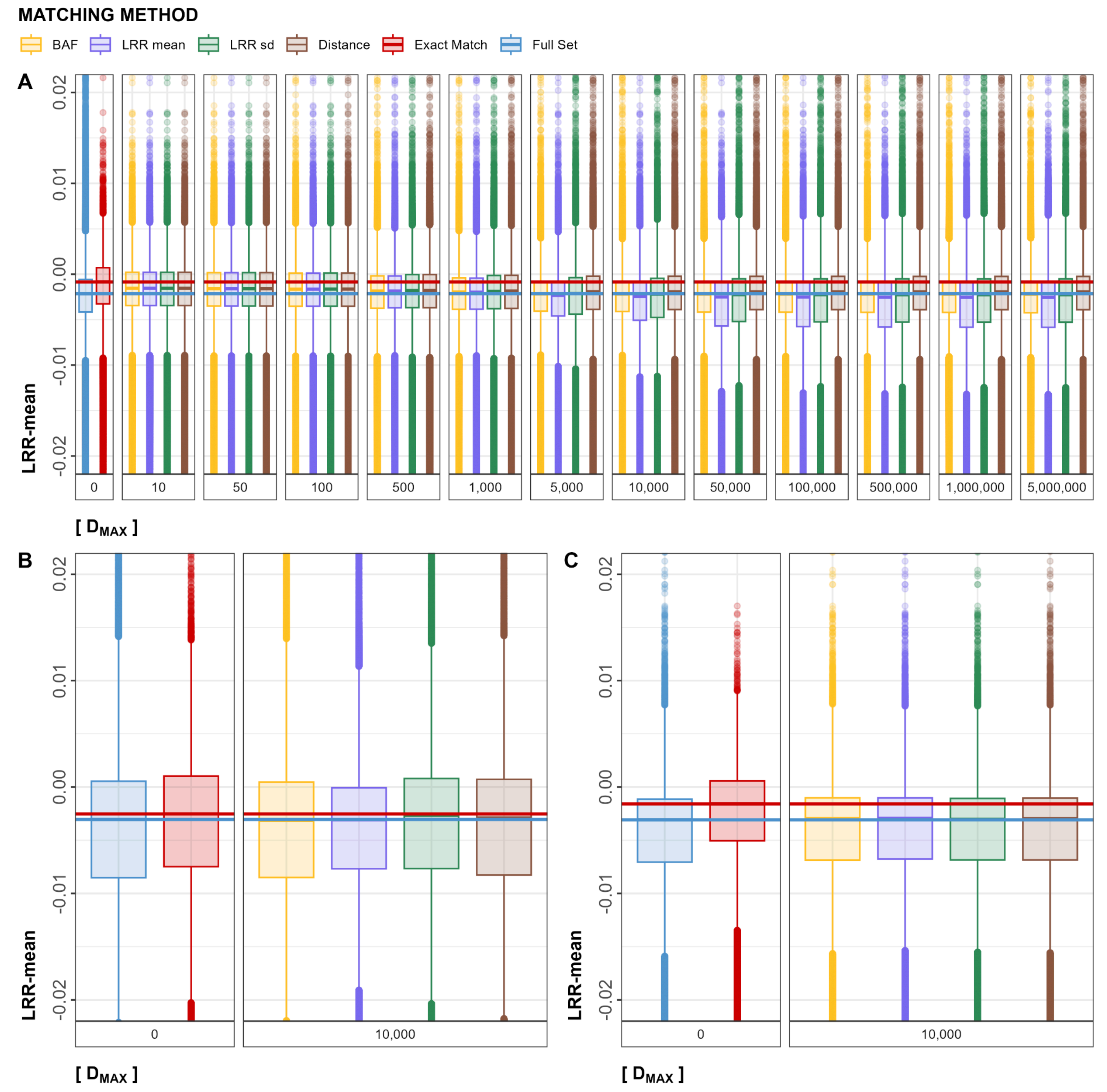

Figure S5. LRR mean metrics in the Within-Array Experiment (A) and Cross-Array Experiment (B, C). Box plots of marker-wise LRR means are shown for full set (in blue), Exact match (in red), and an array of MarkerMatch configurations (BAF based in yellow, LRR mean based in purple, LRR standard deviation based in green, and distance based in brown). X-axis shows Full Set and Exact Match for reference (with median lines spanning the entire graph), as well as different D_{MAX} parameters (ranging from 10bp to 5,000,000bp). LRR means are shown for OMNI array (A), OEE array (B), and GSA1 array (C). The plots are zoomed to LRR mean range -0.02 to 0.02. OMNI: Omni2.5 array, GSA1: Global Screening Array, OEE: Omni Express Exome array.

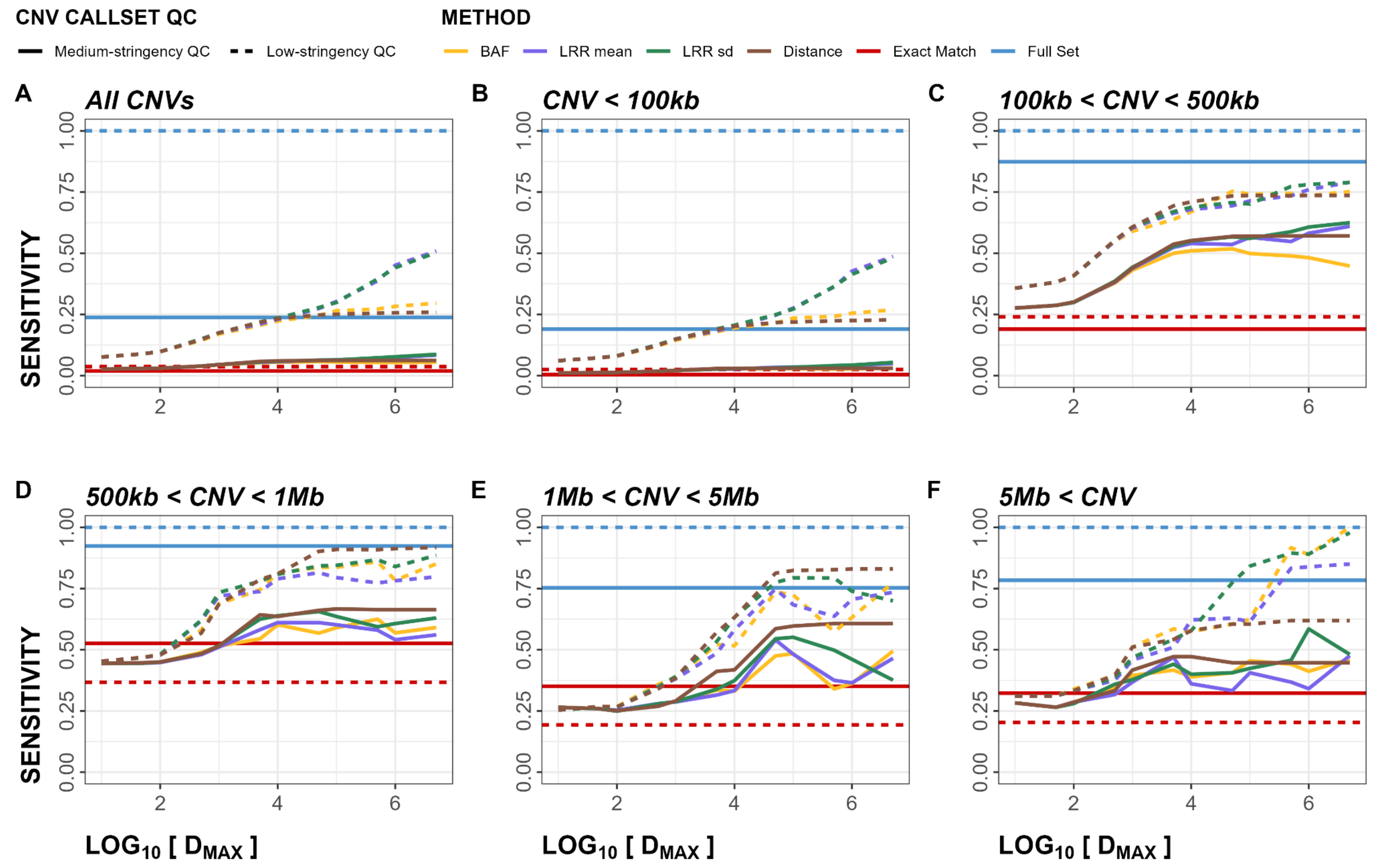

Figure S6. Within-Array Experiment sensitivity plots for both deletions and duplications. Panels A-F show sensitivity metrics stratified by CNV size. Dashed line represents low-stringency QC, solid line represents medium-stringency QC.

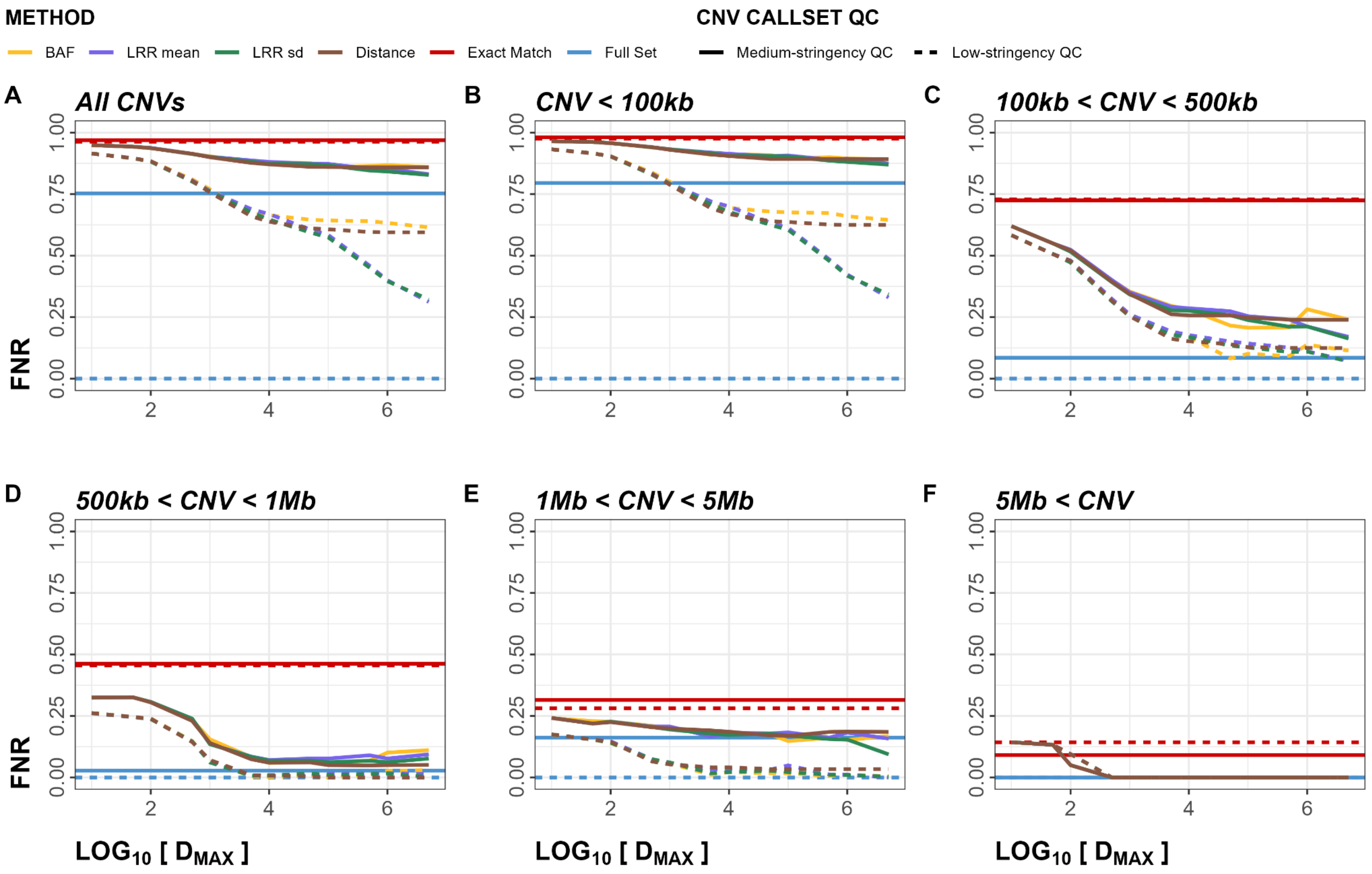

Figure S7. Within-Array Experiment false negative rate (FNR) plots for both deletions and duplications. Panels A-F show FNR metrics stratified by CNV size. Dashed line represents low-stringency QC, solid line represents medium-stringency QC.

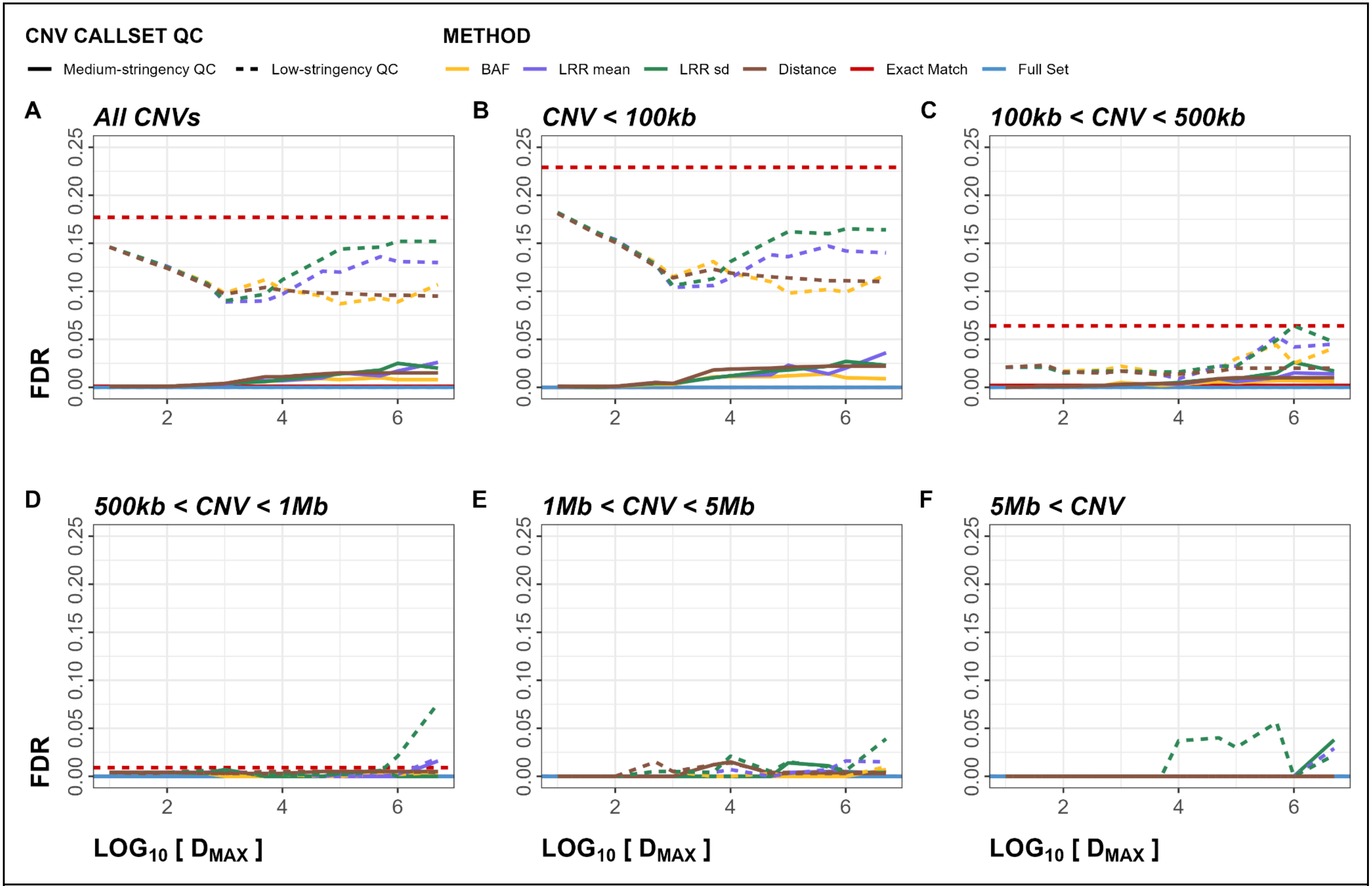

Figure S8. Within-Array Experiment false discovery rate (FDR) plots for both deletions and duplications. Panels A-F show FDR metrics stratified by CNV size. Dashed line represents low-stringency QC, solid line represents medium-stringency QC..

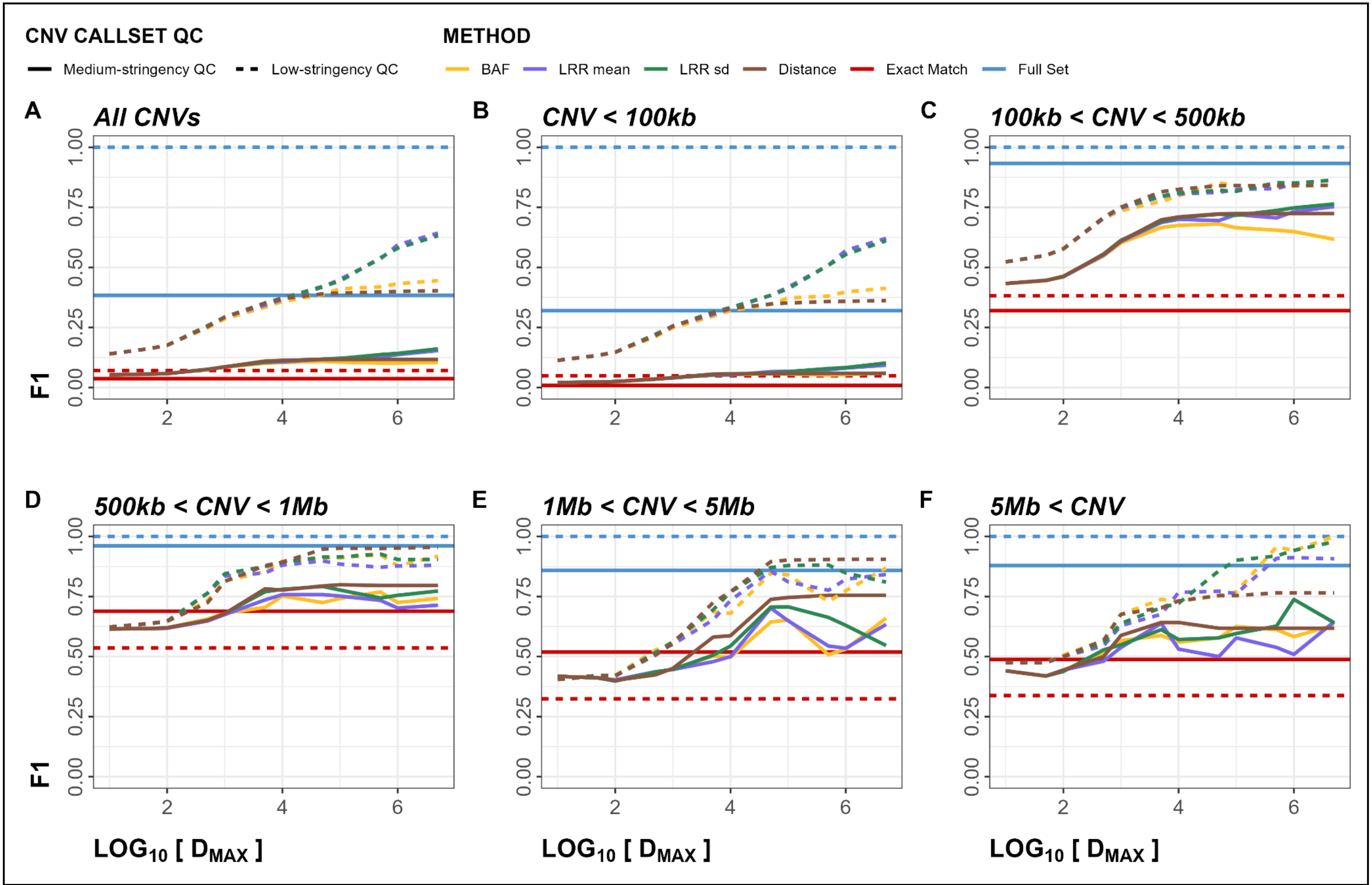

Figure S9. Within-Array Experiment F1 score plots for both deletions and duplications. Panels A-F show F1 metrics stratified by CNV size. Dashed line represents low-stringency QC, solid line represents medium-stringency QC.

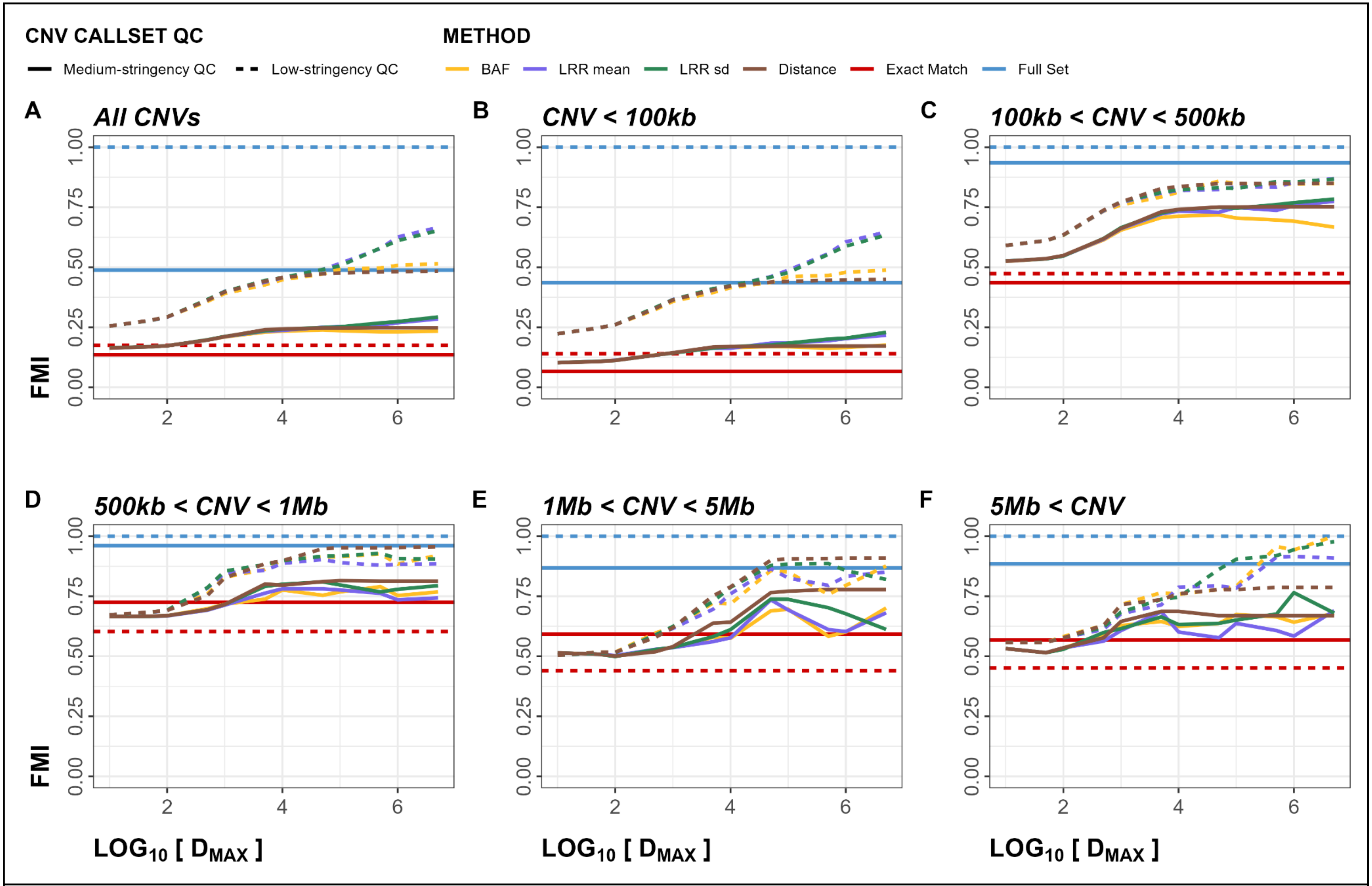

Figure S10. Within-Array Experiment Fowlkes-Mallows index (FMI) plots for both deletions and duplications. Panels A-F show FMI metrics stratified by CNV size. Dashed line represents low-stringency QC, solid line represents medium-stringency QC.

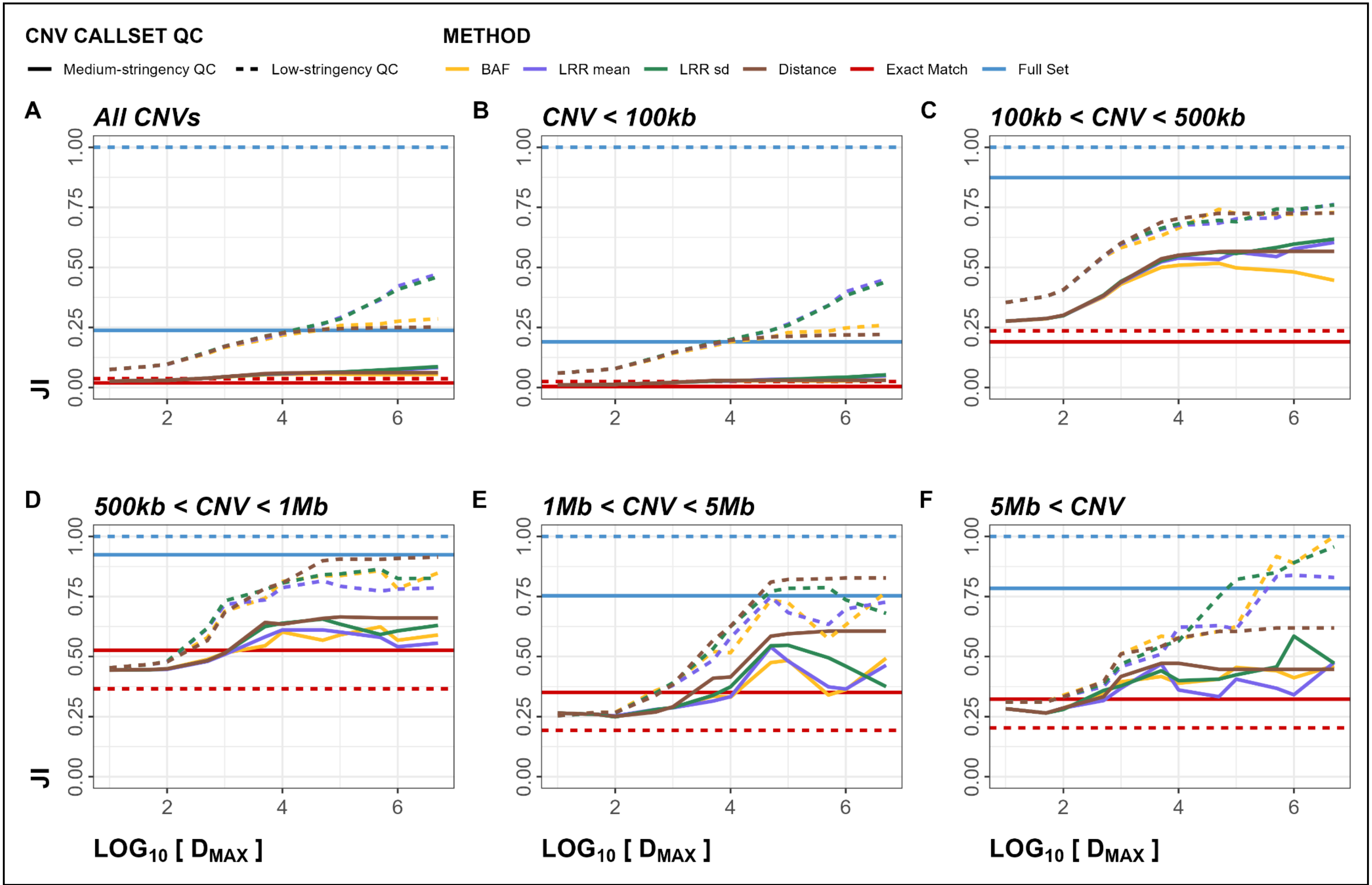

Figure S11. Within-Array Experiment Jaccard index (JI) plots for both deletions and duplications. Panels A-F show JI metrics stratified by CNV size. Dashed line represents low-stringency QC, solid line represents medium-stringency QC.

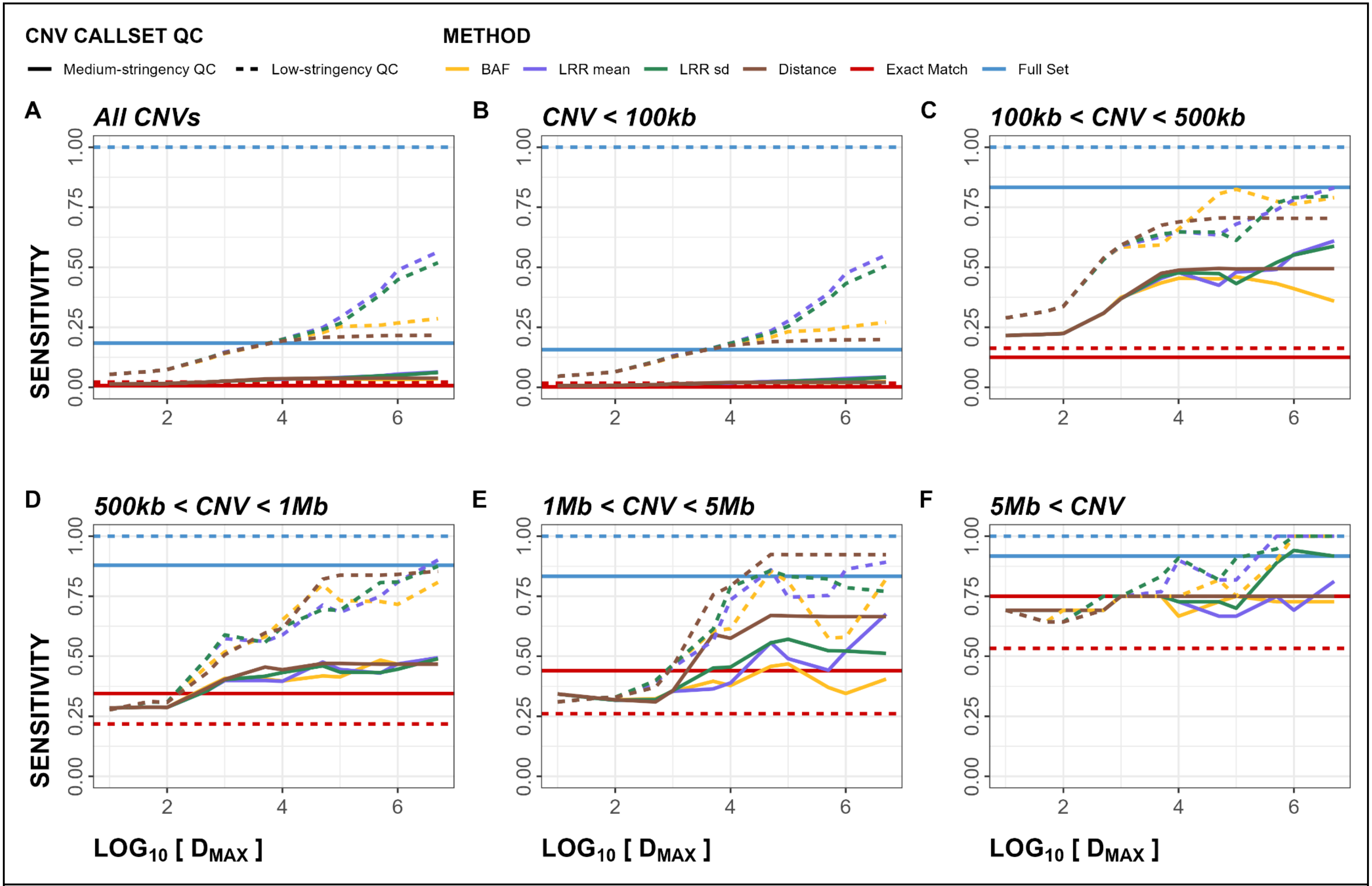

Figure S12. Within-Array Experiment sensitivity plots for deletions. Panels A-F show sensitivity metrics stratified by CNV size. Dashed line represents low-stringency QC, solid line represents medium-stringency QC.

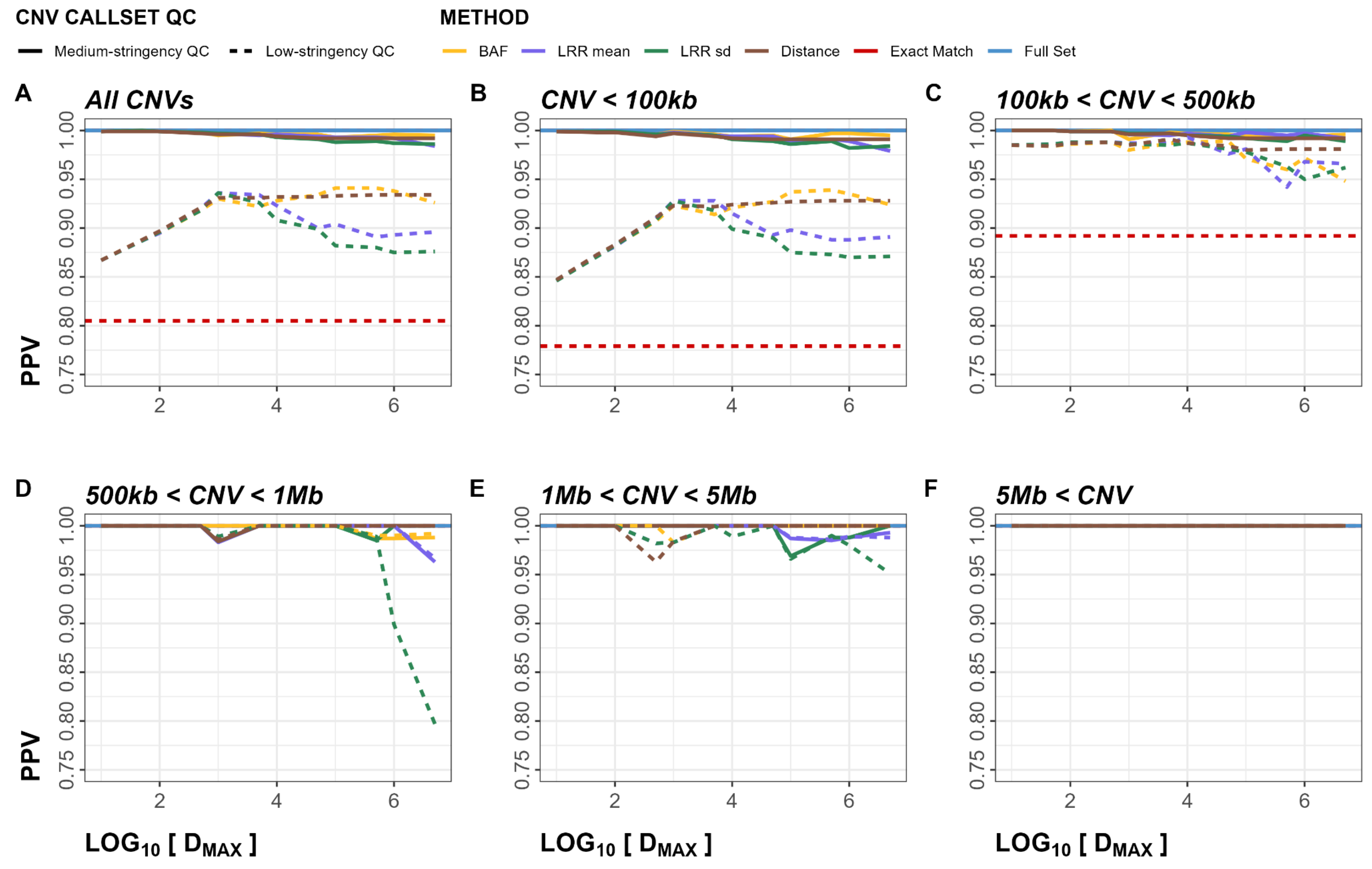

Figure S13. Within-Array Experiment positive predictive values (PPV) plots for deletions. Panels A-F show PPV metrics stratified by CNV size. Dashed line represents low-stringency QC, solid line represents medium-stringency QC.

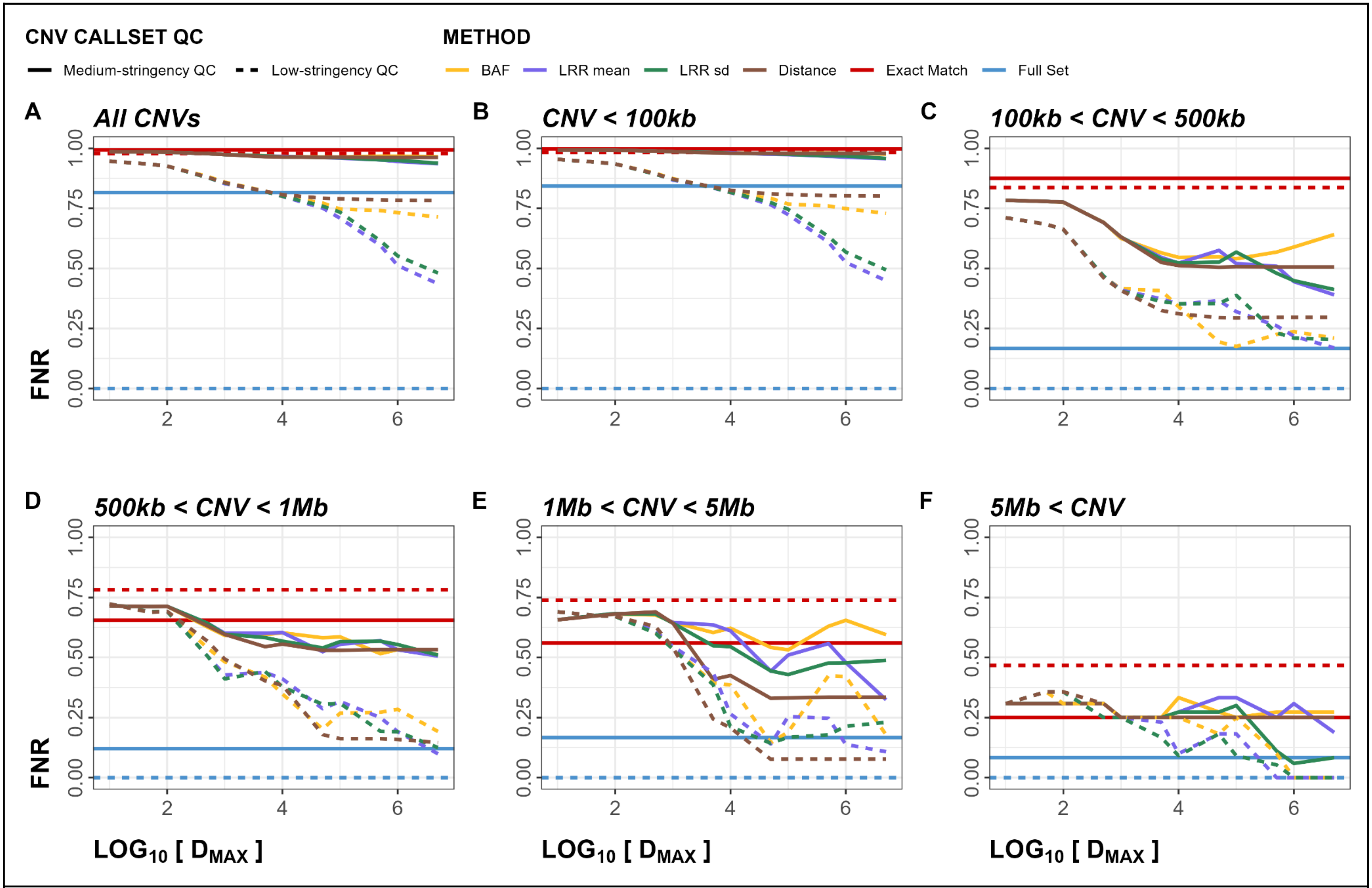

Figure S14. Within-Array Experiment false negative rate (FNR) plots for deletions. Panels A-F show FNR metrics stratified by CNV size. Dashed line represents low-stringency QC, solid line represents medium-stringency QC.

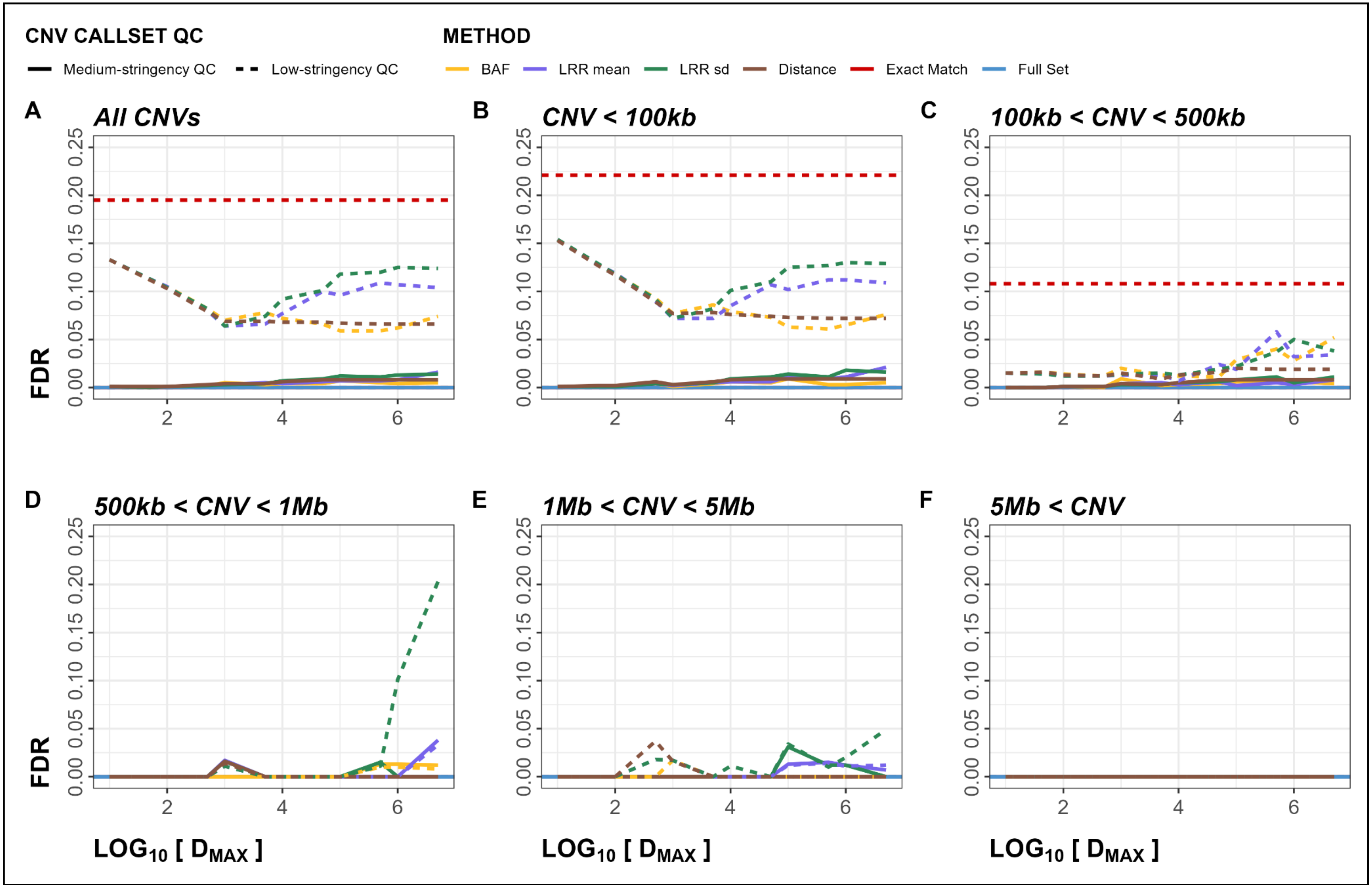

Figure S15. Within-Array Experiment false discovery rate (FDR) plots for deletions. Panels A-F show FDR metrics stratified by CNV size. Dashed line represents low-stringency QC, solid line represents medium-stringency QC.

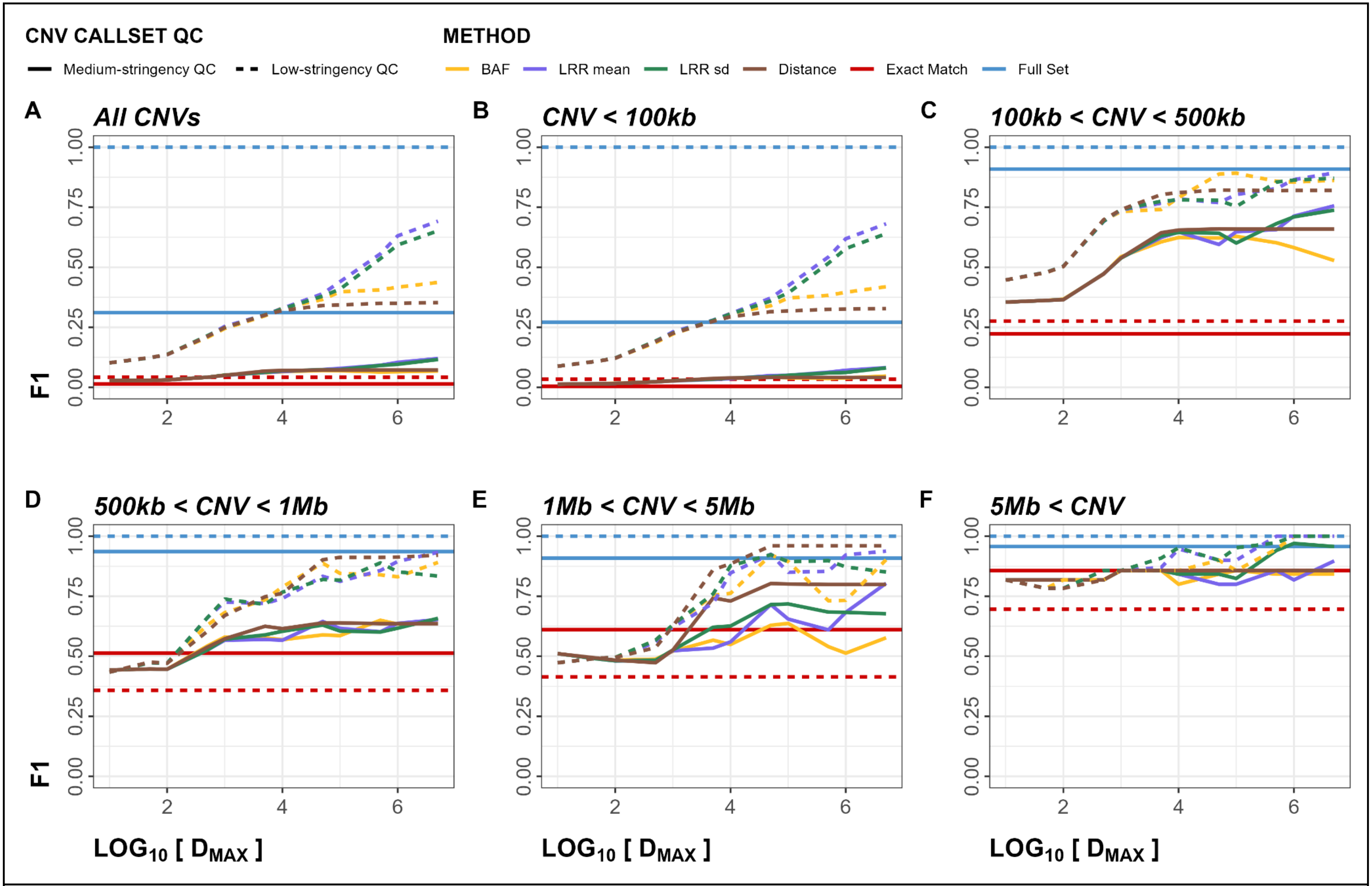

Figure S16. Within-Array Experiment F1 score plots for deletions. Panels A-F show F1 metrics stratified by CNV size. Dashed line represents low-stringency QC, solid line represents medium-stringency QC.

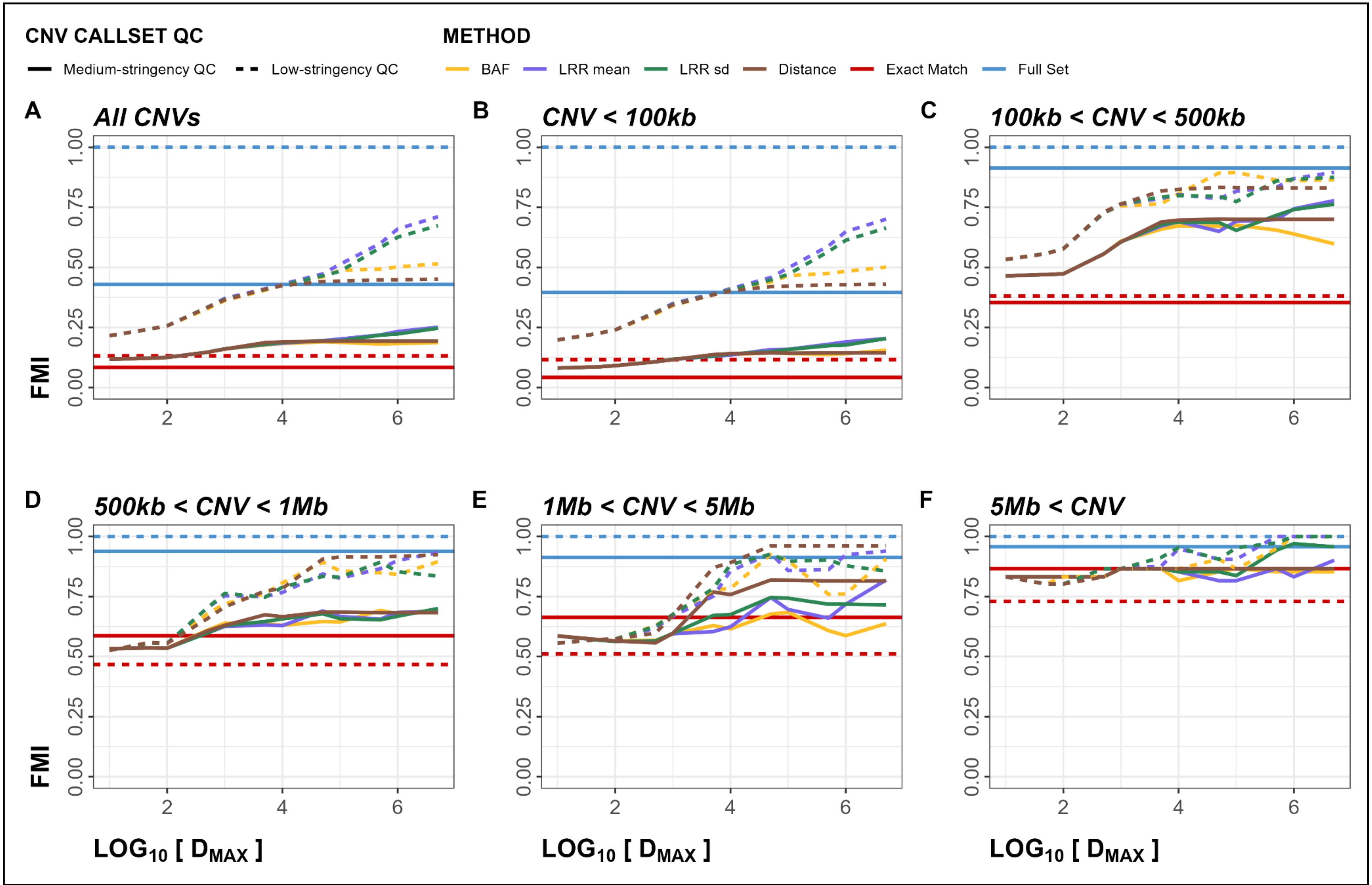

Figure S17. Within-Array Experiment Fowlkes-Mallows index (FMI) plots for deletions. Panels A-F show FMI metrics stratified by CNV size. Dashed line represents low-stringency QC, solid line represents medium-stringency QC.

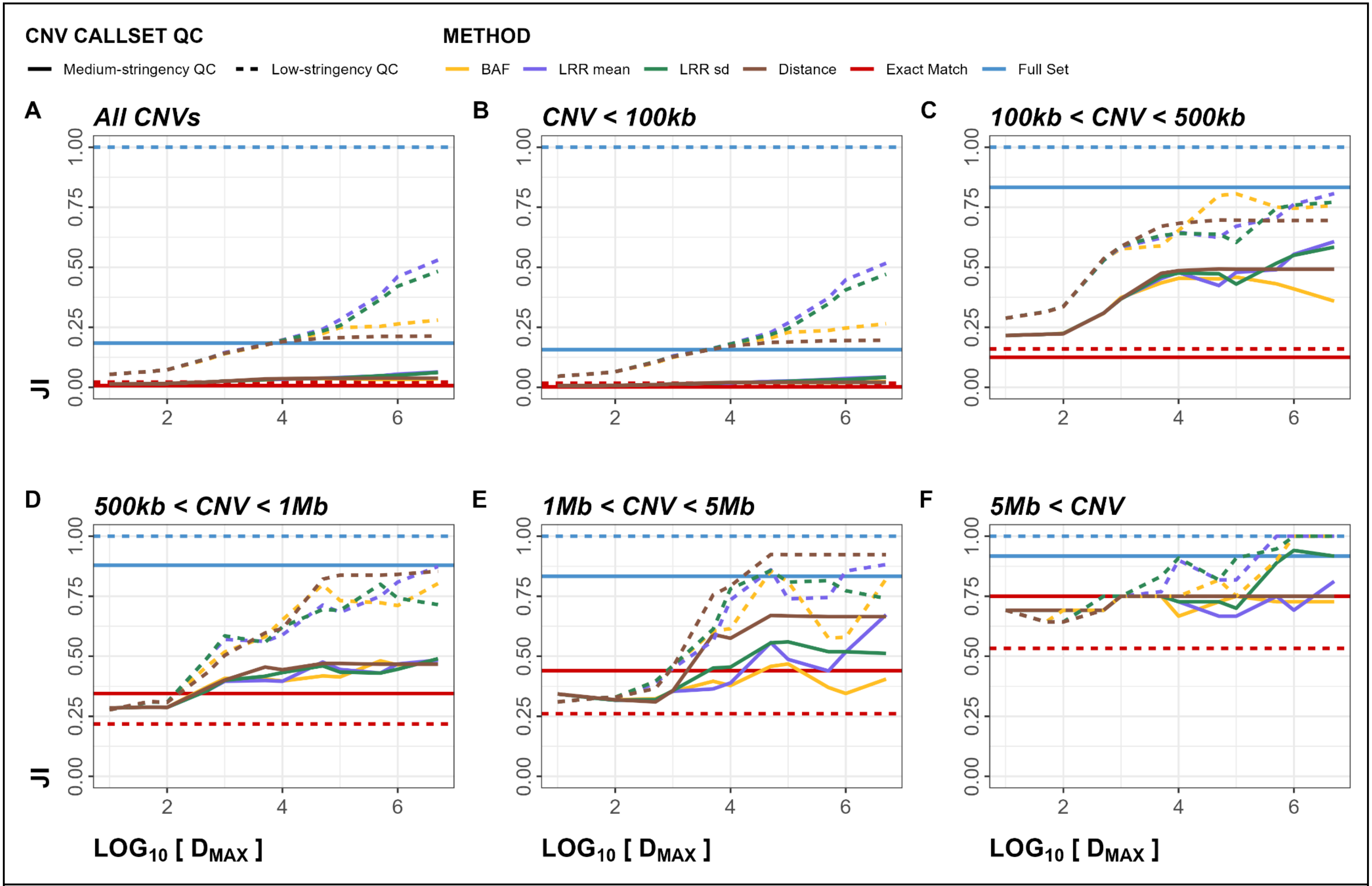

Figure S18. Within-Array Experiment Jaccard index (JI) plots for deletions. Panels A-F show JI metrics stratified by CNV size. Dashed line represents low-stringency QC, solid line represents medium-stringency QC.

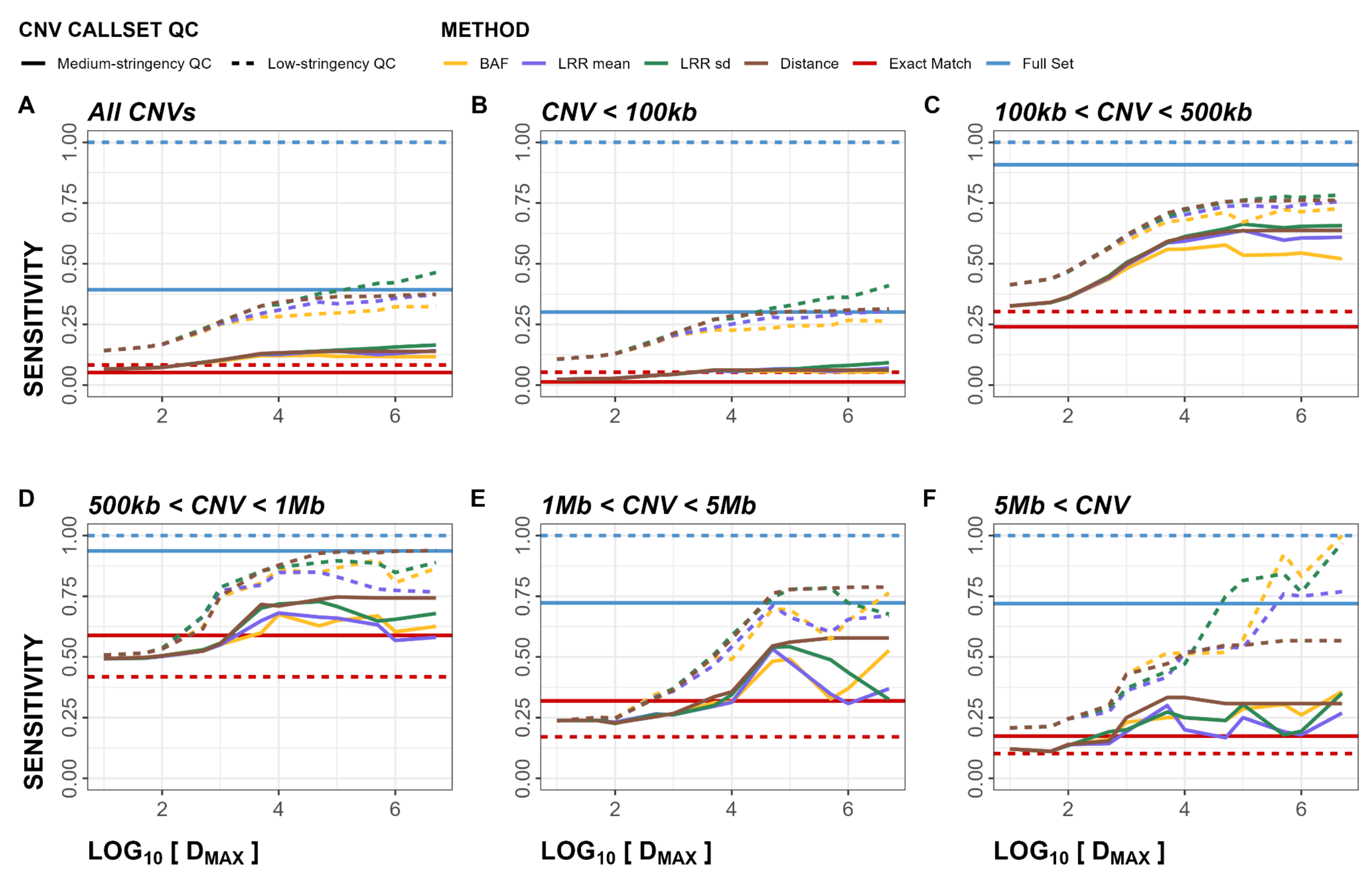

Figure S19. Within-Array Experiment sensitivity plots for duplications. Panels A-F show sensitivity metrics stratified by CNV size. Dashed line represents low-stringency QC, solid line represents medium-stringency QC.

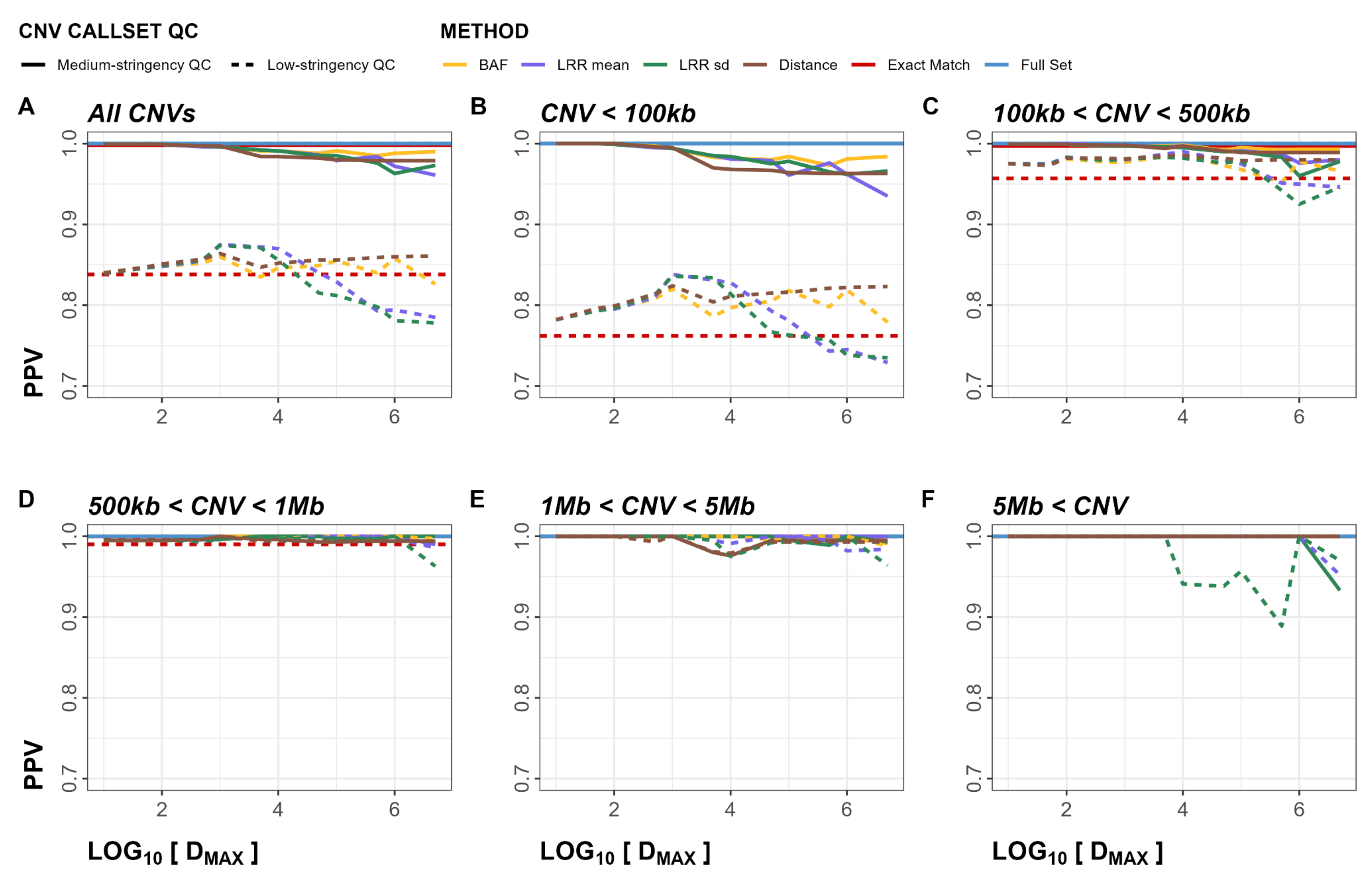

Figure S20. Within-Array Experiment positive predictive values (PPV) plots for duplications. Panels A-F show PPV metrics stratified by CNV size. Dashed line represents low-stringency QC, solid line represents medium-stringency QC.

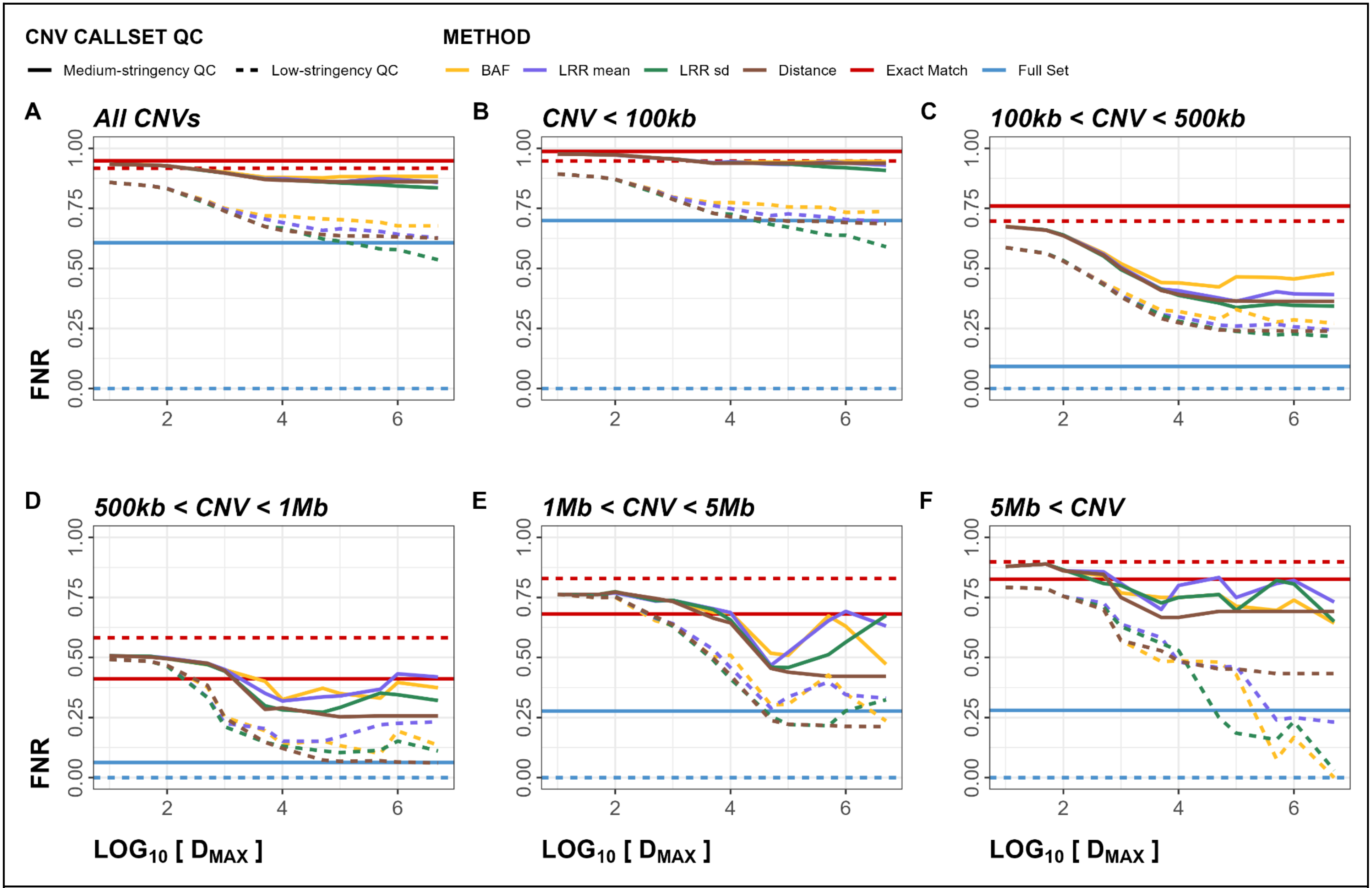

Figure S21. Within-Array Experiment false negative rate (FNR) plots for duplications. Panels A-F show FNR metrics stratified by CNV size. Dashed line represents low-stringency QC, solid line represents medium-stringency QC.

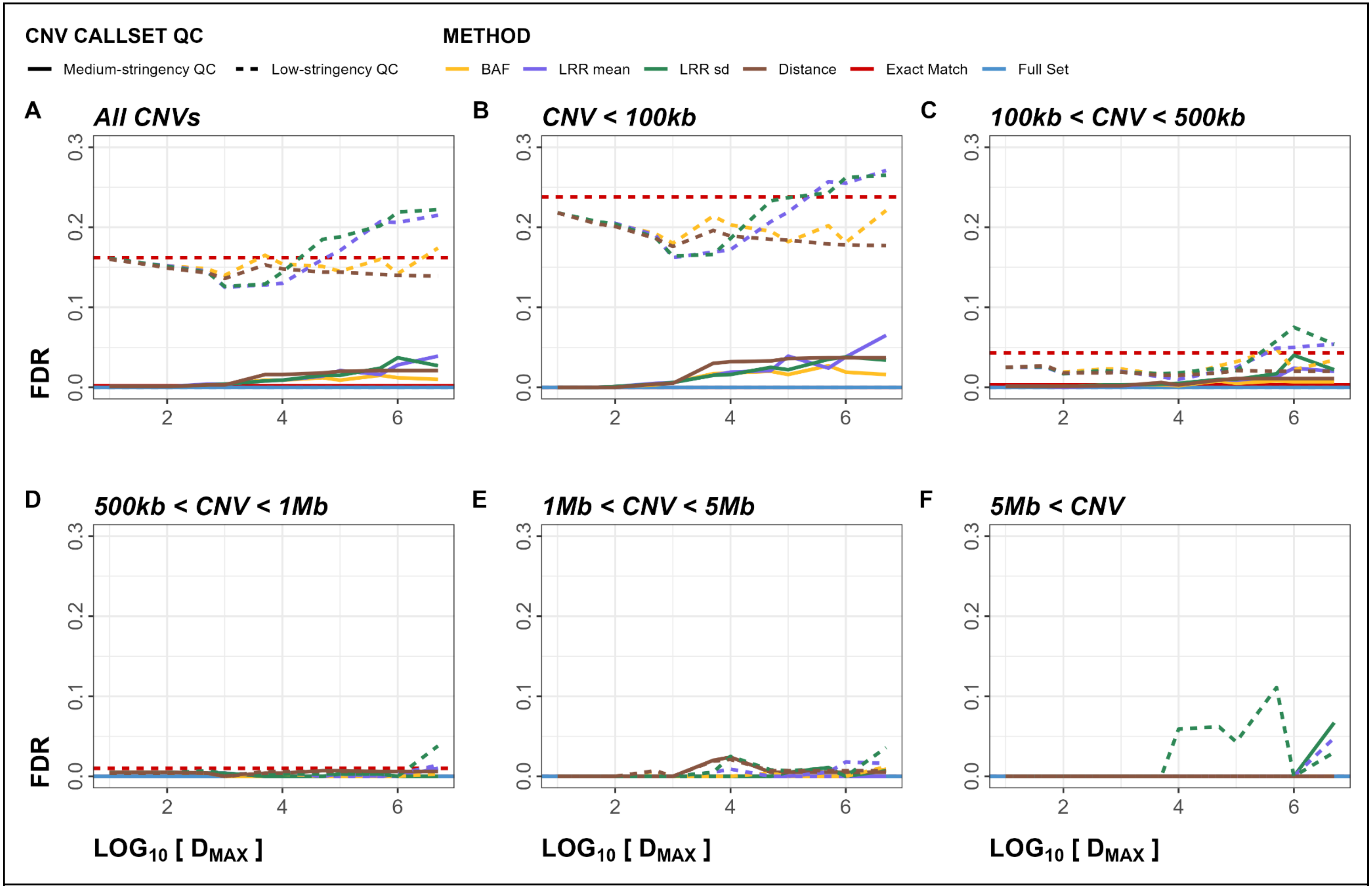

Figure S22. Within-Array Experiment false discovery rate (FDR) plots for duplications. Panels A-F show FDR metrics stratified by CNV size. Dashed line represents low-stringency QC, solid line represents medium-stringency QC.

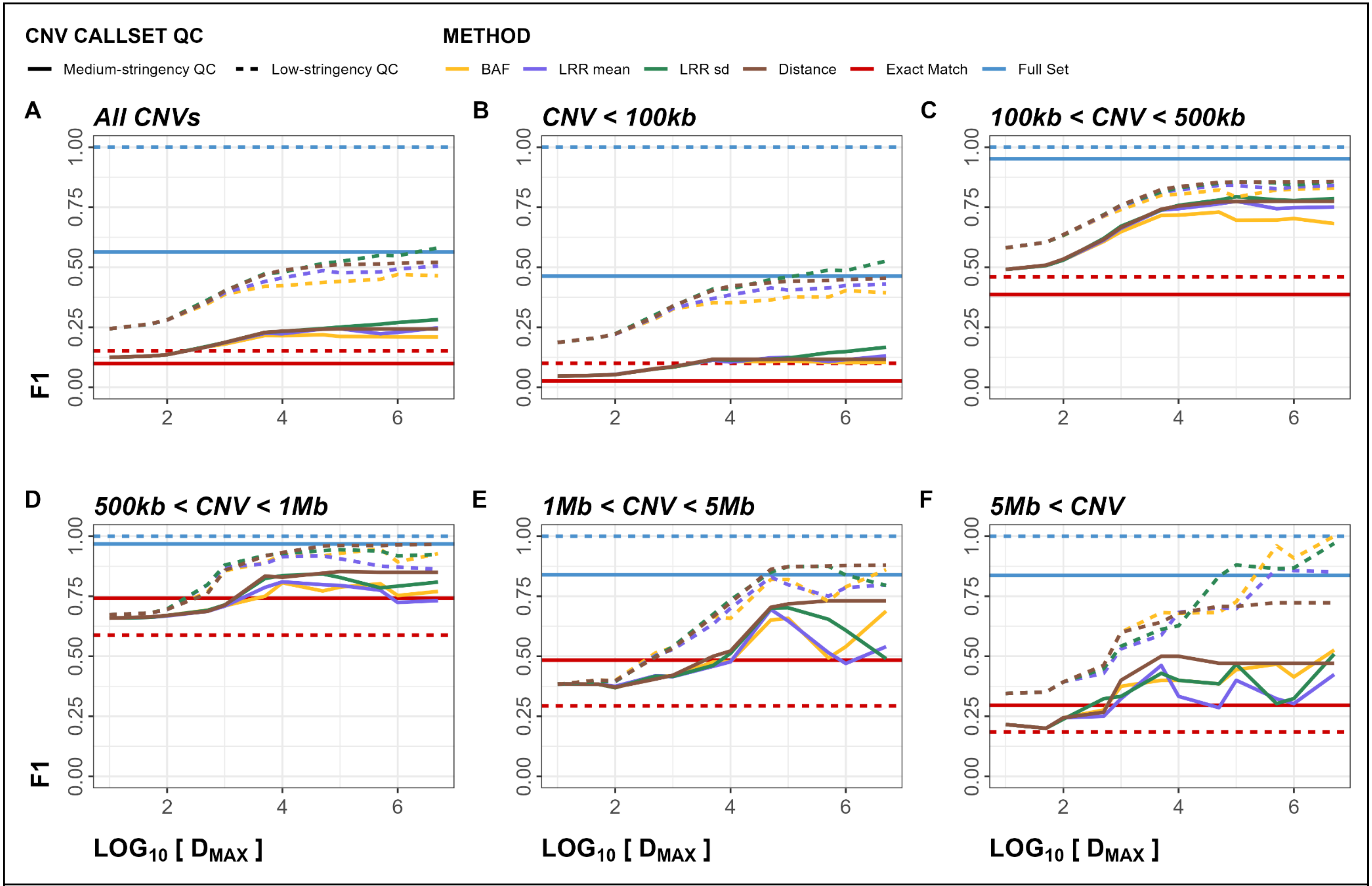

Figure S23. Within-Array Experiment F1 score plots for duplications. Panels A-F show F1 metrics stratified by CNV size. Dashed line represents low-stringency QC, solid line represents medium-stringency QC.

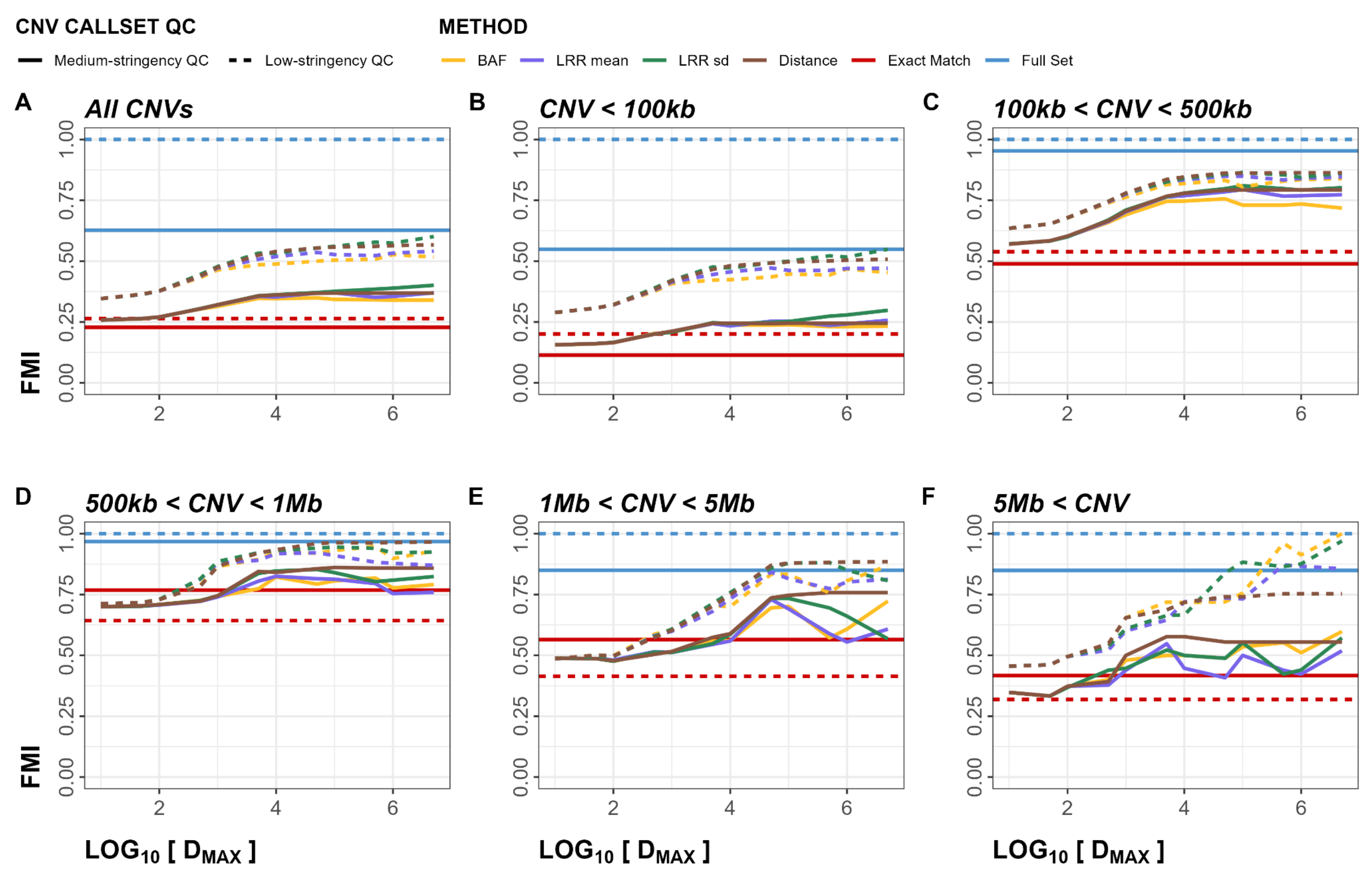

Figure S24. Within-Array Experiment Fowlkes-Mallows index (FMI) plots for duplications. Panels A-F show FMI metrics stratified by CNV size. Dashed line represents low-stringency QC, solid line represents medium-stringency QC.

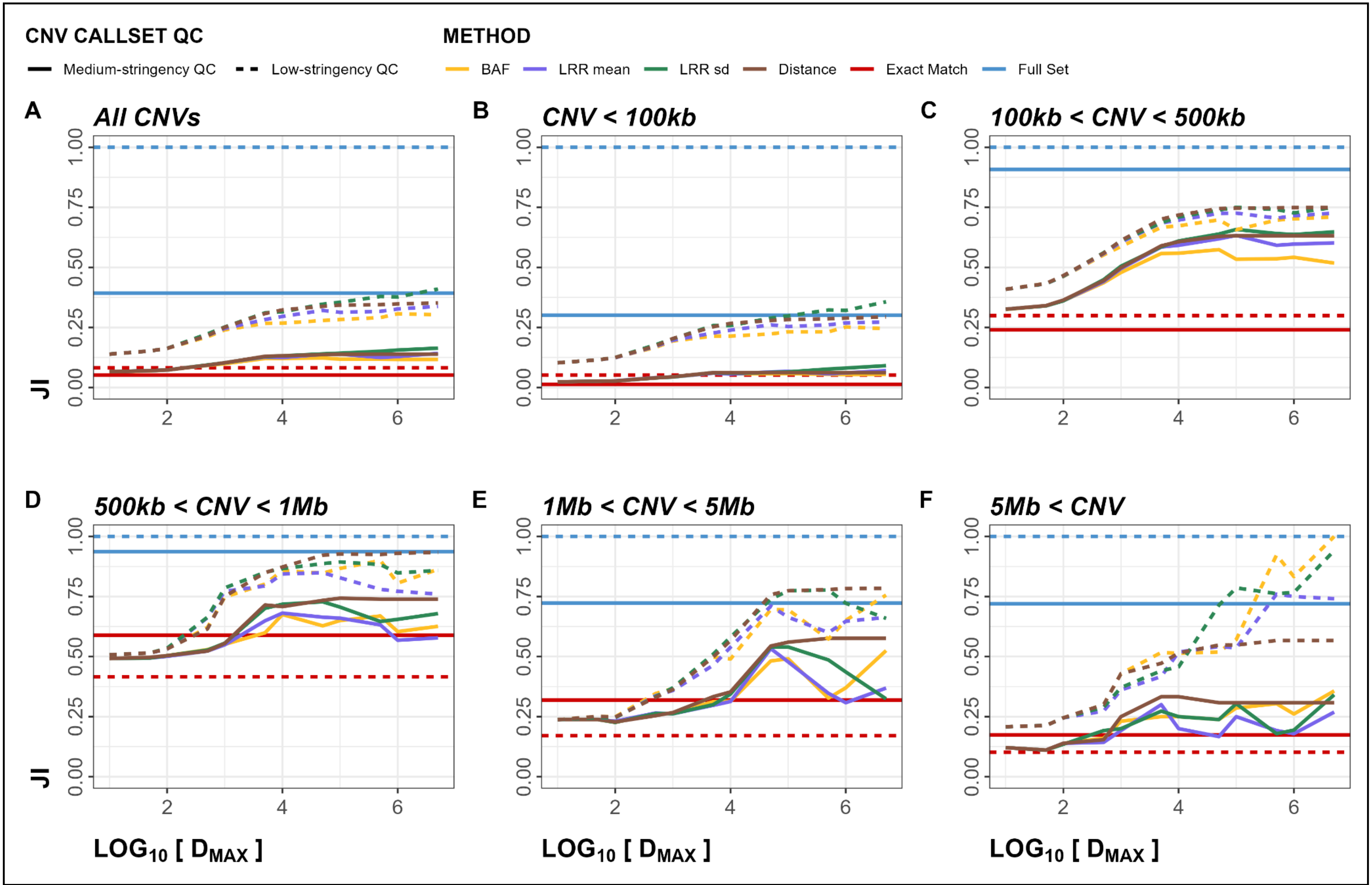

Figure S25. Within-Array Experiment Jaccard index (JI) plots for duplications. Panels A-F show JI metrics stratified by CNV size. Dashed line represents low-stringency QC, solid line represents medium-stringency QC.

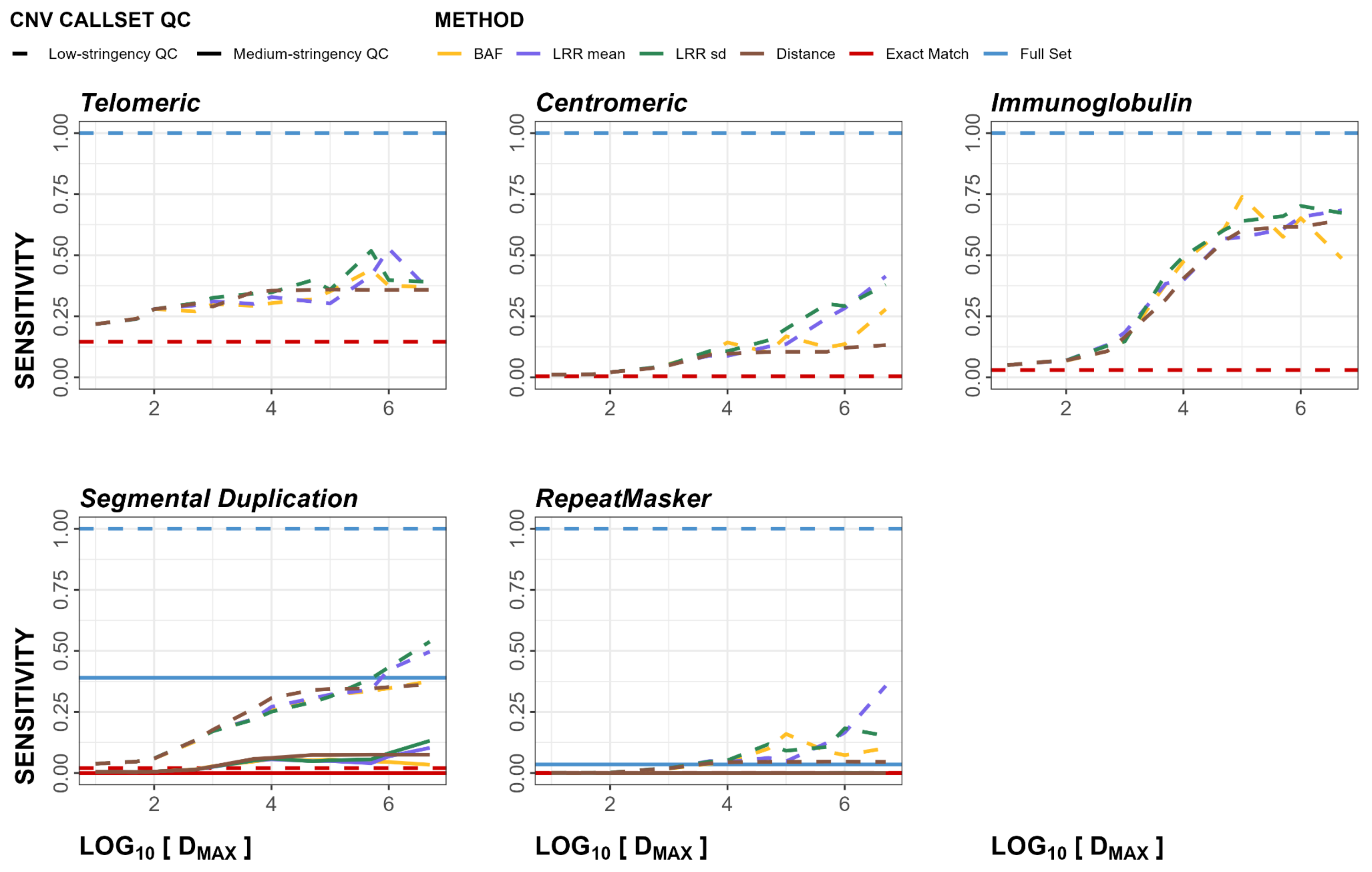

Figure S26. Within-Array Experiment sensitivity regional plots for both deletions and duplications. Panels show telomeric, centromeric, immunoglobulin, segmental duplication, and repeat masker (simple repeat) regions in order, left to right. Dashed line represents low-stringency QC, solid line represents medium stringency QC.

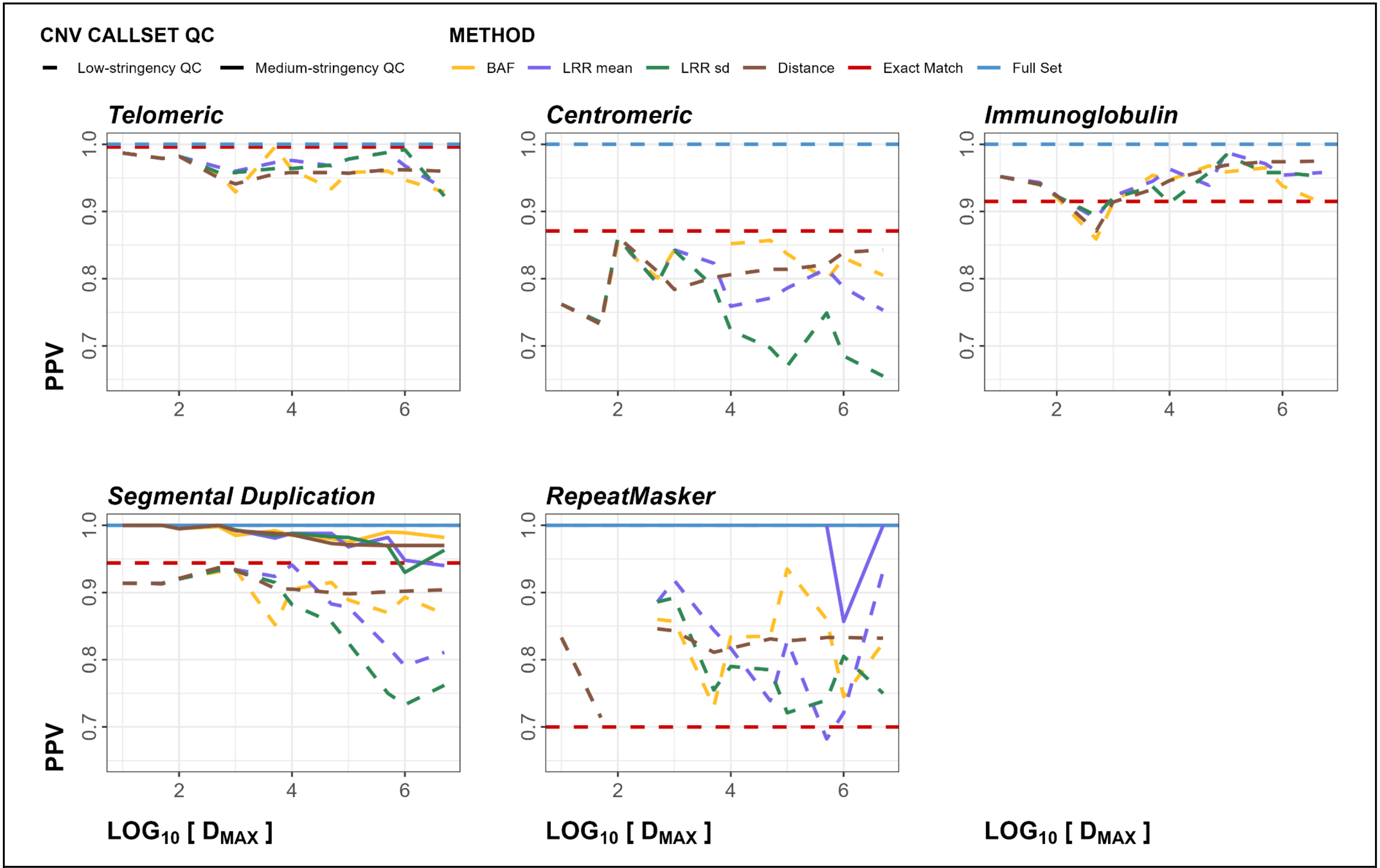

Figure S27. Within-Array Experiment PPV regional plots for both deletions and duplications. Panels show telomeric, centromeric, immunoglobulin, segmental duplication, and repeat masker (simple repeat) regions in order, left to right. Dashed line represents low-stringency QC, solid line represents medium stringency QC.

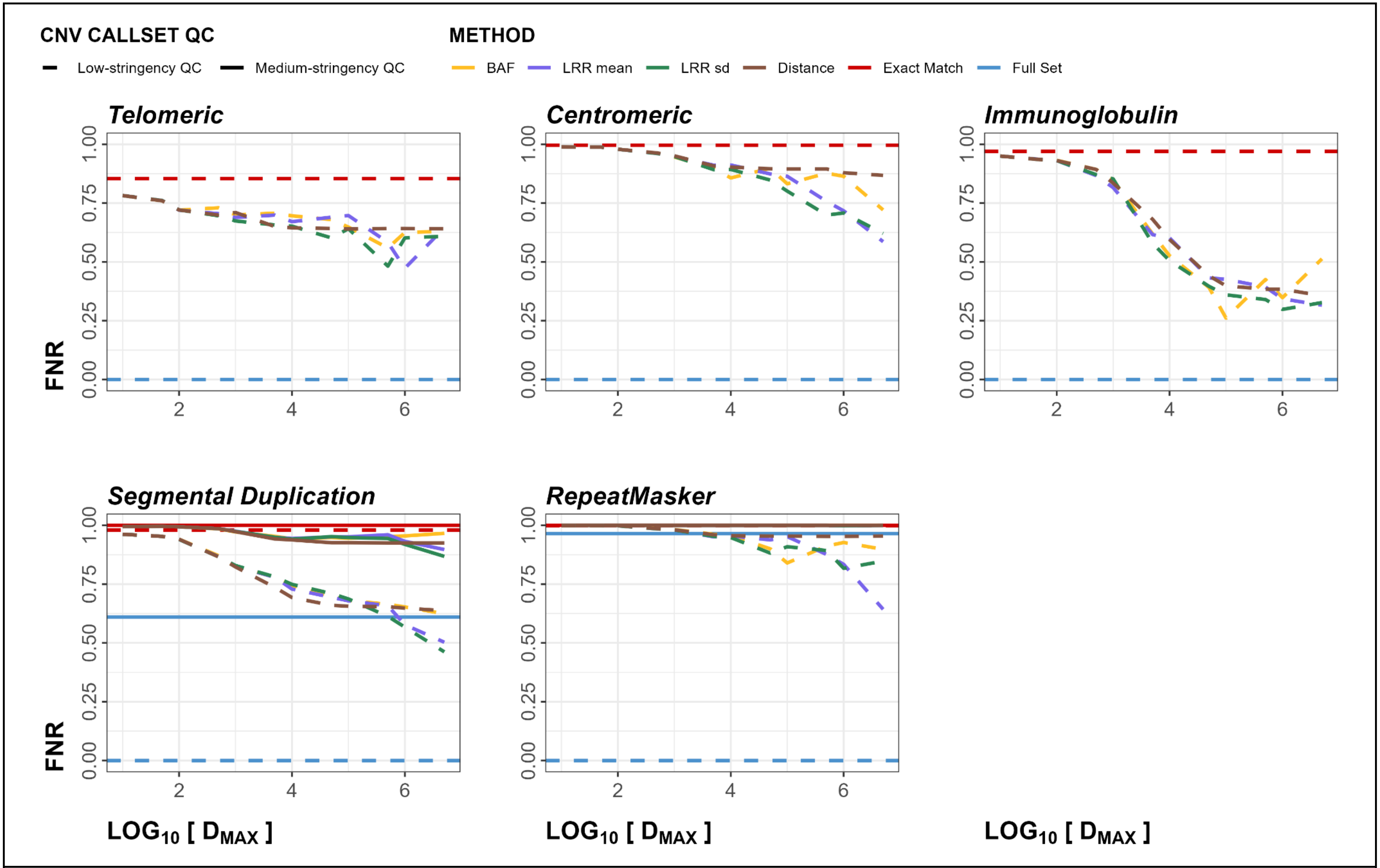

Figure S28. Within-Array Experiment FNR regional plots for both deletions and duplications. Panels show telomeric, centromeric, immunoglobulin, segmental duplication, and repeat masker (simple repeat) regions in order, left to right. Dashed line represents low-stringency QC, solid line represents medium stringency QC.

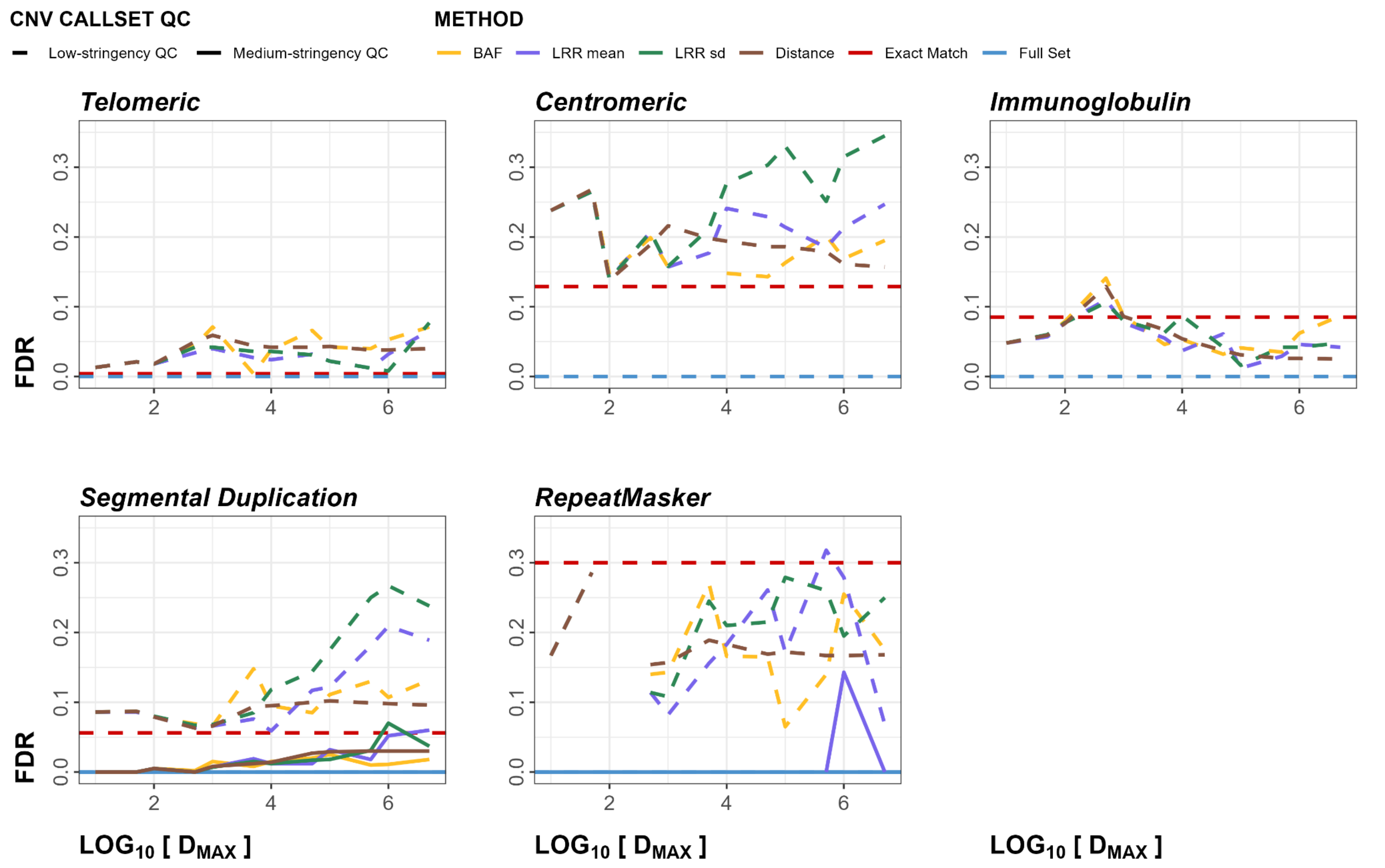

Figure S29. Within-Array Experiment FDR regional plots for both deletions and duplications. Panels show telomeric, centromeric, immunoglobulin, segmental duplication, and repeat masker (simple repeat) regions in order, left to right. Dashed line represents low-stringency QC, solid line represents medium stringency QC.

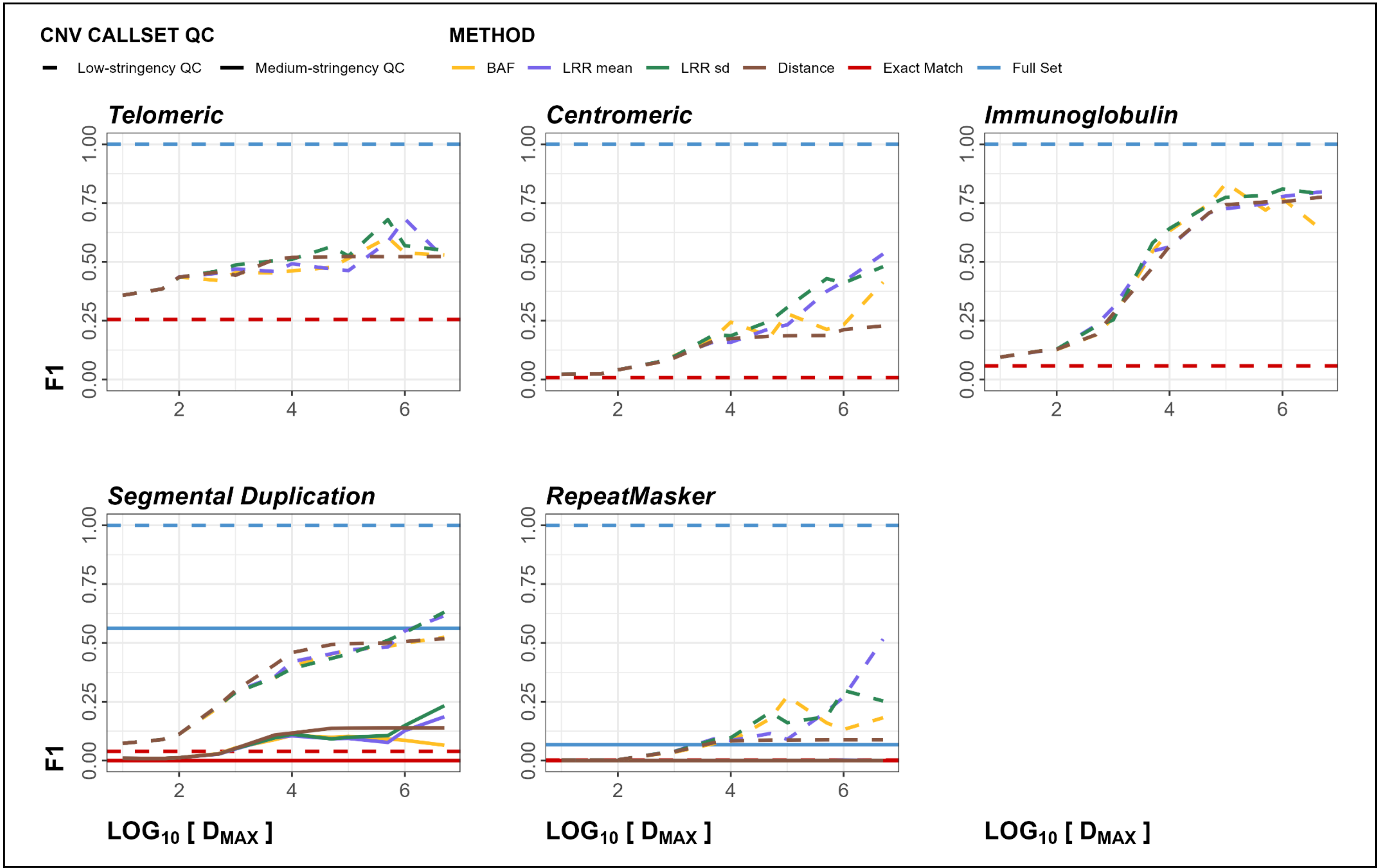

Figure S30. Within-Array Experiment F1 regional plots for both deletions and duplications. Panels show telomeric, centromeric, immunoglobulin, segmental duplication, and repeat masker (simple repeat) regions in order, left to right. Dashed line represents low-stringency QC, solid line represents medium stringency QC.

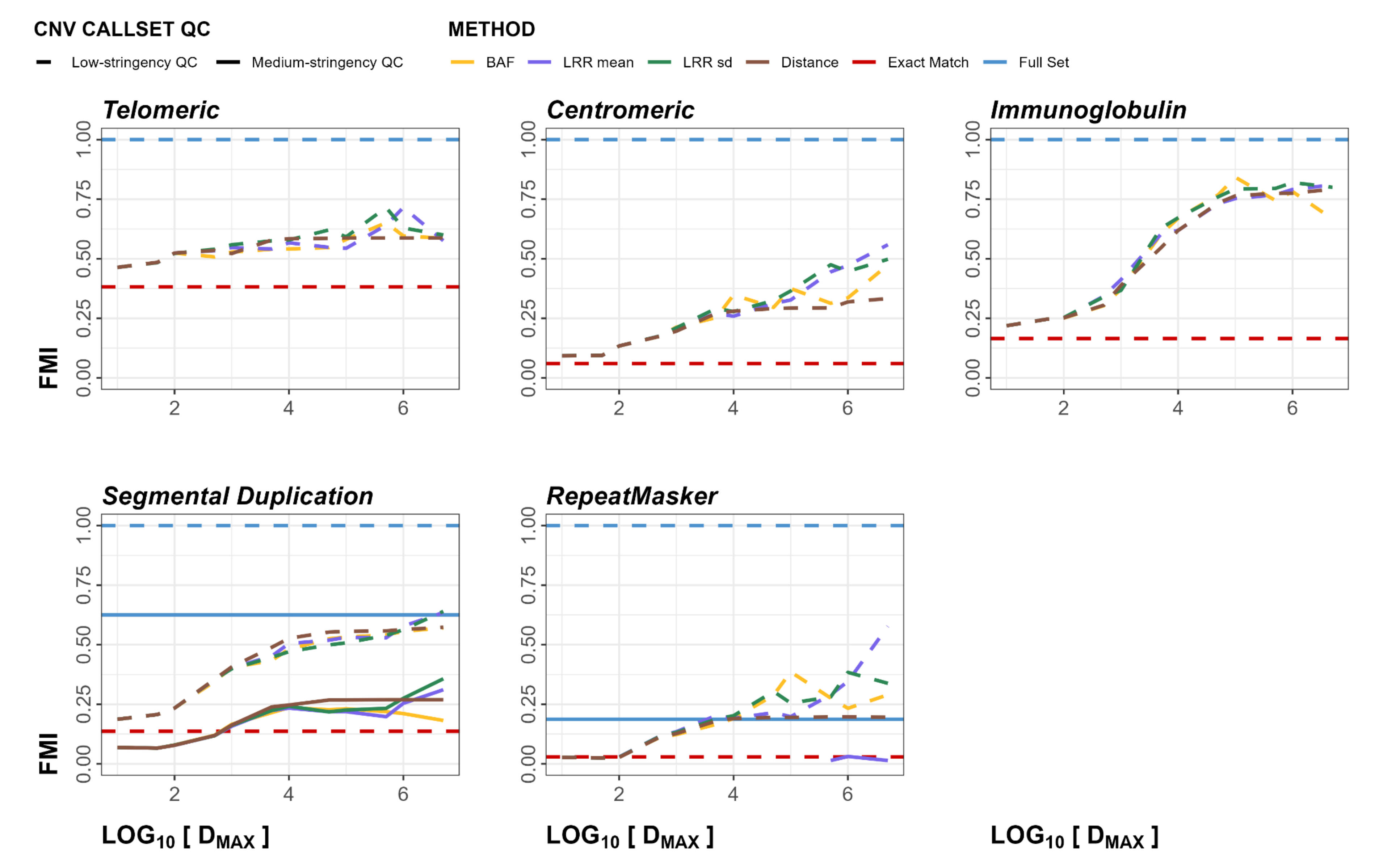

Figure S31. Within-Array Experiment FMI regional plots for both deletions and duplications. Panels show telomeric, centromeric, immunoglobulin, segmental duplication, and repeat masker (simple repeat) regions in order, left to right. Dashed line represents low-stringency QC, solid line represents medium stringency QC.

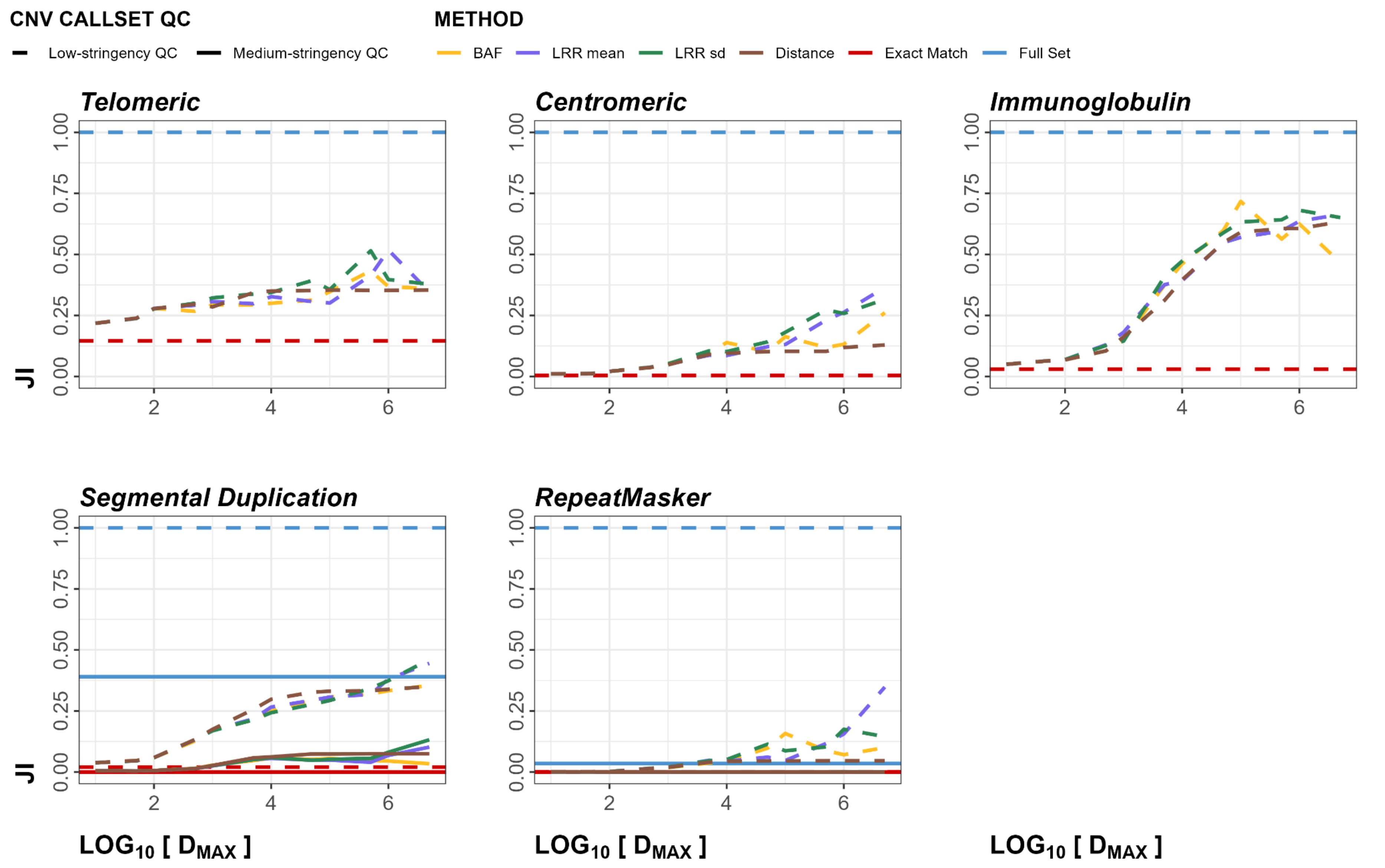

Figure S32. Within-Array Experiment JI regional plots for both deletions and duplications. Panels show telomeric, centromeric, immunoglobulin, segmental duplication, and repeat masker (simple repeat) regions in order, left to right. Dashed line represents low-stringency QC, solid line represents medium stringency QC.

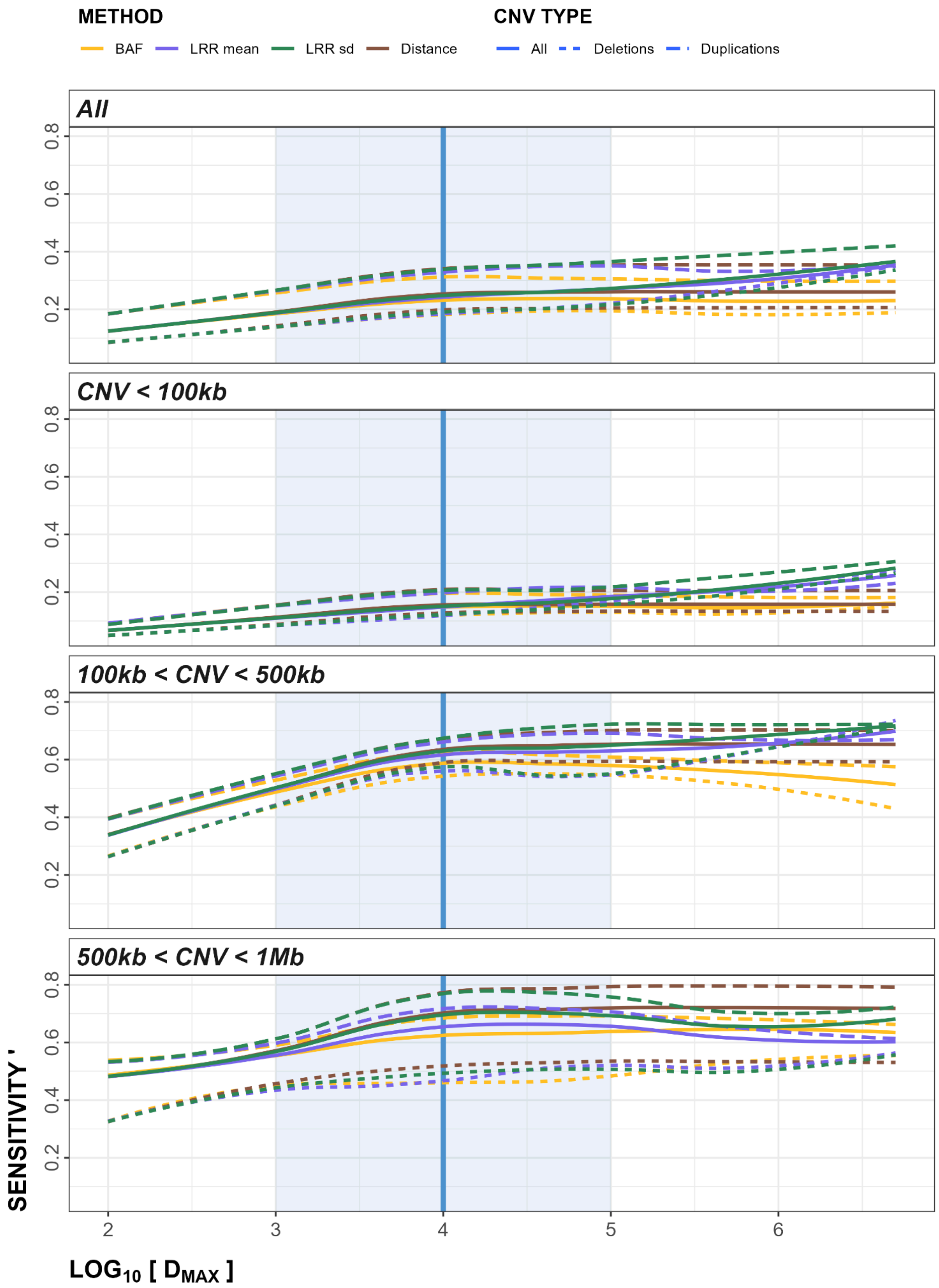

Figure S33. Within-Array Experiment plots of smoothed, scaled sensitivity (sensitivity') metrics to determine optimal D_{MAX} parameter. The blue line represents D_{MAX} = 10kb, the shaded blue region represents 1kb < D_{MAX} < 100kb.

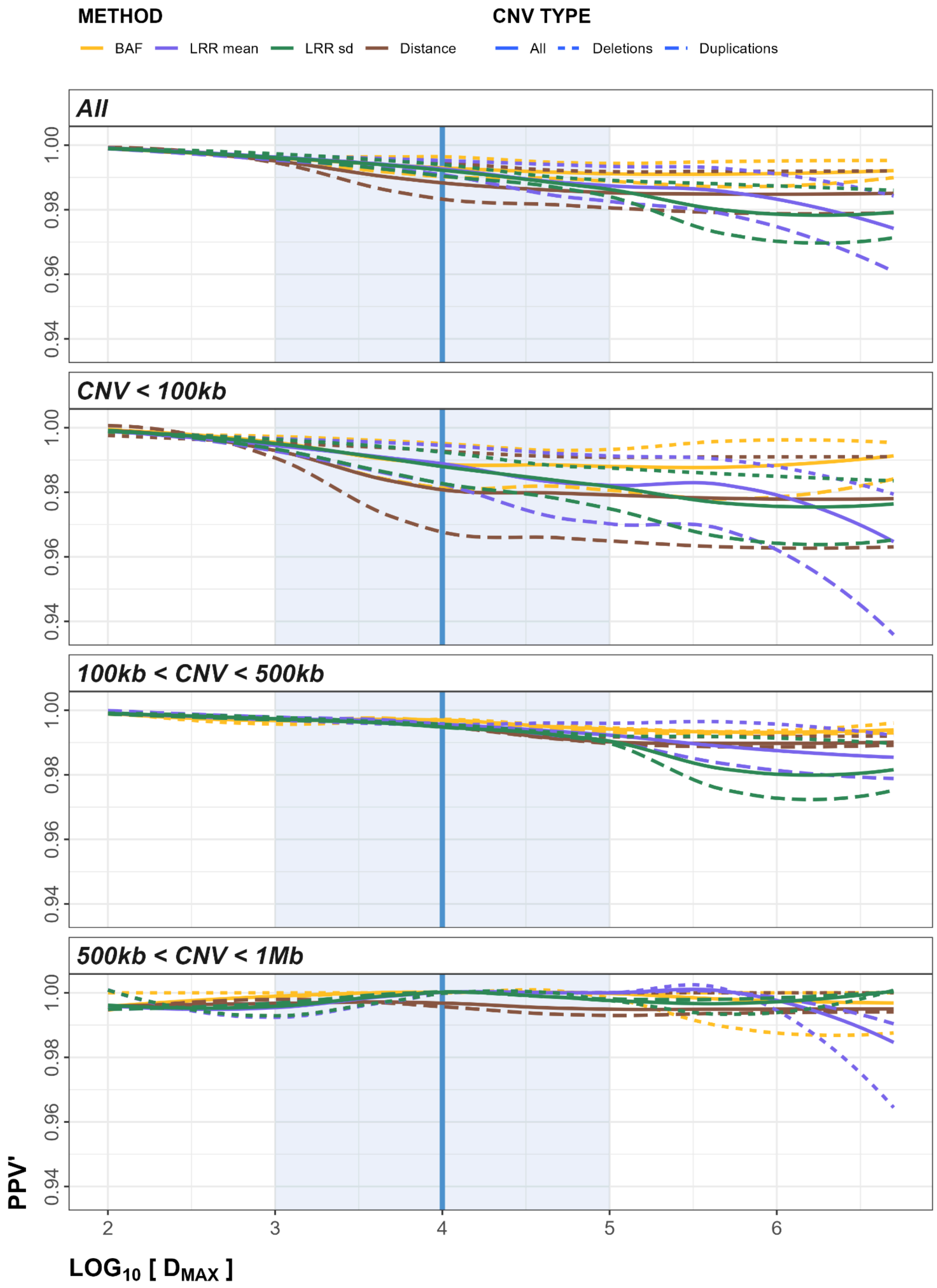

Figure S34. Within-Array Experiment plots of smoothed, scaled PPV (PPV') metrics to determine optimal D_{MAX} parameter. The blue line represents D_{MAX} = 10kb, the shaded blue region represents 1kb < D_{MAX} < 100kb.

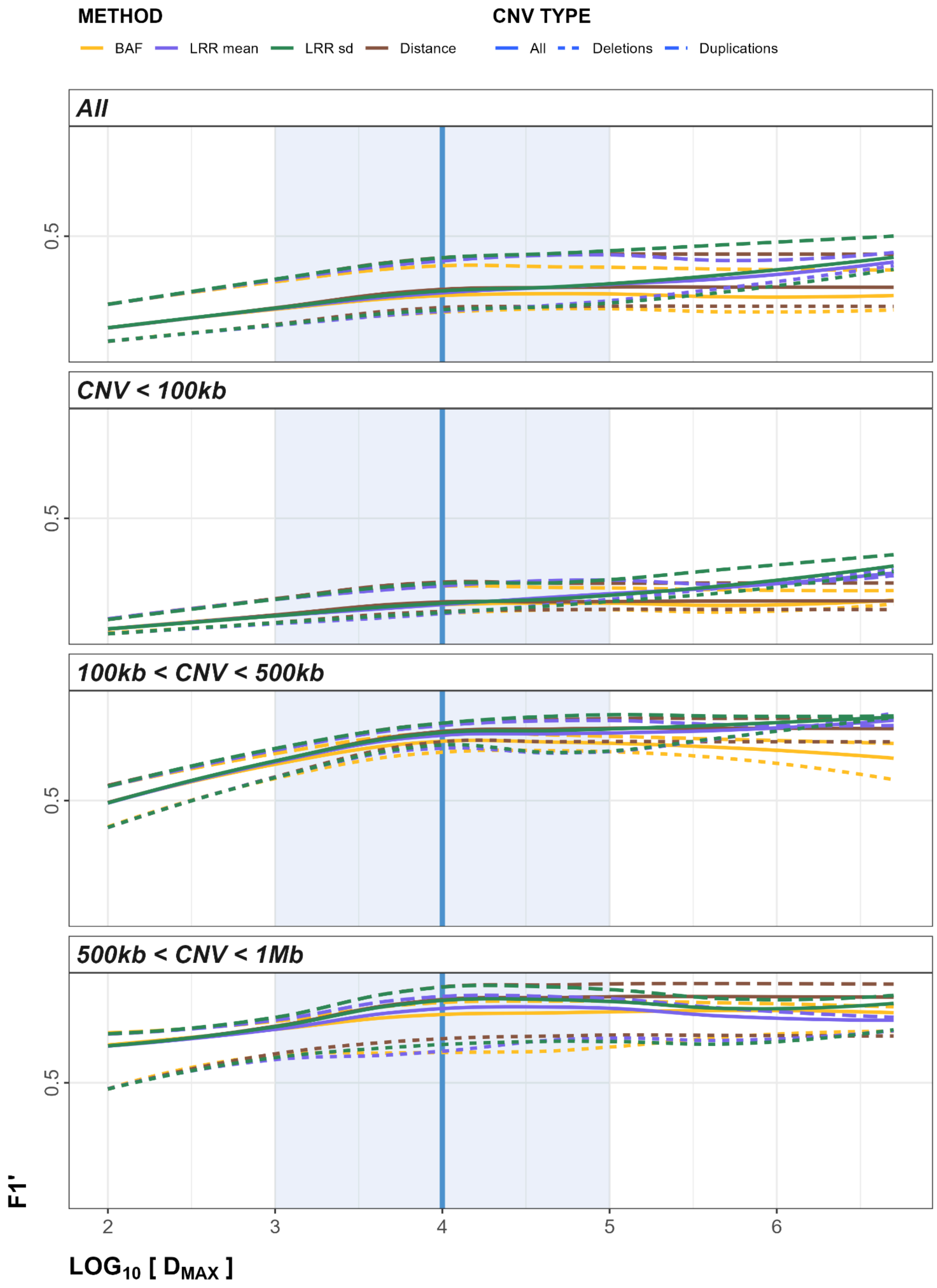

Figure S35. Within-Array Experiment plots of smoothed, scaled F1 (F1‘) metrics to determine optimal D_{MAX} parameter. The blue line represents D_{MAX} = 10kb, the shaded blue region represents 1kb < D_{MAX} < 100kb.

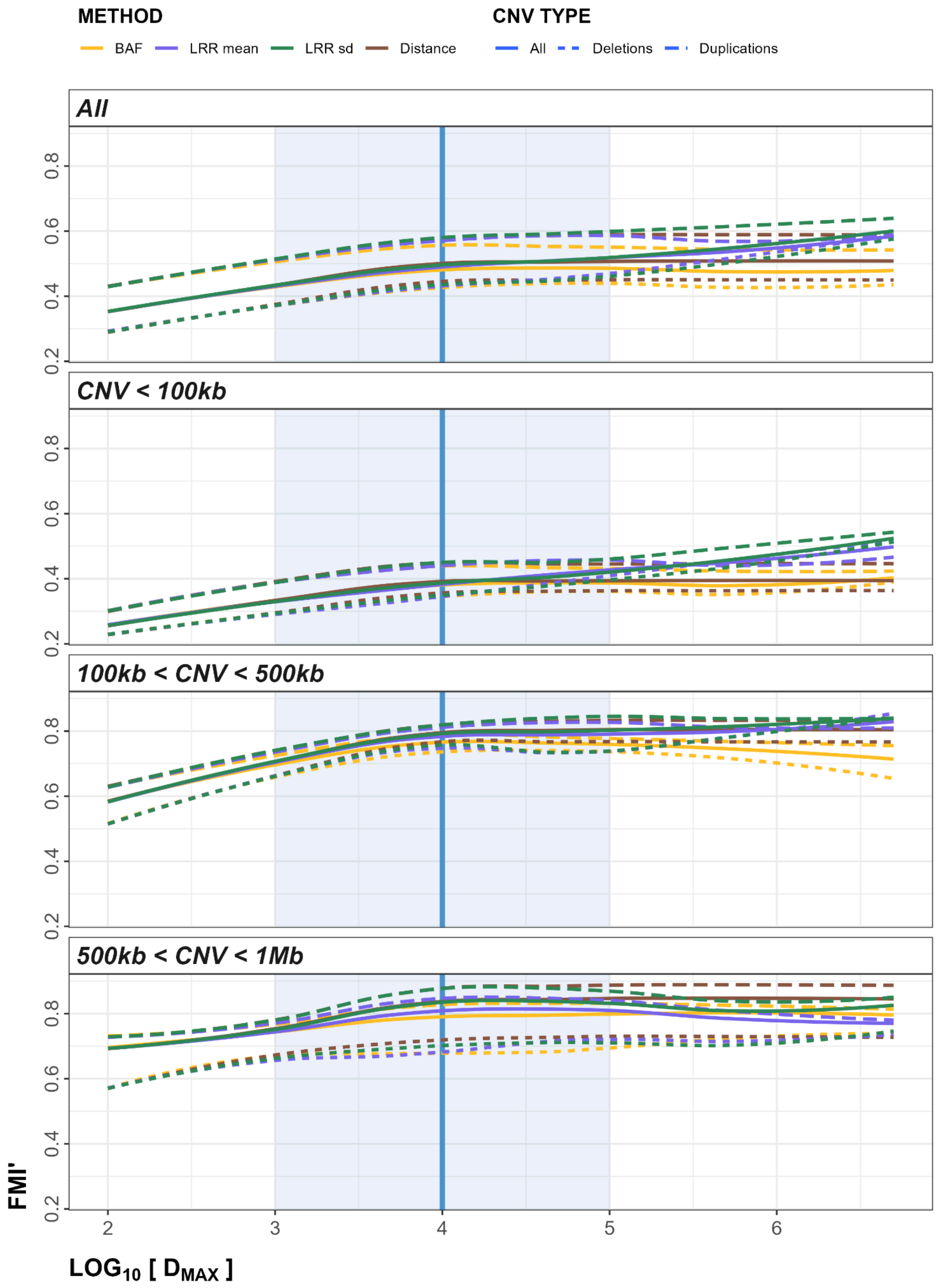

Figure S36. Within-Array Experiment plots of smoothed, scaled FMI (FMI') metrics to determine optimal D_{MAX} parameter. The blue line represents D_{MAX} = 10kb, the shaded blue region represents 1kb < D_{MAX} < 100kb.

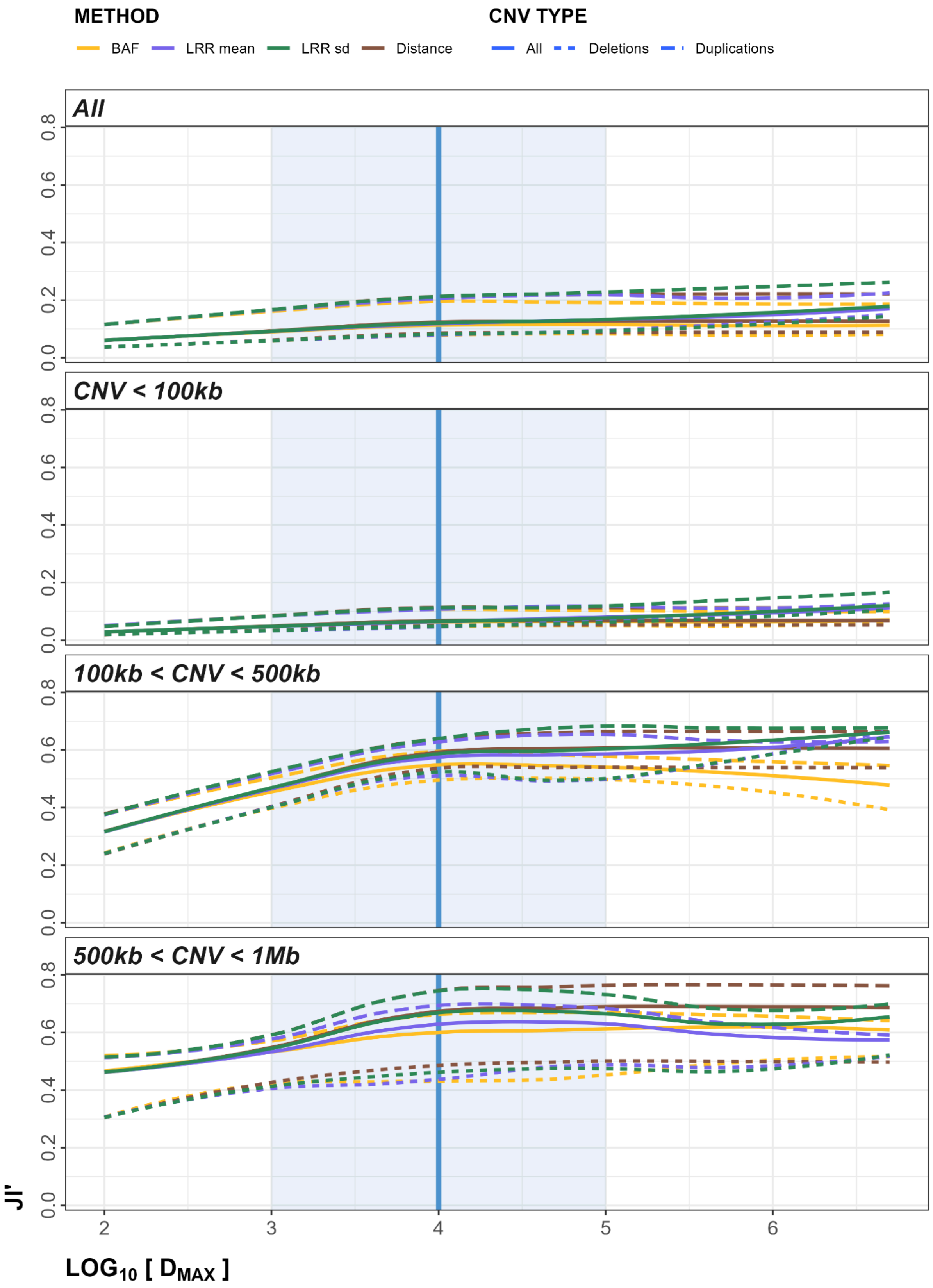

Figure S37. Within-Array Experiment plots of smoothed, scaled JI (JI') metrics to determine optimal D_{MAX} parameter. The blue line represents D_{MAX} = 10kb, the shaded blue region represents 1kb < D_{MAX} < 100kb.

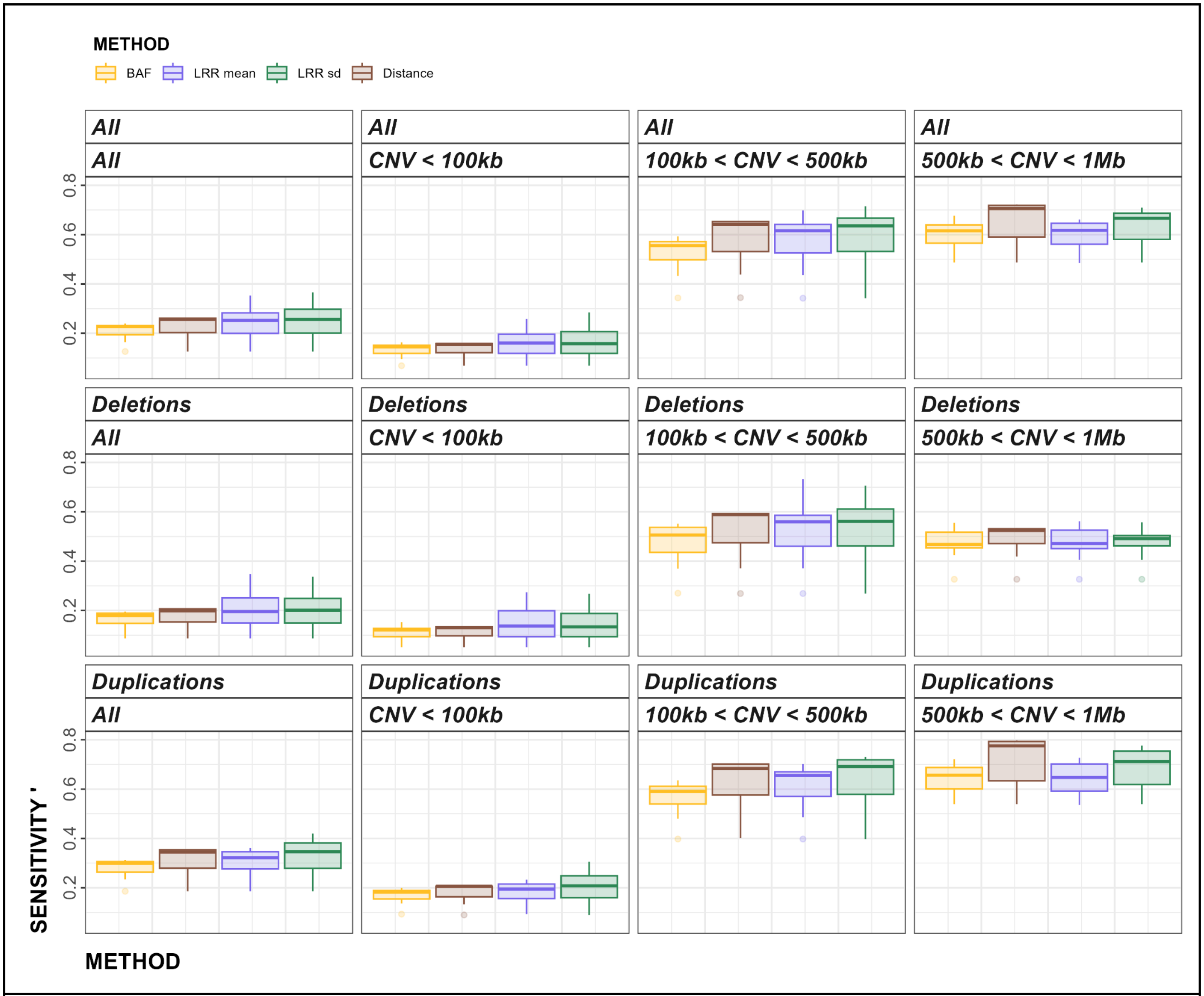

Figure S38. Within-Array Experiment plots of smoothed, scaled sensitivity (sensitivity’) metrics to determine optimal Method parameter.

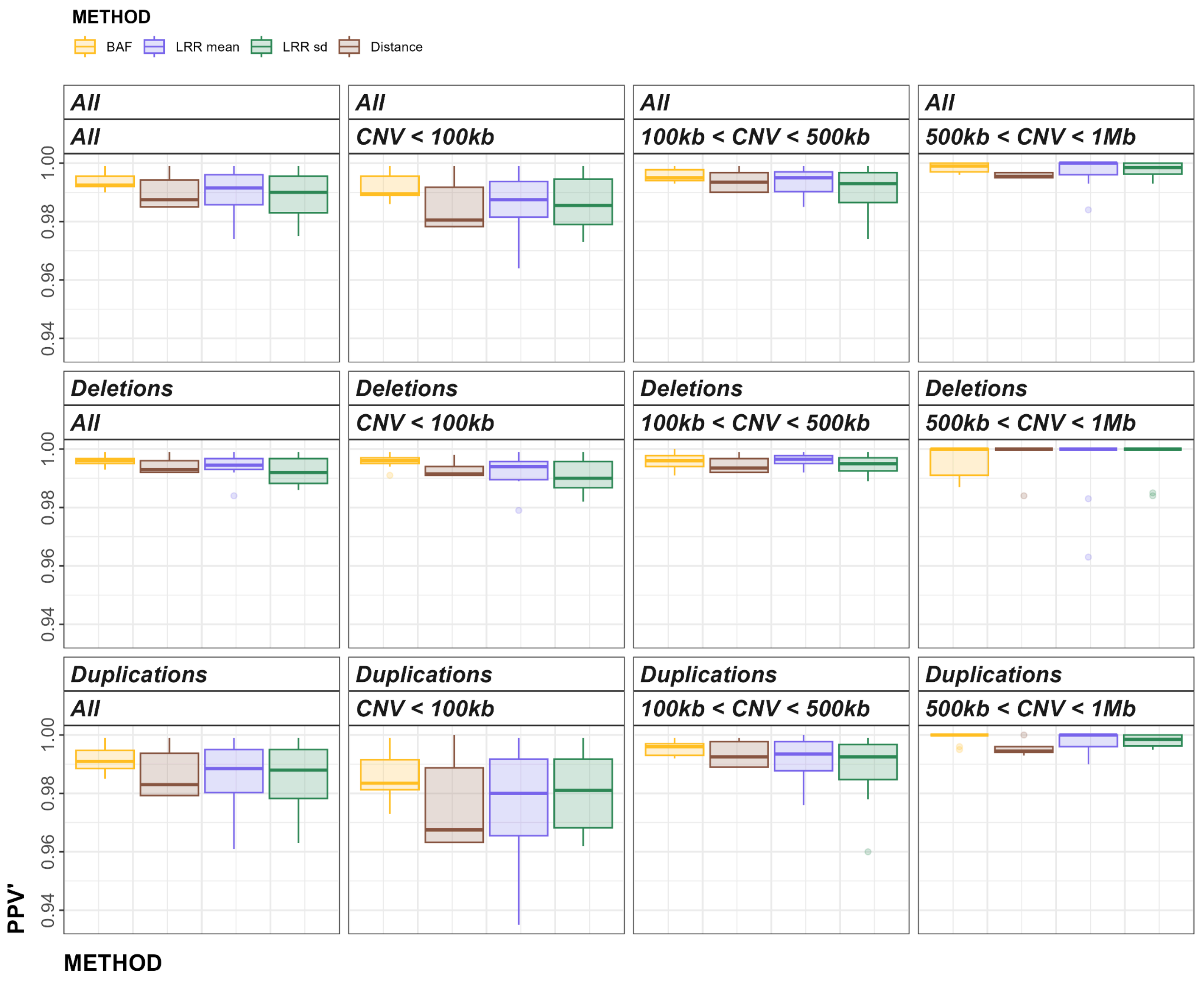

Figure S39. Within-Array Experiment plots of smoothed, scaled PPV (PPV') metrics to determine optimal Method parameter.

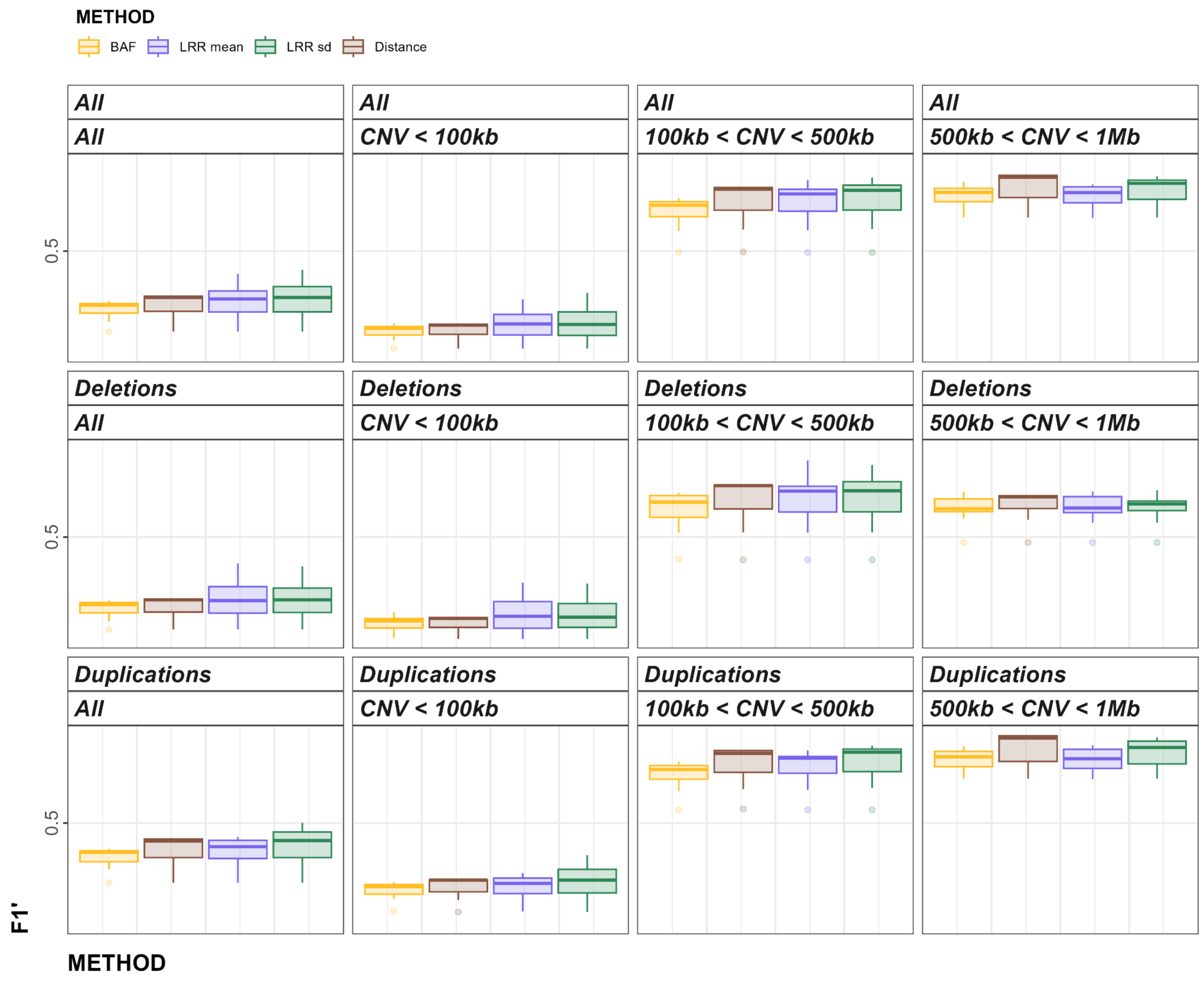

Figure S40. Within-Array Experiment plots of smoothed, scaled F1 (F1') metrics to determine optimal Method parameter.

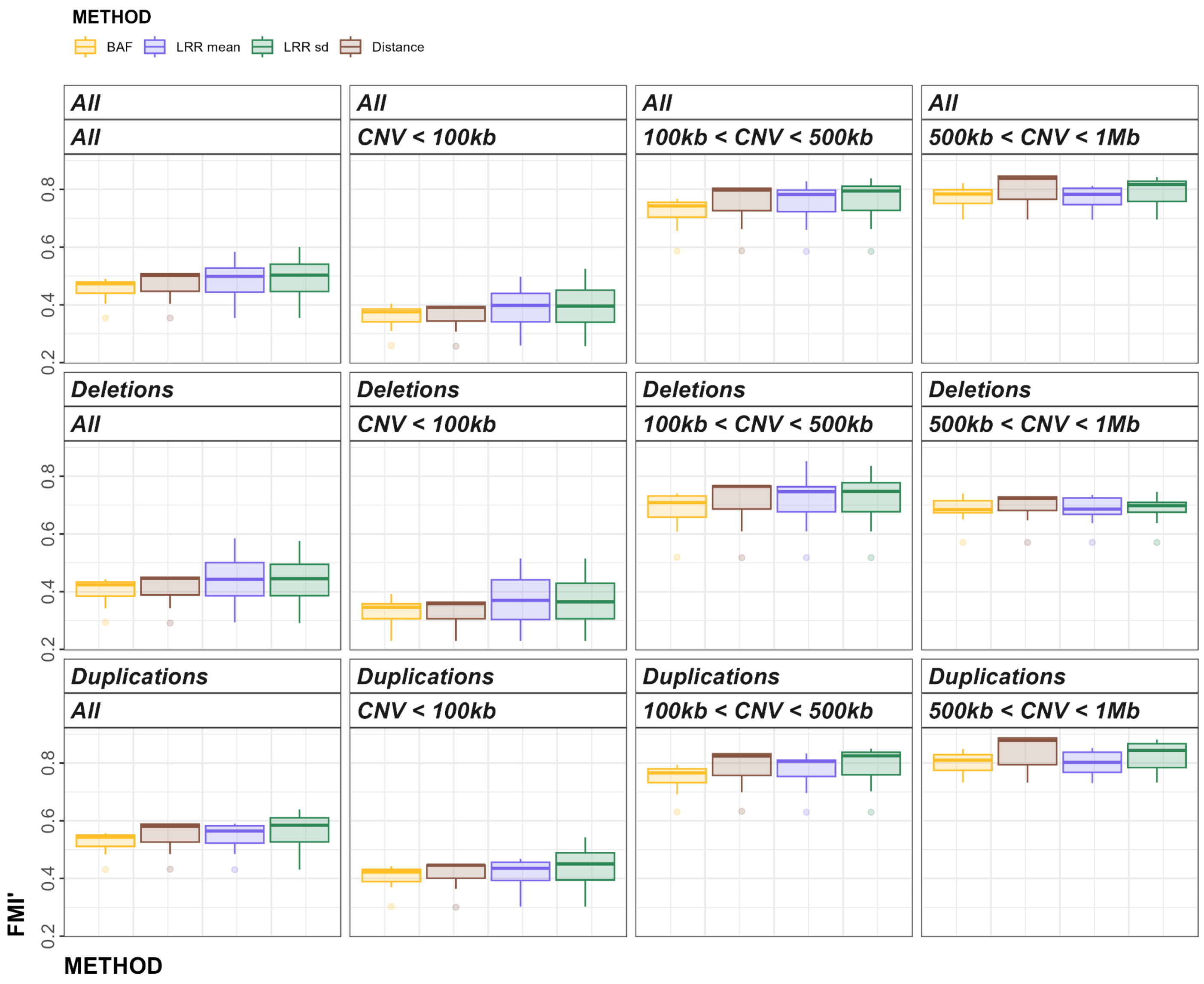

Figure S41. Within-Array Experiment plots of smoothed, scaled FMI (FMI') metrics to determine optimal Method parameter.

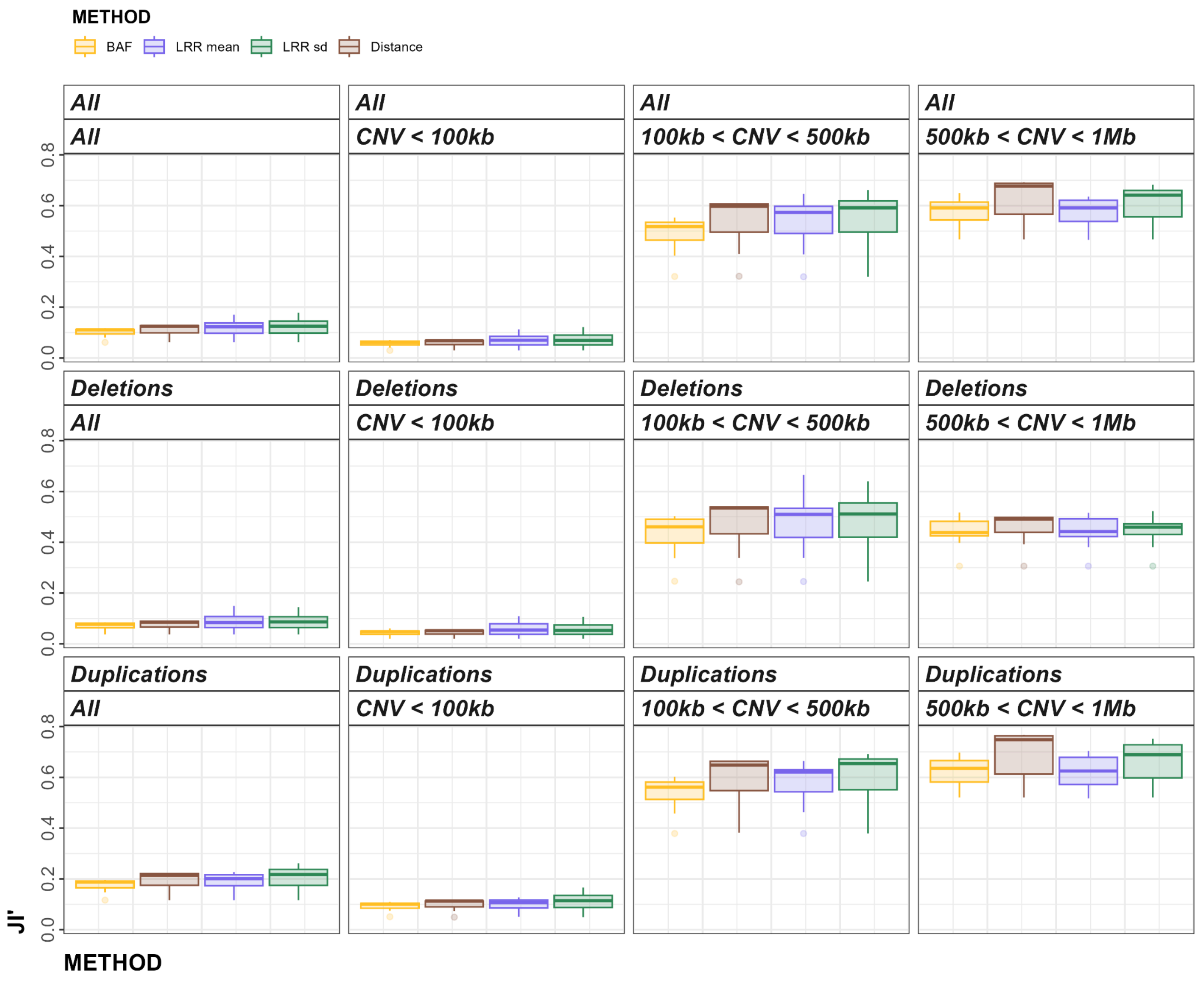

Figure S42. Within-Array Experiment plots of smoothed, scaled JI (JI') metrics to determine optimal Method parameter.

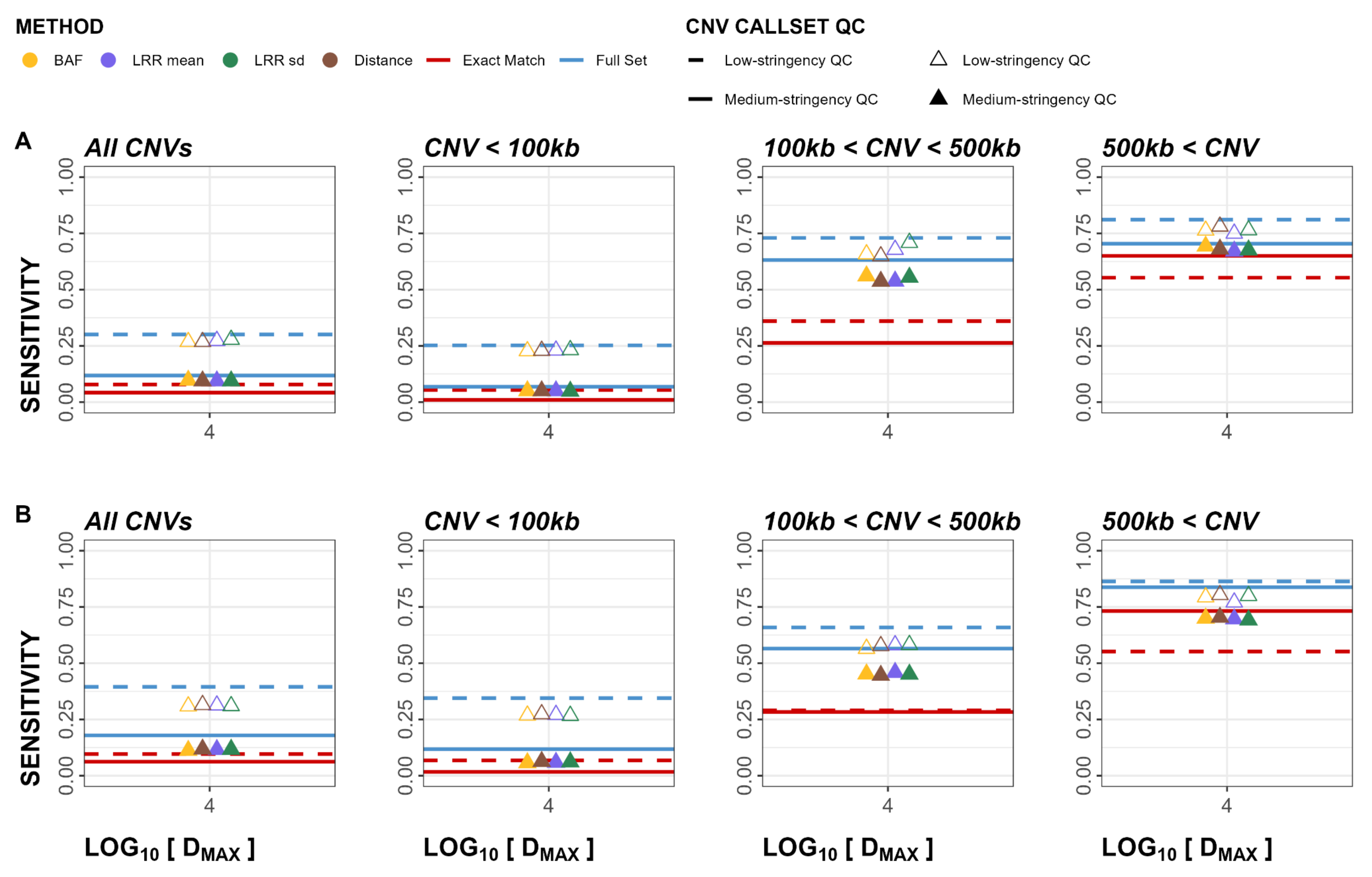

Figure S43. Cross-Array Experiment sensitivity plots for both deletions and duplications. Panel A represents calls in GSA1validated in OEE. Panel B represents calls in OEE validated in GSA1. Empty triangles and dashed lines represent low-stringency QC callsets, whereas solid lines and filled triangles represent medium-stringency QC callsets. GSA1: Global Screening Array, OEE: Omni Express Exome array.

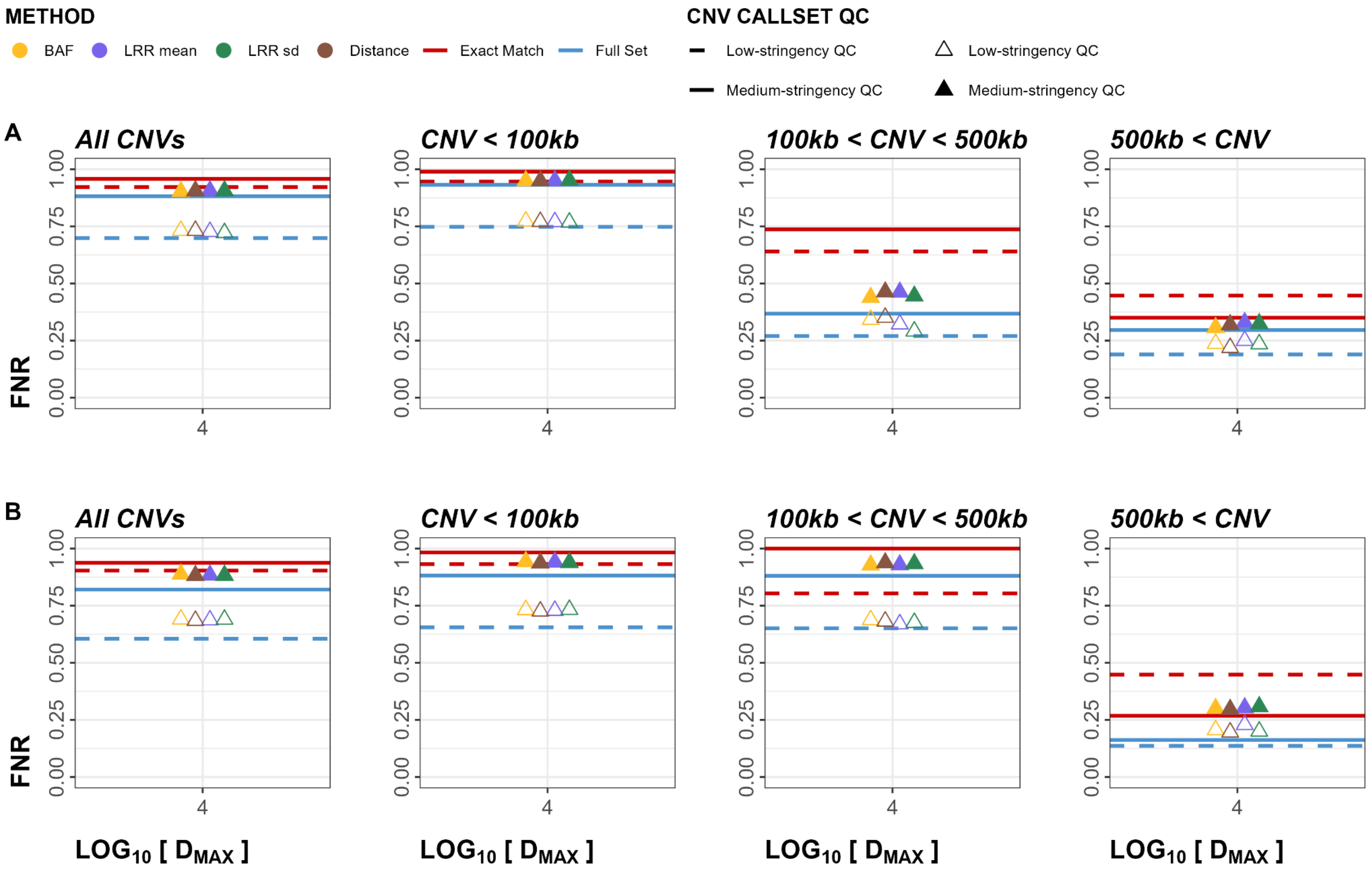

Figure S44. Cross-Array Experiment FNR plots for both deletions and duplications. Panel A represents calls in GSA1validated in OEE. Panel B represents calls in OEE validated in GSA1. Empty triangles and dashed lines represent low-stringency QC callsets, whereas solid lines and filled triangles represent medium-stringency QC callsets. GSA1: Global Screening Array, OEE: Omni Express Exome array.

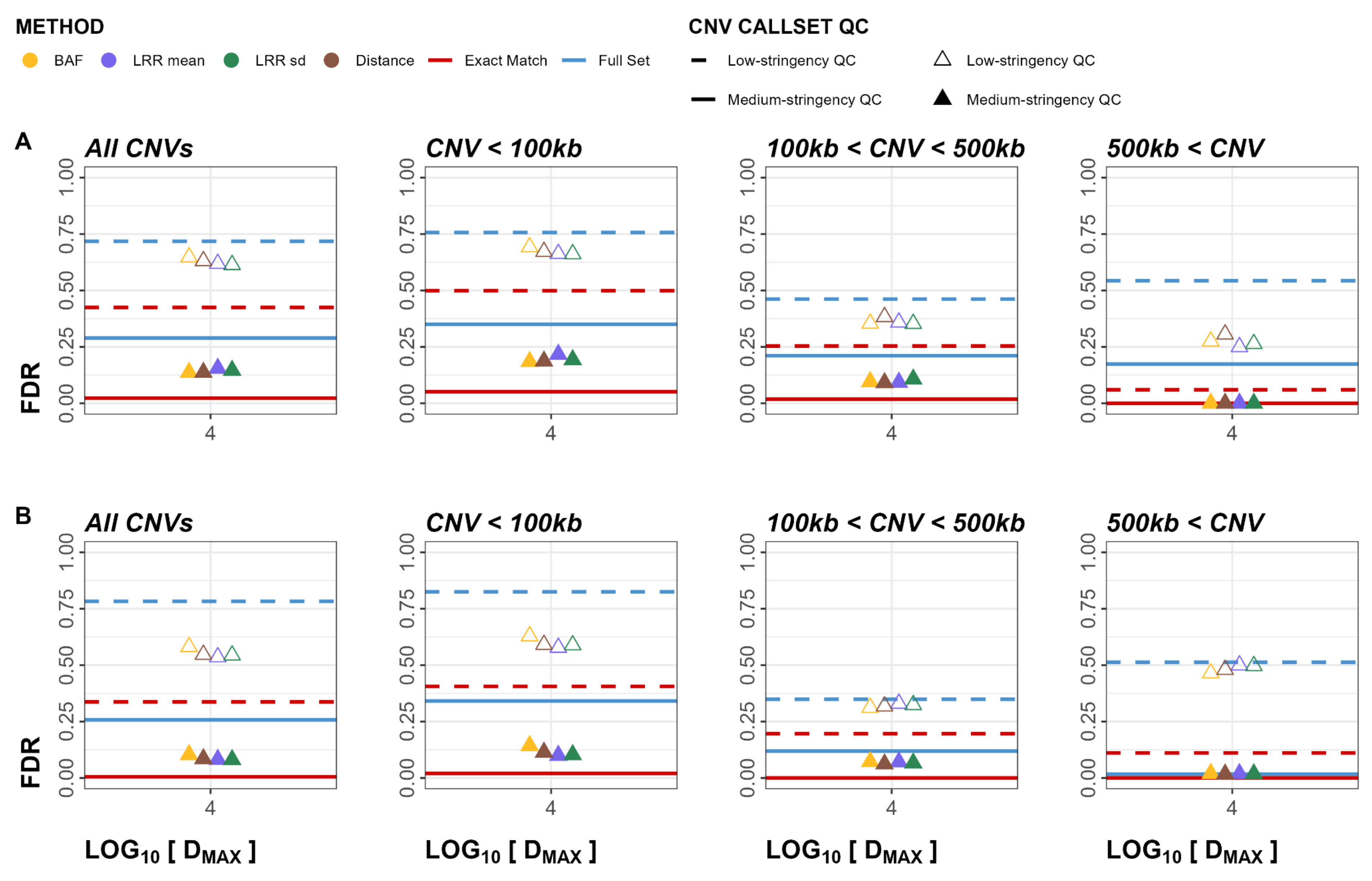

Figure S45. Cross-Array Experiment FDR plots for both deletions and duplications. Panel A represents calls in GSA1validated in OEE. Panel B represents calls in OEE validated in GSA1. Empty triangles and dashed lines represent low-stringency QC callsets, whereas solid lines and filled triangles represent medium-stringency QC callsets. GSA1: Global Screening Array, OEE: Omni Express Exome array.

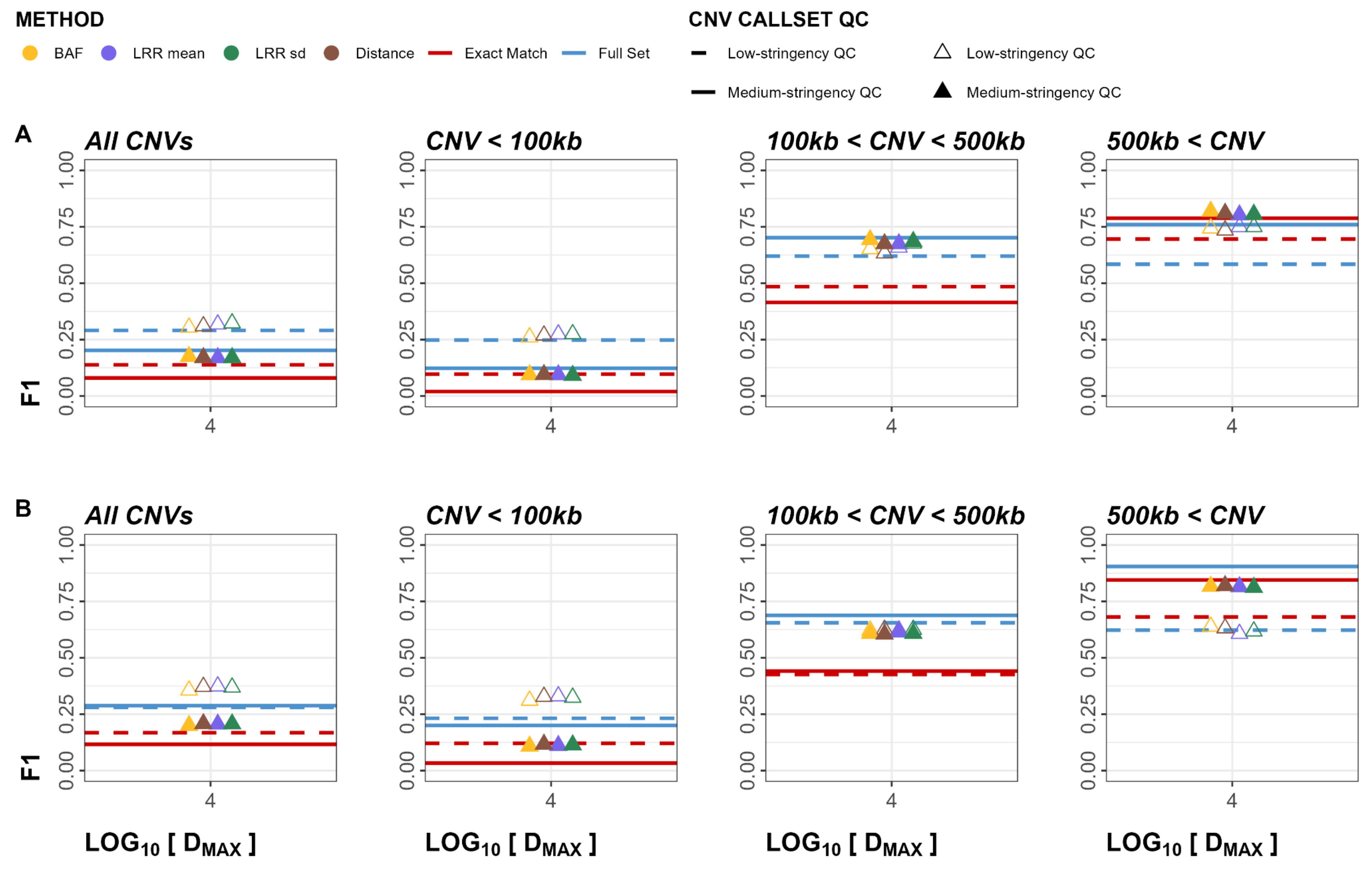

Figure S46. Cross-Array Experiment F1 plots for both deletions and duplications. Panel A represents calls in GSA1validated in OEE. Panel B represents calls in OEE validated in GSA1. Empty triangles and dashed lines represent low-stringency QC callsets, whereas solid lines and filled triangles represent medium-stringency QC callsets. GSA1: Global Screening Array, OEE: Omni Express Exome array.

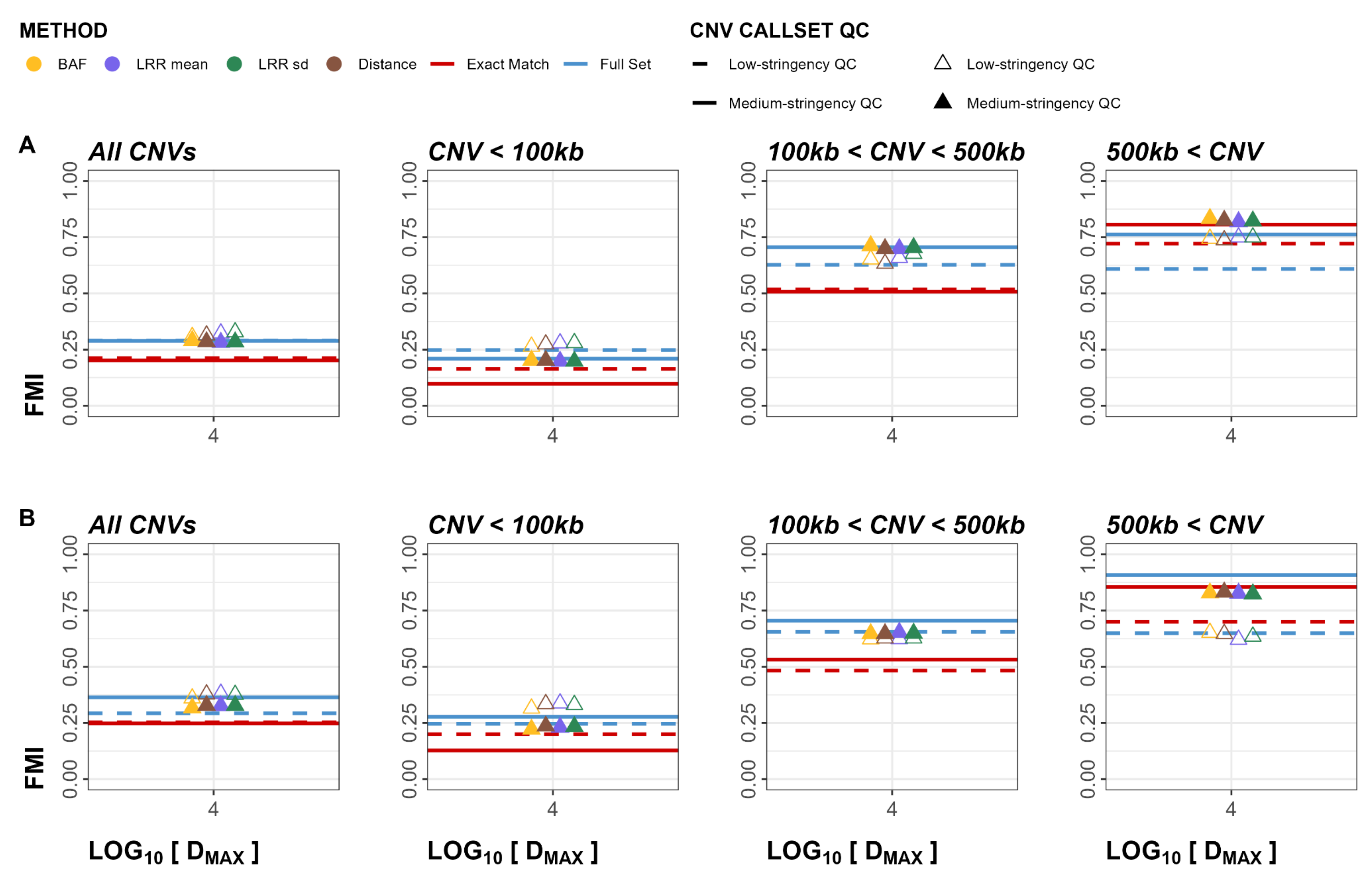

Figure S47. Cross-Array Experiment FMI plots for both deletions and duplications. Panel A represents calls in GSA1validated in OEE. Panel B represents calls in OEE validated in GSA1. Empty triangles and dashed lines represent low-stringency QC callsets, whereas solid lines and filled triangles represent medium-stringency QC callsets. GSA1: Global Screening Array, OEE: Omni Express Exome array.

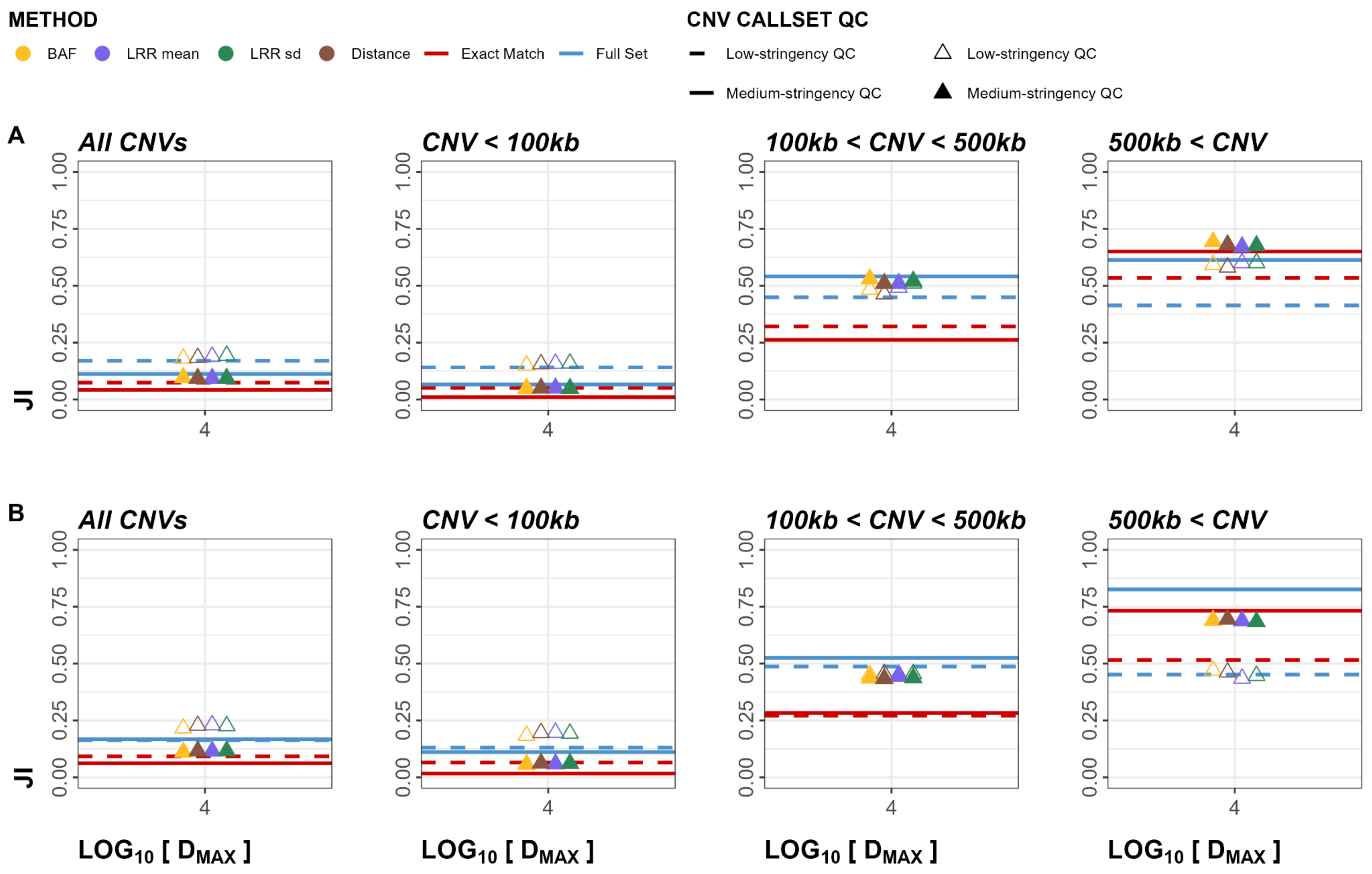

Figure S48. Cross-Array Experiment JI plots for both deletions and duplications. Panel A represents calls in GSA1validated in OEE. Panel B represents calls in OEE validated in GSA1. Empty triangles and dashed lines represent low-stringency QC callsets, whereas solid lines and filled triangles represent medium-stringency QC callsets. GSA1: Global Screening Array, OEE: Omni Express Exome array.

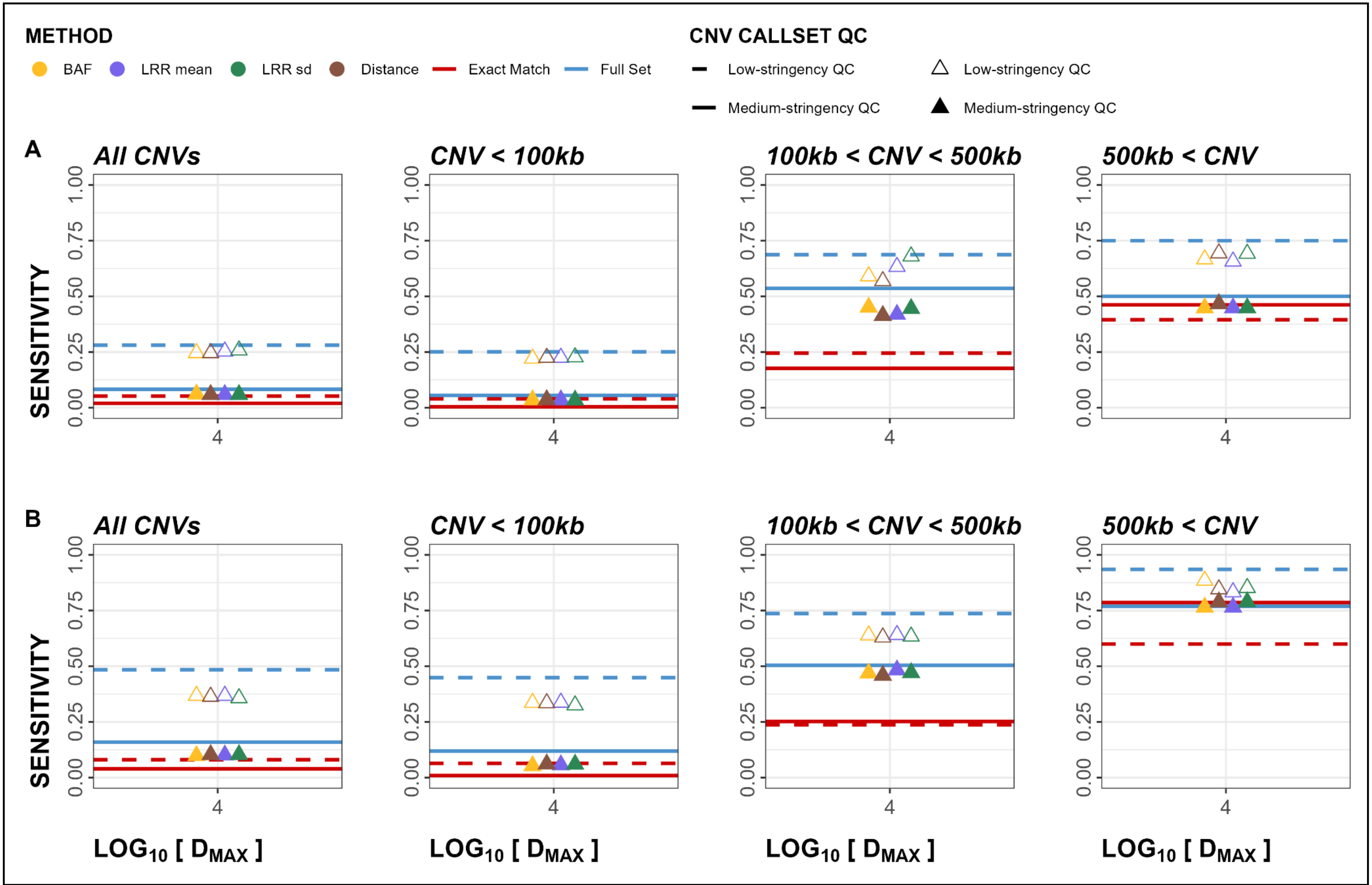

Figure S49. Cross-Array Experiment sensitivity plots for deletions. Panel A represents calls in GSA1validated in OEE. Panel B represents calls in OEE validated in GSA1. Empty triangles and dashed lines represent low-stringency QC callsets, whereas solid lines and filled triangles represent medium-stringency QC callsets. GSA1: Global Screening Array, OEE: Omni Express Exome array.

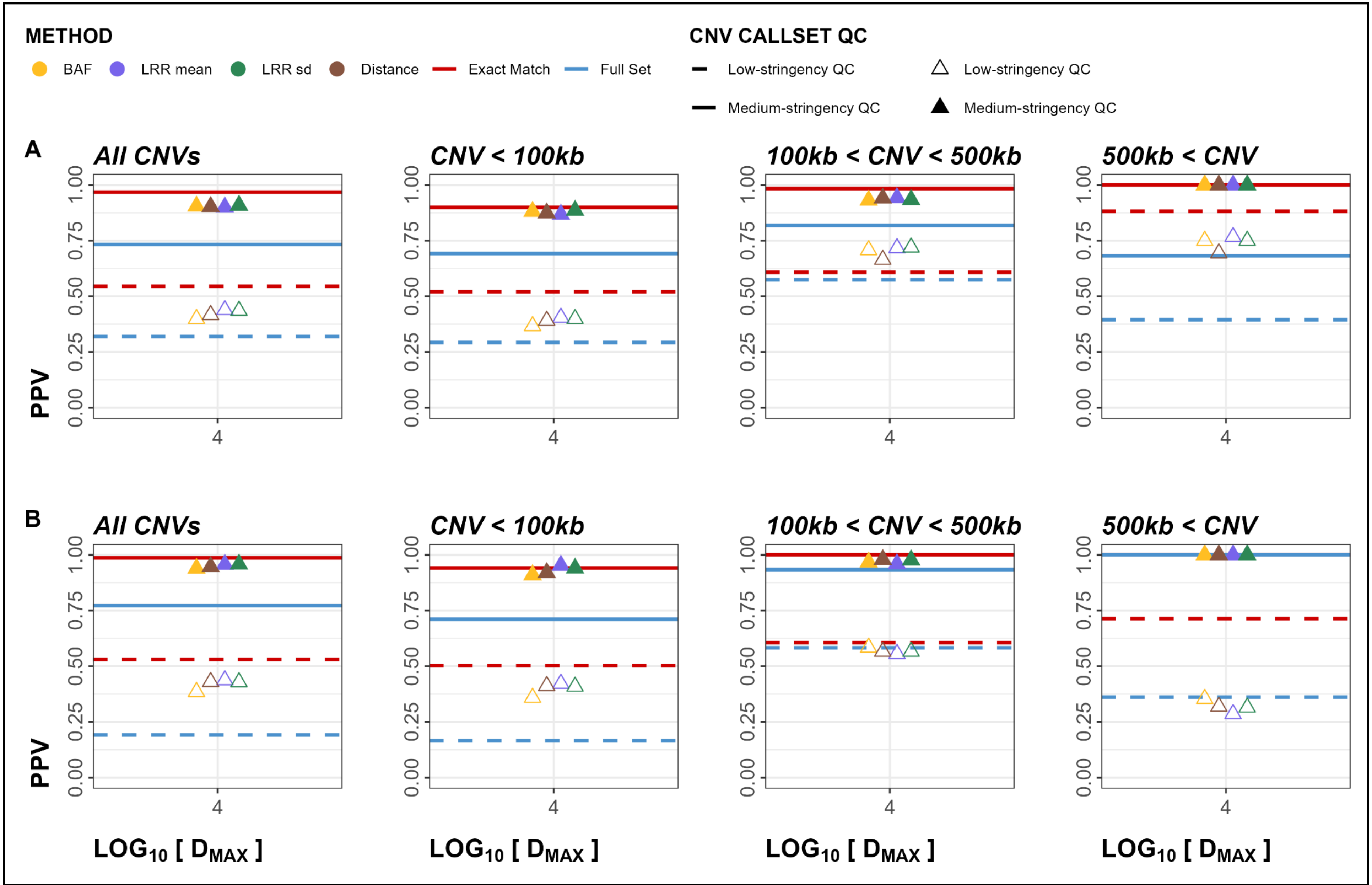

Figure S50. Cross-Array Experiment PPV plots for deletions. Panel A represents calls in GSA1validated in OEE. Panel B represents calls in OEE validated in GSA1. Empty triangles and dashed lines represent low-stringency QC callsets, whereas solid lines and filled triangles represent medium-stringency QC callsets. GSA1: Global Screening Array, OEE: Omni Express Exome array.

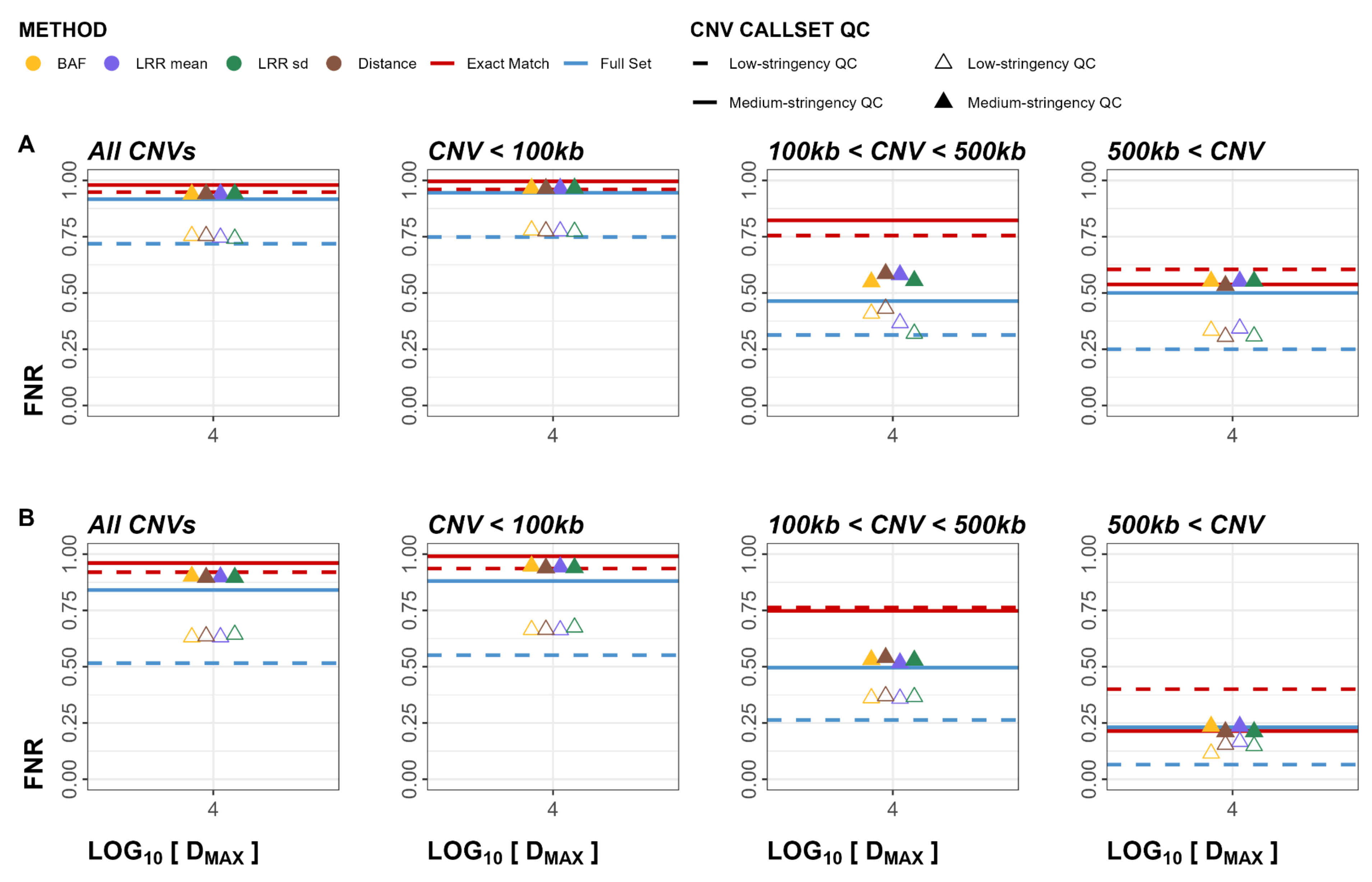

Figure S51. Cross-Array Experiment FNR plots for deletions. Panel A represents calls in GSA1validated in OEE. Panel B represents calls in OEE validated in GSA1. Empty triangles and dashed lines represent low-stringency QC callsets, whereas solid lines and filled triangles represent medium-stringency QC callsets. GSA1: Global Screening Array, OEE: Omni Express Exome array.

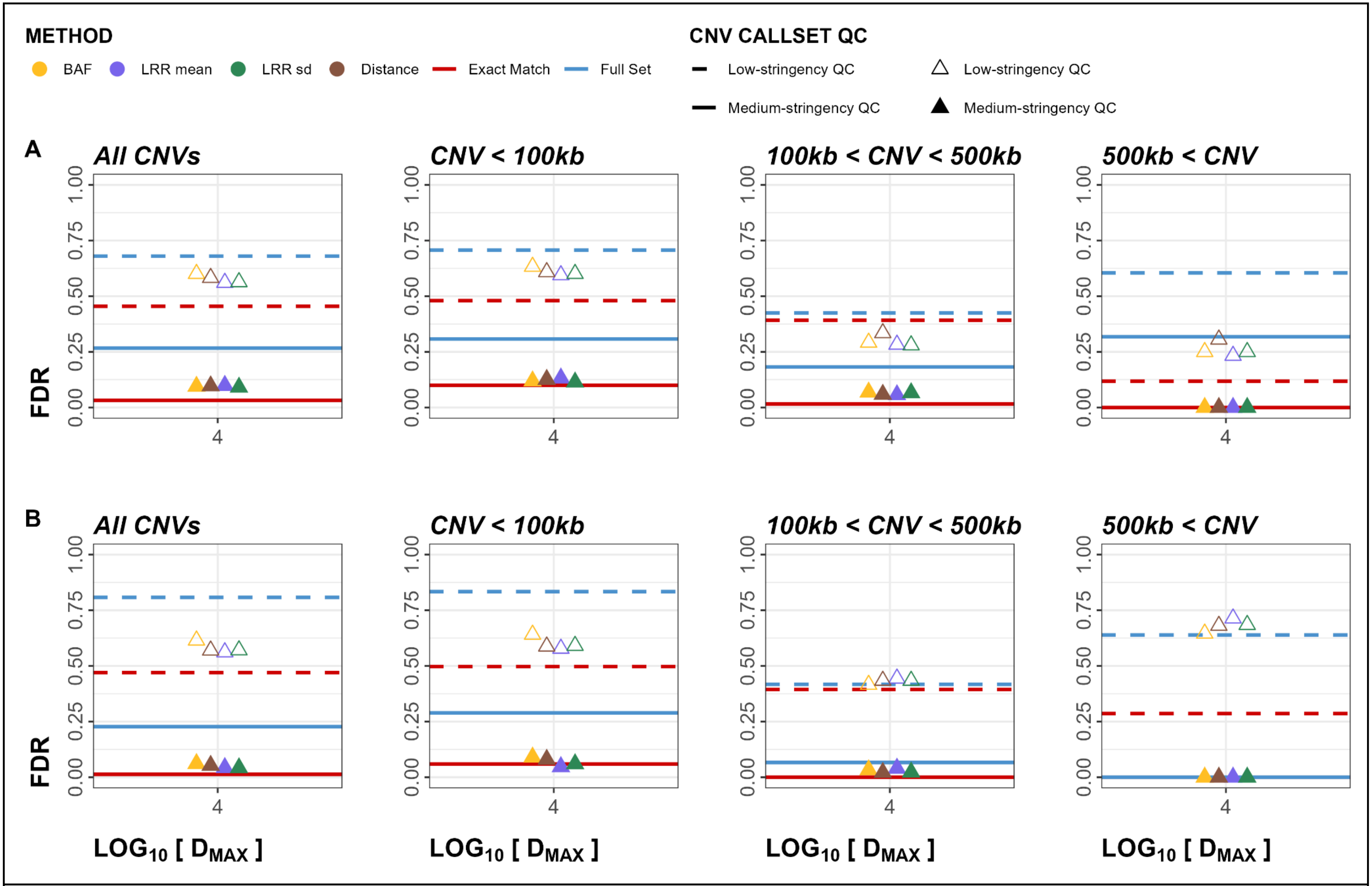

Figure S52. Cross-Array Experiment FDR plots for deletions. Panel A represents calls in GSA1validated in OEE. Panel B represents calls in OEE validated in GSA1. Empty triangles and dashed lines represent low-stringency QC callsets, whereas solid lines and filled triangles represent medium-stringency QC callsets. GSA1: Global Screening Array, OEE: Omni Express Exome array.

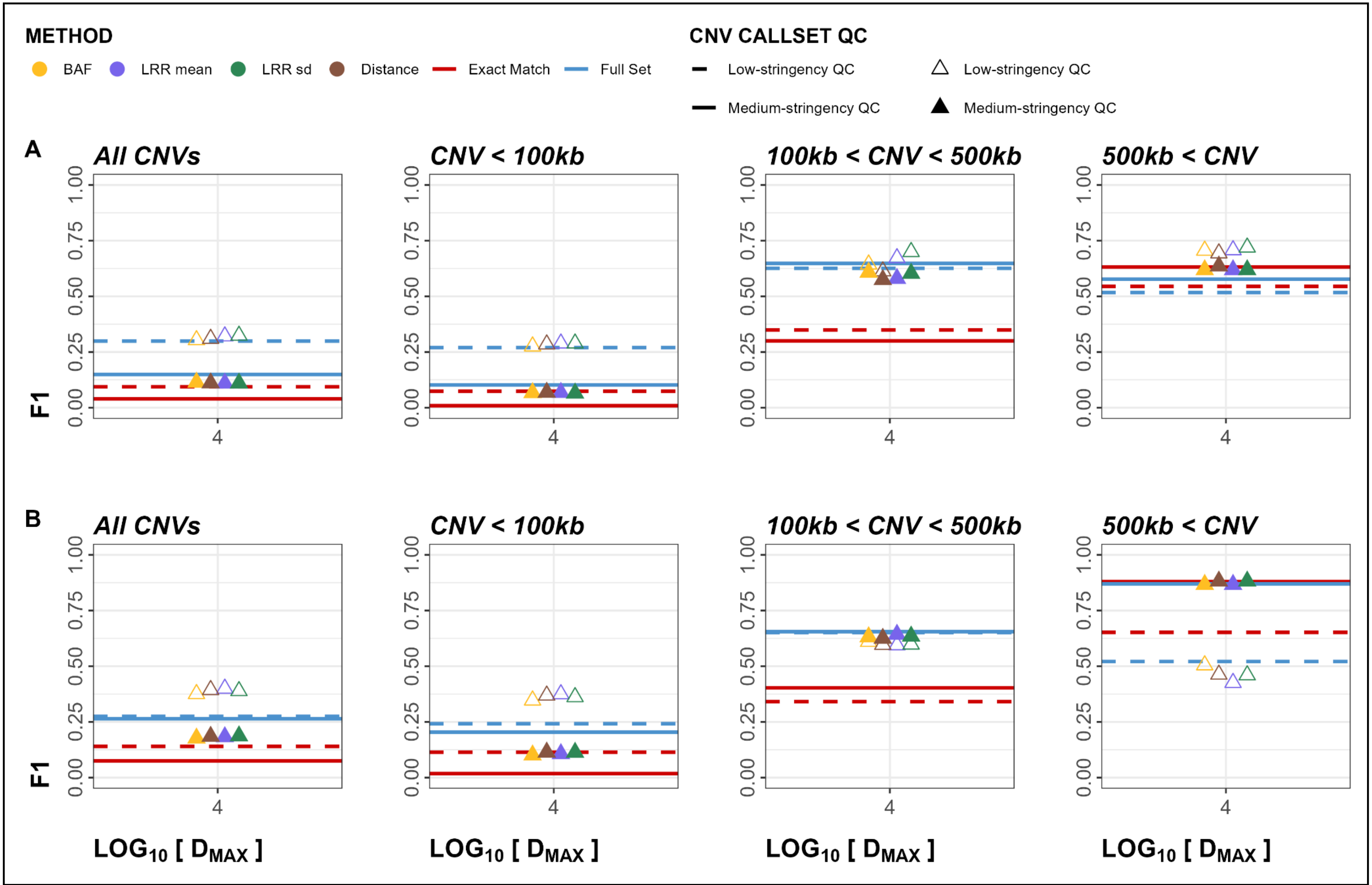

Figure S53. Cross-Array Experiment F1 plots for deletions. Panel A represents calls in GSA1validated in OEE. Panel B represents calls in OEE validated in GSA1. Empty triangles and dashed lines represent low-stringency QC callsets, whereas solid lines and filled triangles represent medium-stringency QC callsets. GSA1: Global Screening Array, OEE: Omni Express Exome array.

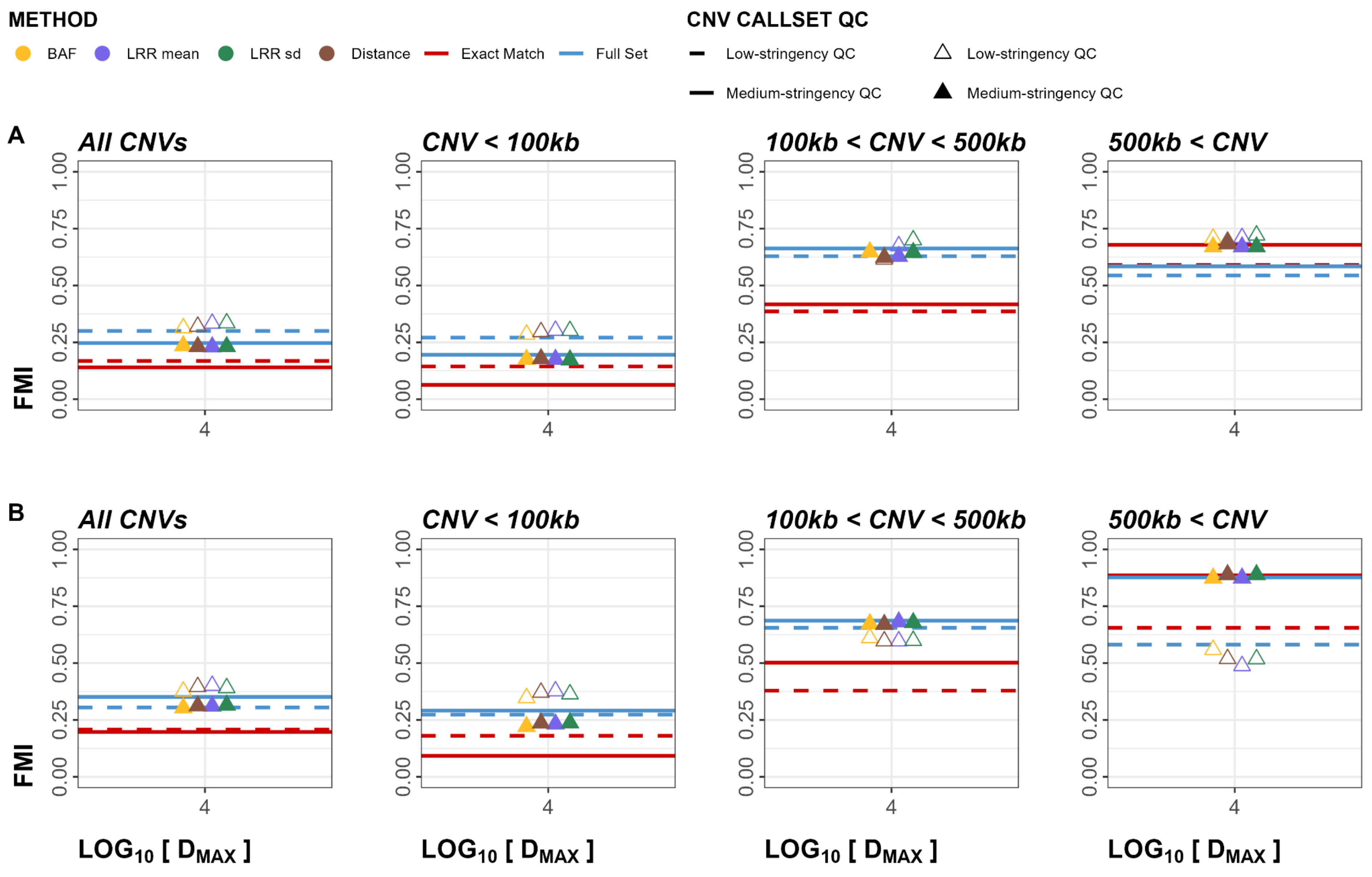

Figure S54. Cross-Array Experiment FMI plots for deletions. Panel A represents calls in GSA1validated in OEE. Panel B represents calls in OEE validated in GSA1. Empty triangles and dashed lines represent low-stringency QC callsets, whereas solid lines and filled triangles represent medium-stringency QC callsets. GSA1: Global Screening Array, OEE: Omni Express Exome array.

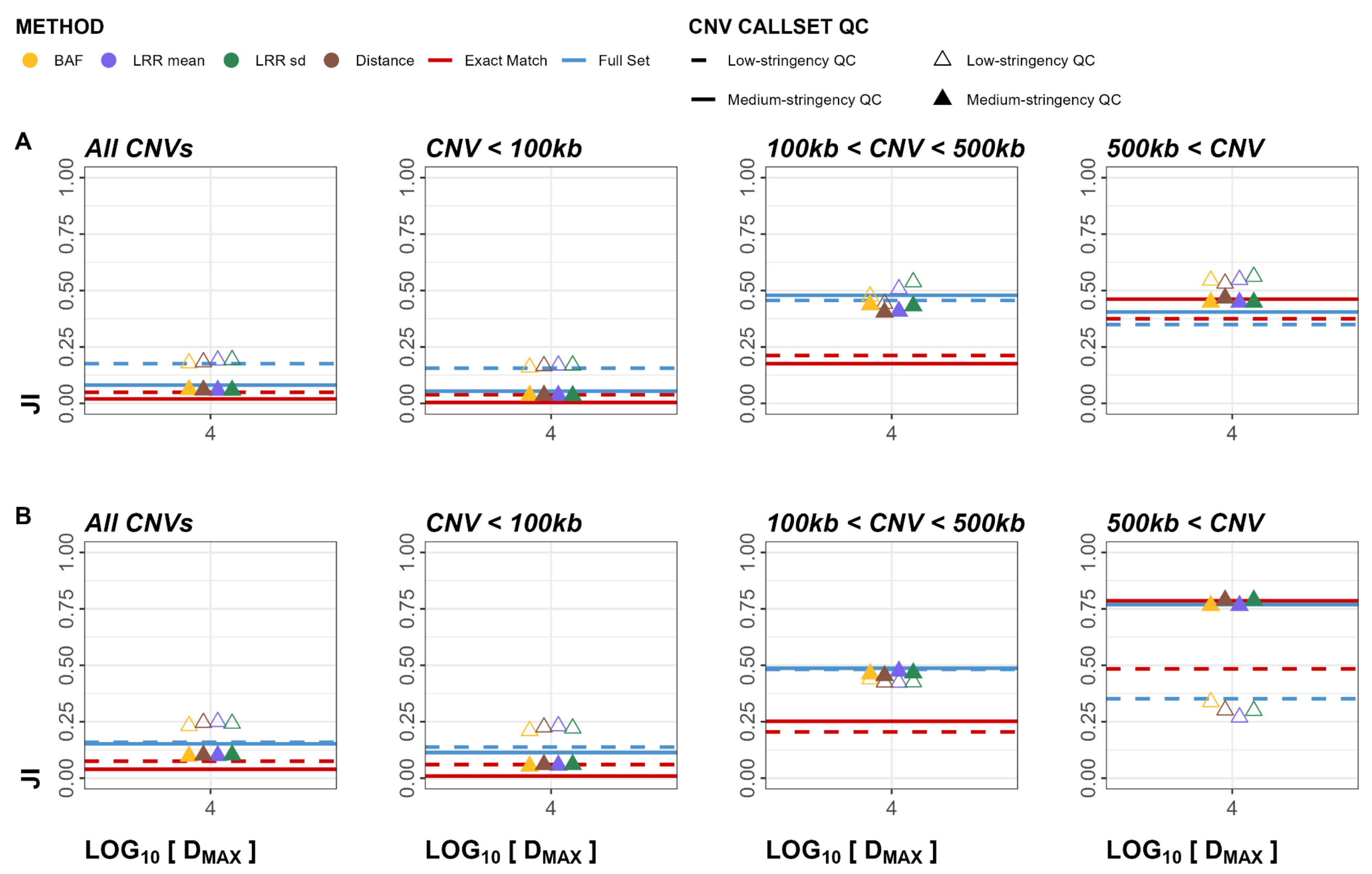

Figure S55. Cross-Array Experiment JI plots for deletions. Panel A represents calls in GSA1validated in OEE. Panel B represents calls in OEE validated in GSA1. Empty triangles and dashed lines represent low-stringency QC callsets, whereas solid lines and filled triangles represent medium-stringency QC callsets. GSA1: Global Screening Array, OEE: Omni Express Exome array.

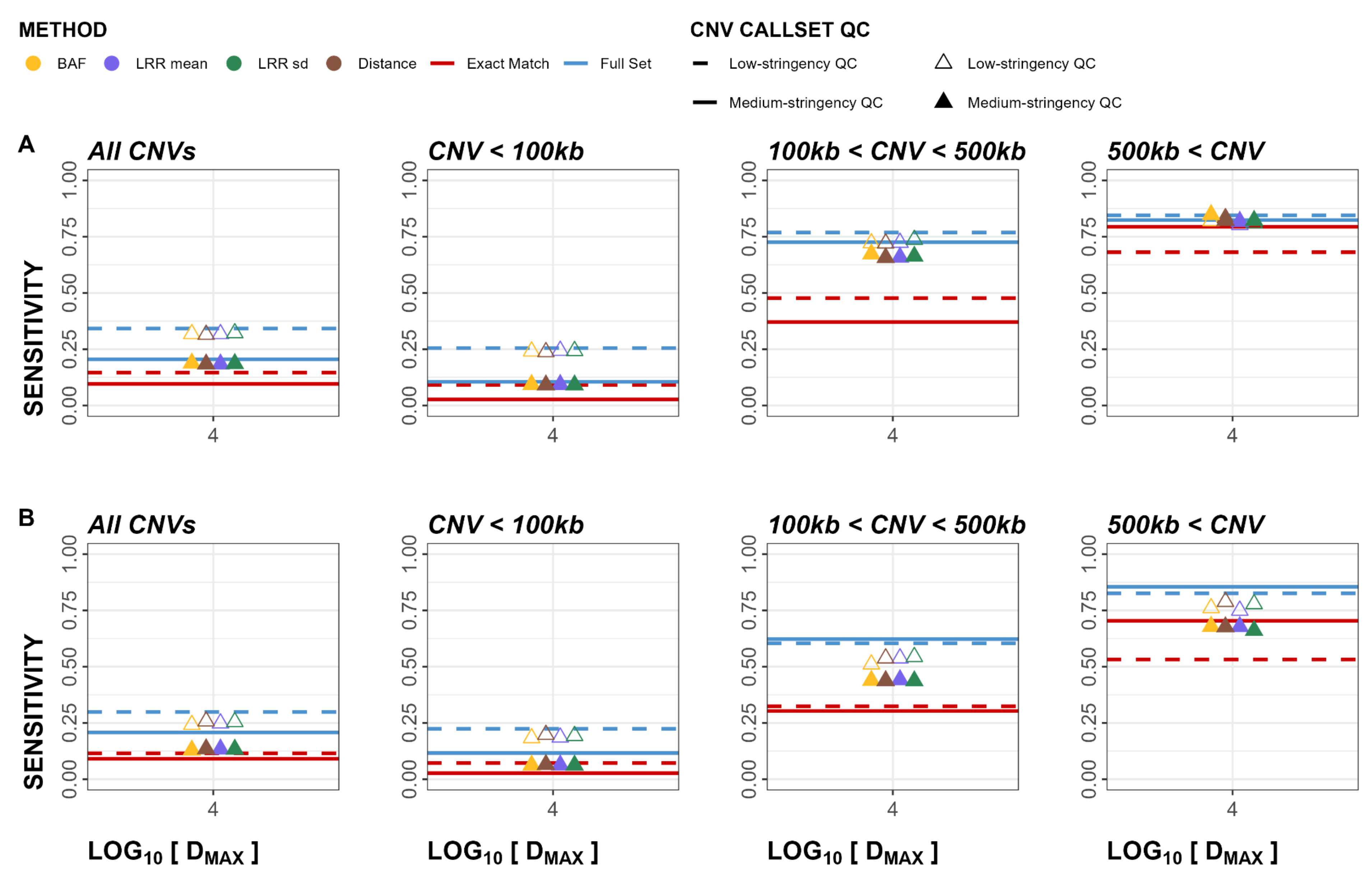

Figure S56. Cross-Array Experiment sensitivity plots for duplications. Panel A represents calls in GSA1validated in OEE. Panel B represents calls in OEE validated in GSA1. Empty triangles and dashed lines represent low-stringency QC callsets, whereas solid lines and filled triangles represent medium-stringency QC callsets. GSA1: Global Screening Array, OEE: Omni Express Exome array.

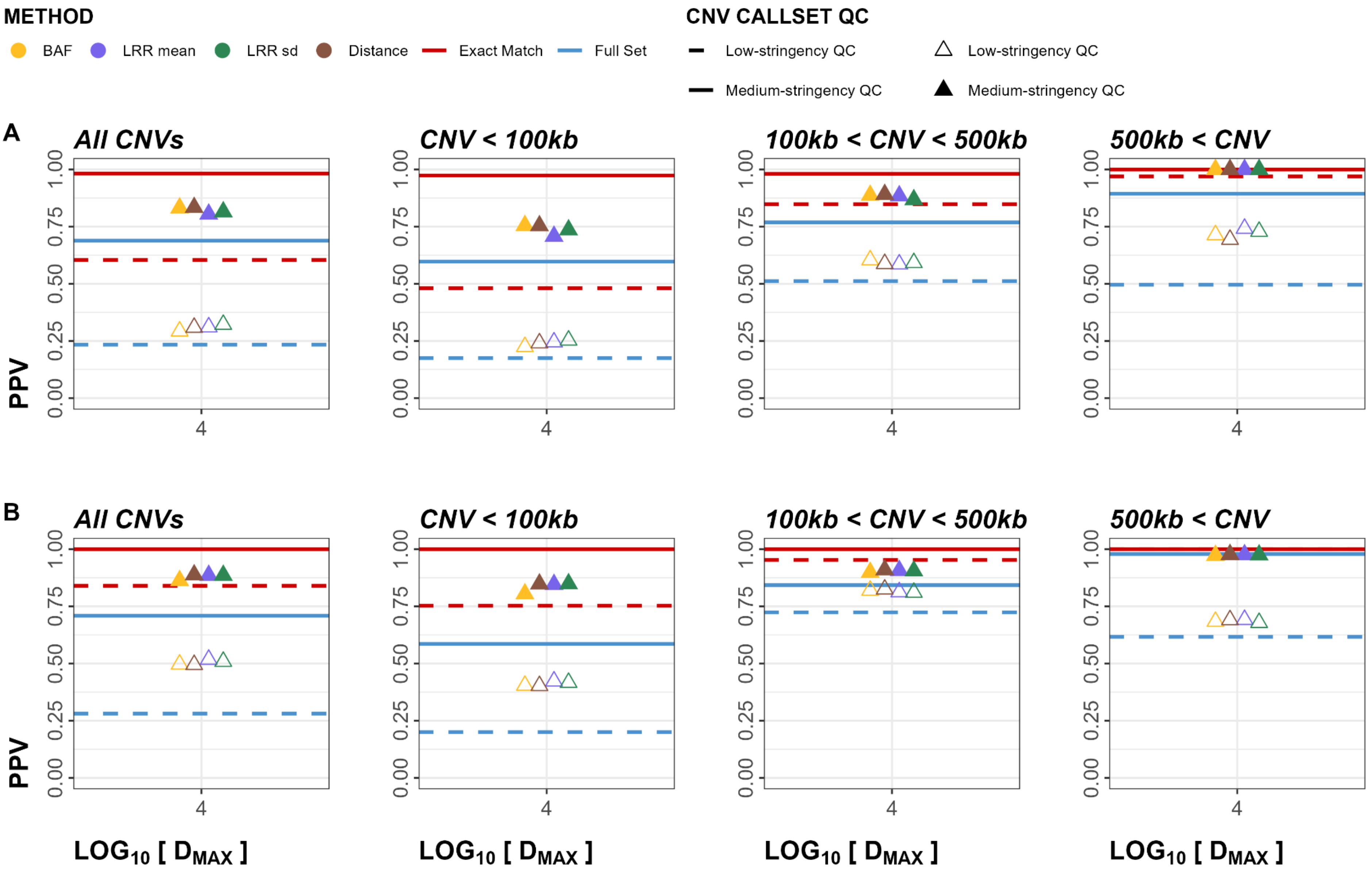

Figure S57. Cross-Array Experiment PPV plots for duplications. Panel A represents calls in GSA1validated in OEE. Panel B represents calls in OEE validated in GSA1. Empty triangles and dashed lines represent low-stringency QC callsets, whereas solid lines and filled triangles represent medium-stringency QC callsets. GSA1: Global Screening Array, OEE: Omni Express Exome array.

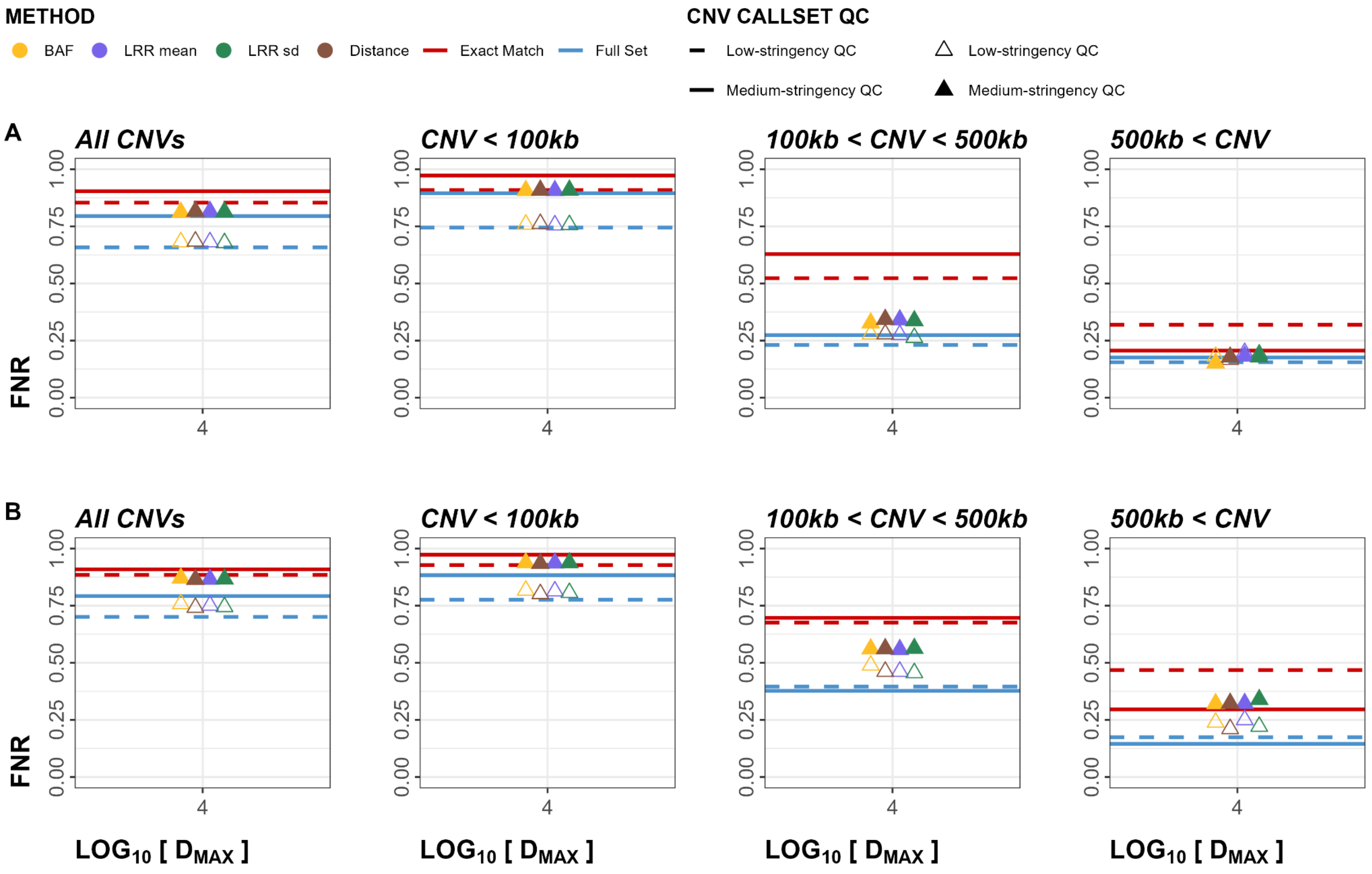

Figure S58. Cross-Array Experiment FNR plots for duplications. Panel A represents calls in GSA1validated in OEE. Panel B represents calls in OEE validated in GSA1. Empty triangles and dashed lines represent low-stringency QC callsets, whereas solid lines and filled triangles represent medium-stringency QC callsets. GSA1: Global Screening Array, OEE: Omni Express Exome array.

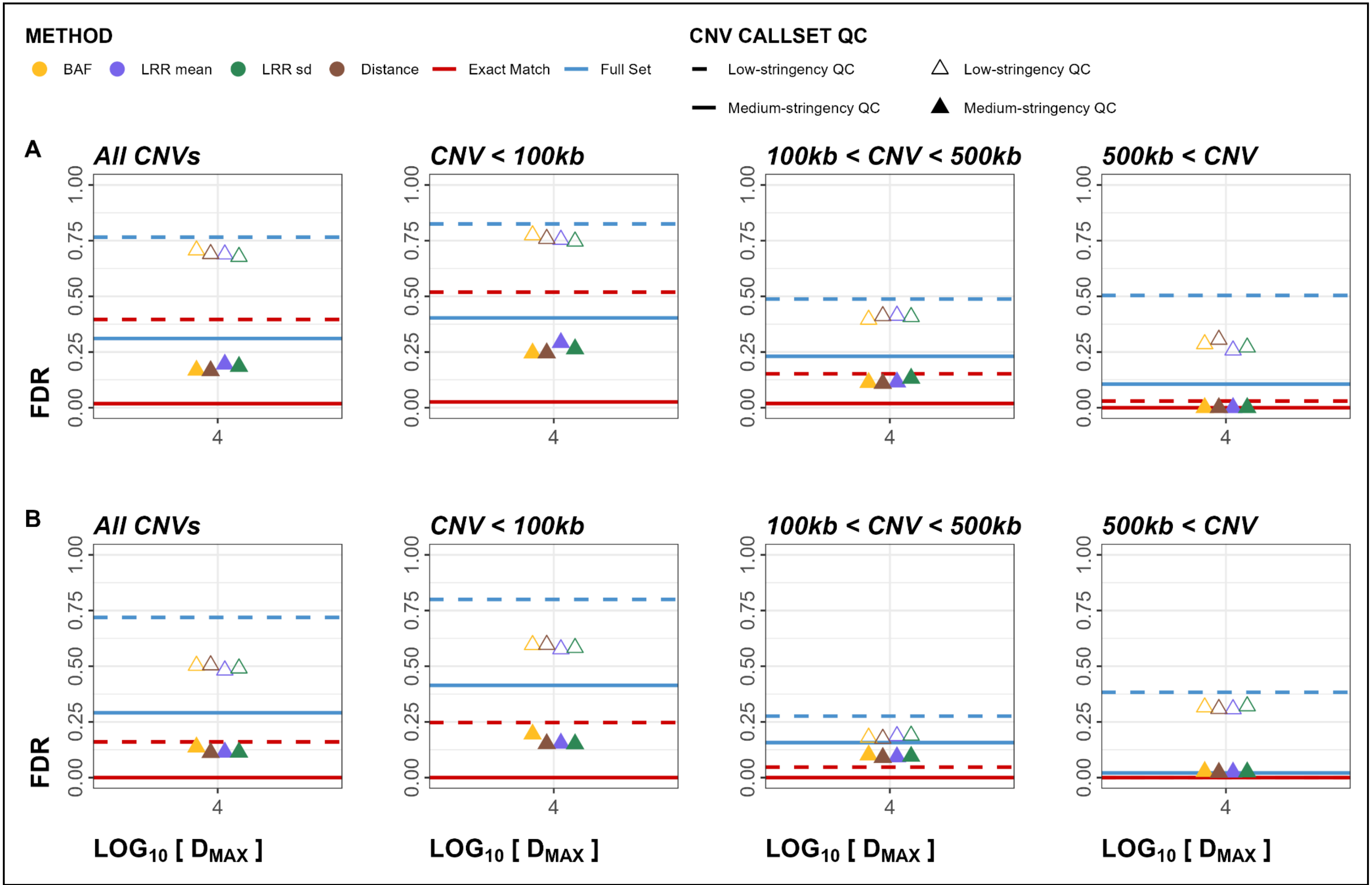

Figure S59. Cross-Array Experiment FDR plots for duplications. Panel A represents calls in GSA1validated in OEE. Panel B represents calls in OEE validated in GSA1. Empty triangles and dashed lines represent low-stringency QC callsets, whereas solid lines and filled triangles represent medium-stringency QC callsets. GSA1: Global Screening Array, OEE: Omni Express Exome array.

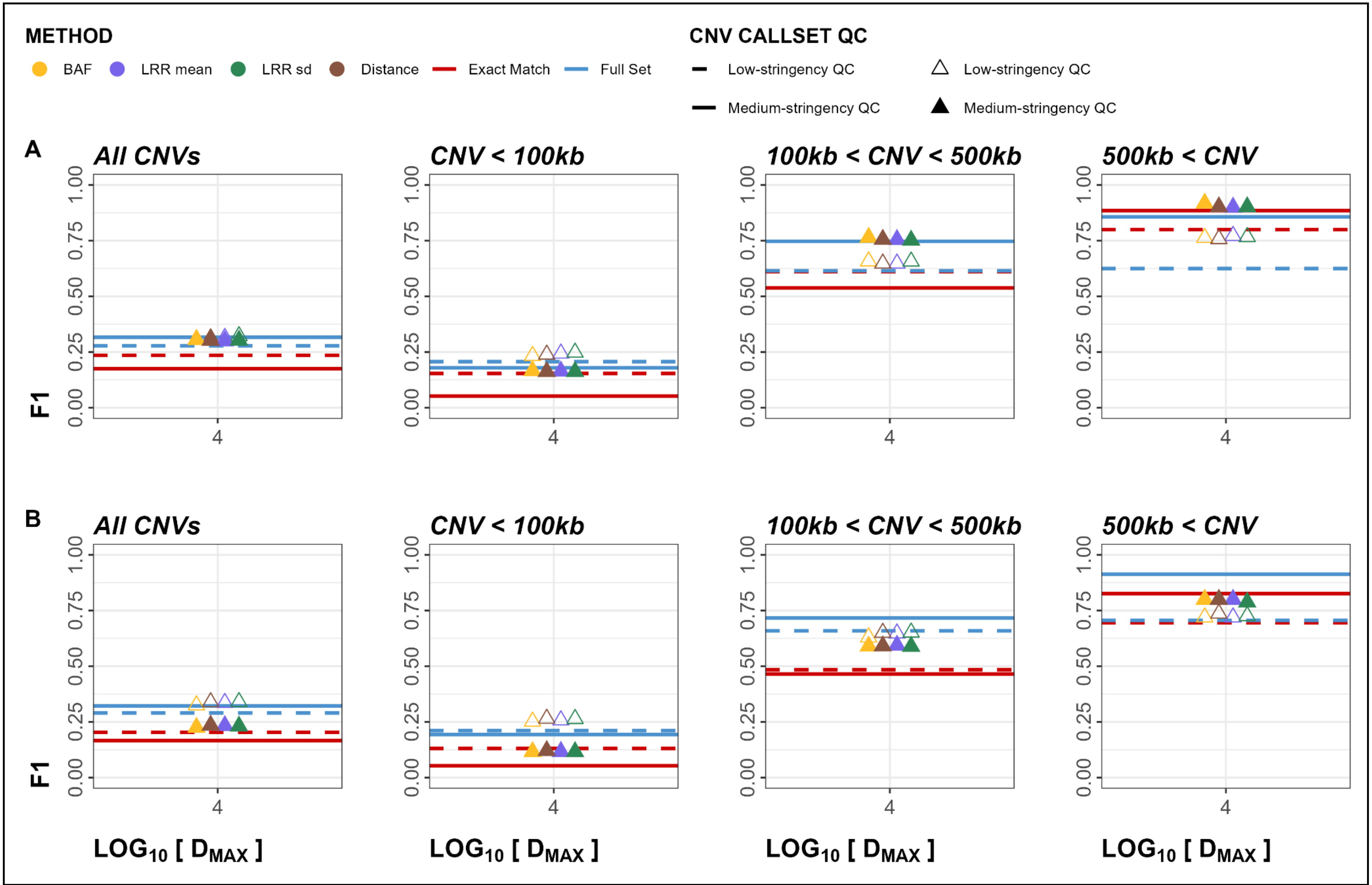

Figure S60. Cross-Array Experiment F1 plots for duplications. Panel A represents calls in GSA1validated in OEE. Panel B represents calls in OEE validated in GSA1. Empty triangles and dashed lines represent low-stringency QC callsets, whereas solid lines and filled triangles represent medium-stringency QC callsets. GSA1: Global Screening Array, OEE: Omni Express Exome array.

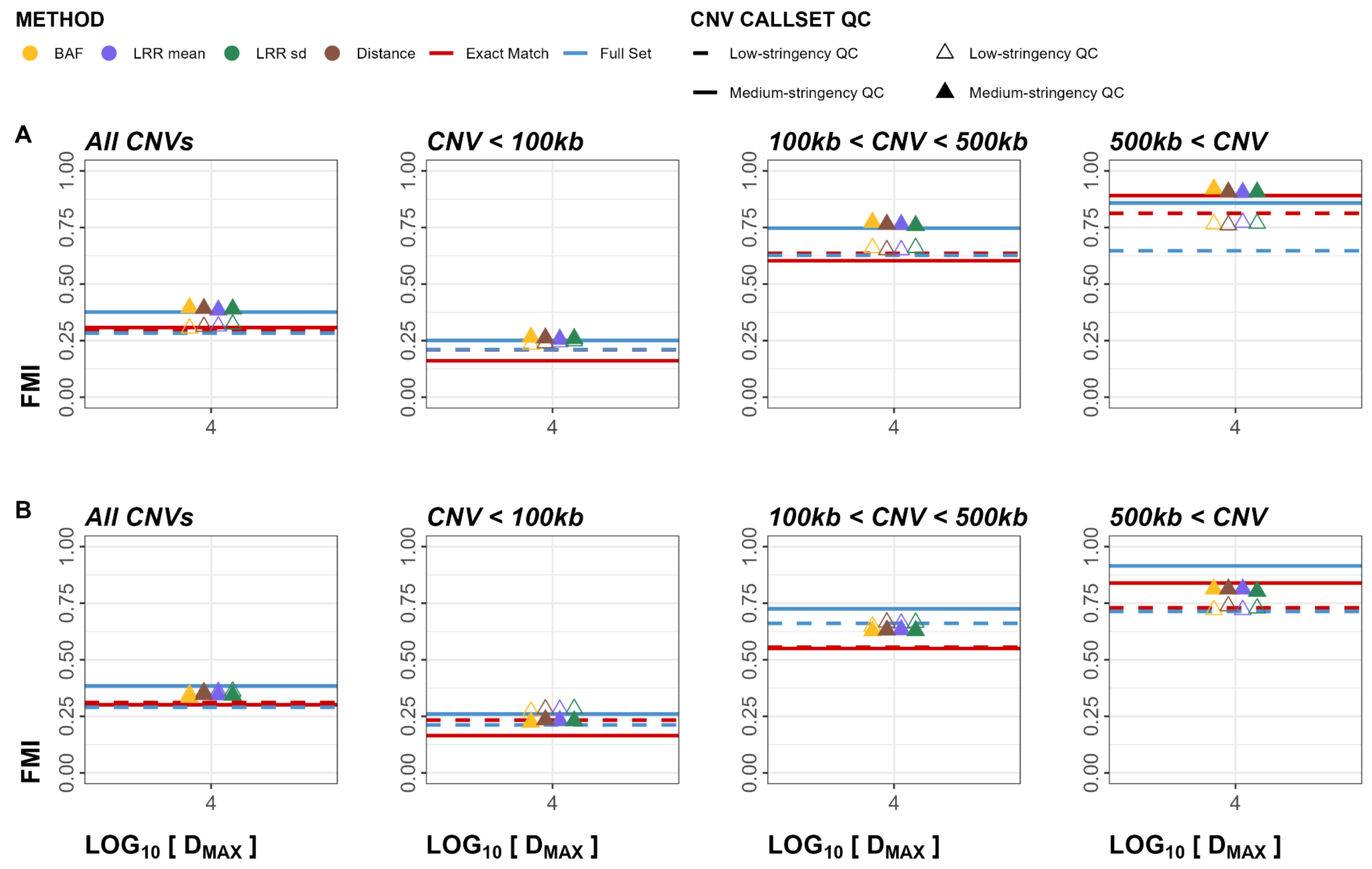

Figure S61. Cross-Array Experiment FMI plots for duplications. Panel A represents calls in GSA1validated in OEE. Panel B represents calls in OEE validated in GSA1. Empty triangles and dashed lines represent low-stringency QC callsets, whereas solid lines and filled triangles represent medium-stringency QC callsets. GSA1: Global Screening Array, OEE: Omni Express Exome array.

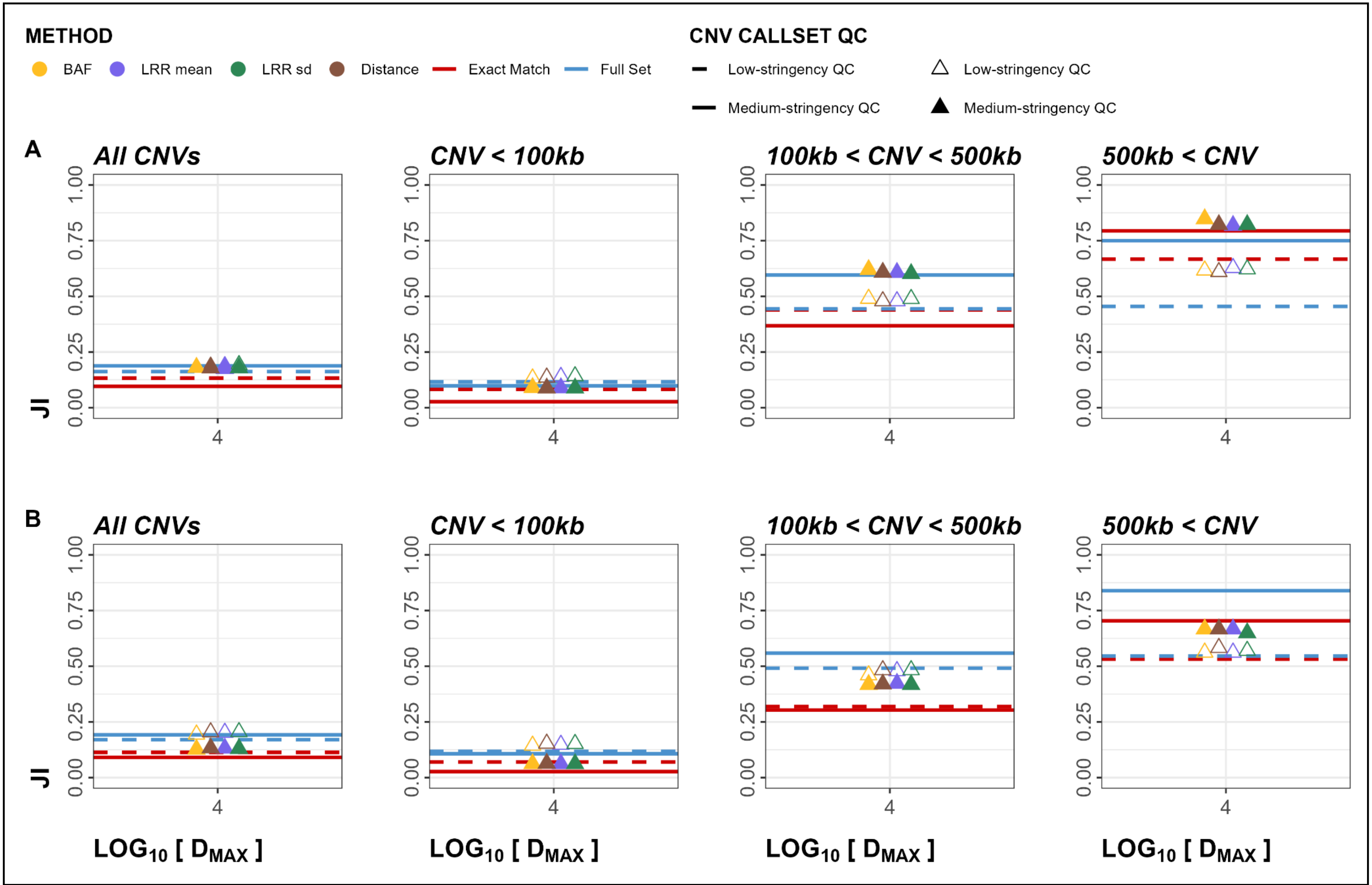

Figure S62. Cross-Array Experiment JI plots for duplications. Panel A represents calls in GSA1validated in OEE. Panel B represents calls in OEE validated in GSA1. Empty triangles and dashed lines represent low-stringency QC callsets, whereas solid lines and filled triangles represent medium-stringency QC callsets. GSA1: Global Screening Array, OEE: Omni Express Exome array.

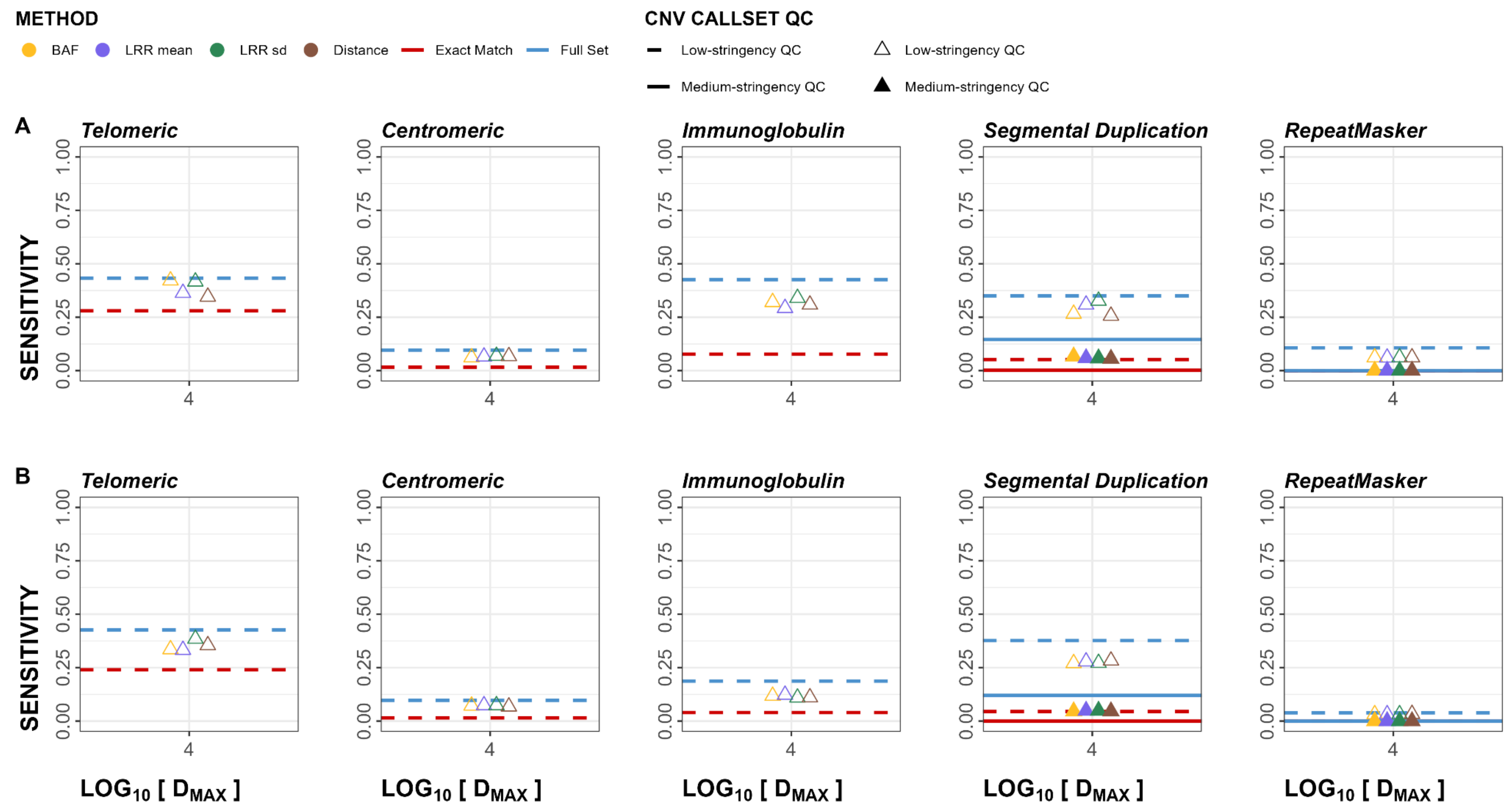

Figure S63. Cross-Array Experiment sensitivity regional plots for both deletions and duplications. Panels show telomeric, centromeric, immunoglobulin, segmental duplication, and repeat masker (simple repeat) regions in order, left to right. Empty triangles and dashed lines represent low-stringency QC callsets, whereas solid lines and filled triangles represent medium-stringency QC callsets. Panel A represents calls in Global Screening Array (GSA1) validated in Omni Express Exome (OEE) array. Panel B represents calls in OEE validated in GSA1.

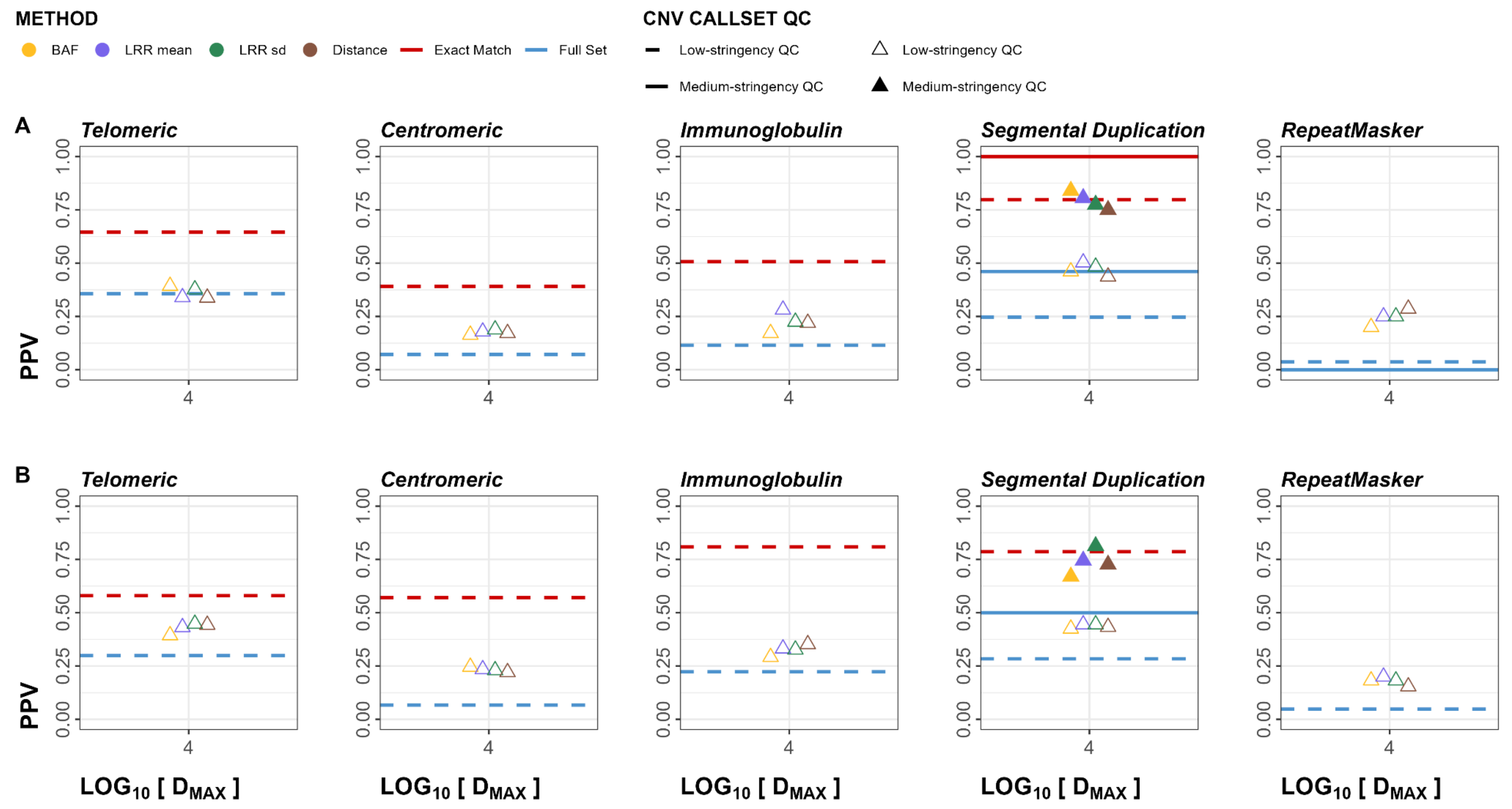

Figure S64. Cross-Array Experiment PPV regional plots for both deletions and duplications. Panels show telomeric, centromeric, immunoglobulin, segmental duplication, and repeat masker (simple repeat) regions in order, left to right. Empty triangles and dashed lines represent low-stringency QC callsets, whereas solid lines and filled triangles represent medium-stringency QC callsets. Panel A represents calls in Global Screening Array (GSA1) validated in Omni Express Exome (OEE) array. Panel B represents calls in OEE validated in GSA1.

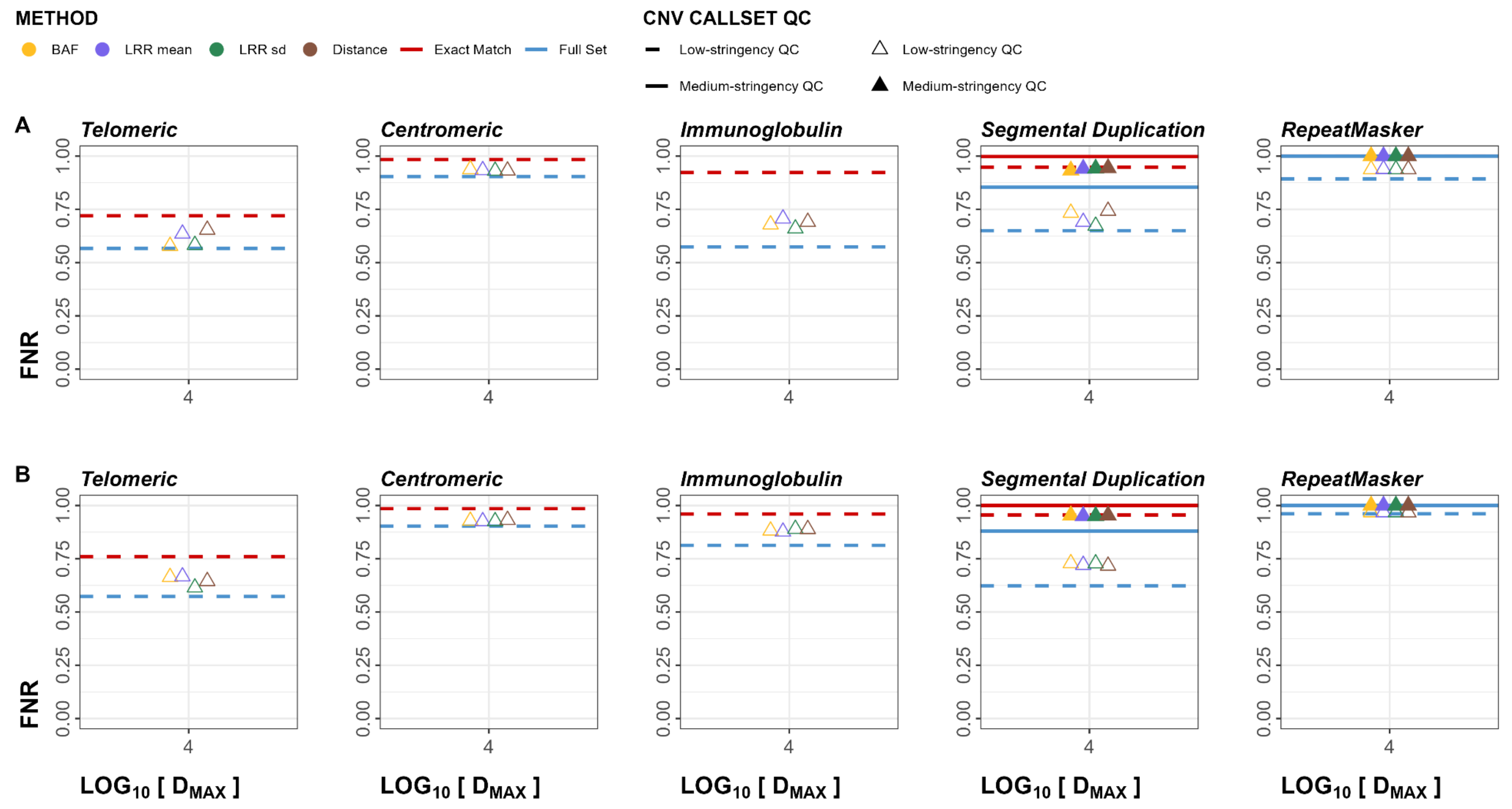

Figure S65. Cross-Array Experiment FNR regional plots for both deletions and duplications. Panels show telomeric, centromeric, immunoglobulin, segmental duplication, and repeat masker (simple repeat) regions in order, left to right. Empty triangles and dashed lines represent low-stringency QC callsets, whereas solid lines and filled triangles represent medium-stringency QC callsets. Panel A represents calls in Global Screening Array (GSA1) validated in Omni Express Exome (OEE) array. Panel B represents calls in OEE validated in GSA1.

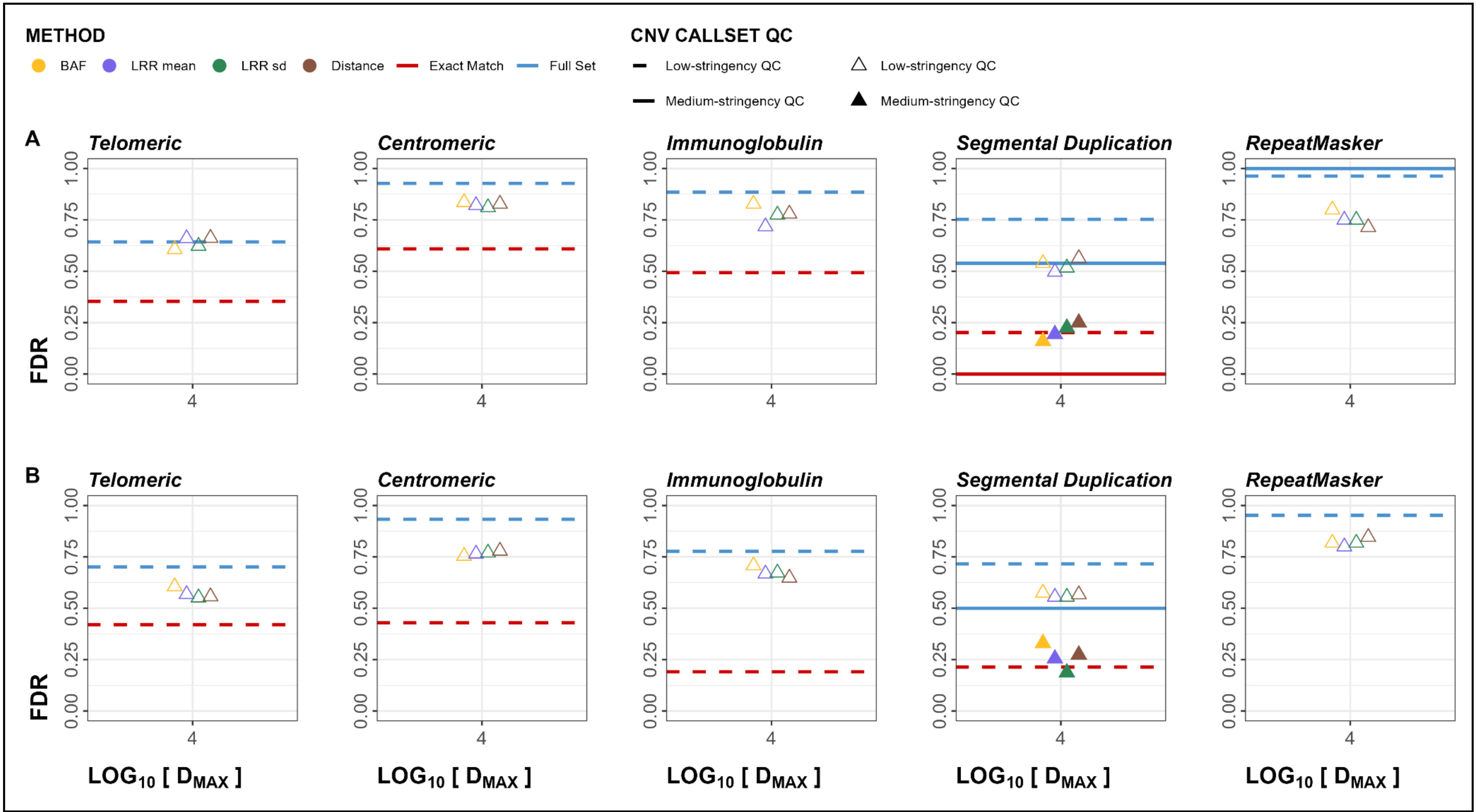

Figure S66. Cross-Array Experiment FDR regional plots for both deletions and duplications. Panels show telomeric, centromeric, immunoglobulin, segmental duplication, and repeat masker (simple repeat) regions in order, left to right. Empty triangles and dashed lines represent low-stringency QC callsets, whereas solid lines and filled triangles represent medium-stringency QC callsets. Panel A represents calls in Global Screening Array (GSA1) validated in Omni Express Exome (OEE) array. Panel B represents calls in OEE validated in GSA1.

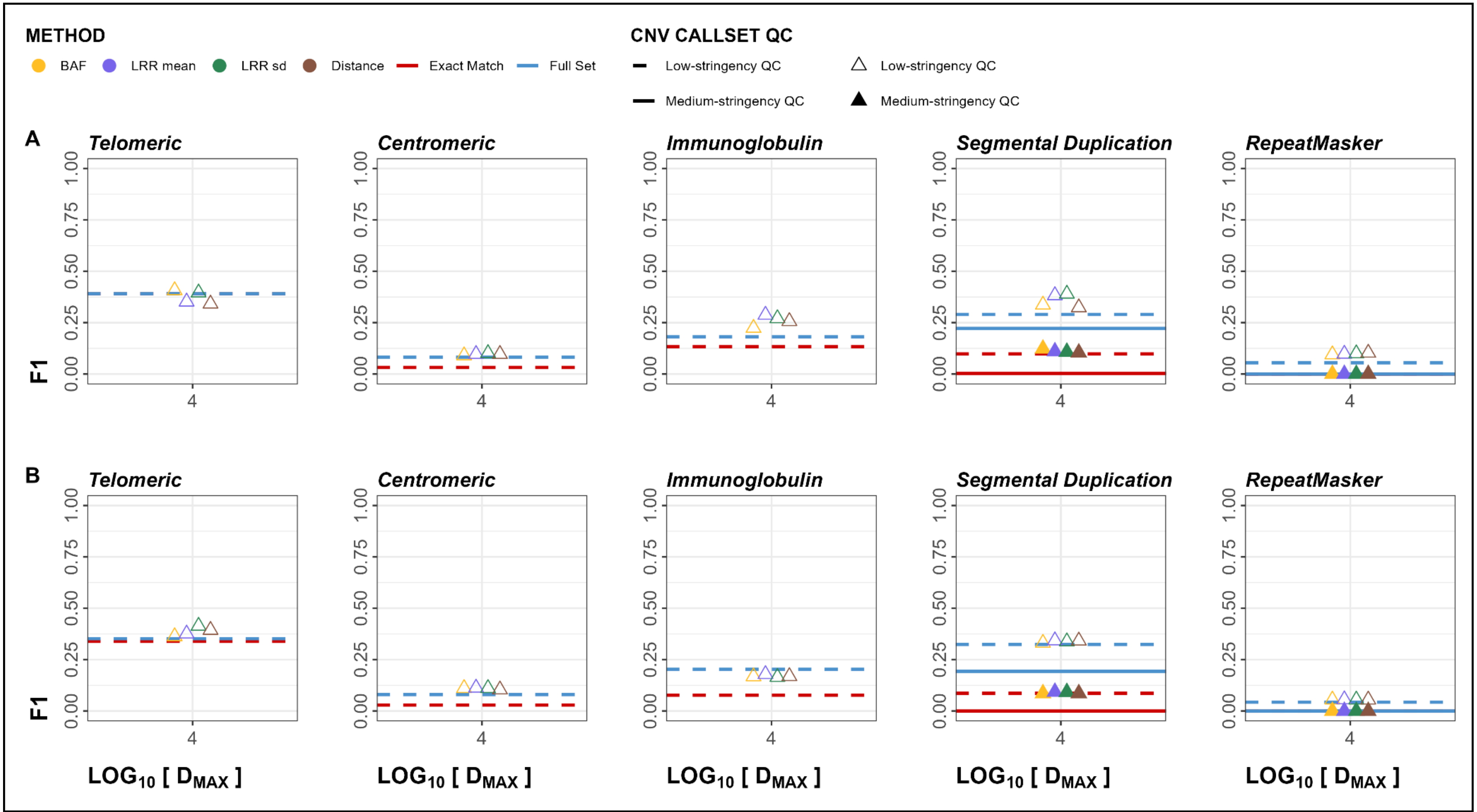

Figure S67. Cross-Array Experiment F1 regional plots for both deletions and duplications. Panels show telomeric, centromeric, immunoglobulin, segmental duplication, and repeat masker (simple repeat) regions in order, left to right. Empty triangles and dashed lines represent low-stringency QC callsets, whereas solid lines and filled triangles represent medium-stringency QC callsets. Panel A represents calls in Global Screening Array (GSA1) validated in Omni Express Exome (OEE) array. Panel B represents calls in OEE validated in GSA1.

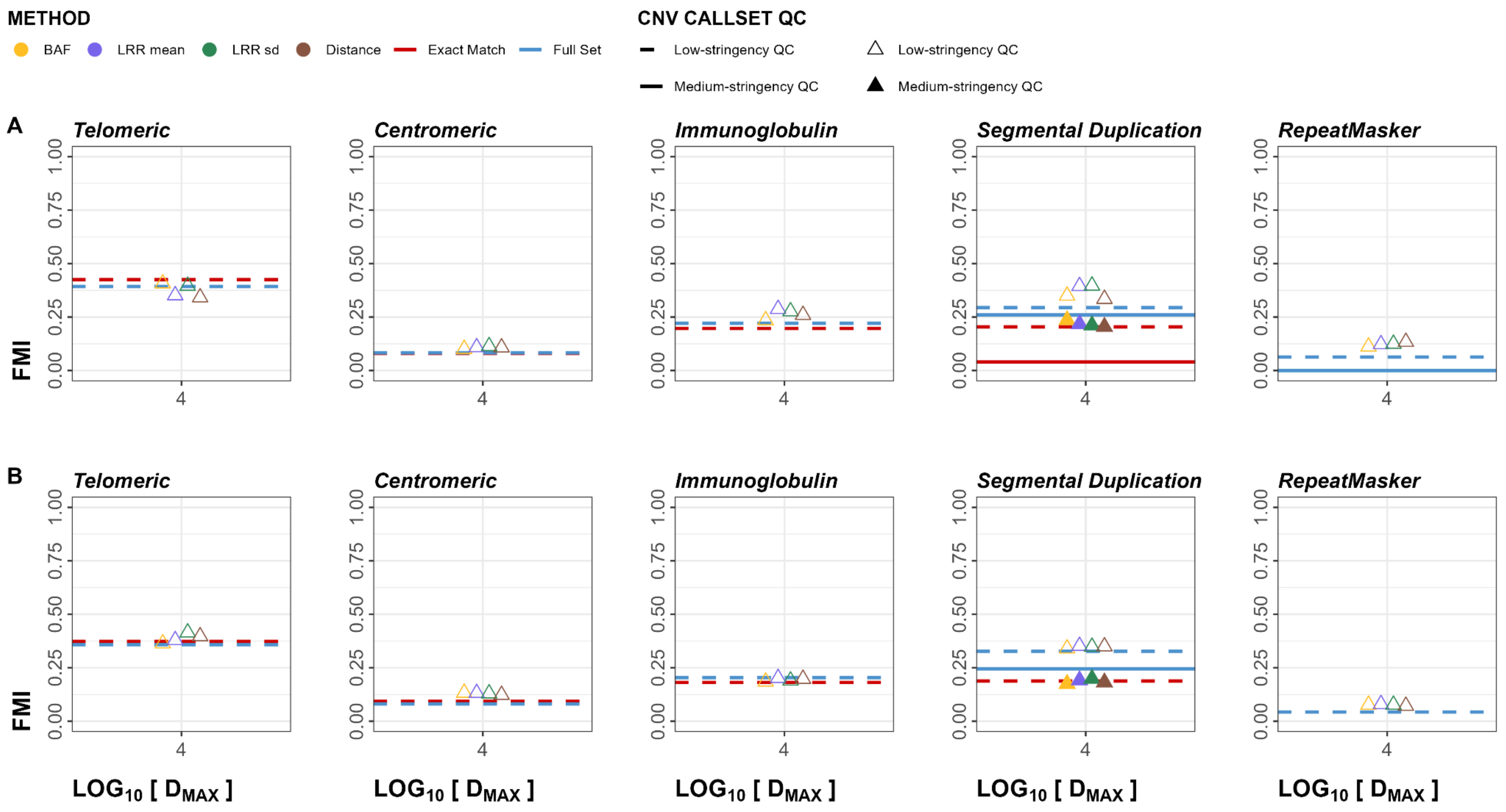

Figure S68. Cross-Array Experiment FMI regional plots for both deletions and duplications. Panels show telomeric, centromeric, immunoglobulin, segmental duplication, and repeat masker (simple repeat) regions in order, left to right. Empty triangles and dashed lines represent low-stringency QC callsets, whereas solid lines and filled triangles represent medium-stringency QC callsets. Panel A represents calls in Global Screening Array (GSA1) validated in Omni Express Exome (OEE) array. Panel B represents calls in OEE validated in GSA1.

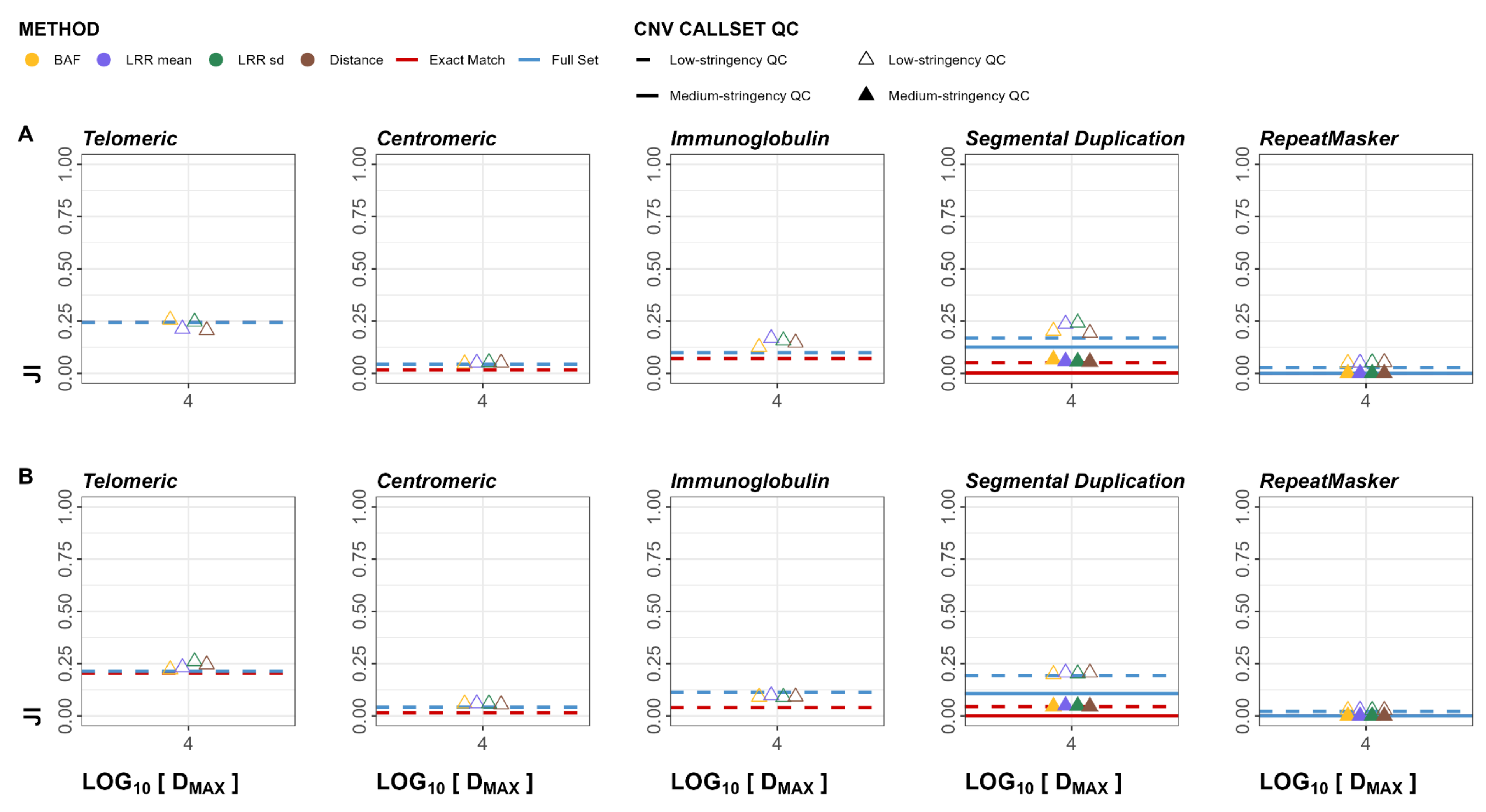

Figure S69. Cross-Array Experiment JI regional plots for both deletions and duplications. Panels show telomeric, centromeric, immunoglobulin, segmental duplication, and repeat masker (simple repeat) regions in order, left to right. Empty triangles and dashed lines represent low-stringency QC callsets, whereas solid lines and filled triangles represent medium-stringency QC callsets. Panel A represents calls in Global Screening Array (GSA1) validated in Omni Express Exome (OEE) array. Panel B represents calls in OEE validated in GSA1.

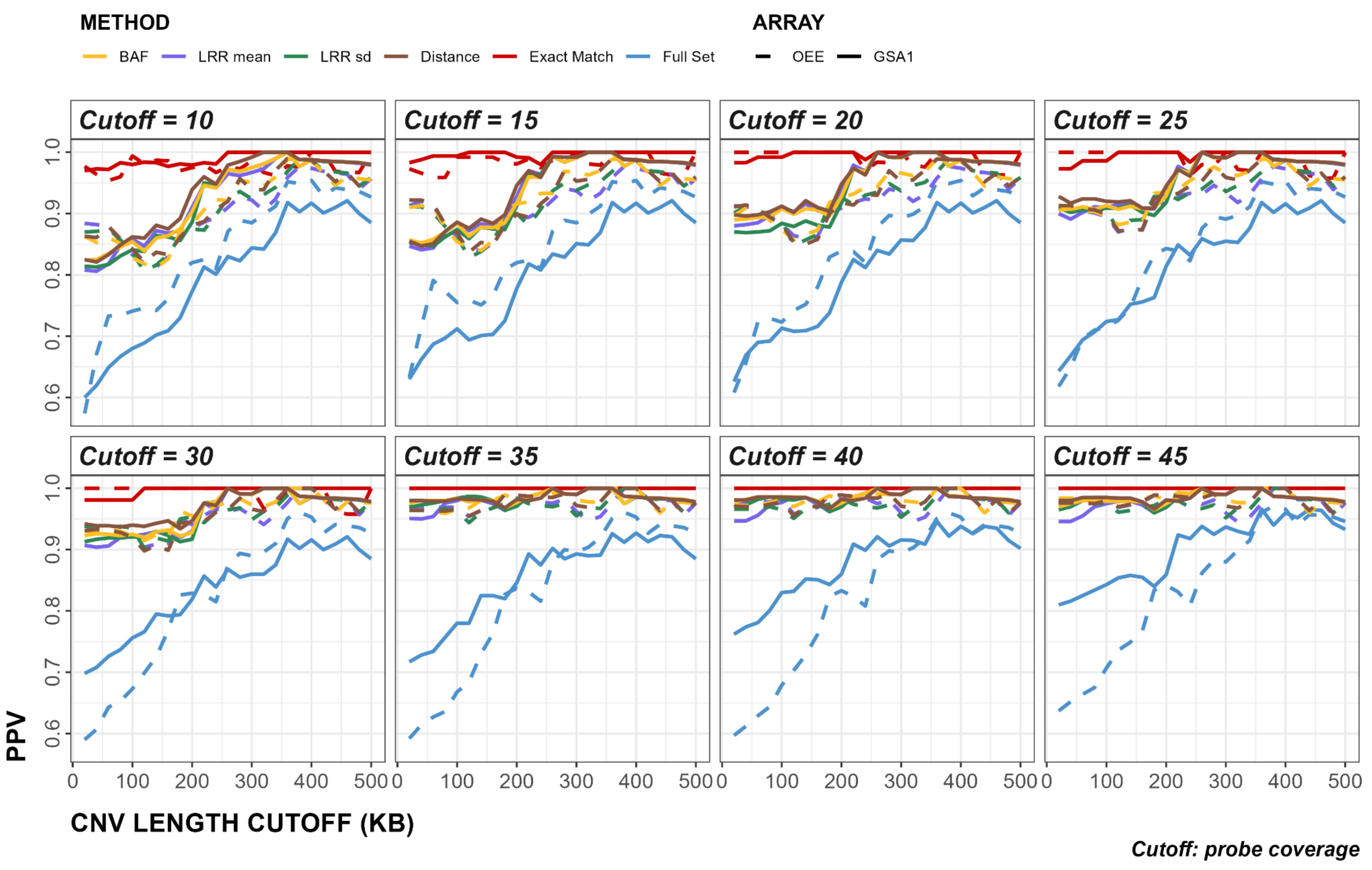

Figure S70. Cross-Array Experiment analysis of minimum cutoffs. Y-axes show PPV values, x-axes show minimum CNV length cutoffs, whereas each panel shows minimum marker coverage cutoffs. Solid and dashed lines show the results from GSA1 and OEE arrays, respectively. GSA1: Global Screening Array, OEE: Omni Express Exome array.

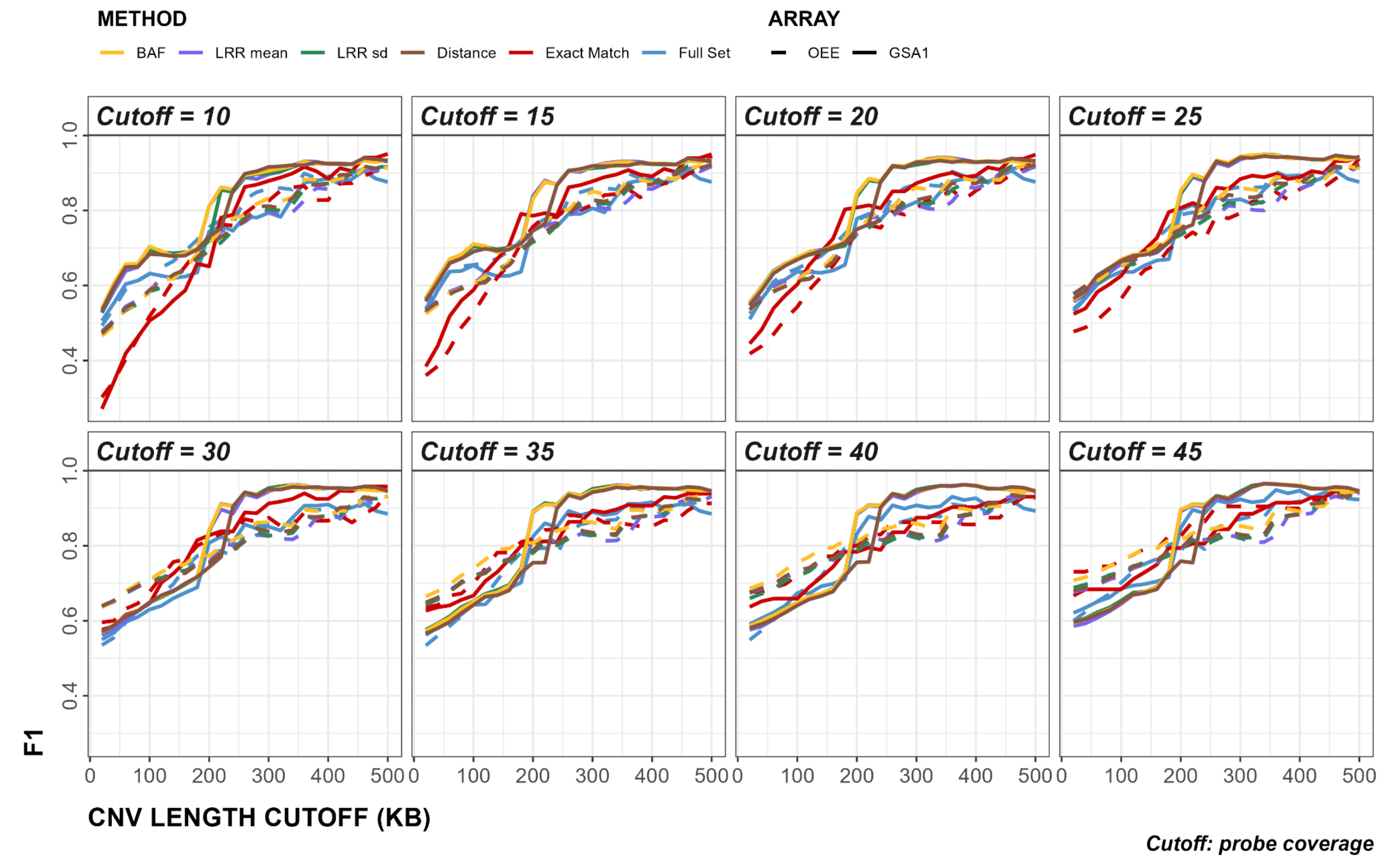

Figure S71. Cross-Array Experiment analysis of minimum cutoffs. Y-axes show F1 values, x-axes show minimum CNV length cutoffs, whereas each panel shows minimum marker coverage cutoffs. Solid and dashed lines show the results from GSA1 and OEE arrays, respectively. GSA1: Global Screening Array, OEE: Omni Express Exome array.

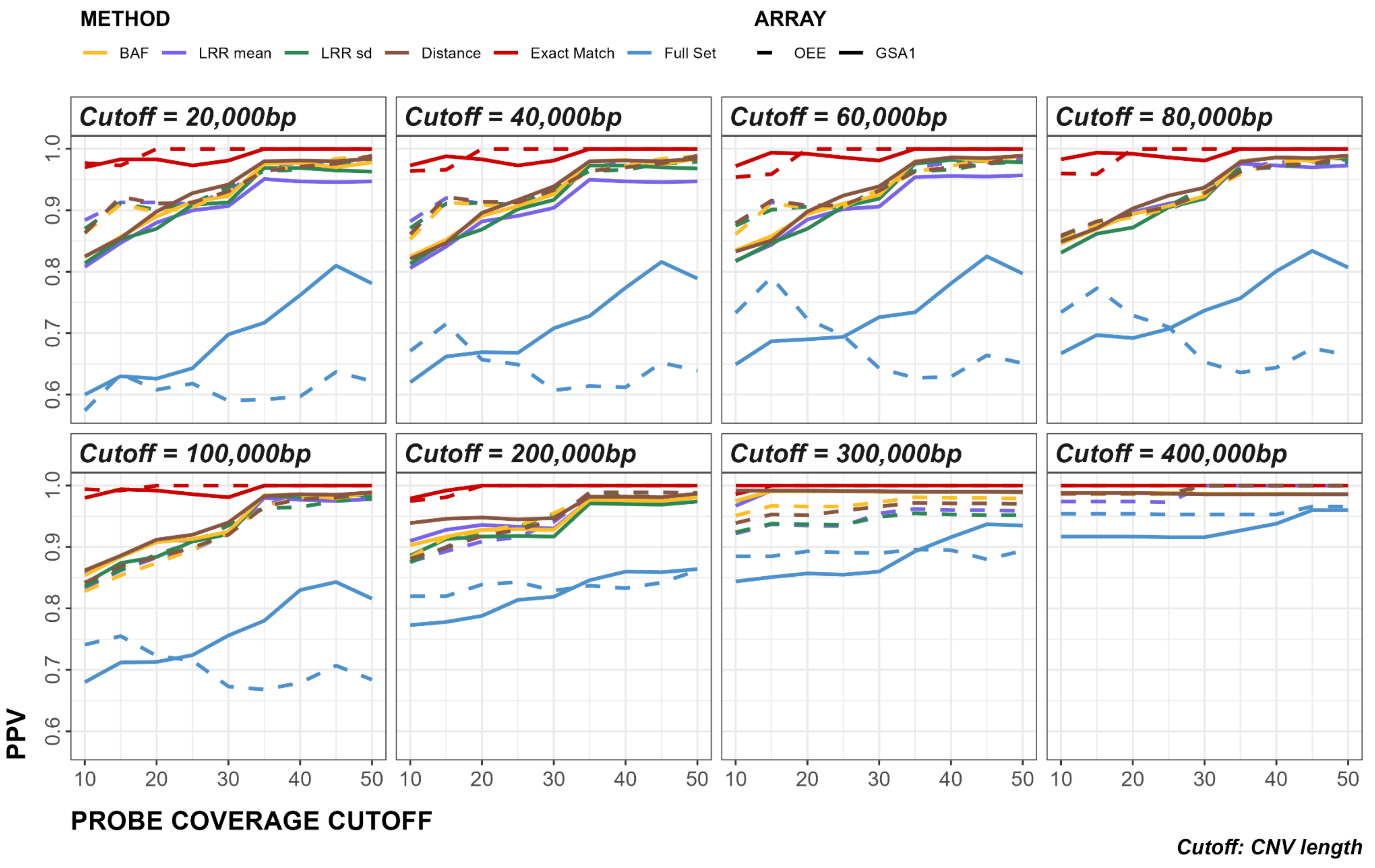

Figure S72. Cross-Array Experiment analysis of minimum cutoffs. Y-axes show PPV values, x-axes show minimum marker coverage cutoffs, whereas each panel shows minimum CNV length cutoffs. Solid and dashed lines show the results from GSA1 and OEE arrays, respectively. GSA1: Global Screening Array, OEE: Omni Express Exome array.

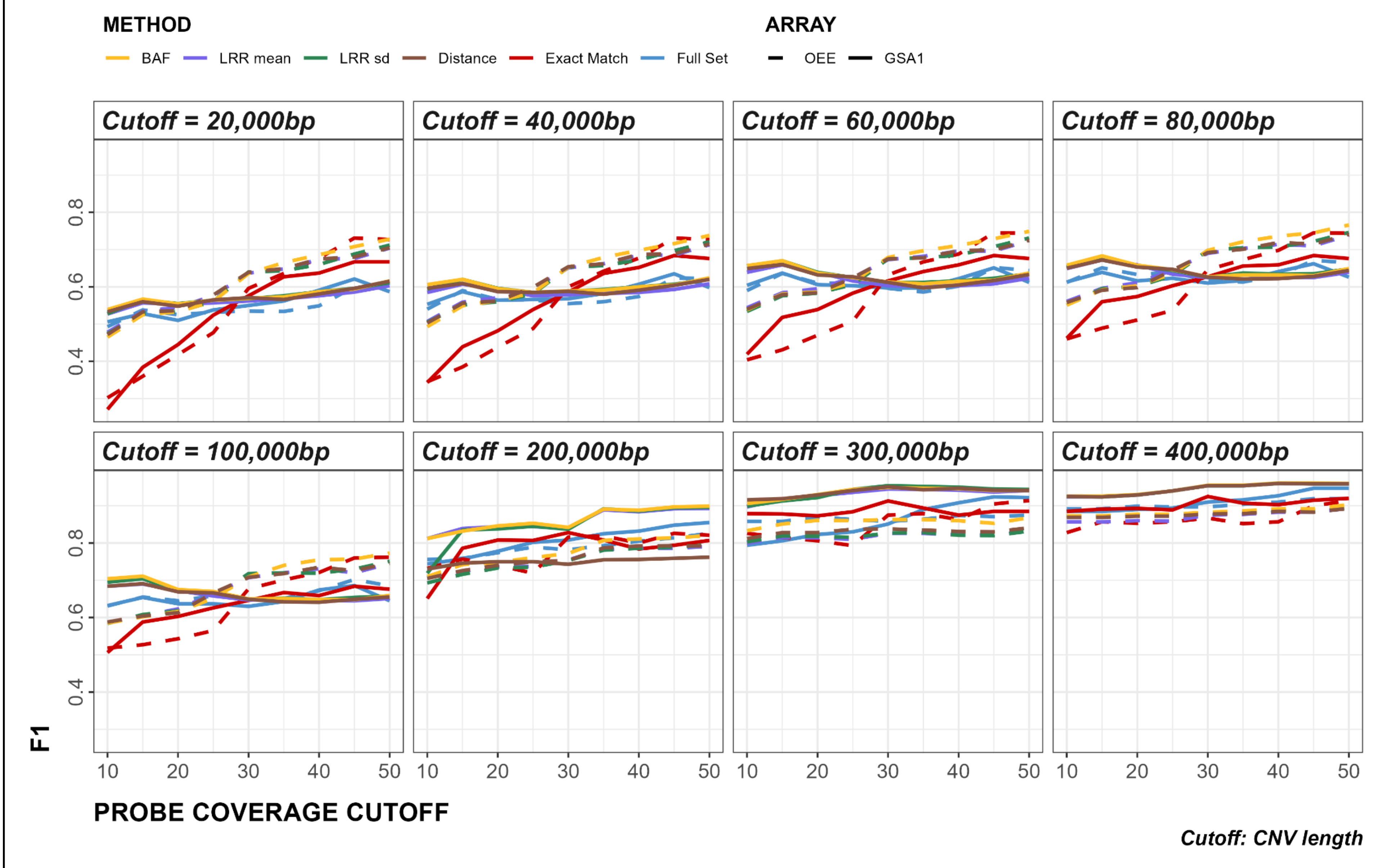

Figure S73. Cross-Array Experiment analysis of minimum cutoffs. Y-axes show F1 values, x-axes show minimum marker coverage cutoffs, whereas each panel shows minimum CNV length cutoffs. Solid and dashed lines show the results from GSA1 and OEE arrays, respectively. GSA1: Global Screening Array, OEE: Omni Express Exome array.

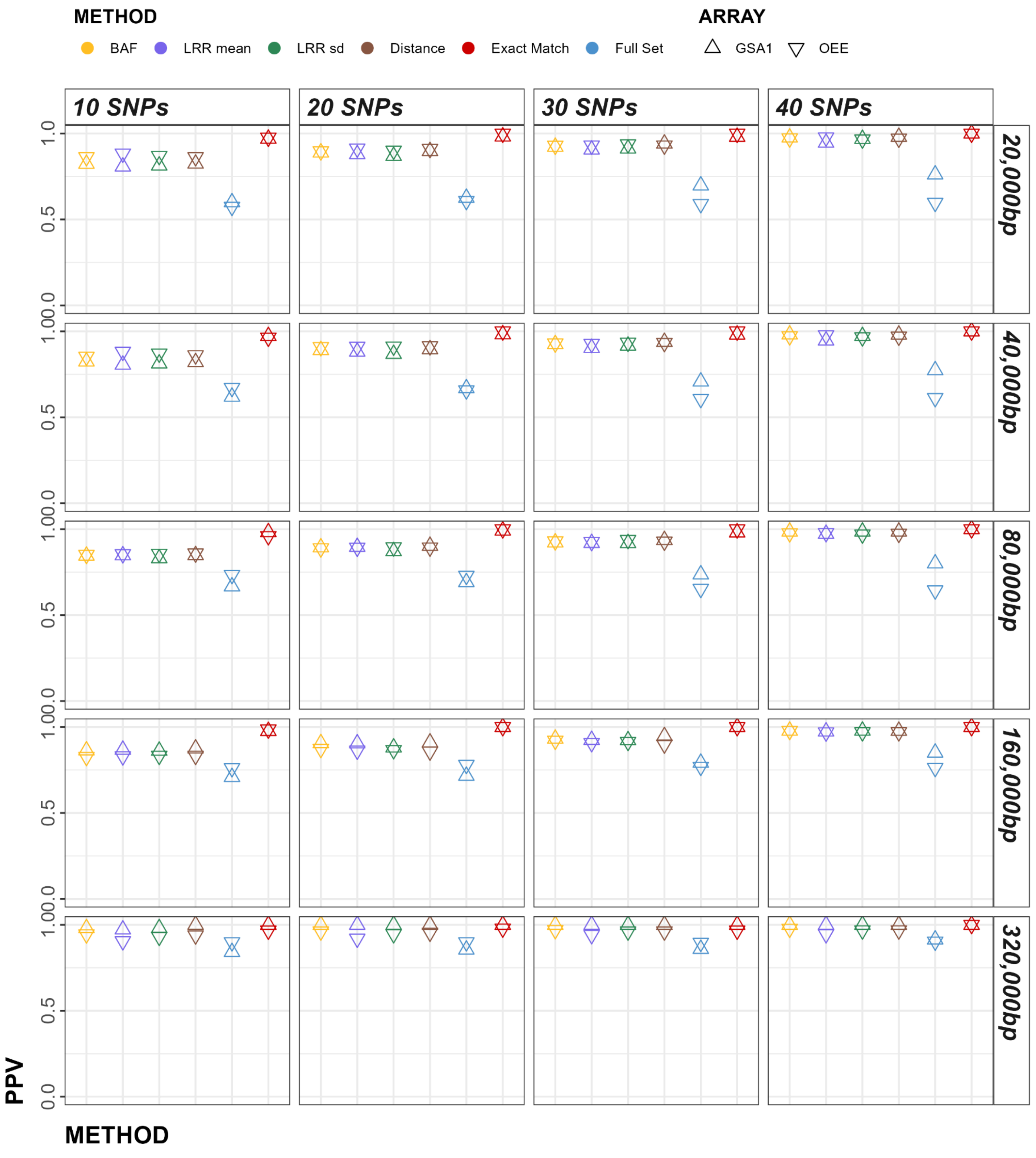

Figure S74. Cross-Array Experiment analysis of minimum cutoffs. Y-axes show PPV values, x-axes show individual Method parameters (also shown in color, Full Set and Exact Match included). Panels horizontally grow set marker coverage cutoffs, panels vertically grow in set CNV length cutoffs. GSA1 and OEE results are shown as triangles pointing up and down, respectively. GSA1: Global Screening Array, OEE: Omni Express Exome array.

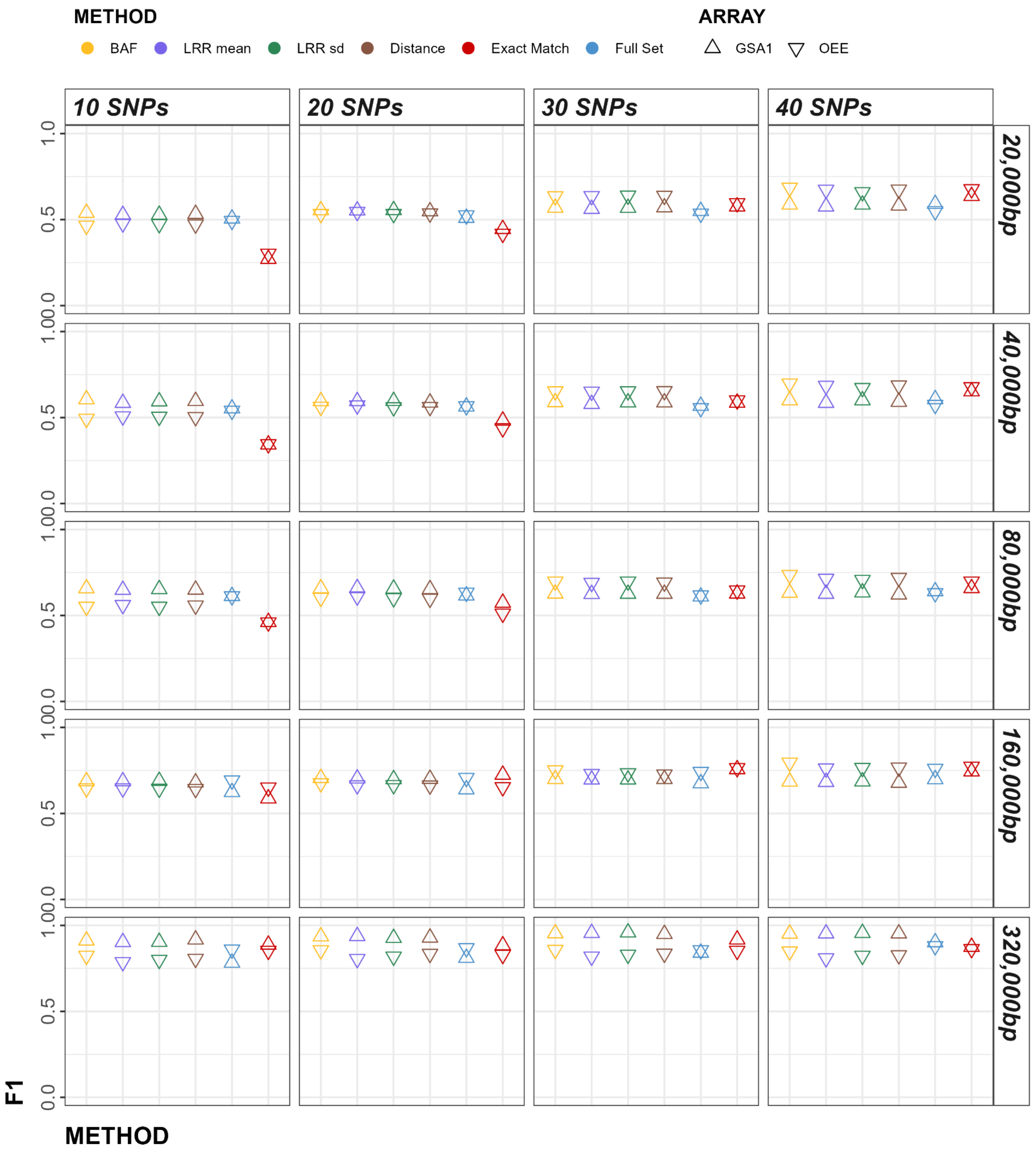

Figure S75. Cross-Array Experiment analysis of minimum cutoffs. Y-axes show F1 values, x-axes show individual Method parameters (also shown in color, Full Set and Exact Match included). Panels horizontally grow set marker coverage cutoffs, panels vertically grow in set CNV length cutoffs. GSA1 and OEE results are shown as triangles pointing up and down, respectively. GSA1: Global Screening Array, OEE: Omni Express Exome array.

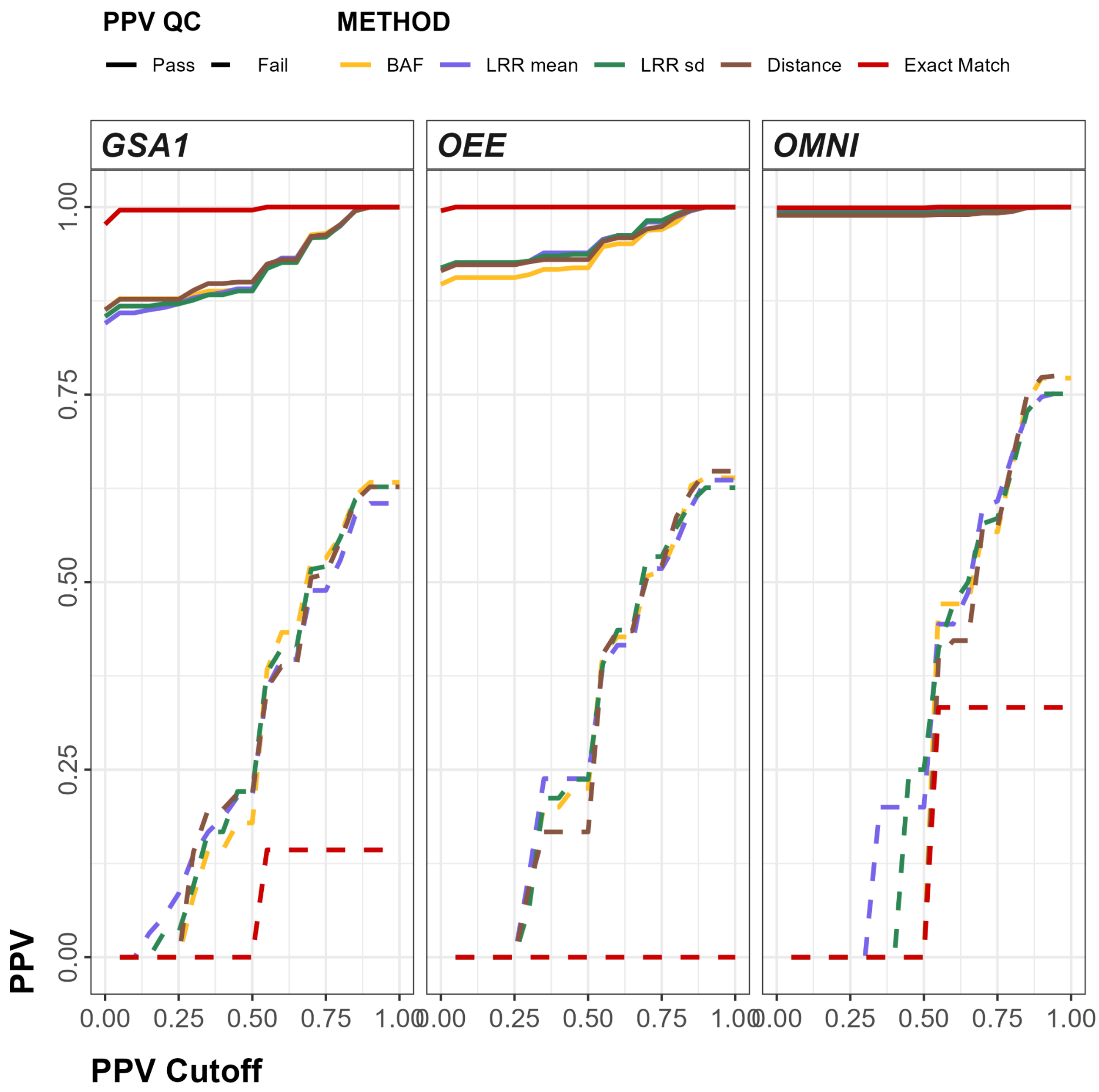

Figure S76. Callset PPV as a function of sample-wise PPV QC. The curves indicate callset PPV, after PPV QC. PPV QC passing (solid lines) and failing (dashed lines) along various PPV Cutoffs (ranging from 0 to 1), stratified by Method (color) and array (panels). GSA1: Global Screening Array, OEE: Omni Express Exome array, OMNI: Omni2.5 array.

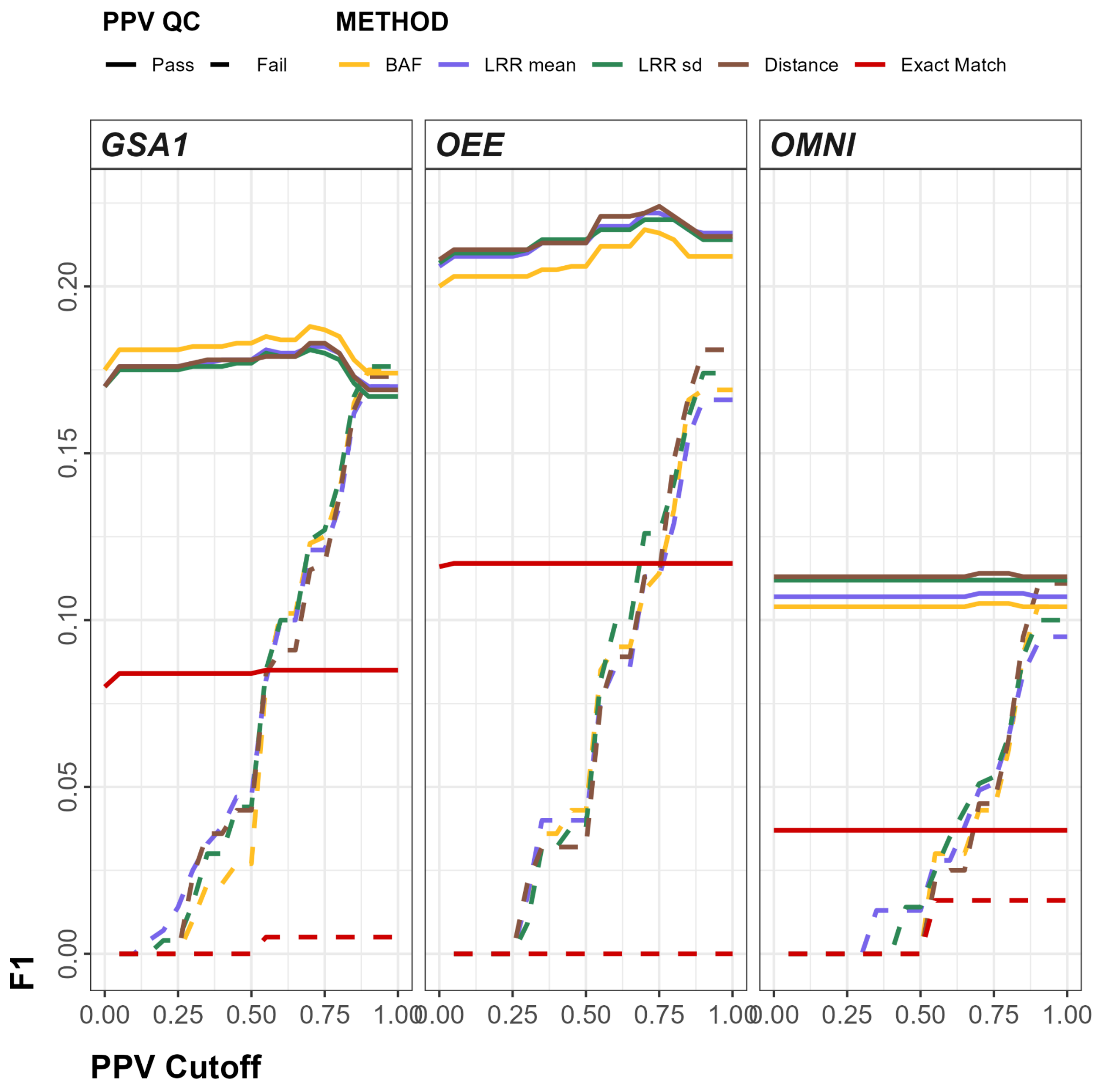

Figure S77. Callset F1 as a function of sample-wise PPV QC. The curves indicate callset F1, after PPV QC. PPV QC passing (solid lines) and failing (dashed lines) along various PPV Cutoffs (ranging from 0 to 1), stratified by Method (color) and array (panels). GSA1: Global Screening Array, OEE: Omni Express Exome array, OMNI: Omni2.5 array.
